# Comparison of Gene Expression Profile in Embryonic Mesencephalon and Neuronal Primary Cultures

**DOI:** 10.1371/journal.pone.0004977

**Published:** 2009-03-23

**Authors:** Dario Greco, Floriana Volpicelli, Antonio Di Lieto, Damiana Leo, Carla Perrone-Capano, Petri Auvinen, Umberto di Porzio

**Affiliations:** 1 Institute of Biotechnology, University of Helsinki, Helsinki, Finland; 2 Institute of Genetics and Biophysics, CNR, Naples, Italy; 3 Neuroscience Center, University of Helsinki, Helsinki, Finland; 4 University of Naples “Federico II”, Naples, Italy; University of Helsinki, Finland

## Abstract

In the mammalian central nervous system (CNS) an important contingent of dopaminergic neurons are localized in the substantia nigra and in the ventral tegmental area of the ventral midbrain. They constitute an anatomically and functionally heterogeneous group of cells involved in a variety of regulatory mechanisms, from locomotion to emotional/motivational behavior. Midbrain dopaminergic neuron (mDA) primary cultures represent a useful tool to study molecular mechanisms involved in their development and maintenance. Considerable information has been gathered on the mDA neurons development and maturation *in vivo*, as well as on the molecular features of mDA primary cultures. Here we investigated in detail the gene expression differences between the tissue of origin and ventral midbrain primary cultures enriched in mDA neurons, using microarray technique. We integrated the results based on different re-annotations of the microarray probes. By using knowledge-based gene network techniques and promoter sequence analysis, we also uncovered mechanisms that might regulate the expression of CNS genes involved in the definition of the identity of specific cell types in the ventral midbrain. We integrate bioinformatics and functional genomics, together with developmental neurobiology. Moreover, we propose guidelines for the computational analysis of microarray gene expression data. Our findings help to clarify some molecular aspects of the development and differentiation of DA neurons within the midbrain.

## Introduction

In the mammalian brain, dopaminergic (DA) neurons are mainly located in the ventral midbrain (mesencephalon, Mes) in which they are arranged in three distinct nuclei: substantia nigra (SN, A10), the ventral tegmental area (VTA, A9) and the retrorubral formation (A8). Neurons originating in the SN project abundantly to the dorsolateral striatum, forming the nigrostriatal pathway. DA neurons of the VTA project mainly to the ventromedial striatum, nucleus accumbens and frontal lobe, forming the mesocorticolimbic pathway. Although midbrain dopamine neurons (mDA) are relatively few (20000–40000 in the rodent), they play an important role in regulating several aspects of basic brain function. Alterations of development or survival, or impairment in DA signalling are involved in a variety of behavioural, movement, and psychiatric disorders. Specifically, nigrostriatal pathway has been implicated in Parkinson disease [1] and Huntington disease [2], as well as in drug abuse toxicity [3]. Instead, alterations in VTA outputs are involved in schizophrenia[4], depression [5], attention deficit hyperactive disorder [6] and addiction [7].

Analyses of mouse mutants defective in mDA development have highlighted several transcription factors contributing the specification of the neurotransmitter identity [8]–[10], as well as neuronal identity and maintenance [11], [12]. The molecular environment surrounding the mDA neurons plays an important role for their differentiation. Cooperative signalling by Sonic Hedgehog (SHH) from the floor plate and fibroblast growth factor (FGF) 8 from the isthmus induces mDA [13]–[16].

The mDA neuronal primary cultures represent a valuable tool to investigate the molecular mechanisms involved in the development and maintenance of these neurons. The expanded mDA cultures are generated from E11.5 rat ventral Mes, when a large number of mDA precursors are present [17]. Previously, we have demonstrated that the addition of the FGF 2 (also known as basic FGF) from the beginning of the culture in serum-free medium induces neuroblasts proliferation [18], [19]. Furthermore mDA differentiation is increased in the culture treated with SHH and FGF8. As *in vivo*, also *in vitro* these factors are the inductive signals that specify mDA phenotype [14], [19], [20]. Markers of differentiated mDA neurons are observed in primary cultures when FGF2, SHH and FGF8 are withdrawn after six days *in vitro* (DIV) and ascorbic acid is added. Particularly, TH immunostainings show a high number of mDA neurons. In fact at this time *in vitro*, the number of TH^+^ cells is increased about 20 folds when compared to 3 DIV cultures [19] and comparable to the number of mDA neurons present in primary cultures with or without serum plated at a 20-fold higher cell concentration [21], [22]. These expanded cultures also show the presence of both neuroblasts, as shown by nestin-immunoreactivity, as well as more mature neurons. At least 90% of the expanded cells are nestin-positive, and at least 70% are medium neurofilament-positive (NFM). Thus there is a co-localization of nestin and NFM in at least 30% of the cells. Cells expressing glial markers, such as the glial fibrillary acid protein (GFAP), are extremely rare (on average one or two GFAP-positive cells/well at 9 DIV) [19], [23]. Glutamatergic and GABAergic neurons are also present, as indicated by the levels of the glutamic acid decarboxylases (GAD65, GAD67 and EP10) and glutamate transporter (EAAT1) mRNAs. Instead serotonergic and noradrenergic neurons are absent, as indicated by lack of 5HT (serotonine transporter, SERT and tryptophan hydroxylase, TrpH) and NE (noradrenaline transporter, NET) markers, respectively [23]. In these cultures, mDA neurons are well differentiated since all molecular mDA markers, such as tyrosine hydroxilase (TH), (Dat), Nr4a2 (also known as Nurr1), and vesicular monoamine transporter 2 (Vmat-2), tyrosine kinase receptor Ret (Ret), GDNF family receptor alpha 1–2 (GFRalpha 1–2) are expressed [17]. Moreover, they also show mature functional signature since high affinity uptake, a marker of mature DA function, is present at 9 DIV. The latter is specific since it is blocked in the presence of selective dopamine uptake inhibitors but not by serotonergic and noradrenergic uptake inhibitors [23]. However, there is a lack of general picture concerning the global gene expression program of these cultures as compared to the tissue of origin.

We have carried out extensive gene expression survey of rat E11.5 mesencephalon (MesE11) and Mes primary culturesPC (MesPC) at 9 DIV generated from it. For this, a microarray experiment using the Affymetrix GeneChips RAE230A has been carried out. Because of the inaccuracies in the original annotation, we have performed the computational analysis by re-annotating all the probes present on the RAE230A chipset according to three major sequence databases, such as Entrez Gene [24], RefSeq [25], and Ensembl gene [26]. The results have been then combined in order to create a robust set of result for further analysis. Finally, by using computational techniques of gene networks and promoter sequence analysis, we have uncovered possible regulatory modules responsible for the expression of multiple genes both in MesE11 and MesPC.

## Results

**Figure 1:**
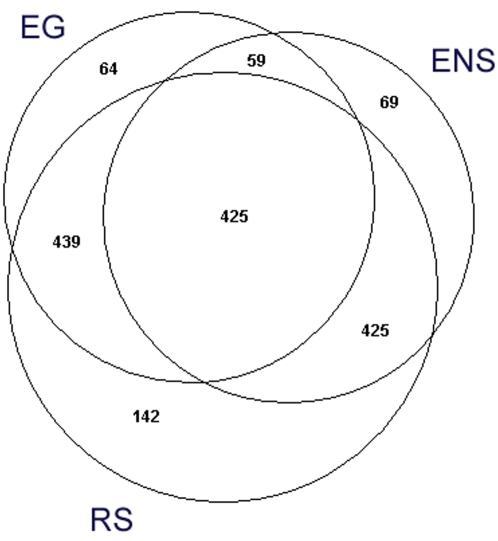
Significant genes with an annotation in DAVID database. The numbers of significant genes with a reliable DAVID annotation in each re-annotation analysis are shown. The intersections show the amount of common genes between two or all the three analyses. Proportional-area Venn diagrams were built as suggested by Chow and Rodgers [55].

In order to characterize the expanded mesencephalic neuronal primary cultures and their expression profiling, as well as to identify new genes involved in mDA neurons differentiation and maturation, we performed the microarray analysis. To fulfill our aim we used three independent re-annotation systems based on the alignment of each oligonucleotide probe present on the RAE230A chipset against the Entrez Gene (EG), RefSeq (RS), and Ensembl gene (ENS) databases. Based on the new identities for probes we performed three independent analyses abbreviated from now on as EG, RS, and ENS. According to the original annotation released by the manufacturer, the RAE230A chipset contains 15923 probe sets; after re-annotation the probes were re-arranged in 8676 (EG), 13224 (RS), and 7600 (ENS) probe sets. After moderated t-test, we obtained 1016 genes in EG (11.7% of all the screened genes), 1568 (9.8% of all the screened genes) genes in RS, and 862 (11.3% of all the screened genes) genes in ENS to be differentially expressed between MesE11 and MesPC with p-value<0.001 (Table 1). We annotated the gene lists using DAVID which is a gene-centered database where gene entities from several databases are uniquely stored [27]. We took advantage of this system for parsing the gene lists from the three analyses. As many as 987 genes from EG, 1038 genes in RS, and 567 genes in ENS respectively had a reliable DAVID identifier. Of these, 425 unique genes were shared between the three annotations (Figure 1). The RefSeq-based set showed the largest overlapping with the other two annotation systems. From the set of 425 common genes, 268 genes were upregulated in MesPC, and 157 were upregulated in MesE11 (Table S1). Functional classification of these groups of genes was carried out with the DAVID-based Fisher's exact test. Overall, the three annotations produced very consistent fold change estimation for all of the 425 common genes (average standard deviation = 0.04).

**Table 1:** Summary of the statistical analysis.

	EG	RS	ENS
Total genes analyzed (a)	8676	13224	7600
Significant genes (% of tot) (b)	1016 (11.7%)	1568 (9.8%)	862 (11.3%)
Significant genes with annotation (% of tot significant) (c)	987 (97%)	1038 (66.2%)	567 (65.7%)

In the columns are shown the three re-annotations used for analyzing the dataset (Entrez Gene (EG), RefSeq (RS), and Ensembl gene (ENS)). The absolute number of genes analyzed (a), found significant (b), and with an annotation in DAVID database (c) is shown. In parenthesis, the percent of all the genes analyzed is also shown.

### Genes upregulated in midbrain neuronal expanded cultures

Five functional categories were dominating among the 268 genes over-expressed in MesPC: developmental process (95 genes), lipid metabolic process (34 genes), mitochondrion (29 genes), extracellular matrix (22 genes), and lysosome (15 genes). Additionally, neurogenesis (16 genes) and neuron differentiation (14 genes) were also significantly over-represented. Interestingly, 10 genes coding for collagens were among this group of genes with high fold change (Table S2).

Microarrays are capable to observe changes in the expression of transcripts providing no explanation on how this is modulated within the cells. Gene transcription is also regulated by proteins that recognize short DNA sequence motifs, called transcription factor binding sites (TFBSs). TFBSs are in most cases located in the promoter regions of the genes. Similar TFBSs patterns within the promoters of transcripts are expressed in the same tissue under similar conditions. Thus, the organization of promoter motifs represents a framework of the regulatory mechanisms in a specific biological context. The smallest entities on the level of TFBSs combinations are called promoter modules. These are defined as two or more individual elements that act coordinately and are similarly arranged in the promoters of co-regulated transcripts.

**Figure 2:**
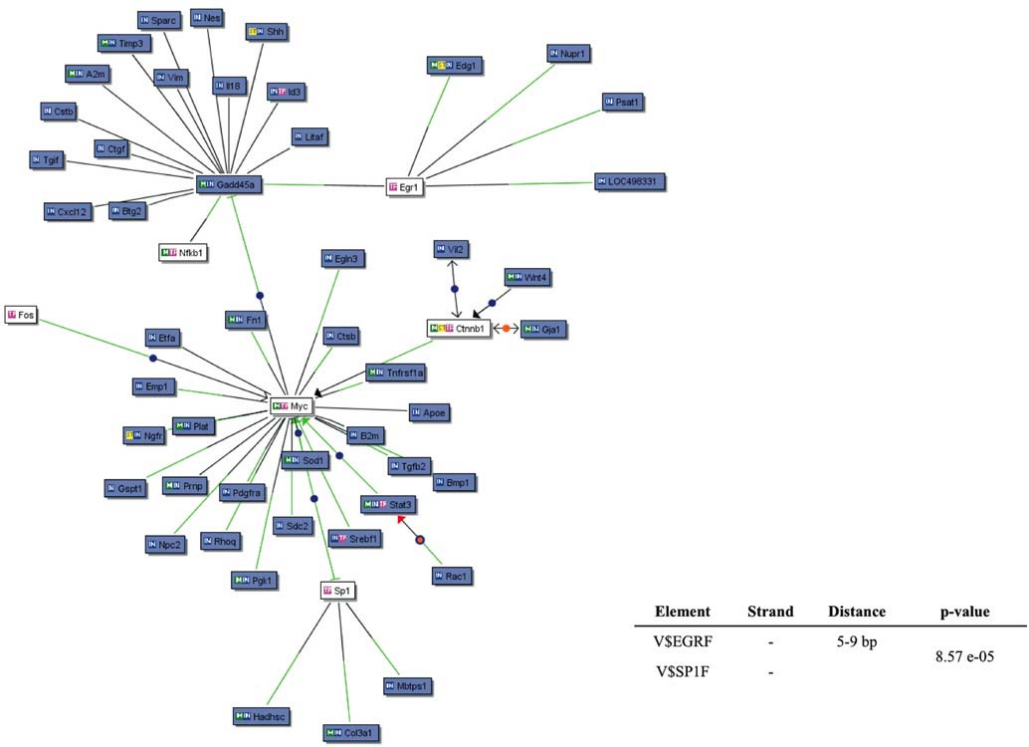
Knowledge-based gene network of the MesPC-specific genes. The nodes represent the genes. In BLUE, the MesPC-upregulated genes; in WHITE, the transcription factors not included in the list of significant genes that interact with the MesPC specific genes. The BLACK edges indicate co-citation of two genes in the PubMed database; the GREEN edges indicate the presence of a significant TFBS on the promoter of the given gene for the specific interacting transcription factor. Lower-right corner: summary of the regulatory model possibly regulating the expression of MesPC genes. The matrix elements present in the model (V$EGRF and V$SP1F), the DNA strand where they are present on the promoter regions, their relative distance, and the p-value are shown.

We investigated the interactions of the MesPC genes based on the PubMed co-citation and the presence of TFBS in their promoter sequences. From this analysis, a number of genes emerged to have a specific consensus binding sequence for EGR1 transcription factor in their promoters (PCR validation of the microarray results shown in Figure S1). By multiple alignments, we found a conserved module constituted by a binding site for EGR1 and a second site for SP1 (Figure 2). Next, we searched for this module in the whole set of known promoter sequences in *Rattus norvegicus*, locating it in the promoters of 659 genes. Geneontology classification showed significant over-representation of such families as neuron differentiation (34 genes), neurogenesis (37 genes), and neuron development (26 genes), suggesting a central role of EGR1-SP1 module during production and differentiation of neurons (Table S3).

### Genes upregulated in mesencephalon at E11

**Figure 3:**
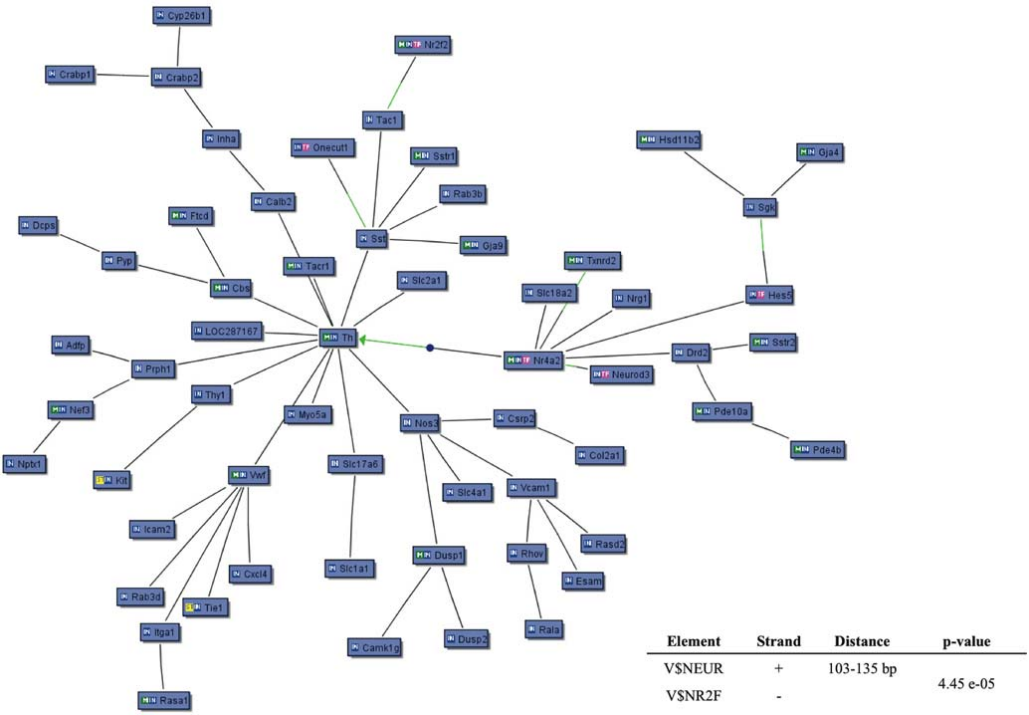
Knowledge-based gene network of the MesE11-specific genes. The nodes represent the genes. In BLUE, the MesE11-upregulated genes. The BLACK edges indicate co-citation of two genes in the PubMed database; the GREEN edges indicate the presence of a significant TFBS on the promoter of the given gene for the specific interacting transcription factor. Lower-right corner: summary of the regulatory model possibly regulating the expression of MesE11 genes. The matrix elements present in the model (V$NEUR and V$NR2F), the DNA strand where they are present on the promoter regions, their relative distance, and the p-value are shown.

We found 157 genes to be significantly over-expressed in MesE11 as compared to MesPC. These genes covered a variety of cellular and molecular functions, such as developmental process (43 genes), synaptic transmission (17 genes), nervous system development (16 genes) and neurogenesis (9 genes), and ion channels (9 genes). Interestingly, genes involved in oxygen transport (5 genes) and iron binding (11 genes) were also in this group (Table S4). As for the MesPC genes, we have inferred MesE11 gene network based on the PubMed co-citation and the presence of TFBS in their promoter sequences. This revealed a possible role for members of transcription factors of the NEUR and NR2F families in regulating the expression of many genes upregulated in MesE11. Particularly, the genes encoding respectively Neurod3 (NEUR family) and Nr2f2 (NR2F family) were significantly over-expressed in MesE11 (Figure 3). We also scanned the whole set of known *Rattus norvegicus* regulatory sequences, finding 726 promoters with the NEUR-NR2F module. Genes of the dopamine metabolism (Cyp2d22, Tgfb2, Nr4a2, Sncaip_predicted, Th, PCR validation of the microarray results for Nr4A2 and Th shown in Figure S1), synaptic transmission (30 genes) and development (24 genes) emerged as possible targets of NEUR-NR2F (Table S5).

### Dopamine-related genes

The Genomatix software Bibliosphere allows to search for genes that are co-cited in the PubMed abstracts with biological themes. By this approach, we have created a catalog of 1339 *Rattus Norvegicus* genes related in literature to dopamine. Of these, 1032 were present on the re-annotated Affymetrix chipset 230A. A total of 84 genes were found differentially expressed in all the three datasets (Table 2), when comparing MesPC (46 genes) and MesE11 (38 genes). Interestingly, 18 dopamine-related genes over-expressed in MesPC are described as involved in cell differentiation; a subgroup of this, composed of 8 genes, is involved in neuron differentiation. Five dopamine-related MesPC genes are also associated with neurodegenerative diseases. The dopamine-related genes over-expressed in MesE11 better resampled neurophysiologic events, as they were found associated to synaptic transmission (9 genes), transmission of nerve impulse (9 genes), and behavior (7 genes). Further, we have tested the hypothesis that the transcriptional models inferred from the MesPC and MesE11 gene networks would have a potential role in the regulation of some dopamine-related genes inferred from the literature scan. This seems to be the case, as 283 (∼21%) dopamine-related genes presented the EGFR-SP1F module in their promoter regions. This group was enriched in genes involved in neuron differentiation. Similarly, the promoters of 132 (∼10%) dopamine-related genes showed the ability to bind the NEUR-NR2F module. This group was enriched in genes involved in neurophysiologic processes, such as transmission of nerve impulse and synaptic transmission.

**Table 2:** Dopamine-related genes found significant comparing MesPC versus MesE11.

EGID	ENSID	RSID	GENESYMBOL	AvgLogFC	AvgAdjPvalue	Relative Over-expression
24772	ENSRNOG00000013589	NM_022177	CXCL12	3.10	3.186E-07	MesPC
24153	ENSRNOG00000028896	NM_012488	A2M	2.30	6.543E-05	MesPC
25692	ENSRNOG00000019018	NM_013151	PLAT	2.13	2.730E-07	MesPC
24596	ENSRNOG00000005392	NM_012610	NGFR	2.06	6.497E-07	MesPC
171163	ENSRNOG00000012876	NM_133623	SLC6A13	2.05	9.467E-07	MesPC
25625	ENSRNOG00000031312	NM_013091	TNFRSF1A	1.94	5.763E-05	MesPC
29539	ENSRNOG00000001344	NM_032416	ALDH2	1.82	1.480E-07	MesPC
64513	ENSRNOG00000005917	NM_033485	PAWR	1.77	5.590E-05	MesPC
83619	ENSRNOG00000001548	NM_031789	NFE2L2	1.77	1.405E-04	MesPC
54702	ENSRNOG00000005053	NM_019371	EGLN3	1.74	2.600E-06	MesPC
117549	ENSRNOG00000002524	NM_057201	GPR37	1.65	5.267E-06	MesPC
24212	ENSRNOG00000007290	NM_012505	ATP1A2	1.64	3.262E-04	MesPC
24423	ENSRNOG00000029726	NM_017014	GSTM1	1.58	3.070E-06	MesPC
315714	ENSRNOG00000008680	NM_001012125	LOXL1	1.52	2.213E-06	MesPC
316742	ENSRNOG00000015906	NM_001015020	TGIF	1.50	4.307E-05	MesPC
29318	ENSRNOG00000001239	NM_024131	DDT	1.46	9.887E-06	MesPC
25728	ENSRNOG00000018454	NM_138828	APOE	1.45	3.105E-04	MesPC
24223	ENSRNOG00000017123	NM_012512	B2M	1.40	2.920E-06	MesPC
295217	ENSRNOG00000013356	NM_001025648	SNAPAP	1.18	1.663E-06	MesPC
85272	ENSRNOG00000007108	XM_342591	BMP7	1.16	1.742E-04	MesPC
81818	ENSRNOG00000018087	NM_031140	VIM	1.15	4.160E-06	MesPC
81632	ENSRNOG00000002636	NM_031003	ABAT	1.10	4.378E-04	MesPC
25112	ENSRNOG00000005615	NM_024127	GADD45A	1.09	9.963E-06	MesPC
25104	ENSRNOG00000019372	NM_012744	PC	1.02	1.747E-04	MesPC
25227	ENSRNOG00000011150	NM_033443	ARSB	1.01	3.541E-04	MesPC
24392	ENSRNOG00000000805	NM_012567	GJA1	1.00	2.673E-04	MesPC
25177	ENSRNOG00000000875	NM_001033926	FHL1	0.93	3.247E-05	MesPC
25491	ENSRNOG00000018681	NM_012987	NES	0.90	4.750E-04	MesPC
83584	ENSRNOG00000009508	NM_031775	CASP6	0.90	2.798E-04	MesPC
24686	ENSRNOG00000021259	NM_012631	PRNP	0.90	1.443E-05	MesPC
25508	ENSRNOG00000033280	NM_013000	PAM	0.84	4.407E-06	MesPC
363875	ENSRNOG00000001068	NM_134366	RAC1	0.83	6.853E-05	MesPC
24530	ENSRNOG00000019573	NM_017024	LCAT	0.81	3.600E-05	MesPC
50719	ENSRNOG00000018824	NM_017353	SLC7A5	0.79	1.085E-04	MesPC
24788	ENSRNOG00000017291	NM_017052	SORD	0.79	9.679E-05	MesPC
79212	ENSRNOG00000006527	NM_024371	SLC6A1	0.79	8.860E-06	MesPC
84485	ENSRNOG00000019691	NM_053425	CCS	0.78	2.463E-04	MesPC
286898	ENSRNOG00000012062	NM_173118	NPC2	0.72	7.973E-06	MesPC
25125	ENSRNOG00000019742	NM_012747	STAT3	0.71	6.022E-04	MesPC
25368	ENSRNOG00000012325	NM_012895	ADK	0.62	2.013E-04	MesPC
171135	ENSRNOG00000014475	NM_133600	SLC31A1	0.62	3.827E-04	MesPC
299858	ENSRNOG00000025053	XM_243524	LRP1	0.57	2.421E-04	MesPC
83799	ENSRNOG00000012640	NM_031973	DPP7	0.57	3.506E-04	MesPC
29499	ENSRNOG00000006120	NM_017221	SHH	0.47	1.974E-04	MesPC
24786	ENSRNOG00000002115	NM_017050	SOD1	0.44	8.478E-04	MesPC
24644	ENSRNOG00000002467	NM_053291	PGK1	0.39	6.110E-04	MesPC
112400	ENSRNOG00000010392	NM_031588	NRG1	−0.33	7.169E-04	MesE11
24807	ENSRNOG00000005853	NM_012667	TACR1	−0.35	3.776E-04	MesE11
24600	ENSRNOG00000009348	NM_021838	NOS3	−0.43	2.880E-04	MesE11
114856	ENSRNOG00000003977	NM_053769	DUSP1	−0.43	3.614E-04	MesE11
171099	ENSRNOG00000014761	NM_133568	RASD2	−0.44	5.449E-04	MesE11
192215	ENSRNOG00000028844	NM_138858	SLC9A5	−0.44	5.824E-04	MesE11
29410	ENSRNOG00000022405	NM_019207	NEUROD3	−0.48	1.579E-04	MesE11
113912	ENSRNOG00000030920	NM_053613	RTN4R	−0.51	1.507E-04	MesE11
29427	ENSRNOG00000000853	NM_017196	AIF1	−0.51	4.323E-05	MesE11
54305	ENSRNOG00000002793	NM_019348	SSTR2	−0.53	4.593E-04	MesE11
25033	ENSRNOG00000022714	NM_012719	SSTR1	−0.69	5.147E-05	MesE11
116669	ENSRNOG00000019689	XM_342759	VWF	−0.73	2.187E-05	MesE11
24832	ENSRNOG00000006604	NM_012673	THY1	−0.75	3.745E-04	MesE11
117059	ENSRNOG00000016977	NM_053988	CALB2	−0.76	3.637E-05	MesE11
54705	ENSRNOG00000016180	NM_019372	PPM2C	−0.79	8.380E-04	MesE11
300519	ENSRNOG00000033217	NM_001004245	ESAM	−0.79	2.047E-04	MesE11
25017	ENSRNOG00000022968	NM_022178	MYO5A	−0.80	1.551E-04	MesE11
24318	ENSRNOG00000008428	NM_012547	DRD2	−0.81	2.270E-05	MesE11
81718	ENSRNOG00000000158	NM_052809	CDO1	−0.90	5.650E-05	MesE11
24626	ENSRNOG00000005905	NM_017031	PDE4B	−0.92	5.956E-04	MesE11
25256	ENSRNOG00000034191	NM_012792	FMO1	−0.92	1.957E-05	MesE11
24778	ENSRNOG00000007284	NM_138827	SLC2A1	−0.94	2.427E-05	MesE11
29700	ENSRNOG00000000566	NM_001007601	PCBD1	−1.02	4.220E-06	MesE11
360918	ENSRNOG00000028015	NM_001007729	CXCL4	−1.05	5.917E-06	MesE11
25550	ENSRNOG00000014816	NM_013032	SLC1A1	−1.07	5.360E-04	MesE11
171128	ENSRNOG00000012290	NM_133595	GCHFR	−1.13	7.110E-05	MesE11
54294	ENSRNOG00000002730	NM_019341	RGS5	−1.17	2.654E-04	MesE11
63885	ENSRNOG00000011310	NM_022236	PDE10A	−1.24	4.630E-04	MesE11
25549	ENSRNOG00000008890	NM_013031	SLC18A2	−1.29	8.150E-06	MesE11
25361	ENSRNOG00000014333	NM_012889	VCAM1	−1.33	4.330E-06	MesE11
54278	ENSRNOG00000005600	NM_019328	NR4A2	−1.38	3.559E-04	MesE11
24688	ENSRNOG00000015643	NM_012633	PRPH1	−1.51	1.306E-05	MesE11
24806	ENSRNOG00000007374	NM_012666	TAC1	−2.02	1.387E-06	MesE11
24588	ENSRNOG00000013916	NM_017029	NEF3	−2.11	2.025E-04	MesE11
84487	ENSRNOG00000016147	NM_053427	SLC17A6	−2.12	3.212E-04	MesE11
25085	ENSRNOG00000020410	NM_012740	TH	−3.01	2.853E-07	MesE11
24797	ENSRNOG00000001837	NM_012659	SST	−3.12	3.522E-07	MesE11
24440	ENSRNOG00000033465	NM_033234	HBB	−6.41	7.133E-10	MesE11

## Discussion

Model organisms are widely used in biomedical research elucidating mechanisms which would be impossible to experiment on using human samples. Mice and rats are often regarded as optimal choices for working on the mammalian CNS. However, *Rattus norvegicus* genome has been annotated much less in detail, as compared to the *Mus musculus* genome. In this paper we utilized up-to-date methods for annotating rat arrays with the best possible accuracy. We have used the transcriptional profiling for comparing tissue samples from rat brain to primary cells from the same origin. We studied the effect of re-annotation and at the same time elucidated how well primary cells resemble the original tissues in the level of transcriptional profile. We have extensively investigated the gene expression in rodents' mesencephalon at E11.5 and neuronal primary cultures after 9 DIV, derived from it. For this, we have carried out a microarray experiment using the Affymetrix GeneChips RAE230A for the *Rattus norvegicus* genome. Because of the design inaccuracies and of the fast speed of updating information concerning the genes and transcripts sequences, many Affymetrix probes are known to have severe design problems as such. Particularly for the chipset RAE230A, several probe sets contain probes with multiple genome hits (13.2%), with no known target (3.6%), with allele-specific probes (19.5%) [28]. Currently, several re-annotation methods are available allowing the probes to be mapped to genes, transcripts, or even exons sequences stored in public databases. However, exon-based re-annotation leads to decreased precision and increased variance in estimating gene expression, probably due to the smaller number of probes that map to each exon [29]. Moreover, due to the fact that the majority of the probes are designed ignoring splicing variances, we find more convenient to work with gene-based rather than transcript-based re-annotations. During the re-annotation process, each single oligonucleotide probe is re-assigned to the correct gene. However, some probes are eliminated, because they don't reliably recognize any transcript, they have been designed for matching the antisense sequence of a given transcript, or they are designed in allele-specific regions. For instance, only the 53.6% (EG), 60.5% (RF), and 44% (ES) of all the RAE230A probes can be utilized for re-annotation. Long lists of differentially expressed candidate genes are usually produced from microarray analysis. However, they cannot be considered as the end point of the analysis but rather as the starting point of a more meaningful interpretation, by taking advantage of the increasing knowledge about the functions of the genes within the cells. The annotation of the genes or transcripts is usually obtained from public libraries such as Gene Ontology [30] or KEGG [31]. Similarly, one can test whether the expression of genes located in specific portions of chromatin (i.e. cytobands or entire chromosome) are involved in certain experimental conditions. For any of the annotations used for grouping the genes, the terms are defined *a priori* and constructed independently from the experimental data. The DAVID database is one of the most reliable tools for annotating genes and transcripts, as well as for finding over-represented functional groups of genes in a given gene list. Interestingly, as many as 97% of all the significant EG entities were mapped into DAVID, while only 66.2% and 65.7% respectively from the RS and ENS presented a reliable DAVID annotation. Therefore, we conclude that the re-annotation of the Affymetrix probes according to the Entrez Gene database is the best in terms of gene annotation and functional analysis. Similar results were observed also during the re-annotation of the Affymetrix probes for human genes [32], [33]. Investigating gene expression by microarrays presents some limitations especially related to the fact that microarrays can estimate only the levels of the transcripts within the cells, not giving any information concerning the post-transcriptional regulations. In addition, only rigorous statistical methods allow keeping the false discovery rates at reasonable levels, as many technical sources of variations can affect the measurements. Other restrictions to be considered when working with large-scale gene expression studies consist in the inaccuracy of the functional annotation of some genes: for instance, many genes are annotated in the ontology “apoptosis”, without being directly correlated to this specific process, but simply being involved more generally in cell homeostasis. Careful inspection is always needed for a correct interpretation of long gene lists resulting from microarray assays. Finally, microarrays detect transcripts at very low concentrations, but when working with complex tissues, such as the brain, assigning the expression patterns to a certain cellular subpopulation is impossible. Nevertheless, we believe that the tissues should always be intended as functional entities and their global gene expression should be target of interest.

When examining whole tissues, gene expression from a variety of cell types, including non-neuronal tissues (especially blood), is recorded. This explains why we observed gene groups of haemoglobins and oxygen transport as significantly over-represented in MesE11 over MesPC. Overall, our results suggest that the MesPC are a reliable tool to be used in developmental neurobiology, as their gene expression programs largely resemble the tissue of origin. As these cell cultures comprise a mixed cell population, they are used by the scientific community because they mirror the midbrain neuronal composition better than more homogeneous cell lines [19], [23]. Thus we believe that it is important to know what the gene expression profiles are in these cultures. We found genes of the extracellular matrix and of the focal adhesions to be upregulated in MesPC. In the mesencephalon, the adhesion structures are synthesized and maintained by glial cells, that are absent in primary cultures. The protocol used to establish midbrain neuronal cultures enhances dopaminergic differentiation, thus leading to enrichment in positive neurons, when compared to standard cultures [34]. However, some genes of the dopamine biosynthesis were found over-expressed in MesE11. This finding is consistent with the decrease of TH mRNA observed during the *in vitro* culture progression [19], [23], [34], and can be due to the decrease of trans-synaptic stimulation following the dissociation of the tissue.

We identified Egr1 and Sp1 as key elements in the regulation of the transcription patterns in MesPC. Particularly, early growth response genes encode for transcription factors that regulate gene expression in response to a variety of stimuli influencing cell growth and differentiation, as well as response to injury and reaction to chronic nervous system diseases [35]. Following depolarization, transcription of the Egr1 gene increases in the MesPC [36], suggesting that this immediate early transcription factor might be a key gene mediating the electrical activity leading to neuronal differentiation. Mice lacking EGR genes present a wide range of developmental abnormalities, including infertility [37], defects of the hindbrain morphogenesis [38], defective myelination in the peripheral nervous system [39], and defects in learning and memory [40], [41]. Gene network and promoter alignment techniques suggested that several key transcripts found in MesE11 can be regulated by the binding of transcription factors of the NEUR and NR2F families. Moreover, the levels of Neurod3 (NEUR) and Nr2f2 (NR2F) were found over-expressed in MesE11. Neurogenic basic helix-loop-helix (bHLH) factors Mash1, neurogenins (Ngns) and NeuroD3 (also known as Ngn1) play important roles in Nurr1-induced mDA neuronal differentiation [42]. While the role of Ngn2 in the development of mDA neurons is well known [43], [44], less information is available concerning Neurod3. In addition to inducing neurogenesis by functioning as a transcriptional activator, Neurod3 seems to inhibit the differentiation of neural stem cells into astrocytes [45]. COUP-TFII (Nr2f2) seems to be involved in tangential GABAergic interneurons migration in the developing brain, through the regulation of short- and long-range guidance cues [46]. Functions of Nr2f2 in the mDA phenotype definition are still to be described. In summary, we present a broad view of the transcriptome of the DA neurons in primary cultures and in Mesencephalon E11. By employing gene network techniques, we propose novel models that could explain the transcription regulatory events taking place in the maintenance of the transcriptional identity of the mesencephalon, and in primary cultures derived from this CNS area, with a crucial role in physiology and pathology.

## Materials and Methods

### Animals and Dissections

Timed pregnant Sprague-Dawley rats (Charles River Breeding Laboratories, Milan, Italy) were sacrificed in accordance with the Society for Neuroscience guidelines and Italian law. Embryonic (E) age was determined by considering the day of insemination (as confirmed by vaginal plug) as day E0. Prenatal brains were quickly removed and placed in phosphate buffered saline (PBS) without calcium and magnesium and supplemented with 33 mM glucose. The ventral midbrain was carefully dissected under a stereoscope in sterile conditions and processed for cell cultures (MesPC) or RNA isolation (MesE11). The use of animals was approved by the Institute of Genetics and Biophysics ethical committee and is in agreement with the European Community Directives. All efforts were made to minimize animal suffering and to reduce the number of animals used.

### Cell Cultures

Cells were dissociated from the embryonic rat ventral midbrain and cultured as described [19], [22], [23]. In brief, the tissues were dissected from E11.5 embryos and dissociated using mechanical trituration with a fire polished Pasteur pipette in culture medium (see below) and 0.01% pancreatic deoxyribonuclease (Sigma, Milan, Italy); cells were centrifuged 10 min at 500 g, suspended in Neural Basal Medium (NBM, Invitrogen, Milan, Italy), counted and plated in NBM at a density of 18.000/cm^2^ in dishes coated with 15 ug/ml of poly-D-Lysine (Sigma). Multiwell plates (Corning Costar, Milan, Italy) were used for all cultures. NBM was supplemented with B27 (Invitrogen, Milan, Italy), fibroblast growth factor 2 (FGF2, 20 ng/ml, Sigma), the N-terminal fragment of Sonic hedgehog protein (SHH, 50 ng/ml) and fibroblast growth factor 8 (FGF8, 10 ng/ml) for 6 days *in vitro*. SHH was purified as previously described [23]. Half of the medium was changed every three days. After six days the medium supplements were withdrawn with the exception of B27, and was added the ascorbic acid. Cultures were left for an additional three days.

### Microarray strategy and sample preparation RNA isolation

To minimize biological variability four pregnant rats were sacrificed and the E11 embryos were mixed obtaining three groups. Each group was treated separately. The ventral midbrains dissected from each group were pooled (MesE11) or dissociated and the cells were cultured in duplicate. After 9 days *in vitro* the cultures were collected (MesPC). Three microarrays were hybridized with MesE11 and three with MesPC independent samples.

### RNA isolation and RT-PCR

RNA obtained from tissues or from primary cultures was extracted using the Tri-Reagent isolation system (Sigma) according to the manufacturer's instructions. The RNA from the culture duplicates was pooled. The yield and integrity of RNA were determined by a spectrophotometer of A_260_ and agarose gel electrophoresis respectively. Total RNA was treated with a DNA free kit (Ambion Inc., Milan, Italy) to eliminate possible DNA contaminations. For microarray hybridation an additional clean-up of total RNA was performed using the RNeasy kit (Qiagen, Milan, Italy). RNA samples were further processed for microarray hybridization or for RT-PCR. RT-PCR analyses were as previously described [47], [48]. In brief, two ug of RNA were reverse transcribed using random hexanucleotides as primers (New England Biolabs Inc., Milan, Italy, 6 mM) and 200 U of moloney-murine leukemia virus reverse transcriptase (Ambion). 1/20 of the reverse transcribed cDNA was amplified in a 25 ul reaction mixture containing AmpliTaq Gold DNA polymerase buffer (Applied Biosystem, Milan, Italy), 0.2 mM dNTPs (Finnzymes OY, Espoo, Finland), 0.4 mM each primer, 1.25 U AmpliTaq Gold DNA polymerase (Applied Biosystem) and 1 mCi [^32^P]dCTP (3000 Ci/mmol, Amersham Biosciences, Milan, Italy). As previously described [48], different sets of primer pairs were used in the same reaction tube to co-amplify cDNA, together with primers for the hypoxanthine-phosphoribosyl-transferase (Hprt), a constantly expressed gene during CNS development, used as an internal standard [49]. After a first denaturing step at 95°C for 8 min, PCR amplification was performed for 28 cycles organized as follows: 95°C for 0.5 min; 56°C–58°C for 0.5 min; 72°C for 0.5 min and was followed by a final extension step (72°C for 5 min). The specificity of PCR primers was determined by performing BLAST searches against the databases. Non-reverse-transcribed RNA templates and mock controls were always run in PCR reactions and never gave amplification products. The [^32^P]-labeled amplified products were separated by electrophoresis in 1.5% agarose gel, dried and exposed to a PhosphorImager screen (Amersham). Quantitation was achieved by integrating the volume areas of each fragment obtained from scanning the screens with PhosphorImager apparatus (Amersham), equipped with ImageQuant software. The ratio between the yield of each amplified product and that of the co-amplified HPRT allowed a relative estimate of the mRNA levels [48]. Triplicate samples allowed statistical analysis.

### Probe preparation and microarray hybridization

Using the protocol supplied by the manufacturer (Affymetrix, Santa Clara, CA), double-stranded cDNA was synthesized from total RNA and was used to obtain biotin-labeled cRNA by an *in vitro* transcription reaction (ENZO Diagnostics, Farmingdale, NY). Biotin-labeled cRNA was fragmented and hybridized with Affymetrix RAE230A rat genome GeneChip microarrays, according to the manufacturer's protocol, after verifying the quality of the biotin-labeled cRNA on a Test Chip (Affymetrix).

### Affymetrix probes re-annotation

We used a sequence-based re-annotation of the Affymetrix probes on RAE-230A chipset [28]. The R packages used in this study can be retrieved at http://brainarray.mbni.med.umich.edu/Brainarray/Database/CustomCDF/CDF_download_v10.asp

### Data Quality Control

Extensive quality control of the data microarray raw data has been carried out using the methods implemented in the BioConductor packages affy [50] and affyQCReport [51]. All the microarrays showed excellent quality according to the standards, thus all of them were considered for further analysis (data not shown available upon request from the authors).

### Data preprocessing

The preprocessing was carried out with the methods implemented in R (http://www.R-project.org) and BioConductor (http://www.bioconductor.org). The CEL files were imported into R environment. After standard quality control (results not shown), the re-annotated data were preprocessed using the RMA algorithm [52]. The RMA allows robust estimation of inter-array variability by employing quantile normalization and by fitting a linear model for each probe set across all the arrays of the dataset.

### Feature Selection

The statistical algorithms implemented in the package Limma [53] were employed for selecting the differentially expressed genes. The genes having p-value<0.001 after Benjamini-Hockberg correction [54] were considered as significantly differentially expressed.

### Functional analysis

The DAVID gene annotation system was used in order to select over-represented biological terms. Default statistical parameters were employed [27].

### Gene network and promoter analysis

For each contrast analyzed, the genes up-regulated in each array group were separately imported into the software Genomatix Bibiosphere to build up gene networks based on their co-citation in the literature as well as the presence of TFBS for known transcription factors in their promoter regions (http://www.genomatix.de/products/BiblioSphere/). The transcription factors (TF) presenting extensive or interesting connectivity within the network were chosen. Consequently, the genes presenting a significant consensus for the selected TFs were further analyzed. The promoter sequences of the genes were retrieved using the software Genomatix Gene2Promoter (http://www.genomatix.de/online help/help eldorado/Gene2Promoter Intro.html) and analyzed with Genomatix FrameWorker (http://www.genomatix.de/online help/help gems/FrameWorker.html) to search for common models containing at least two TFBS. Finally, the significant models were screened for the whole set of known *Rattus norvegicus* promoters by using Genomatix ModelInspector (http://www.genomatix.de/online help/help fastm/modelinspector help.html).

### Statistical analysis

The analyses applied to the microarray data have been described above. For all other experiments, analysis of variance was carried out, followed by post hoc comparison (ANOVA, Scheffè F-test). Data were expressed as mean+/−SEM.

## Supporting Information

Figure S1 Summary of the PCR validations of the microarray results:The Nr4a2, Th, and Egr1 gene expression have been tested by PCR and statistically validated as described in materials and methods.
(0.04 MB TIF)

Table S1 Genes significant in the three analyses with a DAVID annotation. Fields: DAVIDID: DAVID unique ID; EG: Entrez Gene ID; logFC_EG: logarithmic fold change (MesPC-MesE11) based on the EG re-annotation; P.Value_EG: p-value based on the EG re-annotation; adj.P.Val_EG: adjusted (Benjamini-Hochberg) p-value based on the EG re-annotation; ENSID: Ensembl gene ID; logFC_ENS: logarithmic fold change (MesPC-MesE11) based on the ENS re-annotation; P.Value_ENS: p-value based on the ENS re-annotation; adj.P.Val_ENS: adjusted (Benjamini-Hochberg) p-value based on the ENS re-annotation; RSID: RefSeq ID; logFC_RS: logarithmic fold change (MesPC-MesE11) based on the RS re-annotation; P.Value_RS: p-value based on the RS re-annotation; adj.P.Val_RS: adjusted (Benjamini-Hochberg) p-value based on the RS re-annotation; GeneName: official gene name.
(0.15 MB XLS)

Table S2 Functional analysis of the genes significantly over-expressed in MesPC. Each stack represents a group of related functional terms. Each term within each stack is in a row of the table. The number of genes annotated, the percent of representation of the term, and the enrichment p-value are shown.
(0.29 MB XLS)

Table S3 Summary of the Genomatix ModelInspector analysis for V$EGR-V$SP1F. Rattus norvegicus promoter sequences showing the V$EGR-V$SP1F module. For each match, the sequence annotation, the position of the module, and the strand are indicated. Over-represented Geneontology terms are also shown starting from page 53.
(0.39 MB PDF)

Table S4 Functional analysis of the genes significantly over-expressed in MesE11. Each stack represents a group of related functional terms. Each term within each stack is in a row of the table. The number of genes annotated, the percent of representation of the term, and the enrichment p-value are shown.
(0.19 MB XLS)

Table S5 Summary of the Genomatix ModelInspector analysis for V$NEUR-V$NR2F. Rattus norvegicus promoter sequences showing the V$NEUR-V$NR2F module. For each match, the sequence annotation, the position of the module, and the strand are indicated. Over-represented Geneontology terms are also shown starting from page 57.
(0.35 MB PDF)
