# Correction: Comparison of Gene Expression Profile in Embryonic Mesencephalon and Neuronal Primary Cultures

**DOI:** 10.1371/annotation/bb336408-6ec5-480e-ae37-85e9cefe0454

**Published:** 2009-05-15

**Authors:** Dario Greco, Floriana Volpicelli, Antonio Di Lieto, Damiana Leo, Carla Perrone-Capano, Petri Auvinen, Umberto di Porzio

The accession number for the gene discussed in this article was omitted. The accession number GSE15920 is registered with the Gene Expression Omnibus (GEO)database and available at https://www.ncbi.nlm.nih.gov/geo/query/acc.cgi?acc=GSE15920.

