# Supplementary figures and images for: Comparison of Gene Expression Profile in Embryonic Mesencephalon and Neuronal Primary Cultures
Source: PLoS One. 2009 Mar 23;4(3):e4977. doi: 10.1371/journal.pone.0004977 (PMC2654915; doi:10.1371/journal.pone.0004977)

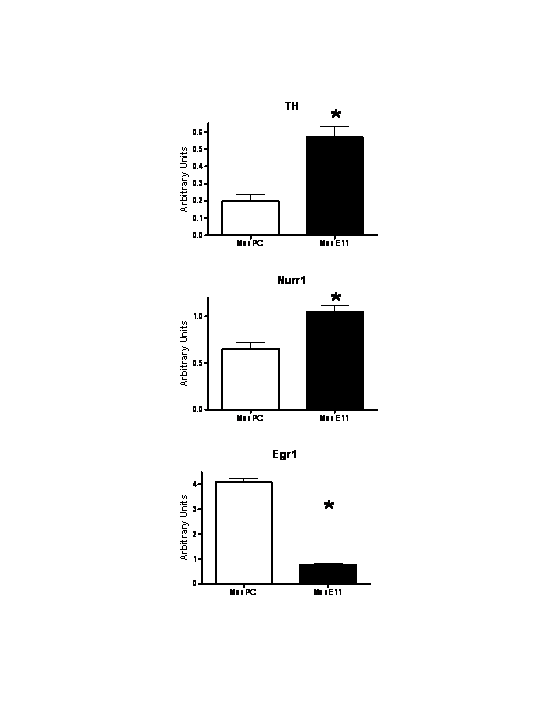

Supplement: Figure S1 — Summary of the PCR validations of the microarray results:The Nr4a2, Th, and Egr1 gene expression have been tested by PCR and statistically validated as described in materials and methods. (0.04 MB TIF) [file pone.0004977.s001.tif]
