# Supplementary material for: Comparison of Gene Expression Profile in Embryonic Mesencephalon and Neuronal Primary Cultures
Source: PLoS One. 2009 Mar 23;4(3):e4977. doi: 10.1371/journal.pone.0004977 (PMC2654915; doi:10.1371/journal.pone.0004977)
Supplement: Table S3 — Summary of the Genomatix ModelInspector analysis for V$EGR-V$SP1F. Rattus norvegicus promoter sequences showing the V$EGR-V$SP1F module. For each match, the sequence annotation, the position of the module, and the strand are indicated. Over-represented Geneontology terms are also shown starting from page 53. (0.39 MB PDF) [file pone.0004977.s004.pdf]

GEMS Launcher Task: *ModelInspector*: Search for user-defined models
working on Rat Promoters

Output overview of ModelInspector matches (1264 matches)

go to:[Output overview] [Detailed output] [Statistics]

ModelInspector Release 5.5 February 2008 **Tue May 27 16:48:09 2008**

Solution parameters:

**Sequence file:**	Rat Promoters
**Models:**	User-defined/EGRF-SP1F.model
**Strand(s) searched:**	both strands
**Threshold for number of elements:**	100.0 % (2 of 2 elements)
**Output sorted by:**	match positions on the sequences
**Maximum number of matches:**	1000

Match List:

Sequence	Model Name	Position	Strand	Select Match
**GXP_960** [GXP_960] (1 - 609)<br>**Hs3st2**, GXL_704, GeneID: 293451, Rattus norvegicus chr. 1<br>heparan sulfate (glucosamine) 3-O-sulfotransferase 2	EGRF-SP1F	537 - 557	(+)	
**GXP_991** [GXP_991] (1 - 1100)<br>**Fgf3**, GXL_732, GeneID: 170633, Rattus norvegicus chr. 1<br>fibroblast growth factor 3	EGRF-SP1F	831 - 851	(+)	
**GXP_995** [GXP_995] (1 - 680)<br>**P4ha3**, GXL_736, GeneID: 361612, Rattus norvegicus chr. 1	EGRF-SP1F<br>EGRF-SP1F	186 - 206<br>192 - 212	(+)<br>(+)	

procollagen-proline, 2-oxoglutarate 4-dioxygenase (proline 4-hydroxylase), alpha polypeptide III	EGRF-SP1F	523 - 501	(-)
	EGRF-SP1F	661 - 641	(-)
**GXP_1027** [GXP_1027] (1 - 881)<br>**Arrb1**, GXL_766, GeneID: 25387, Rattus norvegicus chr. 1<br>arrestin, beta 1	EGRF-SP1F	178 - 157	(-)
	EGRF-SP1F	691 - 670	(-)
	EGRF-SP1F	697 - 676	(-)
**GXP_1043** [GXP_1043] (1 - 792)<br>**Prcp_predicted**, GXL_782, GeneID: 293118, Rattus norvegicus chr. 1<br>prolylcarboxypeptidase (angiotensinase C) (predicted)	EGRF-SP1F	331 - 351	(+)
**GXP_1410284** [GXP_1410284] (1 - 601)<br>**Itgad**, GXL_793, GeneID: 64350, Rattus norvegicus chr. 1<br>integrin, alpha D	EGRF-SP1F	476 - 456	(-)
**GXP_1069** [GXP_1069] (1 - 760)<br>**Ap2a2**, GXL_807, GeneID: 81637, Rattus norvegicus chr. 1<br>adaptor protein complex AP-2, alpha 2 subunit	EGRF-SP1F	428 - 448	(+)
**GXP_1073** [GXP_1073] (1 - 1375)<br>**Bckdk**, GXL_811, GeneID: 29603, Rattus norvegicus chr. 1<br>branched chain ketoacid dehydrogenase kinase	EGRF-SP1F	569 - 547	(-)
**GXP_1082** [GXP_1082] (1 - 780)<br>**Cd81**, GXL_820, GeneID: 25621, Rattus norvegicus chr. 1<br>CD 81 antigen	EGRF-SP1F	230 - 206	(-)
**GXP_1093** [GXP_1093] (1 - 601)<br>**Picalm**, GXL_831, GeneID: 89816, Rattus norvegicus chr. 1<br>phosphatidylinositol binding clathrin assembly protein	EGRF-SP1F	146 - 126	(-)
**GXP_1129** [GXP_1129] (1 - 601)<br>**Olr20_predicted**, GXL_867, GeneID: 293083, Rattus norvegicus chr. 1<br>olfactory receptor 20 (predicted)	EGRF-SP1F	211 - 189	(-)
	EGRF-SP1F	217 - 195	(-)
	EGRF-SP1F	223 - 201	(-)
**GXP_1409786** [GXP_1409786] (1 - 601)<br>**Slc28a1**, GXL_869, GeneID: 116642, Rattus norvegicus chr. 1<br>solute carrier family 28 (sodium-coupled nucleoside transporter), member 1	EGRF-SP1F	367 - 389	(+)
**GXP_1150** [GXP_1150] (1 - 601)<br>**Olr154_predicted**, GXL_887, GeneID: 365333, Rattus norvegicus chr. 1<br>olfactory receptor 154 (predicted)	EGRF-SP1F	219 - 242	(+)
**GXP_1159002** [GXP_1159002] (1 - 650)<br>**Phkg2**, GXL_903, GeneID: 140671, Rattus norvegicus chr. 1<br>phosphorylase kinase, gamma 2 (testis)	EGRF-SP1F	283 - 303	(+)
**GXP_1410256** [GXP_1410256] (1 - 601)<br>**Phkg2**, GXL_903, GeneID: 140671, Rattus norvegicus chr. 1<br>phosphorylase kinase, gamma 2 (testis)	EGRF-SP1F	135 - 113	(-)
	EGRF-SP1F	39 - 19	(-)

**GXP_1179** [GXP_1179] (1 - 1136)<br>**Isg20**, GXL_915, GeneID: 293052, Rattus norvegicus chr. 1<br>interferon stimulated exonuclease 20	EGRF-SP1F	567 - 587	(+)
	EGRF-SP1F	953 - 976	(+)
**GXP_1409737** [GXP_1409737] (1 - 602)<br>**Abhd2_predicted**, GXL_917, GeneID: 293050, Rattus norvegicus chr. 1<br>abhydrolase domain containing 2 (predicted)	EGRF-SP1F	240 - 216	(-)
**GXP_1196** [GXP_1196] (1 - 1110)<br>**Pde3b**, GXL_932, GeneID: 29516, Rattus norvegicus chr. 1<br>phosphodiesterase 3B	EGRF-SP1F	469 - 448	(-)
	EGRF-SP1F	612 - 633	(+)
**GXP_1209** [GXP_1209] (1 - 1227)<br>**Ric8a**, GXL_945, GeneID: 293614, Rattus norvegicus chr. 1<br>resistance to inhibitors of cholinesterase 8 homolog A (C. elegans)	EGRF-SP1F	904 - 882	(-)
**GXP_1409694** [GXP_1409694] (1 - 621)<br>**Igf1r**, GXL_953, GeneID: 25718, Rattus norvegicus chr. 1<br>insulin-like growth factor 1 receptor	EGRF-SP1F	190 - 213	(+)
**GXP_1410370** [GXP_1410370] (1 - 647)<br>**Ppp2r2d**, GXL_957, GeneID: 246255, Rattus norvegicus chr. 1<br>protein phosphatase 2, regulatory subunit B, delta isoform	EGRF-SP1F	469 - 447	(-)
**GXP_1236** [GXP_1236] (1 - 844)<br>**Mesdc2**, GXL_972, GeneID: 308796, Rattus norvegicus chr. 1<br>mesoderm development candiate 2	EGRF-SP1F	679 - 656	(-)
**GXP_1244** [GXP_1244] (1 - 729)<br>**Pde2a**, GXL_980, GeneID: 81743, Rattus norvegicus chr. 1<br>phosphodiesterase 2A, cGMP-stimulated	EGRF-SP1F	477 - 454	(-)
	EGRF-SP1F	483 - 459	(-)
	EGRF-SP1F	488 - 465	(-)
	EGRF-SP1F	580 - 558	(-)
**GXP_1249** [GXP_1249] (1 - 847)<br>**Pde8a**, GXL_985, GeneID: 308776, Rattus norvegicus chr. 1<br>phosphodiesterase 8A	EGRF-SP1F	577 - 597	(+)
	EGRF-SP1F	675 - 696	(+)
**GXP_1410231** [GXP_1410231] (1 - 931)<br>**Doc2a**, GXL_991, GeneID: 65031, Rattus norvegicus chr. 1<br>double C2, alpha	EGRF-SP1F	646 - 623	(-)
	EGRF-SP1F	855 - 878	(+)
	EGRF-SP1F	907 - 928	(+)
**GXP_1410232** [GXP_1410232] (1 - 601)<br>**Doc2a**, GXL_991, GeneID: 65031, Rattus norvegicus chr. 1<br>double C2, alpha	EGRF-SP1F	85 - 108	(+)
	EGRF-SP1F	137 - 158	(+)
**GXP_1258** [GXP_1258] (1 - 860)<br>**Agc1**, GXL_994, GeneID: 58968, Rattus norvegicus chr. 1<br>aggrecan 1	EGRF-SP1F	440 - 461	(+)
	EGRF-SP1F	445 - 467	(+)
	EGRF-SP1F	449 - 471	(+)
	EGRF-SP1F	732 - 756	(+)

**GXP_1266** [GXP_1266] (1 - 684)<br>**Rnf40**, GXL_1002, GeneID: 266712, Rattus norvegicus chr. 1<br>ring finger protein 40	EGRF-SP1F	450 - 430	(-)
**GXP_1410257** [GXP_1410257] (1 - 602)<br>**Rnf40**, GXL_1002, GeneID: 266712, Rattus norvegicus chr. 1<br>ring finger protein 40	EGRF-SP1F	162 - 142	(-)
**GXP_1281** [GXP_1281] (1 - 675)<br>**Lcmt1**, GXL_1017, GeneID: 361643, Rattus norvegicus chr. 1<br>leucine carboxyl methyltransferase 1	EGRF-SP1F	530 - 507	(-)
**GXP_1287** [GXP_1287] (1 - 1290)<br>**Tbx6_predicted**, GXL_1023, GeneID: 365371, Rattus norvegicus chr. 1<br>T-box 6 (predicted)	EGRF-SP1F	375 - 353	(-)
**GXP_1410223** [GXP_1410223] (1 - 601)<br>**Tbx6_predicted**, GXL_1023, GeneID: 365371, Rattus norvegicus chr. 1<br>T-box 6 (predicted)	EGRF-SP1F	25 - 49	(+)
**GXP_1295** [GXP_1295] (1 - 618)<br>**Prap1**, GXL_1031, GeneID: 60574, Rattus norvegicus chr. 1<br>proline-rich acidic protein 1	EGRF-SP1F	407 - 387	(-)
**GXP_1409874** [GXP_1409874] (1 - 679)<br>**Pak1**, GXL_1044, GeneID: 29431, Rattus norvegicus chr. 1<br>p21 (CDKN1A)-activated kinase 1	EGRF-SP1F	467 - 487	(+)
	EGRF-SP1F	471 - 492	(+)
**GXP_1318** [GXP_1318] (1 - 701)<br>**Gab2**, GXL_1053, GeneID: 84477, Rattus norvegicus chr. 1<br>growth factor receptor bound protein 2-associated protein 2	EGRF-SP1F	70 - 47	(-)
	EGRF-SP1F	488 - 509	(+)
**GXP_1350** [GXP_1350] (1 - 601)<br>**Olr41_predicted**, GXL_1085, GeneID: 293195, Rattus norvegicus chr. 1<br>olfactory receptor 41 (predicted)	EGRF-SP1F	127 - 147	(+)
**GXP_1354** [GXP_1354] (1 - 601)<br>**Bag3**, GXL_1089, GeneID: 293524, Rattus norvegicus chr. 1<br>Bcl2-associated athanogene 3	EGRF-SP1F	268 - 292	(+)
**GXP_1409960** [GXP_1409960] (1 - 602)<br>**Trpc2**, GXL_1119, GeneID: 64573, Rattus norvegicus chr. 1<br>transient receptor potential cation channel, subfamily C, member 2	EGRF-SP1F	337 - 357	(+)
	EGRF-SP1F	340 - 361	(+)
**GXP_1385** [GXP_1385] (1 - 601)<br>**Mgmt**, GXL_1120, GeneID: 25332, Rattus norvegicus chr. 1<br>O-6-methylguanine-DNA methyltransferase	EGRF-SP1F	306 - 326	(+)
**GXP_1393** [GXP_1393] (1 - 700)<br>**Prc1_predicted**, GXL_1128, GeneID: 308761, Rattus norvegicus chr. 1<br>protein regulator of cytokinesis 1 (predicted)	EGRF-SP1F	440 - 417	(-)
	EGRF-SP1F	467 - 446	(-)

**GXP_1419** [GXP_1419] (1 - 601)			
**Tm6sf1_predicted**, GXL_1154, GeneID: 361600, Rattus norvegicus chr. 1			
transmembrane 6 superfamily member 1 (predicted)			
**GXP_1425** [GXP_1425] (1 - 742)	EGRF-SP1F	295 - 318	(+)
**Fgf15**, GXL_1160, GeneID: 170582, Rattus norvegicus chr. 1			
fibroblast growth factor 15	EGRF-SP1F	413 - 389	(-)
	EGRF-SP1F	275 - 251	(-)
**GXP_1446** [GXP_1446] (1 - 634)			
**Ncoa2**, GXL_1181, GeneID: 83724, Rattus norvegicus chr. 5	EGRF-SP1F	277 - 256	(-)
nuclear receptor coactivator 2	EGRF-SP1F	279 - 258	(-)
	EGRF-SP1F	287 - 264	(-)
**GXP_1453** [GXP_1453] (1 - 601)	EGRF-SP1F	424 - 403	(-)
**C1qg**, GXL_1188, GeneID: 362634, Rattus norvegicus chr. 5			
complement component 1, q subcomponent, gamma polypeptide	EGRF-SP1F	565 - 589	(+)
**GXP_1416679** [GXP_1416679] (1 - 607)			
**Clcnkb**, GXL_1198, GeneID: 79430, Rattus norvegicus chr. 5	EGRF-SP1F	416 - 395	(-)
chloride channel Kb			
**GXP_1504** [GXP_1504] (1 - 669)	EGRF-SP1F	360 - 384	(+)
**Zfp483**, GXL_1237, GeneID: 170955, Rattus norvegicus chr. 5			
zinc finger protein 483	EGRF-SP1F	366 - 388	(+)
**GXP_1509** [GXP_1509] (1 - 601)	EGRF-SP1F	116 - 137	(+)
**Spink4**, GXL_1242, GeneID: 408233, Rattus norvegicus chr. 5			
Kazal type serine protease inhibitor 4	EGRF-SP1F	576 - 556	(-)
**GXP_1527** [GXP_1527] (1 - 864)	EGRF-SP1F	498 - 519	(+)
**Cdw92**, GXL_1260, GeneID: 85254, Rattus norvegicus chr. 5			
CDW92 antigen	EGRF-SP1F	501 - 523	(+)
**GXP_1545** [GXP_1545] (1 - 601)			
**Ccin**, GXL_1276, GeneID: 298392, Rattus norvegicus chr. 5	EGRF-SP1F	395 - 374	(-)
calicin			
**GXP_1547** [GXP_1547] (1 - 601)			
**Rab2**, GXL_1278, GeneID: 65158, Rattus norvegicus chr. 5	EGRF-SP1F	291 - 268	(-)
RAB2, member RAS oncogene family			
**GXP_1415645** [GXP_1415645] (1 - 601)			
**Rab2**, GXL_1278, GeneID: 65158, Rattus norvegicus chr. 5	EGRF-SP1F	276 - 256	(-)
RAB2, member RAS oncogene family			
**GXP_1558** [GXP_1558] (1 - 982)	EGRF-SP1F	312 - 333	(+)
**Faah**, GXL_1289, GeneID: 29347, Rattus norvegicus chr. 5	EGRF-SP1F	317 - 338	(+)
fatty acid amide hydrolase	EGRF-SP1F	321 - 342	(+)
**GXP_1570** [GXP_1570] (1 - 1176)	EGRF-SP1F	127 - 107	(-)
**Ttc22_predicted**, GXL_1300, GeneID: 298300, Rattus norvegicus chr. 5	EGRF-SP1F	129 - 109	(-)

tetratricopeptide repeat domain 22 (predicted)			
**GXP_1580** [GXP_1580] (1 - 1148)<br>**Wdr32_predicted**, GXL_1310, GeneID: 313242, Rattus norvegicus chr. 5<br>WD repeat domain 32 (predicted)	EGRF-SP1F	674 - 651	(-)
**GXP_1589** [GXP_1589] (1 - 651)<br>**Sdcbp**, GXL_1319, GeneID: 83841, Rattus norvegicus chr. 5<br>syndecan binding protein	EGRF-SP1F	438 - 418	(-)
**GXP_1415635** [GXP_1415635] (1 - 601)<br>**Sdcbp**, GXL_1319, GeneID: 83841, Rattus norvegicus chr. 5<br>syndecan binding protein	EGRF-SP1F	384 - 364	(-)
**GXP_1600** [GXP_1600] (1 - 728)<br>**Klhl17**, GXL_1329, GeneID: 246757, Rattus norvegicus chr. 5<br>kelch-like 17 (Drosophila)	EGRF-SP1F	699 - 720	(+)
**GXP_1610** [GXP_1610] (1 - 844)<br>**Faf1**, GXL_1339, GeneID: 140657, Rattus norvegicus chr. 5<br>Fas-associated factor 1	EGRF-SP1F	481 - 460	(-)
**GXP_1613** [GXP_1613] (1 - 632)<br>**Tmod1**, GXL_1342, GeneID: 25566, Rattus norvegicus chr. 5<br>tropomodulin 1	EGRF-SP1F	457 - 436	(-)
	EGRF-SP1F	475 - 453	(-)
**GXP_1614** [GXP_1614] (1 - 788)<br>**Clta**, GXL_1343, GeneID: 83800, Rattus norvegicus chr. 5<br>clathrin, light polypeptide (Lca)	EGRF-SP1F	638 - 615	(-)
**GXP_1624** [GXP_1624] (1 - 796)<br>**Kif1b**, GXL_1353, GeneID: 117548, Rattus norvegicus chr. 5<br>kinesin family member 1B	EGRF-SP1F	343 - 363	(+)
**GXP_517848** [GXP_517848] (1 - 726)<br>**Fbxo6b**, GXL_1362, GeneID: 192351, Rattus norvegicus chr. 5<br>F-box only protein 6b	EGRF-SP1F	499 - 477	(-)
**GXP_1167450** [GXP_1167450] (1 - 601)<br>**Pde4b**, GXL_1405, GeneID: 24626, Rattus norvegicus chr. 5<br>phosphodiesterase 4B, cAMP specific	EGRF-SP1F	171 - 191	(+)
	EGRF-SP1F	332 - 311	(-)
**GXP_1679** [GXP_1679] (1 - 647)<br>**Clcnk1**, GXL_1407, GeneID: 79425, Rattus norvegicus chr. 5<br>chloride channel K1	EGRF-SP1F	470 - 448	(-)
	EGRF-SP1F	475 - 454	(-)
**GXP_1682** [GXP_1682] (1 - 998)<br>**Nr4a3**, GXL_1409, GeneID: 58853, Rattus norvegicus chr. 5<br>nuclear receptor subfamily 4, group A, member 3	EGRF-SP1F	550 - 570	(+)
**GXP_1167102** [GXP_1167102] (1 - 601)<br>**Nr4a3**, GXL_1409, GeneID: 58853, Rattus norvegicus chr. 5<br>nuclear receptor subfamily 4, group A, member 3	EGRF-SP1F	428 - 452	(+)

**GXP_1688** [GXP_1688] (1 - 602)<br>**Anp32b**, GXL_1415, GeneID: 170724, Rattus norvegicus chr. 5<br>acidic nuclear phosphoprotein 32 family, member B	EGRF-SP1F	213 - 233	(+)
**GXP_1713** [GXP_1713] (1 - 680)<br>**Gabrd**, GXL_1439, GeneID: 29689, Rattus norvegicus chr. 5<br>gamma-aminobutyric acid A receptor, delta	EGRF-SP1F	454 - 432	(-)
**GXP_1416639** [GXP_1416639] (1 - 602)<br>**Pla2g5**, GXL_1442, GeneID: 29354, Rattus norvegicus chr. 5<br>phospholipase A2, group V	EGRF-SP1F	539 - 559	(+)
**GXP_1757** [GXP_1757] (1 - 630)<br>**Tesk1**, GXL_1483, GeneID: 29460, Rattus norvegicus chr. 5<br>testis specific protein kinase 1	EGRF-SP1F	602 - 582	(-)
**GXP_1415804** [GXP_1415804] (1 - 613)<br>**Tesk1**, GXL_1483, GeneID: 29460, Rattus norvegicus chr. 5<br>testis specific protein kinase 1	EGRF-SP1F	297 - 277	(-)
**GXP_1763** [GXP_1763] (1 - 614)<br>**Tnfrsf1b**, GXL_1489, GeneID: 156767, Rattus norvegicus chr. 5<br>tumor necrosis factor receptor superfamily, member 1b	EGRF-SP1F	440 - 464	(+)
**GXP_1775** [GXP_1775] (1 - 601)<br>**Pomgnt1**, GXL_1501, GeneID: 362567, Rattus norvegicus chr. 5<br>protein O-linked mannose beta1, 2-N-acetylglucosaminyltransferase	EGRF-SP1F	496 - 474	(-)
**GXP_1789** [GXP_1789] (1 - 671)<br>**Atp6v1g1_predicted**, GXL_1514, GeneID: 298103, Rattus norvegicus chr. 5<br>ATPase, H transporting, lysosomal V1 subunit G1 (predicted)	EGRF-SP1F	431 - 453	(+)
**GXP_1826** [GXP_1826] (1 - 662)<br>**Ubap1**, GXL_1551, GeneID: 362502, Rattus norvegicus chr. 5<br>ubiquitin-associated protein 1	EGRF-SP1F	496 - 475	(-)
**GXP_1416627** [GXP_1416627] (1 - 784)<br>**Pink1_predicted**, GXL_1553, GeneID: 298575, Rattus norvegicus chr. 5<br>PTEN induced putative kinase 1 (predicted)	EGRF-SP1F	264 - 242	(-)
**GXP_1829** [GXP_1829] (1 - 916)<br>**Msc_predicted**, GXL_1554, GeneID: 312897, Rattus norvegicus chr. 5<br>musculin (predicted)	EGRF-SP1F	466 - 489	(+)
**GXP_1416092** [GXP_1416092] (1 - 602)<br>**Nfia**, GXL_1576, GeneID: 25492, Rattus norvegicus chr. 5<br>nuclear factor I/A	EGRF-SP1F<br>EGRF-SP1F	191 - 171<br>193 - 173	(-)<br>(-)
**GXP_1416094** [GXP_1416094] (1 - 601)<br>**Nfia**, GXL_1576, GeneID: 25492, Rattus norvegicus chr. 5<br>nuclear factor I/A	EGRF-SP1F<br>EGRF-SP1F	331 - 352<br>232 - 252	(+)<br>(+)

GXP_1854 [GXP_1854] (1 - 618)
Eps15, GXL_1579, GeneID: 313474, Rattus norvegicus chr. 5
epidermal growth factor receptor pathway substrate 15

EGRF-SP1F	330 - 351	(+)
EGRF-SP1F	485 - 463	(-)
EGRF-SP1F	490 - 468	(-)

GXP_1867 [GXP_1867] (1 - 601)
Tmeff1, GXL_1591, GeneID: 63845, Rattus norvegicus chr. 5
transmembrane protein with EGF-like and two follistatin-like domains 1

EGRF-SP1F	428 - 449	(+)

GXP_1890 [GXP_1890] (1 - 674)
Alpl, GXL_1614, GeneID: 25586, Rattus norvegicus chr. 5
alkaline phosphatase, tissue-nonspecific

EGRF-SP1F	502 - 480	(-)

GXP_1168112 [GXP_1168112] (1 - 601)
Alpl, GXL_1614, GeneID: 25586, Rattus norvegicus chr. 5
alkaline phosphatase, tissue-nonspecific

EGRF-SP1F	362 - 340	(-)

GXP_1891 [GXP_1891] (1 - 1032)
Per3, GXL_1615, GeneID: 78962, Rattus norvegicus chr. 5
period homolog 3 (Drosophila)

EGRF-SP1F	319 - 341	(+)

GXP_1897 [GXP_1897] (1 - 683)
Ssbp3, GXL_1621, GeneID: 84354, Rattus norvegicus chr. 5
single stranded DNA binding protein 3

EGRF-SP1F	289 - 309	(+)
EGRF-SP1F	588 - 567	(-)
EGRF-SP1F	652 - 630	(-)

GXP_1416170 [GXP_1416170] (1 - 603)
Ssbp3, GXL_1621, GeneID: 84354, Rattus norvegicus chr. 5
single stranded DNA binding protein 3

EGRF-SP1F	161 - 138	(-)

GXP_1898 [GXP_1898] (1 - 1134)
Ugcg, GXL_1622, GeneID: 83626, Rattus norvegicus chr. 5
UDP-glucose ceramide glucosyltransferase

EGRF-SP1F	608 - 586	(-)

GXP_1905 [GXP_1905] (1 - 602)
Spsb1_predicted, GXL_1629, GeneID: 313722, Rattus norvegicus chr. 5
splA/ryanodine receptor domain and SOCS box containing 1 (predicted)

EGRF-SP1F	111 - 133	(+)
EGRF-SP1F	115 - 138	(+)

GXP_1416770 [GXP_1416770] (1 - 640)
Spsb1_predicted, GXL_1629, GeneID: 313722, Rattus norvegicus chr. 5
splA/ryanodine receptor domain and SOCS box containing 1 (predicted)

EGRF-SP1F	315 - 292	(-)
EGRF-SP1F	319 - 297	(-)
EGRF-SP1F	324 - 303	(-)
EGRF-SP1F	331 - 308	(-)

GXP_1913 [GXP_1913] (1 - 985)
Pdpn, GXL_1637, GeneID: 54320, Rattus norvegicus chr. 5
podoplanin

EGRF-SP1F	615 - 637	(+)
EGRF-SP1F	619 - 643	(+)

GXP_1917 [GXP_1917] (1 - 601)
Unc13b, GXL_1641, GeneID: 64830, Rattus norvegicus chr. 5
unc-13 homolog B (C. elegans)

EGRF-SP1F	405 - 428	(+)

GXP_1415859 [GXP_1415859] (1 - 602)
Tdrd7, GXL_1644, GeneID: 85425, Rattus norvegicus chr. 5

EGRF-SP1F	311 - 291	(-)

tudor domain containing 7	EGRF-SP1F	352 - 373	(+)
**GXP_1417545** [GXP_1417545] (1 - 604)<br>**Npuk68**, GXL_10174, GeneID: 192359, Rattus norvegicus chr. 6<br>nuclear protein UKp68	EGRF-SP1F	346 - 322	(-)
**GXP_12416** [GXP_12416] (1 - 706)<br>**Nrcam**, GXL_10179, GeneID: 497815, Rattus norvegicus chr. 6<br>neuron-glia-CAM-related cell adhesion molecule	EGRF-SP1F	410 - 386	(-)
	EGRF-SP1F	515 - 491	(-)
**GXP_12433** [GXP_12433] (1 - 601)<br>**Crip2**, GXL_10195, GeneID: 338401, Rattus norvegicus chr. 6<br>cysteine-rich protein 2	EGRF-SP1F	508 - 484	(-)
**GXP_12478** [GXP_12478] (1 - 1313)<br>**Rhoj**, GXL_10240, GeneID: 299145, Rattus norvegicus chr. 6<br>ras homolog gene family, member J	EGRF-SP1F	206 - 186	(-)
**GXP_12489** [GXP_12489] (1 - 850)<br>**Cgref1**, GXL_10251, GeneID: 245918, Rattus norvegicus chr. 6<br>cell growth regulator with EF hand domain 1	EGRF-SP1F	821 - 800	(-)
**GXP_1417032** [GXP_1417032] (1 - 601)<br>**Cgref1**, GXL_10251, GeneID: 245918, Rattus norvegicus chr. 6<br>cell growth regulator with EF hand domain 1	EGRF-SP1F	333 - 356	(+)
**GXP_12520** [GXP_12520] (1 - 609)<br>**Slc5a6**, GXL_10282, GeneID: 170551, Rattus norvegicus chr. 6<br>solute carrier family 5 (sodium-dependent vitamin transporter), member 6	EGRF-SP1F	250 - 228	(-)
	EGRF-SP1F	443 - 421	(-)
**GXP_1168670** [GXP_1168670] (1 - 904)<br>**Slc5a6**, GXL_10282, GeneID: 170551, Rattus norvegicus chr. 6<br>solute carrier family 5 (sodium-dependent vitamin transporter), member 6	EGRF-SP1F	789 - 767	(-)
**GXP_12531** [GXP_12531] (1 - 601)<br>**Dnmt3a**, GXL_10291, GeneID: 444984, Rattus norvegicus chr. 6<br>DNA methyltransferase 3A	EGRF-SP1F	449 - 469	(+)
	EGRF-SP1F	470 - 492	(+)
	EGRF-SP1F	488 - 509	(+)
	EGRF-SP1F	512 - 536	(+)
**GXP_12533** [GXP_12533] (1 - 601)<br>**Foxg1**, GXL_10293, GeneID: 24370, Rattus norvegicus chr. 6<br>forkhead box G1	EGRF-SP1F	296 - 273	(-)
	EGRF-SP1F	310 - 287	(-)
	EGRF-SP1F	315 - 292	(-)
	EGRF-SP1F	321 - 298	(-)
	EGRF-SP1F	327 - 303	(-)
**GXP_12580** [GXP_12580] (1 - 601)<br>**Smoc1**, GXL_10340, GeneID: 314280, Rattus norvegicus chr. 6<br>SPARC-related modular calcium binding protein 1	EGRF-SP1F	89 - 69	(-)
	EGRF-SP1F	186 - 207	(+)
**GXP_12584** [GXP_12584] (1 - 757)<br>**Rock2**, GXL_10344, GeneID: 25537, Rattus norvegicus chr. 6	EGRF-SP1F	296 - 273	(-)

Rho-associated coiled-coil forming kinase 2	EGRF-SP1F	640 - 617	(-)
**GXP_12617** [GXP_12617] (1 - 630)	EGRF-SP1F	45 - 24	(-)
**Hpcal1**, GXL_10377, GeneID: 50871, Rattus norvegicus chr. 6<br>hippocalcin-like 1	EGRF-SP1F	48 - 28	(-)
**GXP_1417113** [GXP_1417113] (1 - 601)			
**Hpcal1**, GXL_10377, GeneID: 50871, Rattus norvegicus chr. 6<br>hippocalcin-like 1	EGRF-SP1F	202 - 179	(-)
**GXP_1168703** [GXP_1168703] (1 - 784)			
**Dtnb**, GXL_10420, GeneID: 362715, Rattus norvegicus chr. 6<br>dystrobrevin, beta	EGRF-SP1F	490 - 510	(+)
**GXP_12666** [GXP_12666] (1 - 890)	EGRF-SP1F	275 - 297	(+)
**Ppm1a**, GXL_10425, GeneID: 24666, Rattus norvegicus chr. 6	EGRF-SP1F	565 - 542	(-)
protein phosphatase 1A, magnesium dependent, alpha isoform	EGRF-SP1F	760 - 781	(+)
**GXP_1169102** [GXP_1169102] (1 - 601)			
**Ppm1a**, GXL_10425, GeneID: 24666, Rattus norvegicus chr. 6<br>protein phosphatase 1A, magnesium dependent, alpha isoform	EGRF-SP1F	22 - 45	(+)
**GXP_12672** [GXP_12672] (1 - 926)			
**Glrx5_predicted**, GXL_10431, GeneID: 362776, Rattus norvegicus chr. 6<br>glutaredoxin 5 homolog (S. cerevisiae) (predicted)	EGRF-SP1F	889 - 866	(-)
**GXP_12675** [GXP_12675] (1 - 852)	EGRF-SP1F	272 - 296	(+)
**Prkch**, GXL_10434, GeneID: 81749, Rattus norvegicus chr. 6	EGRF-SP1F	510 - 486	(-)
protein kinase C, eta	EGRF-SP1F	694 - 674	(-)
**GXP_12681** [GXP_12681] (1 - 601)	EGRF-SP1F	229 - 209	(-)
**Yy1**, GXL_10440, GeneID: 24919, Rattus norvegicus chr. 6	EGRF-SP1F	370 - 390	(+)
YY1 transcription factor	EGRF-SP1F	426 - 450	(+)
**GXP_1417434** [GXP_1417434] (1 - 775)			
**Sfrs5**, GXL_10459, GeneID: 29667, Rattus norvegicus chr. 6<br>splicing factor, arginine/serine-rich 5	EGRF-SP1F	408 - 384	(-)
**GXP_1417644** [GXP_1417644] (1 - 601)			
**Begain**, GXL_10479, GeneID: 79146, Rattus norvegicus chr. 6<br>brain-enriched guanylate kinase-associated	EGRF-SP1F	270 - 249	(-)
**GXP_1168719** [GXP_1168719] (1 - 601)			
**Adcy3**, GXL_10517, GeneID: 64508, Rattus norvegicus chr. 6<br>adenylate cyclase 3	EGRF-SP1F	379 - 400	(+)
**GXP_1417058** [GXP_1417058] (1 - 944)			
**Adcy3**, GXL_10517, GeneID: 64508, Rattus norvegicus chr. 6<br>adenylate cyclase 3	EGRF-SP1F	179 - 157	(-)
**GXP_1414890** [GXP_1414890] (1 - 1046)	EGRF-SP1F	956 - 977	(+)
**Chn2**, GXL_15677, GeneID: 84031, Rattus norvegicus chr. 4			

chimerin (chimaerin) 2			
**GXP_1414892** [GXP_1414892] (1 - 601)	EGRF-SP1F	527 - 505	(-)
**Chn2**, GXL_15677, GeneID: 84031, Rattus norvegicus chr. 4			
chimerin (chimaerin) 2	EGRF-SP1F	529 - 507	(-)
**GXP_19429** [GXP_19429] (1 - 601)			
**Rtkn**, GXL_15689, GeneID: 297383, Rattus norvegicus chr. 4	EGRF-SP1F	495 - 515	(+)
rhotekin			
**GXP_19431** [GXP_19431] (1 - 624)			
**Sftpb**, GXL_15691, GeneID: 192155, Rattus norvegicus chr. 4	EGRF-SP1F	87 - 65	(-)
surfactant associated protein B			
**GXP_19451** [GXP_19451] (1 - 601)	EGRF-SP1F	90 - 112	(+)
**Oact5**, GXL_15710, GeneID: 362434, Rattus norvegicus chr. 4			
O-acyltransferase (membrane bound) domain containing 5	EGRF-SP1F	98 - 118	(+)
**GXP_19467** [GXP_19467] (1 - 640)			
**Npy**, GXL_15726, GeneID: 24604, Rattus norvegicus chr. 4	EGRF-SP1F	371 - 351	(-)
neuropeptide Y			
**GXP_1414757** [GXP_1414757] (1 - 601)			
**Tbxas1**, GXL_15732, GeneID: 24886, Rattus norvegicus chr. 4	EGRF-SP1F	250 - 270	(+)
thromboxane A synthase 1			
**GXP_19492** [GXP_19492] (1 - 601)			
**V1rb6**, GXL_15749, GeneID: 494310, Rattus norvegicus chr. 4	EGRF-SP1F	215 - 195	(-)
vomeronasal V1r-type receptor V1rb6			
**GXP_1415212** [GXP_1415212] (1 - 641)			
**Slc6a1**, GXL_15750, GeneID: 79212, Rattus norvegicus chr. 4	EGRF-SP1F	469 - 490	(+)
solute carrier family 6 (neurotransmitter transporter, GABA), member 1			
**GXP_19501** [GXP_19501] (1 - 761)			
**Cav2**, GXL_15758, GeneID: 363425, Rattus norvegicus chr. 4	EGRF-SP1F	444 - 464	(+)
caveolin 2			
	EGRF-SP1F	700 - 678	(-)
**GXP_19504** [GXP_19504] (1 - 1089)	EGRF-SP1F	901 - 877	(-)
**Ppp4r2_predicted**, GXL_15761, GeneID: 297486, Rattus norvegicus chr. 4	EGRF-SP1F	913 - 889	(-)
protein phosphatase 4, regulatory subunit 2 (predicted)	EGRF-SP1F	917 - 895	(-)
**GXP_1414450** [GXP_1414450] (1 - 1120)			
**Prkag2**, GXL_15763, GeneID: 373545, Rattus norvegicus chr. 4	EGRF-SP1F	855 - 877	(+)
protein kinase, AMP-activated, gamma 2 non-catalytic subunit			
**GXP_19524** [GXP_19524] (1 - 665)			
**Zfp384**, GXL_15781, GeneID: 171018, Rattus norvegicus chr. 4	EGRF-SP1F	375 - 397	(+)
zinc finger protein 384			
**GXP_515772** [GXP_515772] (1 - 668)	EGRF-SP1F	311 - 333	(+)

Zfp384, GXL_15781, GeneID: 171018, Rattus norvegicus chr. 4
zinc finger protein 384

**GXP_1415353** [GXP_1415353] (1 - 623)<br>**Zfp384**, GXL_15781, GeneID: 171018, Rattus norvegicus chr. 4<br>zinc finger protein 384	EGRF-SP1F	506 - 528	(+)
**GXP_1415354** [GXP_1415354] (1 - 838)<br>**Zfp384**, GXL_15781, GeneID: 171018, Rattus norvegicus chr. 4<br>zinc finger protein 384	EGRF-SP1F	93 - 115	(+)
**GXP_19534** [GXP_19534] (1 - 764)<br>**Arf5**, GXL_15791, GeneID: 79117, Rattus norvegicus chr. 4<br>ADP-ribosylation factor 5	EGRF-SP1F	500 - 477	(-)
**GXP_1414617** [GXP_1414617] (1 - 751)<br>**Met**, GXL_15792, GeneID: 24553, Rattus norvegicus chr. 4<br>met proto-oncogene	EGRF-SP1F	220 - 197	(-)
	EGRF-SP1F	272 - 292	(+)
**GXP_19543** [GXP_19543] (1 - 921)<br>**Bhlhb2**, GXL_15800, GeneID: 79431, Rattus norvegicus chr. 4<br>basic helix-loop-helix domain containing, class B2	EGRF-SP1F	253 - 229	(-)
	EGRF-SP1F	466 - 489	(+)
**GXP_860742** [GXP_860742] (1 - 601)<br>**Grid2**, GXL_15805, GeneID: 79220, Rattus norvegicus chr. 4<br>glutamate receptor, ionotropic, delta 2	EGRF-SP1F	265 - 287	(+)
**GXP_19550** [GXP_19550] (1 - 601)<br>**V1rb5**, GXL_15806, GeneID: 497787, Rattus norvegicus chr. 4<br>vomeronasal V1r-type receptor V1rb5	EGRF-SP1F	170 - 150	(-)
**GXP_19559** [GXP_19559] (1 - 981)<br>**Cyp51**, GXL_15815, GeneID: 25427, Rattus norvegicus chr. 4<br>cytochrome P450, subfamily 51	EGRF-SP1F	659 - 681	(+)
**GXP_19577** [GXP_19577] (1 - 1032)<br>**Tacr1**, GXL_15832, GeneID: 24807, Rattus norvegicus chr. 4<br>tachykinin receptor 1	EGRF-SP1F	121 - 143	(+)
**GXP_19587** [GXP_19587] (1 - 675)<br>**C1galt1**, GXL_15842, GeneID: 65044, Rattus norvegicus chr. 4<br>core 1 UDP-galactose:N-acetylgalactosamine-alpha-R beta 1,3-galactosyltransferase	EGRF-SP1F	448 - 468	(+)
**GXP_19599** [GXP_19599] (1 - 1396)<br>**Tm6p1**, GXL_15854, GeneID: 245966, Rattus norvegicus chr. 4<br>fasting-inducible integral membrane protein TM6P1	EGRF-SP1F	1114 - 1136	(+)
**GXP_19604** [GXP_19604] (1 - 851)<br>**Mfap5_predicted**, GXL_15859, GeneID: 362429, Rattus norvegicus chr. 4<br>microfibrillar associated protein 5 (predicted)	EGRF-SP1F	632 - 652	(+)
	EGRF-SP1F	634 - 654	(+)
**GXP_1166289** [GXP_1166289] (1 - 601)<br>**Mfap5_predicted**, GXL_15859, GeneID: 362429, Rattus norvegicus chr. 4	EGRF-SP1F	195 - 172	(-)

microfibrillar associated protein 5 (predicted)			
**GXP_19608** [GXP_19608] (1 - 663)<br>**Tmub1**, GXL_15863, GeneID: 362301, Rattus norvegicus chr. 4<br>transmembrane and ubiquitin-like domain containing 1	EGRF-SP1F	390 - 368	(-)
	EGRF-SP1F	397 - 377	(-)
**GXP_1415018** [GXP_1415018] (1 - 601)<br>**Dctn1**, GXL_15878, GeneID: 29167, Rattus norvegicus chr. 4<br>dynactin 1	EGRF-SP1F	424 - 445	(+)
**GXP_19631** [GXP_19631] (1 - 691)<br>**Mlf2_predicted**, GXL_15886, GeneID: 312709, Rattus norvegicus chr. 4<br>myeloid leukemia factor 2 (predicted)	EGRF-SP1F	427 - 448	(+)
**GXP_19645** [GXP_19645] (1 - 601)<br>**Syn2**, GXL_15900, GeneID: 29179, Rattus norvegicus chr. 4<br>synapsin II	EGRF-SP1F	145 - 123	(-)
**GXP_19646** [GXP_19646] (1 - 601)<br>**Fkbp9**, GXL_15901, GeneID: 297123, Rattus norvegicus chr. 4<br>FK506 binding protein 9	EGRF-SP1F	524 - 502	(-)
**GXP_19652** [GXP_19652] (1 - 601)<br>**Casp2**, GXL_15907, GeneID: 64314, Rattus norvegicus chr. 4<br>caspase 2	EGRF-SP1F	442 - 419	(-)
**GXP_1414477** [GXP_1414477] (1 - 602)<br>**Kcnh2**, GXL_15922, GeneID: 117018, Rattus norvegicus chr. 4<br>potassium voltage-gated channel, subfamily H (eag-related), member 2	EGRF-SP1F	560 - 537	(-)
	EGRF-SP1F	566 - 542	(-)
**GXP_1414479** [GXP_1414479] (1 - 622)<br>**Kcnh2**, GXL_15922, GeneID: 117018, Rattus norvegicus chr. 4<br>potassium voltage-gated channel, subfamily H (eag-related), member 2	EGRF-SP1F	483 - 463	(-)
**GXP_19692** [GXP_19692] (1 - 753)<br>**Retsat**, GXL_15947, GeneID: 246298, Rattus norvegicus chr. 4<br>all-trans-13,14-dihydroretinol saturase	EGRF-SP1F	320 - 344	(+)
**GXP_860704** [GXP_860704] (1 - 601)<br>**Cr16**, GXL_15950, GeneID: 259242, Rattus norvegicus chr. 4<br>SH3 domain binding protein CR16	EGRF-SP1F	104 - 80	(-)
	EGRF-SP1F	106 - 82	(-)
**GXP_1414894** [GXP_1414894] (1 - 601)<br>**Cr16**, GXL_15950, GeneID: 259242, Rattus norvegicus chr. 4<br>SH3 domain binding protein CR16	EGRF-SP1F	97 - 117	(+)
**GXP_19700** [GXP_19700] (1 - 601)<br>**Smarcd3**, GXL_15955, GeneID: 296732, Rattus norvegicus chr. 4<br>SWI/SNF related, matrix associated, actin dependent regulator of chromatin, subfamily d, member 3	EGRF-SP1F	415 - 437	(+)
	EGRF-SP1F	419 - 439	(+)
	EGRF-SP1F	422 - 443	(+)
	EGRF-SP1F	424 - 445	(+)
**GXP_1414457** [GXP_1414457] (1 - 1011)	EGRF-SP1F	735 - 713	(-)

**Smarcd3**, GXL_15955, GeneID: 296732, Rattus norvegicus chr. 4<br>SWI/SNF related, matrix associated, actin dependent regulator of chromatin, subfamily d, member 3	EGRF-SP1F	738 - 717	(-)
**GXP_19728** [GXP_19728] (1 - 697)<br>**Akap3**, GXL_15982, GeneID: 312720, Rattus norvegicus chr. 4<br>A kinase (PRKA) anchor protein 3	EGRF-SP1F	30 - 10	(-)
**GXP_19752** [GXP_19752] (1 - 716)<br>**Cxcl12**, GXL_16006, GeneID: 24772, Rattus norvegicus chr. 4<br>chemokine (C-X-C motif) ligand 12	EGRF-SP1F	593 - 613	(+)
**GXP_1415200** [GXP_1415200] (1 - 602)<br>**Il17re**, GXL_16035, GeneID: 362417, Rattus norvegicus chr. 4<br>interleukin 17 receptor E	EGRF-SP1F	570 - 590	(+)
**GXP_19786** [GXP_19786] (1 - 608)<br>**Mrps35_predicted**, GXL_16040, GeneID: 297727, Rattus norvegicus chr. 4<br>mitochondrial ribosomal protein S35 (predicted)	EGRF-SP1F	474 - 496	(+)
**GXP_1415225** [GXP_1415225] (1 - 601)<br>**Pparg**, GXL_16044, GeneID: 25664, Rattus norvegicus chr. 4<br>peroxisome proliferator activated receptor gamma	EGRF-SP1F	324 - 301	(-)
**GXP_19797** [GXP_19797] (1 - 897)<br>**Reln**, GXL_16051, GeneID: 24718, Rattus norvegicus chr. 4<br>reelin	EGRF-SP1F	350 - 330	(-)
**GXP_19798** [GXP_19798] (1 - 788)<br>**Slc6a11**, GXL_16052, GeneID: 79213, Rattus norvegicus chr. 4<br>solute carrier family 6 (neurotransmitter transporter, GABA), member 11	EGRF-SP1F<br>EGRF-SP1F	412 - 392<br>652 - 631	(-)<br>(-)
**GXP_1415019** [GXP_1415019] (1 - 603)<br>**Slc4a5**, GXL_16055, GeneID: 297386, Rattus norvegicus chr. 4<br>solute carrier family 4, sodium bicarbonate cotransporter, member 5	EGRF-SP1F	242 - 220	(-)
**GXP_1414849** [GXP_1414849] (1 - 619)<br>**Abp1**, GXL_16063, GeneID: 65029, Rattus norvegicus chr. 4<br>amiloride binding protein 1 (amine oxidase, copper-containing)	EGRF-SP1F	423 - 445	(+)
**GXP_19810** [GXP_19810] (1 - 712)<br>**Ptpn12**, GXL_16064, GeneID: 117255, Rattus norvegicus chr. 4<br>protein tyrosine phosphatase, non-receptor type 12	EGRF-SP1F<br>EGRF-SP1F<br>EGRF-SP1F	337 - 317<br>572 - 552<br>636 - 613	(-)<br>(-)<br>(-)
**GXP_860371** [GXP_860371] (1 - 601)<br>**Ptpn12**, GXL_16064, GeneID: 117255, Rattus norvegicus chr. 4<br>protein tyrosine phosphatase, non-receptor type 12	EGRF-SP1F	562 - 542	(-)
**GXP_19824** [GXP_19824] (1 - 601)<br>**Gpr27**, GXL_16078, GeneID: 65275, Rattus norvegicus chr. 4<br>G protein-coupled receptor 27	EGRF-SP1F	541 - 517	(-)
**GXP_19832** [GXP_19832] (1 - 637)<br>**Spsb2**, GXL_16086, GeneID: 297592, Rattus norvegicus chr. 4	EGRF-SP1F	472 - 449	(-)

splA/ryanodine receptor domain and SOCS box containing 2			
**GXP_1415343** [GXP_1415343] (1 - 1155)<br>**Spsb2**, GXL_16086, GeneID: 297592, Rattus norvegicus chr. 4<br>splA/ryanodine receptor domain and SOCS box containing 2	EGRF-SP1F	162 - 139	(-)
**GXP_19844** [GXP_19844] (1 - 700)<br>**Foxm1**, GXL_16098, GeneID: 58921, Rattus norvegicus chr. 4<br>forkhead box M1	EGRF-SP1F	537 - 560	(+)
**GXP_19845** [GXP_19845] (1 - 710)<br>**Bcl2l13_predicted**, GXL_16099, GeneID: 312682, Rattus norvegicus chr. 4<br>BCL2-like 13 (apoptosis facilitator) (predicted)	EGRF-SP1F	464 - 487	(+)
**GXP_515687** [GXP_515687] (1 - 601)<br>**Bcl2l13_predicted**, GXL_16099, GeneID: 312682, Rattus norvegicus chr. 4<br>BCL2-like 13 (apoptosis facilitator) (predicted)	EGRF-SP1F	240 - 260	(+)
**GXP_19849** [GXP_19849] (1 - 601)<br>**RSA-14-44**, GXL_16103, GeneID: 297173, Rattus norvegicus chr. 4<br>RSA-14-44 protein	EGRF-SP1F	41 - 63	(+)
**GXP_22666** [GXP_22666] (1 - 618)<br>**Prkab2**, GXL_18682, GeneID: 64562, Rattus norvegicus chr. 2<br>protein kinase, AMP-activated, beta 2 non-catalytic subunit	EGRF-SP1F	450 - 427	(-)
**GXP_22677** [GXP_22677] (1 - 1217)<br>**Cryz**, GXL_18692, GeneID: 362061, Rattus norvegicus chr. 2<br>crystallin, zeta	EGRF-SP1F<br>EGRF-SP1F<br>EGRF-SP1F	68 - 48<br>626 - 603<br>628 - 608	(-)<br>(-)<br>(-)
**GXP_22685** [GXP_22685] (1 - 833)<br>**Gclm**, GXL_18700, GeneID: 29739, Rattus norvegicus chr. 2<br>glutamate cysteine ligase, modifier subunit	EGRF-SP1F<br>EGRF-SP1F	407 - 430<br>413 - 433	(+)<br>(+)
**GXP_22690** [GXP_22690] (1 - 602)<br>**Cxxc4**, GXL_18705, GeneID: 83824, Rattus norvegicus chr. 2<br>CXXC finger 4	EGRF-SP1F<br>EGRF-SP1F<br>EGRF-SP1F	355 - 331<br>376 - 352<br>379 - 358	(-)<br>(-)<br>(-)
**GXP_1412759** [GXP_1412759] (1 - 719)<br>**Cxxc4**, GXL_18705, GeneID: 83824, Rattus norvegicus chr. 2<br>CXXC finger 4	EGRF-SP1F	215 - 193	(-)
**GXP_22724** [GXP_22724] (1 - 824)<br>**Cct3**, GXL_18739, GeneID: 295230, Rattus norvegicus chr. 2<br>chaperonin subunit 3 (gamma)	EGRF-SP1F	124 - 101	(-)
**GXP_22737** [GXP_22737] (1 - 1385)<br>**Pias3**, GXL_18752, GeneID: 83614, Rattus norvegicus chr. 2<br>protein inhibitor of activated STAT 3	EGRF-SP1F	1189 - 1167	(-)
**GXP_22740** [GXP_22740] (1 - 1052)<br>**Tspan5**, GXL_18755, GeneID: 362048, Rattus norvegicus chr. 2	EGRF-SP1F	600 - 579	(-)

tetraspanin 5			
**GXP_1412790** [GXP_1412790] (1 - 602)	EGRF-SP1F	312 - 292	(-)
**Tspan5**, GXL_18755, GeneID: 362048, Rattus norvegicus chr. 2			
tetraspanin 5	EGRF-SP1F	319 - 296	(-)
**GXP_22752** [GXP_22752] (1 - 789)	EGRF-SP1F	545 - 522	(-)
**Ubqln4_predicted**, GXL_18767, GeneID: 310633, Rattus norvegicus chr. 2			
ubiquilin 4 (predicted)	EGRF-SP1F	547 - 527	(-)
**GXP_22767** [GXP_22767] (1 - 646)			
**Adar**, GXL_18782, GeneID: 81635, Rattus norvegicus chr. 2	EGRF-SP1F	451 - 474	(+)
adenosine deaminase, RNA-specific			
**GXP_22772** [GXP_22772] (1 - 740)			
**Crabp2**, GXL_18787, GeneID: 29563, Rattus norvegicus chr. 2	EGRF-SP1F	452 - 429	(-)
cellular retinoic acid binding protein 2			
**GXP_22775** [GXP_22775] (1 - 839)	EGRF-SP1F	2 - 22	(+)
**Sf3b4**, GXL_18790, GeneID: 295270, Rattus norvegicus chr. 2			
splicing factor 3b, subunit 4	EGRF-SP1F	21 - 45	(+)
**GXP_22776** [GXP_22776] (1 - 1247)	EGRF-SP1F	488 - 464	(-)
**Ppp3ca**, GXL_18791, GeneID: 24674, Rattus norvegicus chr. 2	EGRF-SP1F	776 - 799	(+)
protein phosphatase 3, catalytic subunit, alpha isoform	EGRF-SP1F	896 - 919	(+)
**GXP_1412772** [GXP_1412772] (1 - 605)			
**Ppp3ca**, GXL_18791, GeneID: 24674, Rattus norvegicus chr. 2	EGRF-SP1F	127 - 107	(-)
protein phosphatase 3, catalytic subunit, alpha isoform			
**GXP_22806** [GXP_22806] (1 - 665)	EGRF-SP1F	343 - 365	(+)
**Plrg1**, GXL_18820, GeneID: 60376, Rattus norvegicus chr. 2			
pleiotropic regulator 1, PRL1 homolog (Arabidopsis)	EGRF-SP1F	353 - 377	(+)
**GXP_22820** [GXP_22820] (1 - 1214)	EGRF-SP1F	757 - 737	(-)
**Lmo4**, GXL_18834, GeneID: 362051, Rattus norvegicus chr. 2			
LIM domain only 4	EGRF-SP1F	1037 - 1058	(+)
**GXP_1412808** [GXP_1412808] (1 - 634)			
**Lmo4**, GXL_18834, GeneID: 362051, Rattus norvegicus chr. 2	EGRF-SP1F	465 - 485	(+)
LIM domain only 4			
	EGRF-SP1F	171 - 191	(+)
**GXP_1412809** [GXP_1412809] (1 - 961)	EGRF-SP1F	430 - 408	(-)
**Lmo4**, GXL_18834, GeneID: 362051, Rattus norvegicus chr. 2			
LIM domain only 4	EGRF-SP1F	538 - 514	(-)
	EGRF-SP1F	783 - 763	(-)
**GXP_22838** [GXP_22838] (1 - 914)			
**Negr1**, GXL_18852, GeneID: 59318, Rattus norvegicus chr. 2	EGRF-SP1F	666 - 646	(-)
neuronal growth regulator 1			
	EGRF-SP1F	206 - 229	(+)

Sequence	Matrix	Position	Strand
**GXP_22839** [GXP_22839] (1 - 602)<br>**Pdgfc**, GXL_18853, GeneID: 79429, Rattus norvegicus chr. 2<br>platelet-derived growth factor, C polypeptide	EGRF-SP1F	211 - 231	(+)
**GXP_22841** [GXP_22841] (1 - 667)<br>**Pla2g12a_predicted**, GXL_18855, GeneID: 362039, Rattus norvegicus chr. 2<br>phospholipase A2, group XIIA (predicted)	EGRF-SP1F	569 - 589	(+)
	EGRF-SP1F	599 - 623	(+)
**GXP_22859** [GXP_22859] (1 - 670)<br>**S100a10**, GXL_18873, GeneID: 81778, Rattus norvegicus chr. 2<br>S100 calcium binding protein A10 (calpactin)	EGRF-SP1F	431 - 408	(-)
**GXP_1412674** [GXP_1412674] (1 - 602)<br>**Cnn3**, GXL_18894, GeneID: 54321, Rattus norvegicus chr. 2<br>calponin 3, acidic	EGRF-SP1F	434 - 414	(-)
**GXP_22885** [GXP_22885] (1 - 1045)<br>**Pde5a**, GXL_18899, GeneID: 171115, Rattus norvegicus chr. 2<br>phosphodiesterase 5A, cGMP-specific	EGRF-SP1F	348 - 328	(-)
**GXP_22886** [GXP_22886] (1 - 601)<br>**S100a6**, GXL_18900, GeneID: 85247, Rattus norvegicus chr. 2<br>S100 calcium binding protein A6 (calcyclin)	EGRF-SP1F	410 - 387	(-)
**GXP_1412243** [GXP_1412243] (1 - 658)<br>**Accn5**, GXL_18906, GeneID: 63866, Rattus norvegicus chr. 2<br>amiloride-sensitive cation channel 5, intestinal	EGRF-SP1F	366 - 387	(+)
**GXP_22897** [GXP_22897] (1 - 601)<br>**Arhgap29**, GXL_18911, GeneID: 310833, Rattus norvegicus chr. 2<br>Rho GTPase activating protein 29	EGRF-SP1F	418 - 395	(-)
**GXP_22899** [GXP_22899] (1 - 1071)<br>**Rsbn1_predicted**, GXL_18913, GeneID: 310749, Rattus norvegicus chr. 2<br>rosbin, round spermatid basic protein 1 (predicted)	EGRF-SP1F	719 - 699	(-)
	EGRF-SP1F	1028 - 1007	(-)
**GXP_22901** [GXP_22901] (1 - 823)<br>**Tspan2**, GXL_18915, GeneID: 64521, Rattus norvegicus chr. 2<br>tetraspanin 2	EGRF-SP1F	638 - 660	(+)
**GXP_1412514** [GXP_1412514] (1 - 602)<br>**Tspan2**, GXL_18915, GeneID: 64521, Rattus norvegicus chr. 2<br>tetraspanin 2	EGRF-SP1F	194 - 216	(+)
**GXP_1412515** [GXP_1412515] (1 - 602)<br>**Tspan2**, GXL_18915, GeneID: 64521, Rattus norvegicus chr. 2<br>tetraspanin 2	EGRF-SP1F	194 - 172	(-)
**GXP_22907** [GXP_22907] (1 - 847)<br>**Prss12**, GXL_18921, GeneID: 85266, Rattus norvegicus chr. 2<br>peptidase, serine, 12 (neurotrypsin, motopsin)	EGRF-SP1F	677 - 699	(+)
	EGRF-SP1F	821 - 844	(+)
	EGRF-SP1F	298 - 278	(-)

Sequence	Matrix	Position	Strand
**GXP_22926** [GXP_22926] (1 - 601)<br>**Tram1l1_predicted**, GXL_18940, GeneID: 310846, Rattus norvegicus chr. 2<br>translocation associated membrane protein 1-like 1 (predicted)			
**GXP_1412530** [GXP_1412530] (1 - 606)<br>**Syt6**, GXL_18958, GeneID: 60565, Rattus norvegicus chr. 2<br>synaptotagmin VI	EGRF-SP1F	275 - 251	(-)
**GXP_1162606** [GXP_1162606] (1 - 1251)<br>**Pitx2**, GXL_18962, GeneID: 54284, Rattus norvegicus chr. 2<br>paired-like homeodomain transcription factor 2	EGRF-SP1F	112 - 92	(-)
	EGRF-SP1F	638 - 616	(-)
**GXP_22950** [GXP_22950] (1 - 601)<br>**Isg20l2**, GXL_18964, GeneID: 361977, Rattus norvegicus chr. 2<br>interferon stimulated exonuclease gene 20-like 2	EGRF-SP1F	519 - 543	(+)
**GXP_22965** [GXP_22965] (1 - 974)<br>**Unc5c**, GXL_18979, GeneID: 362049, Rattus norvegicus chr. 2<br>unc-5 homolog C (C. elegans)	EGRF-SP1F	407 - 385	(-)
**GXP_1162377** [GXP_1162377] (1 - 835)<br>**Kcnd3**, GXL_19014, GeneID: 65195, Rattus norvegicus chr. 2<br>potassium voltage gated channel, Shal-related family, member 3	EGRF-SP1F	27 - 49	(+)
	EGRF-SP1F	465 - 488	(+)
**GXP_1412560** [GXP_1412560] (1 - 601)<br>**Kcnd3**, GXL_19014, GeneID: 65195, Rattus norvegicus chr. 2<br>potassium voltage gated channel, Shal-related family, member 3	EGRF-SP1F	106 - 129	(+)
**GXP_23001** [GXP_23001] (1 - 893)<br>**Sep15**, GXL_19015, GeneID: 113922, Rattus norvegicus chr. 2<br>selenoprotein	EGRF-SP1F	290 - 313	(+)
**GXP_23003** [GXP_23003] (1 - 835)<br>**Notch2**, GXL_19017, GeneID: 29492, Rattus norvegicus chr. 2<br>notch gene homolog 2 (Drosophila)	EGRF-SP1F	280 - 258	(-)
**GXP_23004** [GXP_23004] (1 - 601)<br>**Map2k1ip1**, GXL_19018, GeneID: 362045, Rattus norvegicus chr. 2<br>mitogen-activated protein kinase kinase 1 interacting protein 1	EGRF-SP1F	390 - 368	(-)
**GXP_1412777** [GXP_1412777] (1 - 602)<br>**Map2k1ip1**, GXL_19018, GeneID: 362045, Rattus norvegicus chr. 2<br>mitogen-activated protein kinase kinase 1 interacting protein 1	EGRF-SP1F	96 - 74	(-)
**GXP_25354** [GXP_25354] (1 - 690)<br>**Ptpn1**, GXL_21003, GeneID: 24697, Rattus norvegicus chr. 3<br>protein tyrosine phosphatase, non-receptor type 1	EGRF-SP1F	260 - 284	(+)
	EGRF-SP1F	412 - 434	(+)
	EGRF-SP1F	416 - 439	(+)
**GXP_25360** [GXP_25360] (1 - 601)<br>**Plcg1**, GXL_21008, GeneID: 25738, Rattus norvegicus chr. 3<br>phospholipase C, gamma 1	EGRF-SP1F	540 - 561	(+)
	EGRF-SP1F	609 - 632	(+)

**GXP_25362** [GXP_25362] (1 - 1338)<br>**Slc32a1**, GXL_21010, GeneID: 83612, Rattus norvegicus chr. 3<br>solute carrier family 32 (GABA vesicular transporter), member 1			
**GXP_25370** [GXP_25370] (1 - 709)<br>**Gdap1l1_predicted**, GXL_21018, GeneID: 311616, Rattus norvegicus chr. 3<br>ganglioside-induced differentiation-associated protein 1-like 1 (predicted)	EGRF-SP1F	521 - 499	(-)
**GXP_25374** [GXP_25374] (1 - 772)<br>**Pofut1**, GXL_21022, GeneID: 311551, Rattus norvegicus chr. 3<br>protein O-fucosyltransferase 1	EGRF-SP1F	312 - 332	(+)
	EGRF-SP1F	314 - 337	(+)
	EGRF-SP1F	619 - 640	(+)
**GXP_25383** [GXP_25383] (1 - 889)<br>**Tcfap2c**, GXL_21031, GeneID: 362280, Rattus norvegicus chr. 3<br>transcription factor AP-2, gamma	EGRF-SP1F	133 - 111	(-)
	EGRF-SP1F	748 - 728	(-)
**GXP_1414342** [GXP_1414342] (1 - 602)<br>**Tcfap2c**, GXL_21031, GeneID: 362280, Rattus norvegicus chr. 3<br>transcription factor AP-2, gamma	EGRF-SP1F	168 - 146	(-)
**GXP_1414141** [GXP_1414141] (1 - 611)<br>**A**, GXL_21039, GeneID: 24152, Rattus norvegicus chr. 3<br>agouti	EGRF-SP1F	455 - 477	(+)
**GXP_25392** [GXP_25392] (1 - 698)<br>**Epb4.1l1**, GXL_21040, GeneID: 59317, Rattus norvegicus chr. 3<br>erythrocyte protein band 4.1-like 1	EGRF-SP1F	274 - 296	(+)
	EGRF-SP1F	614 - 637	(+)
**GXP_1414183** [GXP_1414183] (1 - 796)<br>**Epb4.1l1**, GXL_21040, GeneID: 59317, Rattus norvegicus chr. 3<br>erythrocyte protein band 4.1-like 1	EGRF-SP1F	634 - 613	(-)
**GXP_25402** [GXP_25402] (1 - 766)<br>**Hnf4a**, GXL_21050, GeneID: 25735, Rattus norvegicus chr. 3<br>hepatocyte nuclear factor 4, alpha	EGRF-SP1F	414 - 437	(+)
**GXP_25418** [GXP_25418] (1 - 883)<br>**Ss18l1**, GXL_21066, GeneID: 192352, Rattus norvegicus chr. 3<br>synovial sarcoma translocation gene on chromosome 18-like 1	EGRF-SP1F	404 - 425	(+)
	EGRF-SP1F	682 - 706	(+)
**GXP_25421** [GXP_25421] (1 - 639)<br>**Rps21**, GXL_21069, GeneID: 81775, Rattus norvegicus chr. 3<br>ribosomal protein S21	EGRF-SP1F	310 - 290	(-)
**GXP_1414360** [GXP_1414360] (1 - 603)<br>**Rps21**, GXL_21069, GeneID: 81775, Rattus norvegicus chr. 3<br>ribosomal protein S21	EGRF-SP1F	75 - 55	(-)
**GXP_25426** [GXP_25426] (1 - 642)<br>**Trp53rk_predicted**, GXL_21074, GeneID: 362272, Rattus norvegicus chr. 3<br>TP53 regulating kinase (predicted)	EGRF-SP1F	469 - 447	(-)
	EGRF-SP1F	472 - 452	(-)
	EGRF-SP1F	692 - 672	(-)

**GXP_25430** [GXP_25430] (1 - 733)<br>**Zgpat**, GXL_21078, GeneID: 296478, Rattus norvegicus chr. 3<br>zinc finger, CCCH-type with G patch domain			
**GXP_1164963** [GXP_1164963] (1 - 604)<br>**Zgpat**, GXL_21078, GeneID: 296478, Rattus norvegicus chr. 3<br>zinc finger, CCCH-type with G patch domain	EGRF-SP1F	405 - 425	(+)
**GXP_1414207** [GXP_1414207] (1 - 636)<br>**Src**, GXL_21079, GeneID: 83805, Rattus norvegicus chr. 3<br>Rous sarcoma oncogene	EGRF-SP1F	326 - 305	(-)
**GXP_25434** [GXP_25434] (1 - 802)<br>**Bpi**, GXL_21082, GeneID: 296321, Rattus norvegicus chr. 3<br>bactericidal/permeability-increasing protein	EGRF-SP1F	568 - 588	(+)
**GXP_1414219** [GXP_1414219] (1 - 601)<br>**Bpi**, GXL_21082, GeneID: 296321, Rattus norvegicus chr. 3<br>bactericidal/permeability-increasing protein	EGRF-SP1F	336 - 313	(-)
**GXP_25447** [GXP_25447] (1 - 801)<br>**Xkr7**, GXL_21095, GeneID: 311549, Rattus norvegicus chr. 3<br>X Kell blood group precursor related family member 7 homolog (human)	EGRF-SP1F<br>EGRF-SP1F<br>EGRF-SP1F	473 - 495<br>790 - 767<br>792 - 772	(+)<br>(-)<br>(-)
**GXP_25462** [GXP_25462] (1 - 929)<br>**Bwk1**, GXL_21110, GeneID: 296466, Rattus norvegicus chr. 3<br>Bwk1 leukemia-related gene	EGRF-SP1F<br>EGRF-SP1F	731 - 754<br>803 - 826	(+)<br>(+)
**GXP_1164639** [GXP_1164639] (1 - 601)<br>**Dlgap4**, GXL_21115, GeneID: 286930, Rattus norvegicus chr. 3<br>discs, large homolog-associated protein 4 (Drosophila)	EGRF-SP1F	326 - 347	(+)
**GXP_1164641** [GXP_1164641] (1 - 601)<br>**Dlgap4**, GXL_21115, GeneID: 286930, Rattus norvegicus chr. 3<br>discs, large homolog-associated protein 4 (Drosophila)	EGRF-SP1F	443 - 466	(+)
**GXP_25490** [GXP_25490] (1 - 769)<br>**Arfgef2**, GXL_21138, GeneID: 296380, Rattus norvegicus chr. 3<br>ADP-ribosylation factor guanine nucleotide-exchange factor 2 (brefeldin A-inhibited)	EGRF-SP1F	363 - 387	(+)
**GXP_25506** [GXP_25506] (1 - 601)<br>**Top1**, GXL_21154, GeneID: 64550, Rattus norvegicus chr. 3<br>topoisomerase (DNA) I	EGRF-SP1F	290 - 312	(+)
**GXP_25509** [GXP_25509] (1 - 605)<br>**Dnmt3b**, GXL_21157, GeneID: 444985, Rattus norvegicus chr. 3<br>DNA methyltransferase 3B	EGRF-SP1F	423 - 400	(-)
**GXP_25517** [GXP_25517] (1 - 790)<br>**Vapb**, GXL_21165, GeneID: 60431, Rattus norvegicus chr. 3<br>vesicle-associated membrane protein, associated protein B and C	EGRF-SP1F<br>EGRF-SP1F<br>EGRF-SP1F	607 - 629<br>617 - 641<br>155 - 178	(+)<br>(+)<br>(+)

**GXP_27556** [GXP_27556] (1 - 748)<br>**Psap**, GXL_23131, GeneID: 25524, Rattus norvegicus chr. 20<br>prosaposin	EGRF-SP1F	480 - 500	(+)
**GXP_27566** [GXP_27566] (1 - 768)<br>**Cd164**, GXL_23141, GeneID: 83689, Rattus norvegicus chr. 20<br>CD164 antigen	EGRF-SP1F	465 - 488	(+)
**GXP_27578** [GXP_27578] (1 - 785)<br>**Prep**, GXL_23153, GeneID: 83471, Rattus norvegicus chr. 20<br>prolyl endopeptidase	EGRF-SP1F	187 - 166	(-)
**GXP_27620** [GXP_27620] (1 - 920)<br>**Fabp7**, GXL_23195, GeneID: 80841, Rattus norvegicus chr. 20<br>fatty acid binding protein 7, brain	EGRF-SP1F	618 - 638	(+)
**GXP_27621** [GXP_27621] (1 - 615)<br>**Cd24**, GXL_23196, GeneID: 25145, Rattus norvegicus chr. 20<br>CD24 antigen	EGRF-SP1F	197 - 176	(-)
**GXP_27627** [GXP_27627] (1 - 691)<br>**Gja1**, GXL_23202, GeneID: 24392, Rattus norvegicus chr. 20<br>gap junction membrane channel protein alpha 1	EGRF-SP1F	178 - 198	(+)
**GXP_27631** [GXP_27631] (1 - 601)<br>**Neurog3**, GXL_23206, GeneID: 60329, Rattus norvegicus chr. 20<br>neurogenin 3	EGRF-SP1F<br>EGRF-SP1F	403 - 380<br>491 - 469	(-)<br>(-)
**GXP_45150** [GXP_45150] (1 - 802)<br>**Fnbp1**, GXL_37299, GeneID: 192348, Rattus norvegicus chr. 3<br>formin binding protein 1	EGRF-SP1F<br>EGRF-SP1F<br>EGRF-SP1F	450 - 472<br>472 - 494<br>771 - 751	(+)<br>(+)<br>(-)
**GXP_1413530** [GXP_1413530] (1 - 601)<br>**Gyltl1b**, GXL_37302, GeneID: 311202, Rattus norvegicus chr. 3<br>glycosyltransferase-like 1B	EGRF-SP1F	163 - 183	(+)
**GXP_45169** [GXP_45169] (1 - 676)<br>**Ndufs3_predicted**, GXL_37318, GeneID: 295923, Rattus norvegicus chr. 3<br>NADH dehydrogenase (ubiquinone) Fe-S protein 3 (predicted)	EGRF-SP1F	210 - 234	(+)
**GXP_45172** [GXP_45172] (1 - 663)<br>**Ptges**, GXL_37320, GeneID: 59103, Rattus norvegicus chr. 3<br>prostaglandin E synthase	EGRF-SP1F	472 - 449	(-)
**GXP_45174** [GXP_45174] (1 - 601)<br>**Nfe2l2**, GXL_37322, GeneID: 83619, Rattus norvegicus chr. 3<br>nuclear factor, erythroid derived 2, like 2	EGRF-SP1F	433 - 410	(-)
**GXP_1413439** [GXP_1413439] (1 - 626)<br>**Tmx2**, GXL_37330, GeneID: 295701, Rattus norvegicus chr. 3<br>thioredoxin-related transmembrane protein 2	EGRF-SP1F<br>EGRF-SP1F	38 - 18<br>425 - 402	(-)<br>(-)

Sequence	Matrix	Position	Strand
**GXP_45200** [GXP_45200] (1 - 601)<br>**Atp5g3**, GXL_37348, GeneID: 114630, Rattus norvegicus chr. 3<br>ATP synthase, H+ transporting, mitochondrial F0 complex, subunit c (subunit 9) isoform 3			
**GXP_1413375** [GXP_1413375] (1 - 955)<br>**Atp5g3**, GXL_37348, GeneID: 114630, Rattus norvegicus chr. 3<br>ATP synthase, H+ transporting, mitochondrial F0 complex, subunit c (subunit 9) isoform 3	EGRF-SP1F	644 - 621	(-)
**GXP_45219** [GXP_45219] (1 - 601)<br>**Tmem15_predicted**, GXL_37367, GeneID: 311847, Rattus norvegicus chr. 3<br>transmembrane protein 15 (predicted)	EGRF-SP1F	307 - 330	(+)
**GXP_45246** [GXP_45246] (1 - 897)<br>**Kcnh7**, GXL_37394, GeneID: 170739, Rattus norvegicus chr. 3<br>potassium voltage-gated channel, subfamily H (eag-related), member 7	EGRF-SP1F	534 - 557	(+)
	EGRF-SP1F	541 - 562	(+)
**GXP_45266** [GXP_45266] (1 - 655)<br>**Lcn2**, GXL_37414, GeneID: 170496, Rattus norvegicus chr. 3<br>lipocalin 2	EGRF-SP1F	142 - 120	(-)
	EGRF-SP1F	216 - 193	(-)
	EGRF-SP1F	222 - 198	(-)
	EGRF-SP1F	307 - 327	(+)
**GXP_1163336** [GXP_1163336] (1 - 710)<br>**Cacnb4**, GXL_37423, GeneID: 58942, Rattus norvegicus chr. 3<br>calcium channel, voltage-dependent, beta 4 subunit	EGRF-SP1F	448 - 469	(+)
	EGRF-SP1F	523 - 543	(+)
**GXP_1413218** [GXP_1413218] (1 - 663)<br>**Cacnb4**, GXL_37423, GeneID: 58942, Rattus norvegicus chr. 3<br>calcium channel, voltage-dependent, beta 4 subunit	EGRF-SP1F	188 - 209	(+)
	EGRF-SP1F	263 - 283	(+)
**GXP_45279** [GXP_45279] (1 - 849)<br>**Fibcd1_predicted**, GXL_37427, GeneID: 311861, Rattus norvegicus chr. 3<br>fibrinogen C domain containing 1 (predicted)	EGRF-SP1F	706 - 729	(+)
**GXP_45284** [GXP_45284] (1 - 894)<br>**Ubadc1**, GXL_37432, GeneID: 362087, Rattus norvegicus chr. 3<br>ubiquitin associated domain containing 1	EGRF-SP1F	620 - 597	(-)
**GXP_45311** [GXP_45311] (1 - 1418)<br>**Cdk9**, GXL_37459, GeneID: 362110, Rattus norvegicus chr. 3<br>cyclin-dependent kinase 9 (CDC2-related kinase)	EGRF-SP1F	219 - 199	(-)
	EGRF-SP1F	897 - 873	(-)
**GXP_45327** [GXP_45327] (1 - 784)<br>**Psmd5_predicted**, GXL_37475, GeneID: 296651, Rattus norvegicus chr. 3<br>proteasome (prosome, macropain) 26S subunit, non-ATPase, 5 (predicted)	EGRF-SP1F	112 - 136	(+)
	EGRF-SP1F	118 - 138	(+)
**GXP_1413607** [GXP_1413607] (1 - 601)<br>**Hipk3**, GXL_37476, GeneID: 83617, Rattus norvegicus chr. 3<br>homeodomain interacting protein kinase 3	EGRF-SP1F	386 - 406	(+)
**GXP_45331** [GXP_45331] (1 - 850)<br>**Arl5a**, GXL_37479, GeneID: 117050, Rattus norvegicus chr. 3<br>ADP-ribosylation factor-like 5A	EGRF-SP1F	563 - 583	(+)
	EGRF-SP1F	571 - 591	(+)

	EGRF-SP1F	579 - 603	(+)
**GXP_45333** [GXP_45333] (1 - 918)<br>**Grin1**, GXL_37481, GeneID: 24408, Rattus norvegicus chr. 3<br>glutamate receptor, ionotropic, N-methyl D-aspartate 1	EGRF-SP1F	541 - 518	(-)
**GXP_1412915** [GXP_1412915] (1 - 602)<br>**Grin1**, GXL_37481, GeneID: 24408, Rattus norvegicus chr. 3<br>glutamate receptor, ionotropic, N-methyl D-aspartate 1	EGRF-SP1F	142 - 163	(+)
**GXP_1412916** [GXP_1412916] (1 - 601)<br>**Grin1**, GXL_37481, GeneID: 24408, Rattus norvegicus chr. 3<br>glutamate receptor, ionotropic, N-methyl D-aspartate 1	EGRF-SP1F	391 - 368	(-)
**GXP_1163272** [GXP_1163272] (1 - 601)<br>**Rpl35**, GXL_37484, GeneID: 296709, Rattus norvegicus chr. 3<br>ribosomal protein L35	EGRF-SP1F	339 - 316	(-)
	EGRF-SP1F	345 - 321	(-)
**GXP_45351** [GXP_45351] (1 - 828)<br>**Cacna1b**, GXL_37498, GeneID: 257648, Rattus norvegicus chr. 3<br>calcium channel, voltage-dependent, N type, alpha 1B subunit	EGRF-SP1F	512 - 488	(-)
**GXP_45396** [GXP_45396] (1 - 1018)<br>**Stk39**, GXL_37542, GeneID: 54348, Rattus norvegicus chr. 3<br>serine/threonine kinase 39, STE20/SPS1 homolog (yeast)	EGRF-SP1F	624 - 604	(-)
	EGRF-SP1F	720 - 743	(+)
**GXP_1413101** [GXP_1413101] (1 - 602)<br>**Stxbp1**, GXL_37543, GeneID: 25558, Rattus norvegicus chr. 3<br>syntaxin binding protein 1	EGRF-SP1F	26 - 46	(+)
	EGRF-SP1F	28 - 51	(+)
**GXP_45417** [GXP_45417] (1 - 715)<br>**Calcrl**, GXL_37563, GeneID: 25029, Rattus norvegicus chr. 3<br>calcitonin receptor-like	EGRF-SP1F	155 - 179	(+)
**GXP_45424** [GXP_45424] (1 - 656)<br>**Serping1**, GXL_37570, GeneID: 295703, Rattus norvegicus chr. 3<br>serine (or cysteine) peptidase inhibitor, clade G, member 1	EGRF-SP1F	179 - 200	(+)
	EGRF-SP1F	183 - 206	(+)
**GXP_45441** [GXP_45441] (1 - 770)<br>**Pde11a**, GXL_37587, GeneID: 140928, Rattus norvegicus chr. 3<br>phosphodiesterase 11A	EGRF-SP1F	155 - 133	(-)
**GXP_1413385** [GXP_1413385] (1 - 602)<br>**Pde11a**, GXL_37587, GeneID: 140928, Rattus norvegicus chr. 3<br>phosphodiesterase 11A	EGRF-SP1F	105 - 85	(-)
**GXP_45447** [GXP_45447] (1 - 710)<br>**Atf2**, GXL_37593, GeneID: 81647, Rattus norvegicus chr. 3<br>activating transcription factor 2	EGRF-SP1F	354 - 374	(+)
**GXP_45479** [GXP_45479] (1 - 620)<br>**Rtn4rl2**, GXL_37625, GeneID: 311169, Rattus norvegicus chr. 3<br>reticulon 4 receptor-like 2	EGRF-SP1F	307 - 287	(-)
	EGRF-SP1F	476 - 499	(+)
	EGRF-SP1F	484 - 505	(+)

**GXP_45489** [GXP_45489] (1 - 841)<br>**Chn1**, GXL_37635, GeneID: 84030, Rattus norvegicus chr. 3<br>chimerin (chimaerin) 1	EGRF-SP1F	544 - 523	(-)
**GXP_45503** [GXP_45503] (1 - 601)<br>**Cd44**, GXL_37649, GeneID: 25406, Rattus norvegicus chr. 3<br>CD44 antigen	EGRF-SP1F	298 - 278	(-)
**GXP_1412908** [GXP_1412908] (1 - 602)<br>**Slc34a3**, GXL_37660, GeneID: 246234, Rattus norvegicus chr. 3<br>solute carrier family 34 (sodium phosphate), member 3	EGRF-SP1F	354 - 334	(-)
**GXP_859235** [GXP_859235] (1 - 603)<br>**Acvr1**, GXL_37664, GeneID: 79558, Rattus norvegicus chr. 3<br>activin A receptor, type 1	EGRF-SP1F	144 - 168	(+)
	EGRF-SP1F	256 - 276	(+)
	EGRF-SP1F	337 - 357	(+)
	EGRF-SP1F	339 - 362	(+)
	EGRF-SP1F	372 - 393	(+)
	EGRF-SP1F	436 - 458	(+)
	EGRF-SP1F	441 - 463	(+)
	EGRF-SP1F	454 - 477	(+)
**GXP_1413236** [GXP_1413236] (1 - 601)<br>**Acvr1**, GXL_37664, GeneID: 79558, Rattus norvegicus chr. 3<br>activin A receptor, type 1	EGRF-SP1F	66 - 86	(+)
	EGRF-SP1F	147 - 167	(+)
	EGRF-SP1F	149 - 172	(+)
	EGRF-SP1F	182 - 203	(+)
	EGRF-SP1F	246 - 268	(+)
	EGRF-SP1F	251 - 273	(+)
	EGRF-SP1F	264 - 287	(+)
**GXP_45522** [GXP_45522] (1 - 601)<br>**Rbms1**, GXL_37668, GeneID: 362138, Rattus norvegicus chr. 3<br>RNA binding motif, single stranded interacting protein 1	EGRF-SP1F	198 - 218	(+)
**GXP_1412907** [GXP_1412907] (1 - 602)<br>**Tubb2c**, GXL_37681, GeneID: 296554, Rattus norvegicus chr. 3<br>tubulin, beta 2c	EGRF-SP1F	352 - 332	(-)
**GXP_45537** [GXP_45537] (1 - 601)<br>**Olr678_predicted**, GXL_37683, GeneID: 295884, Rattus norvegicus chr. 3<br>olfactory receptor 678 (predicted)	EGRF-SP1F	86 - 109	(+)
**GXP_45562** [GXP_45562] (1 - 702)<br>**Dgkz**, GXL_37708, GeneID: 81821, Rattus norvegicus chr. 3<br>diacylglycerol kinase zeta	EGRF-SP1F	307 - 287	(-)
**GXP_1162920** [GXP_1162920] (1 - 601)<br>**Phpt1_predicted**, GXL_37713, GeneID: 296571, Rattus norvegicus chr. 3	EGRF-SP1F	396 - 373	(-)

phosphohistidine phosphatase 1 (predicted)			
**GXP_45583** [GXP_45583] (1 - 813)<br>**Sh3glb2**, GXL_37729, GeneID: 311848, Rattus norvegicus chr. 3<br>SH3-domain GRB2-like endophilin B2	EGRF-SP1F	661 - 638	(-)
**GXP_45608** [GXP_45608] (1 - 956)<br>**Dlx2**, GXL_37754, GeneID: 296499, Rattus norvegicus chr. 3<br>distal-less homeobox 2	EGRF-SP1F	730 - 753	(+)
**GXP_52111** [GXP_52111] (1 - 601)<br>**Olr917_predicted**, GXL_42851, GeneID: 288795, Rattus norvegicus chr. 7<br>olfactory receptor 917 (predicted)	EGRF-SP1F	46 - 26	(-)
**GXP_52136** [GXP_52136] (1 - 627)<br>**Akap8**, GXL_42875, GeneID: 116633, Rattus norvegicus chr. 7<br>A kinase (PRKA) anchor protein 8	EGRF-SP1F	152 - 131	(-)
**GXP_52137** [GXP_52137] (1 - 603)<br>**Aes**, GXL_42876, GeneID: 29466, Rattus norvegicus chr. 7<br>amino-terminal enhancer of split	EGRF-SP1F	472 - 452	(-)
**GXP_1417784** [GXP_1417784] (1 - 1021)<br>**Aes**, GXL_42876, GeneID: 29466, Rattus norvegicus chr. 7<br>amino-terminal enhancer of split	EGRF-SP1F<br>EGRF-SP1F	70 - 49<br>1021 - 997	(-)<br>(-)
**GXP_52151** [GXP_52151] (1 - 917)<br>**Tmem19**, GXL_42890, GeneID: 299800, Rattus norvegicus chr. 7<br>transmembrane protein 19	EGRF-SP1F	511 - 489	(-)
**GXP_52154** [GXP_52154] (1 - 682)<br>**Hrsp12**, GXL_42893, GeneID: 65151, Rattus norvegicus chr. 7<br>heat-responsive protein 12	EGRF-SP1F	23 - 43	(+)
**GXP_52157** [GXP_52157] (1 - 896)<br>**Map2k2**, GXL_42896, GeneID: 58960, Rattus norvegicus chr. 7<br>mitogen activated protein kinase kinase 2	EGRF-SP1F	535 - 514	(-)
**GXP_52161** [GXP_52161] (1 - 919)<br>**Zbtb7a**, GXL_42900, GeneID: 117107, Rattus norvegicus chr. 7<br>zinc finger and BTB domain containing 7a	EGRF-SP1F<br>EGRF-SP1F<br>EGRF-SP1F	237 - 257<br>544 - 566<br>808 - 830	(+)<br>(+)<br>(+)
**GXP_52165** [GXP_52165] (1 - 695)<br>**Vps28_predicted**, GXL_42903, GeneID: 300052, Rattus norvegicus chr. 7<br>vacuolar protein sorting 28 (yeast) (predicted)	EGRF-SP1F	435 - 415	(-)
**GXP_52172** [GXP_52172] (1 - 602)<br>**Matk**, GXL_42910, GeneID: 60450, Rattus norvegicus chr. 7<br>megakaryocyte-associated tyrosine kinase	EGRF-SP1F	468 - 488	(+)
**GXP_1417808** [GXP_1417808] (1 - 601)<br>**Matk**, GXL_42910, GeneID: 60450, Rattus norvegicus chr. 7<br>megakaryocyte-associated tyrosine kinase	EGRF-SP1F	159 - 179	(+)

**GXP_52173** [GXP_52173] (1 - 633)<br>**Lynx1_predicted**, GXL_42911, GeneID: 300018, Rattus norvegicus chr. 7<br>Ly6/neurotoxin 1 (predicted)	EGRF-SP1F	488 - 466	(-)
**GXP_52177** [GXP_52177] (1 - 601)<br>**Ptbp1**, GXL_42914, GeneID: 29497, Rattus norvegicus chr. 7<br>polypyrimidine tract binding protein 1	EGRF-SP1F	218 - 240	(+)
**GXP_52180** [GXP_52180] (1 - 1010)<br>**Cand1**, GXL_42917, GeneID: 117152, Rattus norvegicus chr. 7<br>cullin associated and neddylation disassociated 1	EGRF-SP1F	603 - 583	(-)
**GXP_52189** [GXP_52189] (1 - 1084)<br>**Cart1**, GXL_42926, GeneID: 25401, Rattus norvegicus chr. 7<br>cartilage homeo protein 1	EGRF-SP1F	409 - 389	(-)
	EGRF-SP1F	679 - 656	(-)
	EGRF-SP1F	683 - 661	(-)
	EGRF-SP1F	876 - 896	(+)
**GXP_52203** [GXP_52203] (1 - 804)<br>**Plec1**, GXL_42940, GeneID: 64204, Rattus norvegicus chr. 7<br>plectin 1	EGRF-SP1F	15 - 35	(+)
**GXP_1170757** [GXP_1170757] (1 - 601)<br>**Plec1**, GXL_42940, GeneID: 64204, Rattus norvegicus chr. 7<br>plectin 1	EGRF-SP1F	128 - 108	(-)
	EGRF-SP1F	131 - 110	(-)
	EGRF-SP1F	550 - 528	(-)
**GXP_1418410** [GXP_1418410] (1 - 605)<br>**Plec1**, GXL_42940, GeneID: 64204, Rattus norvegicus chr. 7<br>plectin 1	EGRF-SP1F	434 - 454	(+)
**GXP_1418412** [GXP_1418412] (1 - 662)<br>**Plec1**, GXL_42940, GeneID: 64204, Rattus norvegicus chr. 7<br>plectin 1	EGRF-SP1F	315 - 295	(-)
**GXP_1418416** [GXP_1418416] (1 - 657)<br>**Plec1**, GXL_42940, GeneID: 64204, Rattus norvegicus chr. 7<br>plectin 1	EGRF-SP1F	454 - 432	(-)
	EGRF-SP1F	459 - 437	(-)
	EGRF-SP1F	465 - 442	(-)
	EGRF-SP1F	469 - 447	(-)
**GXP_1418418** [GXP_1418418] (1 - 602)<br>**Plec1**, GXL_42940, GeneID: 64204, Rattus norvegicus chr. 7<br>plectin 1	EGRF-SP1F	174 - 154	(-)
	EGRF-SP1F	177 - 156	(-)
	EGRF-SP1F	596 - 574	(-)
**GXP_52220** [GXP_52220] (1 - 666)<br>**Tra1_predicted**, GXL_42957, GeneID: 362862, Rattus norvegicus chr. 7<br>tumor rejection antigen gp96 (predicted)	EGRF-SP1F	616 - 593	(-)
**GXP_1170421** [GXP_1170421] (1 - 1063)<br>**Geft**, GXL_42965, GeneID: 314904, Rattus norvegicus chr. 7<br>RAC/CDC42 exchange factor	EGRF-SP1F	198 - 178	(-)
	EGRF-SP1F	937 - 915	(-)

**GXP_1418207** [GXP_1418207] (1 - 601)<br>**Geft**, GXL_42965, GeneID: 314904, Rattus norvegicus chr. 7<br>RAC/CDC42 exchange factor	EGRF-SP1F<br>EGRF-SP1F	51 - 27<br>565 - 588	(-)<br>(+)
**GXP_52230** [GXP_52230] (1 - 601)<br>**Usp15**, GXL_42967, GeneID: 171329, Rattus norvegicus chr. 7<br>ubiquitin specific peptidase 15	EGRF-SP1F<br>EGRF-SP1F	394 - 418<br>402 - 422	(+)<br>(+)
**GXP_52233** [GXP_52233] (1 - 724)<br>**Shmt2**, GXL_42970, GeneID: 299857, Rattus norvegicus chr. 7<br>serine hydroxymethyltransferase 2 (mitochondrial)	EGRF-SP1F	413 - 392	(-)
**GXP_52246** [GXP_52246] (1 - 665)<br>**Rpl30**, GXL_42983, GeneID: 64640, Rattus norvegicus chr. 7<br>ribosomal protein L30	EGRF-SP1F	137 - 115	(-)
**GXP_52262** [GXP_52262] (1 - 948)<br>**Hcn2**, GXL_42999, GeneID: 114244, Rattus norvegicus chr. 7<br>hyperpolarization activated cyclic nucleotide-gated potassium channel 2	EGRF-SP1F	877 - 898	(+)
**GXP_52275** [GXP_52275] (1 - 962)<br>**Ywhaz**, GXL_43012, GeneID: 25578, Rattus norvegicus chr. 7<br>tyrosine 3-monooxygenase/tryptophan 5-monooxygenase activation protein, zeta polypeptide	EGRF-SP1F<br>EGRF-SP1F<br>EGRF-SP1F	269 - 292<br>522 - 501<br>526 - 506	(+)<br>(-)<br>(-)
**GXP_1418271** [GXP_1418271] (1 - 604)<br>**Ywhaz**, GXL_43012, GeneID: 25578, Rattus norvegicus chr. 7<br>tyrosine 3-monooxygenase/tryptophan 5-monooxygenase activation protein, zeta polypeptide	EGRF-SP1F	393 - 373	(-)
**GXP_1418272** [GXP_1418272] (1 - 602)<br>**Ywhaz**, GXL_43012, GeneID: 25578, Rattus norvegicus chr. 7<br>tyrosine 3-monooxygenase/tryptophan 5-monooxygenase activation protein, zeta polypeptide	EGRF-SP1F	376 - 397	(+)
**GXP_1418273** [GXP_1418273] (1 - 602)<br>**Ywhaz**, GXL_43012, GeneID: 25578, Rattus norvegicus chr. 7<br>tyrosine 3-monooxygenase/tryptophan 5-monooxygenase activation protein, zeta polypeptide	EGRF-SP1F	464 - 485	(+)
**GXP_52298** [GXP_52298] (1 - 1171)<br>**Tmpo**, GXL_43035, GeneID: 25359, Rattus norvegicus chr. 7<br>thymopoietin	EGRF-SP1F<br>EGRF-SP1F<br>EGRF-SP1F	411 - 431<br>419 - 443<br>582 - 605	(+)<br>(+)<br>(+)
**GXP_52312** [GXP_52312] (1 - 649)<br>**Fstl3**, GXL_43049, GeneID: 114031, Rattus norvegicus chr. 7<br>follistatin-like 3	EGRF-SP1F	448 - 470	(+)
**GXP_519485** [GXP_519485] (1 - 601)<br>**Suox**, GXL_43054, GeneID: 81805, Rattus norvegicus chr. 7<br>sulfite oxidase	EGRF-SP1F	468 - 490	(+)
**GXP_52330** [GXP_52330] (1 - 689)<br>**Notch3**, GXL_43067, GeneID: 56761, Rattus norvegicus chr. 7<br>Notch gene homolog 3 (Drosophila)	EGRF-SP1F	639 - 660	(+)

**GXP_52346** [GXP_52346] (1 - 821)<br>**Dgat1**, GXL_43083, GeneID: 84497, Rattus norvegicus chr. 7<br>diacylglycerol O-acyltransferase 1	EGRF-SP1F	650 - 626	(-)
**GXP_52355** [GXP_52355] (1 - 601)<br>**Tdg**, GXL_43092, GeneID: 114521, Rattus norvegicus chr. 7<br>thymine-DNA glycosylase	EGRF-SP1F	254 - 274	(+)
**GXP_52364** [GXP_52364] (1 - 601)<br>**Siahbp1**, GXL_43101, GeneID: 84401, Rattus norvegicus chr. 7<br>siah binding protein 1, FBP interacting repressor, pyrimidine tract binding splicing factor, Ro ribonucleoprotein-binding protein 1	EGRF-SP1F	322 - 342	(+)
**GXP_52366** [GXP_52366] (1 - 853)<br>**Naprt1**, GXL_43103, GeneID: 315085, Rattus norvegicus chr. 7<br>nicotinate phosphoribosyltransferase domain containing 1	EGRF-SP1F	710 - 689	(-)
**GXP_863846** [GXP_863846] (1 - 601)<br>**Naprt1**, GXL_43103, GeneID: 315085, Rattus norvegicus chr. 7<br>nicotinate phosphoribosyltransferase domain containing 1	EGRF-SP1F	137 - 157	(+)
**GXP_52398** [GXP_52398] (1 - 677)<br>**Vezt**, GXL_43135, GeneID: 299738, Rattus norvegicus chr. 7<br>vezatin, adherens junctions transmembrane protein	EGRF-SP1F	392 - 414	(+)
**GXP_52405** [GXP_52405] (1 - 756)<br>**Nxph4**, GXL_43142, GeneID: 59316, Rattus norvegicus chr. 7<br>neurexophilin 4	EGRF-SP1F<br>EGRF-SP1F<br>EGRF-SP1F	425 - 405<br>458 - 481<br>501 - 524	(-)<br>(+)<br>(+)
**GXP_52448** [GXP_52448] (1 - 756)<br>**Glipr1**, GXL_43185, GeneID: 299783, Rattus norvegicus chr. 7<br>GLI pathogenesis-related 1 (glioma)	EGRF-SP1F<br>EGRF-SP1F<br>EGRF-SP1F	368 - 345<br>374 - 352<br>382 - 358	(-)<br>(-)<br>(-)
**GXP_52461** [GXP_52461] (1 - 694)<br>**Fbxo7**, GXL_43198, GeneID: 366854, Rattus norvegicus chr. 7<br>F-box only protein 7	EGRF-SP1F	359 - 336	(-)
**GXP_1170056** [GXP_1170056] (1 - 609)<br>**Fbxo7**, GXL_43198, GeneID: 366854, Rattus norvegicus chr. 7<br>F-box only protein 7	EGRF-SP1F	101 - 78	(-)
**GXP_1418136** [GXP_1418136] (1 - 656)<br>**Yeats4_predicted**, GXL_43224, GeneID: 299810, Rattus norvegicus chr. 7<br>YEATS domain containing 4 (predicted)	EGRF-SP1F	541 - 517	(-)
**GXP_52490** [GXP_52490] (1 - 1079)<br>**Kcnq3**, GXL_43227, GeneID: 29682, Rattus norvegicus chr. 7<br>potassium voltage-gated channel, subfamily Q, member 3	EGRF-SP1F<br>EGRF-SP1F<br>EGRF-SP1F<br>EGRF-SP1F	505 - 481<br>797 - 777<br>1014 - 993<br>1051 - 1030	(-)<br>(-)<br>(-)<br>(-)
**GXP_1418044** [GXP_1418044] (1 - 686)<br>**Socs2**, GXL_43253, GeneID: 84607, Rattus norvegicus chr. 7	EGRF-SP1F	466 - 446	(-)

suppressor of cytokine signaling 2			
**GXP_52556** [GXP_52556] (1 - 844)	EGRF-SP1F	776 - 753	(-)
**Rab21**, GXL_43293, GeneID: 299799, Rattus norvegicus chr. 7			
RAB21, member RAS oncogene family	EGRF-SP1F	779 - 758	(-)
**GXP_52578** [GXP_52578] (1 - 614)	EGRF-SP1F	365 - 344	(-)
**Ascl1**, GXL_43315, GeneID: 64186, Rattus norvegicus chr. 7	EGRF-SP1F	402 - 382	(-)
achaete-scute complex homolog-like 1 (Drosophila)	EGRF-SP1F	437 - 417	(-)
**GXP_52583** [GXP_52583] (1 - 834)			
**Has2**, GXL_43320, GeneID: 25694, Rattus norvegicus chr. 7	EGRF-SP1F	445 - 465	(+)
hyaluronan synthase 2			
**GXP_1417835** [GXP_1417835] (1 - 602)			
**Csnk1g2**, GXL_43324, GeneID: 65278, Rattus norvegicus chr. 7	EGRF-SP1F	383 - 360	(-)
casein kinase 1, gamma 2			
**GXP_1418130** [GXP_1418130] (1 - 1321)	EGRF-SP1F	1167 - 1144	(-)
**Cnot2**, GXL_43336, GeneID: 299805, Rattus norvegicus chr. 7	EGRF-SP1F	1171 - 1149	(-)
CCR4-NOT transcription complex, subunit 2	EGRF-SP1F	1174 - 1154	(-)
**GXP_52602** [GXP_52602] (1 - 696)			
**Cyp4f6**, GXL_43339, GeneID: 266689, Rattus norvegicus chr. 7	EGRF-SP1F	282 - 262	(-)
cytochrome P450 4F6			
**GXP_59022** [GXP_59022] (1 - 1224)	EGRF-SP1F	109 - 130	(+)
**Gpr157**, GXL_48343, GeneID: 313725, Rattus norvegicus chr. 5			
G protein-coupled receptor 157	EGRF-SP1F	180 - 201	(+)
**GXP_59024** [GXP_59024] (1 - 601)	EGRF-SP1F	307 - 331	(+)
**Capzb**, GXL_48345, GeneID: 298584, Rattus norvegicus chr. 5			
capping protein (actin filament) muscle Z-line, beta	EGRF-SP1F	473 - 451	(-)
**GXP_59036** [GXP_59036] (1 - 980)			
**Id3**, GXL_48357, GeneID: 25585, Rattus norvegicus chr. 5	EGRF-SP1F	782 - 760	(-)
inhibitor of DNA binding 3			
**GXP_1416790** [GXP_1416790] (1 - 736)			
**Plekhg5**, GXL_48371, GeneID: 310999, Rattus norvegicus chr. 5	EGRF-SP1F	426 - 402	(-)
pleckstrin homology domain containing, family G (with RhoGef domain) member 5			
**GXP_59056** [GXP_59056] (1 - 915)	EGRF-SP1F	279 - 299	(+)
**Heyl_predicted**, GXL_48377, GeneID: 313575, Rattus norvegicus chr. 5			
hairy/enhancer-of-split related with YRPW motif-like (predicted)	EGRF-SP1F	731 - 710	(-)
**GXP_59059** [GXP_59059] (1 - 601)	EGRF-SP1F	31 - 53	(+)
**Pou3f1**, GXL_48380, GeneID: 192110, Rattus norvegicus chr. 5			
POU domain, class 3, transcription factor 1	EGRF-SP1F	173 - 196	(+)
**GXP_1168288** [GXP_1168288] (1 - 869)	EGRF-SP1F	459 - 435	(-)
**Mad2l2**, GXL_48384, GeneID: 313702, Rattus norvegicus chr. 5			

MAD2 mitotic arrest deficient-like 2 (yeast)			
**GXP_59064** [GXP_59064] (1 - 644)<br>**Lyk4**, GXL_48385, GeneID: 360230, Rattus norvegicus chr. 5<br>Ser/Thr-like protein kinase lyk4	EGRF-SP1F<br>EGRF-SP1F	583 - 603<br>591 - 615	(+)<br>(+)
**GXP_1416774** [GXP_1416774] (1 - 648)<br>**Slc2a5**, GXL_48397, GeneID: 65197, Rattus norvegicus chr. 5<br>solute carrier family 2, member 5	EGRF-SP1F	44 - 22	(-)
**GXP_1416312** [GXP_1416312] (1 - 601)<br>**Ebna1bp2**, GXL_48417, GeneID: 114021, Rattus norvegicus chr. 5<br>EBNA1 binding protein 2	EGRF-SP1F<br>EGRF-SP1F	90 - 113<br>96 - 119	(+)<br>(+)
**GXP_1416316** [GXP_1416316] (1 - 985)<br>**Ebna1bp2**, GXL_48417, GeneID: 114021, Rattus norvegicus chr. 5<br>EBNA1 binding protein 2	EGRF-SP1F	215 - 195	(-)
**GXP_1416557** [GXP_1416557] (1 - 601)<br>**Ubxd5**, GXL_48432, GeneID: 192207, Rattus norvegicus chr. 5<br>UBX domain containing 5	EGRF-SP1F	165 - 189	(+)
**GXP_1416351** [GXP_1416351] (1 - 870)<br>**Rims3**, GXL_48441, GeneID: 65025, Rattus norvegicus chr. 5<br>regulating synaptic membrane exocytosis 3	EGRF-SP1F	642 - 663	(+)
**GXP_59155** [GXP_59155] (1 - 650)<br>**Hpcal4**, GXL_48474, GeneID: 50872, Rattus norvegicus chr. 5<br>hippocalcin-like 4	EGRF-SP1F<br>EGRF-SP1F	106 - 84<br>109 - 88	(-)<br>(-)
**GXP_1416363** [GXP_1416363] (1 - 903)<br>**Hpcal4**, GXL_48474, GeneID: 50872, Rattus norvegicus chr. 5<br>hippocalcin-like 4	EGRF-SP1F<br>EGRF-SP1F<br>EGRF-SP1F	269 - 249<br>635 - 613<br>638 - 617	(-)<br>(-)<br>(-)
**GXP_59157** [GXP_59157] (1 - 859)<br>**Wnt4**, GXL_48476, GeneID: 84426, Rattus norvegicus chr. 5<br>wingless-related MMTV integration site 4	EGRF-SP1F	262 - 241	(-)
**GXP_1416567** [GXP_1416567] (1 - 616)<br>**Pafah2**, GXL_48480, GeneID: 313611, Rattus norvegicus chr. 5<br>platelet-activating factor acetylhydrolase 2	EGRF-SP1F	118 - 139	(+)
**GXP_59171** [GXP_59171] (1 - 760)<br>**Srm**, GXL_48490, GeneID: 84596, Rattus norvegicus chr. 5<br>spermidine synthase	EGRF-SP1F<br>EGRF-SP1F	293 - 316<br>586 - 606	(+)<br>(+)
**GXP_59178** [GXP_59178] (1 - 679)<br>**Nt5c1a_predicted**, GXL_48497, GeneID: 313574, Rattus norvegicus chr. 5<br>5'-nucleotidase, cytosolic IA (predicted)	EGRF-SP1F	441 - 420	(-)
**GXP_59180** [GXP_59180] (1 - 642)<br>**Nppb**, GXL_48499, GeneID: 25105, Rattus norvegicus chr. 5<br>natriuretic peptide precursor type B	EGRF-SP1F	277 - 299	(+)

**GXP_1416448** [GXP_1416448] (1 - 603)<br>**Dlgap3**, GXL_48527, GeneID: 286923, Rattus norvegicus chr. 5<br>discs, large (Drosophila) homolog-associated protein 3	EGRF-SP1F	417 - 437	(+)
**GXP_59209** [GXP_59209] (1 - 967)<br>**Nppa**, GXL_48528, GeneID: 24602, Rattus norvegicus chr. 5<br>natriuretic peptide precursor type A	EGRF-SP1F	357 - 377	(+)
	EGRF-SP1F	615 - 595	(-)
**GXP_59216** [GXP_59216] (1 - 813)<br>**Casp9**, GXL_48535, GeneID: 58918, Rattus norvegicus chr. 5<br>caspase 9	EGRF-SP1F	653 - 630	(-)
	EGRF-SP1F	655 - 635	(-)
**GXP_59218** [GXP_59218] (1 - 733)<br>**Grik3**, GXL_48537, GeneID: 298521, Rattus norvegicus chr. 5<br>glutamate receptor, ionotropic, kainate 3	EGRF-SP1F	197 - 176	(-)
**GXP_59251** [GXP_59251] (1 - 620)<br>**Mfap2_predicted**, GXL_48570, GeneID: 313662, Rattus norvegicus chr. 5<br>microfibrillar-associated protein 2 (predicted)	EGRF-SP1F	487 - 465	(-)
**GXP_59253** [GXP_59253] (1 - 703)<br>**Padi2**, GXL_48572, GeneID: 29511, Rattus norvegicus chr. 5<br>peptidyl arginine deiminase, type II	EGRF-SP1F	458 - 478	(+)
	EGRF-SP1F	460 - 483	(+)
**GXP_59270** [GXP_59270] (1 - 1231)<br>**Usp48**, GXL_48589, GeneID: 362636, Rattus norvegicus chr. 5<br>ubiquitin specific protease 48	EGRF-SP1F	982 - 960	(-)
**GXP_1168070** [GXP_1168070] (1 - 1000)<br>**Gale**, GXL_48593, GeneID: 114860, Rattus norvegicus chr. 5<br>galactose-4-epimerase, UDP	EGRF-SP1F	101 - 81	(-)
	EGRF-SP1F	501 - 478	(-)
**GXP_59279** [GXP_59279] (1 - 687)<br>**Acot7**, GXL_48598, GeneID: 26759, Rattus norvegicus chr. 5<br>acyl-CoA thioesterase 7	EGRF-SP1F	219 - 243	(+)
	EGRF-SP1F	633 - 654	(+)
**GXP_1150373** [GXP_1150373] (1 - 601)<br>**Acads**, GXL_50825, GeneID: 64304, Rattus norvegicus chr. 12<br>acetyl-Coenzyme A dehydrogenase, short chain	EGRF-SP1F	506 - 526	(+)
**GXP_61679** [GXP_61679] (1 - 1089)<br>**Rnf10**, GXL_50826, GeneID: 288710, Rattus norvegicus chr. 12<br>ring finger protein 10	EGRF-SP1F	690 - 713	(+)
**GXP_61684** [GXP_61684] (1 - 631)<br>**Abcb9**, GXL_50831, GeneID: 63886, Rattus norvegicus chr. 12<br>ATP-binding cassette, sub-family B (MDR/TAP), member 9	EGRF-SP1F	475 - 499	(+)
**GXP_1404556** [GXP_1404556] (1 - 1168)<br>**Atp2a2**, GXL_50840, GeneID: 29693, Rattus norvegicus chr. 12<br>ATPase, Ca++ transporting, cardiac muscle, slow twitch 2	EGRF-SP1F	824 - 801	(-)
	EGRF-SP1F	413 - 435	(+)

**GXP_61694** [GXP_61694] (1 - 754)<br>**Pxmp2**, GXL_50841, GeneID: 29533, Rattus norvegicus chr. 12<br>peroxisomal membrane protein 2			
**GXP_61722** [GXP_61722] (1 - 601)<br>**Dynll1**, GXL_50869, GeneID: 58945, Rattus norvegicus chr. 12<br>dynein light chain LC8-type 1	EGRF-SP1F	280 - 260	(-)
**GXP_61739** [GXP_61739] (1 - 827)<br>**Gtpbp6_predicted**, GXL_50886, GeneID: 363931, Rattus norvegicus chr. 12<br>GTP binding protein 6 (putative) (predicted)	EGRF-SP1F	88 - 68	(-)
**GXP_61745** [GXP_61745] (1 - 1042)<br>**Tcf1**, GXL_50892, GeneID: 24817, Rattus norvegicus chr. 12<br>transcription factor 1	EGRF-SP1F	296 - 317	(+)
	EGRF-SP1F	298 - 319	(+)
	EGRF-SP1F	300 - 321	(+)
	EGRF-SP1F	302 - 323	(+)
	EGRF-SP1F	560 - 582	(+)
**GXP_61747** [GXP_61747] (1 - 784)<br>**Erp29**, GXL_50894, GeneID: 117030, Rattus norvegicus chr. 12<br>endoplasmic reticulum protein 29	EGRF-SP1F	419 - 443	(+)
**GXP_61754** [GXP_61754] (1 - 647)<br>**Diablo**, GXL_50901, GeneID: 288753, Rattus norvegicus chr. 12<br>diablo homolog (Drosophila)	EGRF-SP1F	169 - 191	(+)
	EGRF-SP1F	174 - 196	(+)
	EGRF-SP1F	179 - 201	(+)
	EGRF-SP1F	244 - 264	(+)
**GXP_61755** [GXP_61755] (1 - 707)<br>**Ptpn11**, GXL_50902, GeneID: 25622, Rattus norvegicus chr. 12<br>protein tyrosine phosphatase, non-receptor type 11	EGRF-SP1F	536 - 516	(-)
**GXP_61759** [GXP_61759] (1 - 623)<br>**Eif2b1**, GXL_50906, GeneID: 64514, Rattus norvegicus chr. 12<br>eukaryotic translation initiation factor 2B, subunit 1 alpha	EGRF-SP1F	326 - 346	(+)
**GXP_61763** [GXP_61763] (1 - 635)<br>**Trpv4**, GXL_50910, GeneID: 66026, Rattus norvegicus chr. 12<br>transient receptor potential cation channel, subfamily V, member 4	EGRF-SP1F	490 - 466	(-)
**GXP_1404540** [GXP_1404540] (1 - 682)<br>**Vps33a**, GXL_50912, GeneID: 65081, Rattus norvegicus chr. 12<br>vacuolar protein sorting 33A (yeast)	EGRF-SP1F	439 - 415	(-)
**GXP_1404541** [GXP_1404541] (1 - 602)<br>**Vps33a**, GXL_50912, GeneID: 65081, Rattus norvegicus chr. 12<br>vacuolar protein sorting 33A (yeast)	EGRF-SP1F	561 - 540	(-)
**GXP_61767** [GXP_61767] (1 - 601)<br>**Lnk**, GXL_50914, GeneID: 58838, Rattus norvegicus chr. 12<br>linker of T-cell receptor pathways	EGRF-SP1F	265 - 286	(+)

**GXP_508171** [GXP_508171] (1 - 703)<br>**Mbp**, GXL_52005, GeneID: 24547, Rattus norvegicus chr. 18<br>myelin basic protein	EGRF-SP1F	568 - 545	(-)
**GXP_1404014** [GXP_1404014] (1 - 602)<br>**Fgf12**, GXL_60581, GeneID: 170630, Rattus norvegicus chr. 11<br>fibroblast growth factor 12	EGRF-SP1F	287 - 265	(-)
**GXP_1404015** [GXP_1404015] (1 - 756)<br>**Fgf12**, GXL_60581, GeneID: 170630, Rattus norvegicus chr. 11<br>fibroblast growth factor 12	EGRF-SP1F	107 - 130	(+)
	EGRF-SP1F	114 - 137	(+)
	EGRF-SP1F	221 - 242	(+)
**GXP_72925** [GXP_72925] (1 - 608)<br>**Scarf2_predicted**, GXL_60614, GeneID: 287949, Rattus norvegicus chr. 11<br>scavenger receptor class F, member 2 (predicted)	EGRF-SP1F	223 - 244	(+)
	EGRF-SP1F	225 - 246	(+)
	EGRF-SP1F	499 - 523	(+)
	EGRF-SP1F	570 - 594	(+)
**GXP_72937** [GXP_72937] (1 - 601)<br>**Dscr1**, GXL_60625, GeneID: 266766, Rattus norvegicus chr. 11<br>Down syndrome critical region homolog 1 (human)	EGRF-SP1F	398 - 419	(+)
**GXP_1149481** [GXP_1149481] (1 - 601)<br>**Txnrd2**, GXL_60673, GeneID: 50551, Rattus norvegicus chr. 11<br>thioredoxin reductase 2	EGRF-SP1F	53 - 29	(-)
**GXP_72992** [GXP_72992] (1 - 797)<br>**P2rxl1**, GXL_60679, GeneID: 25041, Rattus norvegicus chr. 11<br>purinergic receptor P2X-like 1, orphan receptor	EGRF-SP1F	158 - 138	(-)
	EGRF-SP1F	549 - 529	(-)
**GXP_72995** [GXP_72995] (1 - 728)<br>**Ttc3_predicted**, GXL_60682, GeneID: 360702, Rattus norvegicus chr. 11<br>tetratricopeptide repeat domain 3 (predicted)	EGRF-SP1F	484 - 464	(-)
**GXP_73016** [GXP_73016] (1 - 601)<br>**Alcam**, GXL_60703, GeneID: 79559, Rattus norvegicus chr. 11<br>activated leukocyte cell adhesion molecule	EGRF-SP1F	89 - 69	(-)
	EGRF-SP1F	492 - 472	(-)
**GXP_73018** [GXP_73018] (1 - 601)<br>**Lrrc15**, GXL_60705, GeneID: 246296, Rattus norvegicus chr. 11<br>leucine rich repeat containing 15	EGRF-SP1F	129 - 108	(-)
	EGRF-SP1F	131 - 110	(-)
	EGRF-SP1F	133 - 112	(-)
	EGRF-SP1F	135 - 114	(-)
**GXP_73033** [GXP_73033] (1 - 676)<br>**Bdh1**, GXL_60720, GeneID: 117099, Rattus norvegicus chr. 11<br>3-hydroxybutyrate dehydrogenase, type 1	EGRF-SP1F	474 - 496	(+)
**GXP_73036** [GXP_73036] (1 - 1188)<br>**Masp1**, GXL_60723, GeneID: 64023, Rattus norvegicus chr. 11<br>mannan-binding lectin serine peptidase 1	EGRF-SP1F	949 - 929	(-)

**GXP_1403943** [GXP_1403943] (1 - 601)<br>**Slc12a8**, GXL_60751, GeneID: 266733, Rattus norvegicus chr. 11<br>solute carrier family 12 (potassium/chloride transporters), member 8	EGRF-SP1F	478 - 498	(+)
**GXP_1403856** [GXP_1403856] (1 - 602)<br>**Qtrtd1_predicted**, GXL_60761, GeneID: 288364, Rattus norvegicus chr. 11<br>queuine tRNA-ribosyltransferase domain containing 1 (predicted)	EGRF-SP1F	300 - 320	(+)
**GXP_1403858** [GXP_1403858] (1 - 601)<br>**Qtrtd1_predicted**, GXL_60761, GeneID: 288364, Rattus norvegicus chr. 11<br>queuine tRNA-ribosyltransferase domain containing 1 (predicted)	EGRF-SP1F	114 - 135	(+)
**GXP_1403741** [GXP_1403741] (1 - 601)<br>**Erg**, GXL_60785, GeneID: 170909, Rattus norvegicus chr. 11<br>v-ets erythroblastosis virus E26 oncogene like (avian)	EGRF-SP1F	411 - 390	(-)
	EGRF-SP1F	421 - 399	(-)
**GXP_73124** [GXP_73124] (1 - 602)<br>**St3gal6**, GXL_60809, GeneID: 304023, Rattus norvegicus chr. 11<br>ST3 beta-galactoside alpha-2,3-sialyltransferase 6	EGRF-SP1F	105 - 125	(+)
**GXP_73139** [GXP_73139] (1 - 671)<br>**B4galt4**, GXL_60824, GeneID: 303923, Rattus norvegicus chr. 11<br>UDP-Gal:betaGlcNAc beta 1,4-galactosyltransferase, polypeptide 4	EGRF-SP1F	531 - 510	(-)
**GXP_1403875** [GXP_1403875] (1 - 601)<br>**B4galt4**, GXL_60824, GeneID: 303923, Rattus norvegicus chr. 11<br>UDP-Gal:betaGlcNAc beta 1,4-galactosyltransferase, polypeptide 4	EGRF-SP1F	447 - 426	(-)
**GXP_1404054** [GXP_1404054] (1 - 656)<br>**Dgkg**, GXL_60839, GeneID: 25666, Rattus norvegicus chr. 11<br>diacylglycerol kinase, gamma	EGRF-SP1F	32 - 55	(+)
**GXP_73158** [GXP_73158] (1 - 703)<br>**Ncam2**, GXL_60843, GeneID: 288280, Rattus norvegicus chr. 11<br>neural cell adhesion molecule 2	EGRF-SP1F	462 - 482	(+)
	EGRF-SP1F	470 - 492	(+)
**GXP_73159** [GXP_73159] (1 - 601)<br>**Adamts5**, GXL_60844, GeneID: 304135, Rattus norvegicus chr. 11<br>a disintegrin-like and metallopeptidase (reprolysin type) with thrombospondin type 1 motif, 5 (aggrecanase-2)	EGRF-SP1F	399 - 423	(+)
**GXP_73168** [GXP_73168] (1 - 955)<br>**Tnk2**, GXL_60853, GeneID: 303882, Rattus norvegicus chr. 11<br>tyrosine kinase, non-receptor, 2	EGRF-SP1F	77 - 53	(-)
	EGRF-SP1F	272 - 251	(-)
**GXP_1149316** [GXP_1149316] (1 - 601)<br>**Tnk2**, GXL_60853, GeneID: 303882, Rattus norvegicus chr. 11<br>tyrosine kinase, non-receptor, 2	EGRF-SP1F	273 - 294	(+)
**GXP_1403972** [GXP_1403972] (1 - 602)<br>**Tnk2**, GXL_60853, GeneID: 303882, Rattus norvegicus chr. 11<br>tyrosine kinase, non-receptor, 2	EGRF-SP1F	435 - 457	(+)
	EGRF-SP1F	393 - 373	(-)

**GXP_73173** [GXP_73173] (1 - 668)<br>**Cxadr**, GXL_60858, GeneID: 89843, Rattus norvegicus chr. 11<br>coxsackie virus and adenovirus receptor	EGRF-SP1F	399 - 375	(-)
**GXP_73189** [GXP_73189] (1 - 601)<br>**Gsk3b**, GXL_60874, GeneID: 84027, Rattus norvegicus chr. 11<br>glycogen synthase kinase 3 beta	EGRF-SP1F	27 - 48	(+)
**GXP_73209** [GXP_73209] (1 - 601)<br>**Atp5j**, GXL_60894, GeneID: 94271, Rattus norvegicus chr. 11<br>ATP synthase, H+ transporting, mitochondrial F0 complex, subunit F6	EGRF-SP1F	583 - 563	(-)
**GXP_73215** [GXP_73215] (1 - 839)<br>**Clcn2**, GXL_60900, GeneID: 29232, Rattus norvegicus chr. 11<br>chloride channel 2	EGRF-SP1F	285 - 308	(+)
**GXP_73243** [GXP_73243] (1 - 601)<br>**Ap2m1**, GXL_60928, GeneID: 116563, Rattus norvegicus chr. 11<br>adaptor-related protein complex 2, mu 1 subunit	EGRF-SP1F	240 - 220	(-)
**GXP_73247** [GXP_73247] (1 - 1041)<br>**Sfrs10**, GXL_60932, GeneID: 117259, Rattus norvegicus chr. 11<br>splicing factor, arginine/serine-rich 10 (transformer 2 homolog, Drosophila)	EGRF-SP1F	868 - 848	(-)
**GXP_73256** [GXP_73256] (1 - 601)<br>**Clic6**, GXL_60941, GeneID: 304081, Rattus norvegicus chr. 11<br>chloride intracellular channel 6	EGRF-SP1F	35 - 55	(+)
**GXP_73294** [GXP_73294] (1 - 601)<br>**Tssk2**, GXL_60979, GeneID: 304181, Rattus norvegicus chr. 11<br>testis-specific serine kinase 2	EGRF-SP1F	336 - 313	(-)
**GXP_73296** [GXP_73296] (1 - 847)<br>**Ifnar1_predicted**, GXL_60981, GeneID: 288264, Rattus norvegicus chr. 11<br>interferon (alpha and beta) receptor 1 (predicted)	EGRF-SP1F	667 - 646	(-)
**GXP_73336** [GXP_73336] (1 - 739)<br>**Kcne1**, GXL_61021, GeneID: 25471, Rattus norvegicus chr. 11<br>potassium voltage-gated channel, Isk-related subfamily, member 1	EGRF-SP1F	338 - 318	(-)
	EGRF-SP1F	343 - 320	(-)
**GXP_73356** [GXP_73356] (1 - 742)<br>**Cd47**, GXL_61041, GeneID: 29364, Rattus norvegicus chr. 11<br>CD47 antigen (Rh-related antigen, integrin-associated signal transducer)	EGRF-SP1F	555 - 535	(-)
**GXP_73359** [GXP_73359] (1 - 601)<br>**Adcy5**, GXL_61044, GeneID: 64532, Rattus norvegicus chr. 11<br>adenylate cyclase 5	EGRF-SP1F	282 - 303	(+)
**GXP_73380** [GXP_73380] (1 - 775)<br>**Rtn4r**, GXL_61065, GeneID: 113912, Rattus norvegicus chr. 11<br>reticulon 4 receptor	EGRF-SP1F	350 - 373	(+)
	EGRF-SP1F	448 - 427	(-)

**GXP_1408509** [GXP_1408509] (1 - 620)<br>**Tradd**, GXL_64615, GeneID: 246756, Rattus norvegicus chr. 19<br>TNFRSF1A-associated via death domain			
**GXP_1408439** [GXP_1408439] (1 - 601)<br>**Lphn1**, GXL_64617, GeneID: 65096, Rattus norvegicus chr. 19<br>latrophilin 1	EGRF-SP1F	196 - 220	(+)
**GXP_77652** [GXP_77652] (1 - 721)<br>**Dhps**, GXL_64625, GeneID: 288923, Rattus norvegicus chr. 19<br>deoxyhypusine synthase	EGRF-SP1F	504 - 526	(+)
**GXP_77664** [GXP_77664] (1 - 757)<br>**Cd97**, GXL_64637, GeneID: 361383, Rattus norvegicus chr. 19<br>CD97 antigen	EGRF-SP1F	752 - 731	(-)
**GXP_77688** [GXP_77688] (1 - 654)<br>**Tfip11**, GXL_64659, GeneID: 288718, Rattus norvegicus chr. 19<br>tuftelin interacting protein 11	EGRF-SP1F	631 - 608	(-)
**GXP_77691** [GXP_77691] (1 - 679)<br>**Slc12a4**, GXL_64661, GeneID: 29501, Rattus norvegicus chr. 19<br>solute carrier family 12, member 4	EGRF-SP1F	472 - 448	(-)
**GXP_1408375** [GXP_1408375] (1 - 603)<br>**Gpt2_predicted**, GXL_64667, GeneID: 307759, Rattus norvegicus chr. 19<br>glutamic pyruvate transaminase (alanine aminotransferase) 2 (predicted)	EGRF-SP1F	332 - 354	(+)
**GXP_1408573** [GXP_1408573] (1 - 658)<br>**Has3**, GXL_64675, GeneID: 266805, Rattus norvegicus chr. 19<br>hyaluronan synthase 3	EGRF-SP1F	121 - 101	(-)
**GXP_1408533** [GXP_1408533] (1 - 662)<br>**Ctcf**, GXL_64687, GeneID: 83726, Rattus norvegicus chr. 19<br>CCCTC-binding factor	EGRF-SP1F	117 - 137	(+)
	EGRF-SP1F	394 - 415	(+)
	EGRF-SP1F	414 - 436	(+)
**GXP_77741** [GXP_77741] (1 - 683)<br>**MAST1**, GXL_64693, GeneID: 353118, Rattus norvegicus chr. 19<br>microtubule associated serine/threonine kinase 1	EGRF-SP1F	438 - 459	(+)
	EGRF-SP1F	443 - 465	(+)
	EGRF-SP1F	447 - 469	(+)
**GXP_1408401** [GXP_1408401] (1 - 686)<br>**MAST1**, GXL_64693, GeneID: 353118, Rattus norvegicus chr. 19<br>microtubule associated serine/threonine kinase 1	EGRF-SP1F	505 - 525	(+)
**GXP_77750** [GXP_77750] (1 - 601)<br>**Bcar1**, GXL_64702, GeneID: 25414, Rattus norvegicus chr. 19<br>breast cancer anti-estrogen resistance 1	EGRF-SP1F	443 - 422	(-)
**GXP_508295** [GXP_508295] (1 - 601)<br>**Dok4_predicted**, GXL_64721, GeneID: 361364, Rattus norvegicus chr. 19<br>docking protein 4 (predicted)	EGRF-SP1F	437 - 413	(-)

Sequence	Matrix	Position	Strand
**GXP_77775** [GXP_77775] (1 - 1162)<br>**Egln1**, GXL_64725, GeneID: 308913, Rattus norvegicus chr. 19<br>EGL nine homolog 1 (C. elegans)	EGRF-SP1F	1122 - 1142	(+)
	EGRF-SP1F	1130 - 1152	(+)
**GXP_77781** [GXP_77781] (1 - 602)<br>**Gnao**, GXL_64729, GeneID: 50664, Rattus norvegicus chr. 19<br>guanine nucleotide binding protein, alpha o	EGRF-SP1F	129 - 109	(-)
**GXP_1408282** [GXP_1408282] (1 - 1241)<br>**Gnao**, GXL_64729, GeneID: 50664, Rattus norvegicus chr. 19<br>guanine nucleotide binding protein, alpha o	EGRF-SP1F	734 - 757	(+)
	EGRF-SP1F	1082 - 1062	(-)
**GXP_77791** [GXP_77791] (1 - 770)<br>**Vps4a**, GXL_64739, GeneID: 246772, Rattus norvegicus chr. 19<br>vacuolar protein sorting 4a (yeast)	EGRF-SP1F	247 - 270	(+)
**GXP_77808** [GXP_77808] (1 - 601)<br>**Pou4f2**, GXL_64755, GeneID: 171355, Rattus norvegicus chr. 19<br>POU domain, class 4, transcription factor 2	EGRF-SP1F	428 - 406	(-)
	EGRF-SP1F	432 - 410	(-)
	EGRF-SP1F	453 - 431	(-)
	EGRF-SP1F	457 - 435	(-)
	EGRF-SP1F	459 - 439	(-)
**GXP_1408268** [GXP_1408268] (1 - 668)<br>**Nup93**, GXL_64781, GeneID: 291874, Rattus norvegicus chr. 19<br>nucleoporin 93	EGRF-SP1F	420 - 400	(-)
**GXP_1408273** [GXP_1408273] (1 - 647)<br>**Nup93**, GXL_64781, GeneID: 291874, Rattus norvegicus chr. 19<br>nucleoporin 93	EGRF-SP1F	390 - 413	(+)
**GXP_77876** [GXP_77876] (1 - 753)<br>**Hsd11b2**, GXL_64822, GeneID: 25117, Rattus norvegicus chr. 19<br>hydroxysteroid 11-beta dehydrogenase 2	EGRF-SP1F	387 - 364	(-)
	EGRF-SP1F	389 - 369	(-)
	EGRF-SP1F	436 - 459	(+)
	EGRF-SP1F	447 - 470	(+)
	EGRF-SP1F	588 - 611	(+)
**GXP_77881** [GXP_77881] (1 - 601)<br>**Gpr56**, GXL_64827, GeneID: 260326, Rattus norvegicus chr. 19<br>G protein-coupled receptor 56	EGRF-SP1F	462 - 440	(-)
**GXP_77885** [GXP_77885] (1 - 615)<br>**Glg1**, GXL_64831, GeneID: 29476, Rattus norvegicus chr. 19<br>golgi apparatus protein 1	EGRF-SP1F	540 - 520	(-)
**GXP_77901** [GXP_77901] (1 - 797)<br>**Atp6v0d1**, GXL_64847, GeneID: 291969, Rattus norvegicus chr. 19<br>ATPase, H+ transporting, V0 subunit D isoform 1	EGRF-SP1F	96 - 73	(-)
**GXP_77913** [GXP_77913] (1 - 810)<br>**Cirh1a**, GXL_64858, GeneID: 291987, Rattus norvegicus chr. 19	EGRF-SP1F	348 - 370	(+)

cirrhosis, autosomal recessive 1A (human)	EGRF-SP1F	358 - 381	(+)
	EGRF-SP1F	405 - 427	(+)
**GXP_77946** [GXP_77946] (1 - 775)<br>**Cyb5b**, GXL_64891, GeneID: 80773, Rattus norvegicus chr. 19<br>cytochrome b5 type B	EGRF-SP1F	280 - 258	(-)
	EGRF-SP1F	286 - 264	(-)
**GXP_77973** [GXP_77973] (1 - 601)<br>**Siah1a**, GXL_64918, GeneID: 140941, Rattus norvegicus chr. 19<br>seven in absentia 1A	EGRF-SP1F	498 - 474	(-)
**GXP_77995** [GXP_77995] (1 - 720)<br>**Mbtps1**, GXL_64939, GeneID: 89842, Rattus norvegicus chr. 19<br>membrane-bound transcription factor peptidase, site 1	EGRF-SP1F	398 - 421	(+)
**GXP_1408707** [GXP_1408707] (1 - 602)<br>**Mbtps1**, GXL_64939, GeneID: 89842, Rattus norvegicus chr. 19<br>membrane-bound transcription factor peptidase, site 1	EGRF-SP1F	26 - 49	(+)
**GXP_77996** [GXP_77996] (1 - 601)<br>**Maf**, GXL_64940, GeneID: 54267, Rattus norvegicus chr. 19<br>v-maf musculoaponeurotic fibrosarcoma oncogene homolog (avian)	EGRF-SP1F	489 - 469	(-)
	EGRF-SP1F	501 - 477	(-)
**GXP_1408490** [GXP_1408490] (1 - 602)<br>**Mmaa_predicted**, GXL_64943, GeneID: 291939, Rattus norvegicus chr. 19<br>methylmalonic aciduria (cobalamin deficiency) cblA type (predicted)	EGRF-SP1F	488 - 468	(-)
**GXP_1408491** [GXP_1408491] (1 - 601)<br>**Mmaa_predicted**, GXL_64943, GeneID: 291939, Rattus norvegicus chr. 19<br>methylmalonic aciduria (cobalamin deficiency) cblA type (predicted)	EGRF-SP1F	232 - 212	(-)
**GXP_78006** [GXP_78006] (1 - 601)<br>**Pkn1**, GXL_64950, GeneID: 29355, Rattus norvegicus chr. 19<br>protein kinase N1	EGRF-SP1F	383 - 407	(+)
	EGRF-SP1F	389 - 412	(+)
**GXP_78009** [GXP_78009] (1 - 601)<br>**Sipa1l2**, GXL_64953, GeneID: 361442, Rattus norvegicus chr. 19<br>signal-induced proliferation-associated 1 like 2	EGRF-SP1F	462 - 439	(-)
	EGRF-SP1F	472 - 450	(-)
	EGRF-SP1F	475 - 454	(-)
**GXP_78023** [GXP_78023] (1 - 670)<br>**Rnf166**, GXL_64967, GeneID: 365022, Rattus norvegicus chr. 19<br>ring finger protein 166	EGRF-SP1F	578 - 554	(-)
**GXP_1408743** [GXP_1408743] (1 - 602)<br>**Rnf166**, GXL_64967, GeneID: 365022, Rattus norvegicus chr. 19<br>ring finger protein 166	EGRF-SP1F	405 - 383	(-)
**GXP_78024** [GXP_78024] (1 - 638)<br>**Pdp2**, GXL_64968, GeneID: 246311, Rattus norvegicus chr. 19<br>pyruvate dehydrogenase phosphatase isoenzyme 2	EGRF-SP1F	351 - 372	(+)
**GXP_78025** [GXP_78025] (1 - 1360)	EGRF-SP1F	372 - 349	(-)

**Rasd2**, GXL_64969, GeneID: 171099, Rattus norvegicus chr. 19<br>RASD family, member 2			
**GXP_1408292** [GXP_1408292] (1 - 601)<br>**Rasd2**, GXL_64969, GeneID: 171099, Rattus norvegicus chr. 19<br>RASD family, member 2	EGRF-SP1F	465 - 442	(-)
**GXP_78041** [GXP_78041] (1 - 948)<br>**Slc7a5**, GXL_64985, GeneID: 50719, Rattus norvegicus chr. 19<br>solute carrier family 7 (cationic amino acid transporter, y+ system), member 5	EGRF-SP1F	519 - 498	(-)
**GXP_78042** [GXP_78042] (1 - 757)<br>**Tomm20**, GXL_64986, GeneID: 266601, Rattus norvegicus chr. 19<br>translocase of outer mitochondrial membrane 20 homolog (yeast)	EGRF-SP1F	479 - 459	(-)
**GXP_78099** [GXP_78099] (1 - 785)<br>**Dnaja2**, GXL_65043, GeneID: 84026, Rattus norvegicus chr. 19<br>DnaJ (Hsp40) homolog, subfamily A, member 2	EGRF-SP1F	521 - 543	(+)
**GXP_1174919** [GXP_1174919] (1 - 601)<br>**Armcx6**, GXL_69230, GeneID: 363496, Rattus norvegicus chr. X<br>armadillo repeat containing, X-linked 6	EGRF-SP1F	150 - 174	(+)
**GXP_83294** [GXP_83294] (1 - 647)<br>**Bcap31**, GXL_69231, GeneID: 293852, Rattus norvegicus chr. X<br>B-cell receptor-associated protein 31	EGRF-SP1F<br>EGRF-SP1F<br>EGRF-SP1F	25 - 46<br>134 - 155<br>586 - 562	(+)<br>(+)<br>(-)
**GXP_83316** [GXP_83316] (1 - 761)<br>**Timp1**, GXL_69253, GeneID: 116510, Rattus norvegicus chr. X<br>tissue inhibitor of metallopeptidase 1	EGRF-SP1F	589 - 610	(+)
**GXP_83325** [GXP_83325] (1 - 664)<br>**Cacna1f**, GXL_69262, GeneID: 114493, Rattus norvegicus chr. X<br>calcium channel, voltage-dependent, alpha 1F subunit	EGRF-SP1F	430 - 450	(+)
**GXP_83346** [GXP_83346] (1 - 707)<br>**Gla**, GXL_69280, GeneID: 363494, Rattus norvegicus chr. X<br>galactosidase, alpha	EGRF-SP1F	91 - 68	(-)
**GXP_83374** [GXP_83374] (1 - 1117)<br>**Ndufb11_predicted**, GXL_69308, GeneID: 299310, Rattus norvegicus chr. X<br>NADH dehydrogenase (ubiquinone) 1 beta subcomplex, 11 (predicted)	EGRF-SP1F	70 - 46	(-)
**GXP_83385** [GXP_83385] (1 - 758)<br>**Igsf1**, GXL_69319, GeneID: 302822, Rattus norvegicus chr. X<br>immunoglobulin superfamily, member 1	EGRF-SP1F	560 - 536	(-)
**GXP_83400** [GXP_83400] (1 - 1127)<br>**Otc**, GXL_69334, GeneID: 25611, Rattus norvegicus chr. X<br>ornithine transcarbamylase	EGRF-SP1F	415 - 395	(-)
**GXP_1421009** [GXP_1421009] (1 - 1285)<br>**Mid1**, GXL_69368, GeneID: 54252, Rattus norvegicus chr. X	EGRF-SP1F	427 - 450	(+)

midline 1

**GXP_83441** [GXP_83441] (1 - 843)			
**Sh3kbp1**, GXL_69374, GeneID: 84357, Rattus norvegicus chr. X	EGRF-SP1F	210 - 234	(+)
SH3-domain kinase binding protein 1			
**GXP_1421058** [GXP_1421058] (1 - 640)			
**Sh3kbp1**, GXL_69374, GeneID: 84357, Rattus norvegicus chr. X	EGRF-SP1F	470 - 447	(-)
SH3-domain kinase binding protein 1			
**GXP_1421059** [GXP_1421059] (1 - 603)	EGRF-SP1F	73 - 53	(-)
**Sh3kbp1**, GXL_69374, GeneID: 84357, Rattus norvegicus chr. X	EGRF-SP1F	94 - 72	(-)
SH3-domain kinase binding protein 1	EGRF-SP1F	257 - 235	(-)
**GXP_83475** [GXP_83475] (1 - 602)	EGRF-SP1F	545 - 522	(-)
**Birc4**, GXL_69408, GeneID: 63879, Rattus norvegicus chr. X	EGRF-SP1F	547 - 527	(-)
baculoviral IAP repeat-containing 4	EGRF-SP1F	553 - 533	(-)
**GXP_1420813** [GXP_1420813] (1 - 612)	EGRF-SP1F	65 - 42	(-)
**Birc4**, GXL_69408, GeneID: 63879, Rattus norvegicus chr. X	EGRF-SP1F	67 - 47	(-)
baculoviral IAP repeat-containing 4	EGRF-SP1F	73 - 53	(-)
**GXP_83494** [GXP_83494] (1 - 977)			
**Gdi1**, GXL_69427, GeneID: 25183, Rattus norvegicus chr. X	EGRF-SP1F	484 - 462	(-)
guanosine diphosphate dissociation inhibitor 1			
**GXP_1421121** [GXP_1421121] (1 - 601)	EGRF-SP1F	226 - 250	(+)
**Maged1**, GXL_69461, GeneID: 84469, Rattus norvegicus chr. X			
melanoma antigen, family D, 1	EGRF-SP1F	478 - 498	(+)
**GXP_1421197** [GXP_1421197] (1 - 602)	EGRF-SP1F	427 - 403	(-)
**Taf9l**, GXL_69464, GeneID: 171152, Rattus norvegicus chr. X	EGRF-SP1F	433 - 409	(-)
TAF9-like RNA polymerase II, TATA box binding protein (TBP)-associated factor, 31kDa	EGRF-SP1F	435 - 415	(-)
**GXP_1421255** [GXP_1421255] (1 - 752)			
**Morf4l2**, GXL_69470, GeneID: 317413, Rattus norvegicus chr. X	EGRF-SP1F	165 - 143	(-)
mortality factor 4 like 2			
**GXP_1421256** [GXP_1421256] (1 - 602)			
**Morf4l2**, GXL_69470, GeneID: 317413, Rattus norvegicus chr. X	EGRF-SP1F	376 - 354	(-)
mortality factor 4 like 2			
**GXP_1174480** [GXP_1174480] (1 - 602)			
**Rbbp7**, GXL_69494, GeneID: 83712, Rattus norvegicus chr. X	EGRF-SP1F	91 - 113	(+)
retinoblastoma binding protein 7			
**GXP_524271** [GXP_524271] (1 - 601)			
**Foxp3_predicted**, GXL_69502, GeneID: 317382, Rattus norvegicus chr. X	EGRF-SP1F	510 - 487	(-)
forkhead box P3 (predicted)			
	EGRF-SP1F	270 - 290	(+)

**GXP_83576** [GXP_83576] (1 - 640)<br>**Mig12**, GXL_69508, GeneID: 404280, Rattus norvegicus chr. X<br>MID1 interacting G12-like protein			
**GXP_83595** [GXP_83595] (1 - 684)<br>**Atp1b4**, GXL_69526, GeneID: 84396, Rattus norvegicus chr. X<br>ATPase, (Na+)/K+ transporting, beta 4 polypeptide	EGRF-SP1F	123 - 143	(+)
**GXP_1421286** [GXP_1421286] (1 - 1106)<br>**Tsc22d3**, GXL_69534, GeneID: 83514, Rattus norvegicus chr. X<br>TSC22 domain family 3	EGRF-SP1F<br>EGRF-SP1F	632 - 609<br>636 - 614	(-)<br>(-)
**GXP_83609** [GXP_83609] (1 - 810)<br>**L1cam**, GXL_69540, GeneID: 50687, Rattus norvegicus chr. X<br>L1 cell adhesion molecule	EGRF-SP1F	595 - 619	(+)
**GXP_1421408** [GXP_1421408] (1 - 687)<br>**L1cam**, GXL_69540, GeneID: 50687, Rattus norvegicus chr. X<br>L1 cell adhesion molecule	EGRF-SP1F	466 - 488	(+)
**GXP_83622** [GXP_83622] (1 - 1216)<br>**Rgn**, GXL_69553, GeneID: 25106, Rattus norvegicus chr. X<br>regucalcin	EGRF-SP1F	335 - 358	(+)
**GXP_83634** [GXP_83634] (1 - 635)<br>**Gripap1**, GXL_69565, GeneID: 116493, Rattus norvegicus chr. X<br>GRIP1 associated protein 1	EGRF-SP1F<br>EGRF-SP1F	532 - 512<br>537 - 514	(-)<br>(-)
**GXP_1174253** [GXP_1174253] (1 - 601)<br>**Gripap1**, GXL_69565, GeneID: 116493, Rattus norvegicus chr. X<br>GRIP1 associated protein 1	EGRF-SP1F	75 - 95	(+)
**GXP_1421154** [GXP_1421154] (1 - 603)<br>**Slc7a3**, GXL_69585, GeneID: 29485, Rattus norvegicus chr. X<br>solute carrier family 7 (cationic amino acid transporter, y+ system), member 3	EGRF-SP1F	195 - 215	(+)
**GXP_83663** [GXP_83663] (1 - 655)<br>**Atp6ap1**, GXL_69594, GeneID: 83615, Rattus norvegicus chr. X<br>ATPase, H+ transporting, lysosomal accessory protein 1	EGRF-SP1F	508 - 485	(-)
**GXP_1421424** [GXP_1421424] (1 - 601)<br>**Atp6ap1**, GXL_69594, GeneID: 83615, Rattus norvegicus chr. X<br>ATPase, H+ transporting, lysosomal accessory protein 1	EGRF-SP1F	318 - 295	(-)
**GXP_83674** [GXP_83674] (1 - 626)<br>**Apln**, GXL_69605, GeneID: 58812, Rattus norvegicus chr. X<br>apelin, AGTRL1 ligand	EGRF-SP1F<br>EGRF-SP1F<br>EGRF-SP1F	396 - 418<br>402 - 424<br>406 - 430	(+)<br>(+)<br>(+)
**GXP_1421022** [GXP_1421022] (1 - 696)<br>**Gpm6b**, GXL_69607, GeneID: 192179, Rattus norvegicus chr. X<br>glycoprotein m6b	EGRF-SP1F<br>EGRF-SP1F<br>EGRF-SP1F	289 - 312<br>295 - 315<br>237 - 213	(+)<br>(+)<br>(-)

GXP_83700 [GXP_83700] (1 - 602)
Slc38a5, GXL_69631, GeneID: 192208, Rattus norvegicus chr. X
solute carrier family 38, member 5

GXP_1420900 [GXP_1420900] (1 - 842)
Slc38a5, GXL_69631, GeneID: 192208, Rattus norvegicus chr. X
solute carrier family 38, member 5

EGRF-SP1F 813 - 791 (-)

GXP_1421381 [GXP_1421381] (1 - 1469)
Gabra3, GXL_69638, GeneID: 24947, Rattus norvegicus chr. X
gamma-aminobutyric acid (GABA-A) receptor, subunit alpha 3

EGRF-SP1F 993 - 971 (-)

GXP_1421240 [GXP_1421240] (1 - 628)
Btk, GXL_69696, GeneID: 367901, Rattus norvegicus chr. X
Bruton agammaglobulinemia tyrosine kinase

EGRF-SP1F 375 - 354 (-)

GXP_102713 [GXP_102713] (1 - 648)
Dgcr14, GXL_86327, GeneID: 360741, Rattus norvegicus chr. 11
DiGeorge syndrome critical region gene 14 homolog (human)

EGRF-SP1F 510 - 531 (+)

GXP_102724 [GXP_102724] (1 - 830)
Dgcr2, GXL_86337, GeneID: 360742, Rattus norvegicus chr. 11
DiGeorge syndrome critical region gene 2

EGRF-SP1F 237 - 257 (+)
EGRF-SP1F 493 - 513 (+)

GXP_102739 [GXP_102739] (1 - 1010)
Slc25a1, GXL_86351, GeneID: 29743, Rattus norvegicus chr. 11
solute carrier family 25, member 1

EGRF-SP1F 803 - 780 (-)

GXP_1403539 [GXP_1403539] (1 - 601)
Sept9, GXL_86458, GeneID: 83788, Rattus norvegicus chr. 10
septin 9

EGRF-SP1F 424 - 402 (-)
EGRF-SP1F 430 - 450 (+)
EGRF-SP1F 590 - 570 (-)

GXP_102859 [GXP_102859] (1 - 837)
Slc9a3r1, GXL_86465, GeneID: 59114, Rattus norvegicus chr. 10
solute carrier family 9 (sodium/hydrogen exchanger), isoform 3 regulator 1

EGRF-SP1F 92 - 70 (-)

GXP_102869 [GXP_102869] (1 - 1428)
Axin2, GXL_86473, GeneID: 29134, Rattus norvegicus chr. 10
axin2

EGRF-SP1F 209 - 186 (-)
EGRF-SP1F 213 - 191 (-)

GXP_1403405 [GXP_1403405] (1 - 659)
Axin2, GXL_86473, GeneID: 29134, Rattus norvegicus chr. 10
axin2

EGRF-SP1F 452 - 429 (-)

GXP_1403441 [GXP_1403441] (1 - 602)
Dnai2, GXL_86480, GeneID: 360654, Rattus norvegicus chr. 10
dynein, axonemal, intermediate polypeptide 2

EGRF-SP1F 384 - 360 (-)

GXP_102880 [GXP_102880] (1 - 863)
Slc16a6, GXL_86484, GeneID: 303772, Rattus norvegicus chr. 10
solute carrier family 16 (monocarboxylic acid transporters), member 6

EGRF-SP1F 587 - 564 (-)

EGRF-SP1F 198 - 177 (-)

**GXP_1403408** [GXP_1403408] (1 - 644)<br>**Slc16a6**, GXL_86484, GeneID: 303772, Rattus norvegicus chr. 10<br>solute carrier family 16 (monocarboxylic acid transporters), member 6			
**GXP_102904** [GXP_102904] (1 - 773)<br>**Mrps7**, GXL_86508, GeneID: 113958, Rattus norvegicus chr. 10<br>mitchondrial ribosomal protein S7	EGRF-SP1F	260 - 237	(-)
**GXP_107734** [GXP_107734] (1 - 1016)<br>**Vegfc**, GXL_90006, GeneID: 114111, Rattus norvegicus chr. 16<br>vascular endothelial growth factor C	EGRF-SP1F	93 - 115	(+)
	EGRF-SP1F	434 - 454	(+)
**GXP_107743** [GXP_107743] (1 - 602)<br>**Lamp1**, GXL_90015, GeneID: 25328, Rattus norvegicus chr. 16<br>lysosomal membrane glycoprotein 1	EGRF-SP1F	41 - 61	(+)
**GXP_107765** [GXP_107765] (1 - 723)<br>**Myo9b**, GXL_90036, GeneID: 25486, Rattus norvegicus chr. 16<br>myosin IXb	EGRF-SP1F	660 - 640	(-)
**GXP_1407147** [GXP_1407147] (1 - 601)<br>**Upf3a**, GXL_90080, GeneID: 361176, Rattus norvegicus chr. 16<br>UPF3 regulator of nonsense transcripts homolog A (yeast)	EGRF-SP1F	476 - 455	(-)
**GXP_107831** [GXP_107831] (1 - 601)<br>**Prkcd**, GXL_90086, GeneID: 170538, Rattus norvegicus chr. 16<br>protein kinase C, delta	EGRF-SP1F	353 - 374	(+)
**GXP_1406736** [GXP_1406736] (1 - 602)<br>**Prkcd**, GXL_90086, GeneID: 170538, Rattus norvegicus chr. 16<br>protein kinase C, delta	EGRF-SP1F	56 - 33	(-)
	EGRF-SP1F	358 - 336	(-)
**GXP_107851** [GXP_107851] (1 - 751)<br>**Cacna2d3**, GXL_90106, GeneID: 306243, Rattus norvegicus chr. 16<br>calcium channel, voltage-dependent, alpha 2/delta 3 subunit	EGRF-SP1F	156 - 176	(+)
	EGRF-SP1F	337 - 357	(+)
	EGRF-SP1F	468 - 491	(+)
	EGRF-SP1F	479 - 500	(+)
**GXP_1406728** [GXP_1406728] (1 - 631)<br>**Chdh**, GXL_90115, GeneID: 290551, Rattus norvegicus chr. 16<br>choline dehydrogenase	EGRF-SP1F	200 - 180	(-)
	EGRF-SP1F	202 - 182	(-)
**GXP_1406803** [GXP_1406803] (1 - 611)<br>**Bmpr1a**, GXL_90129, GeneID: 81507, Rattus norvegicus chr. 16<br>bone morphogenetic protein receptor, type 1A	EGRF-SP1F	365 - 388	(+)
	EGRF-SP1F	491 - 470	(-)
**GXP_107885** [GXP_107885] (1 - 601)<br>**Lzts1**, GXL_90139, GeneID: 266711, Rattus norvegicus chr. 16<br>leucine zipper, putative tumor suppressor 1	EGRF-SP1F	420 - 442	(+)
	EGRF-SP1F	425 - 447	(+)
	EGRF-SP1F	430 - 452	(+)
	EGRF-SP1F	440 - 462	(+)
	EGRF-SP1F	685 - 663	(-)

**GXP_107891** [GXP_107891] (1 - 915)<br>**Ppp2cb**, GXL_90145, GeneID: 24673, Rattus norvegicus chr. 16<br>protein phosphatase 2 (formerly 2A), catalytic subunit, beta isoform			
**GXP_1406705** [GXP_1406705] (1 - 602)<br>**Arf4**, GXL_90154, GeneID: 79120, Rattus norvegicus chr. 16<br>ADP-ribosylation factor 4	EGRF-SP1F	246 - 266	(+)
**GXP_107911** [GXP_107911] (1 - 691)<br>**Fgfr1**, GXL_90165, GeneID: 79114, Rattus norvegicus chr. 16<br>Fibroblast growth factor receptor 1	EGRF-SP1F	346 - 326	(-)
**GXP_107919** [GXP_107919] (1 - 846)<br>**Sncg**, GXL_90173, GeneID: 64347, Rattus norvegicus chr. 16<br>synuclein, gamma	EGRF-SP1F	557 - 578	(+)
**GXP_1406888** [GXP_1406888] (1 - 602)<br>**Homer3**, GXL_90183, GeneID: 29548, Rattus norvegicus chr. 16<br>homer homolog 3 (Drosophila)	EGRF-SP1F	46 - 67	(+)
**GXP_107935** [GXP_107935] (1 - 1055)<br>**Dusp4**, GXL_90189, GeneID: 60587, Rattus norvegicus chr. 16<br>dual specificity phosphatase 4	EGRF-SP1F<br>EGRF-SP1F	141 - 121<br>469 - 446	(-)<br>(-)
**GXP_107937** [GXP_107937] (1 - 667)<br>**Slc27a1**, GXL_90191, GeneID: 94172, Rattus norvegicus chr. 16<br>solute carrier family 27 (fatty acid transporter), member 1	EGRF-SP1F	497 - 475	(-)
**GXP_1407166** [GXP_1407166] (1 - 601)<br>**F7**, GXL_90200, GeneID: 260320, Rattus norvegicus chr. 16<br>coagulation factor VII	EGRF-SP1F	549 - 569	(+)
**GXP_107999** [GXP_107999] (1 - 902)<br>**Ghitm**, GXL_90249, GeneID: 290596, Rattus norvegicus chr. 16<br>growth hormone inducible transmembrane protein	EGRF-SP1F	886 - 863	(-)
**GXP_108003** [GXP_108003] (1 - 601)<br>**Casp3**, GXL_90253, GeneID: 25402, Rattus norvegicus chr. 16<br>caspase 3, apoptosis related cysteine protease	EGRF-SP1F<br>EGRF-SP1F	454 - 431<br>465 - 442	(-)<br>(-)
**GXP_108022** [GXP_108022] (1 - 601)<br>**Itih3**, GXL_90272, GeneID: 50693, Rattus norvegicus chr. 16<br>inter-alpha trypsin inhibitor, heavy chain 3	EGRF-SP1F	369 - 389	(+)
**GXP_108068** [GXP_108068] (1 - 795)<br>**Mustn1**, GXL_90318, GeneID: 290553, Rattus norvegicus chr. 16<br>musculoskeletal, embryonic nuclear protein 1	EGRF-SP1F	368 - 388	(+)
**GXP_1407121** [GXP_1407121] (1 - 601)<br>**Vps36_predicted**, GXL_90322, GeneID: 290851, Rattus norvegicus chr. 16<br>vacuolar protein sorting 36 (yeast) (predicted)	EGRF-SP1F	225 - 245	(+)
	EGRF-SP1F	124 - 104	(-)

**GXP_108098** [GXP_108098] (1 - 601)<br>**Mk1**, GXL_90348, GeneID: 171436, Rattus norvegicus chr. 16<br>Mk1 protein			
**GXP_108112** [GXP_108112] (1 - 672)<br>**Spfh2_predicted**, GXL_90362, GeneID: 290823, Rattus norvegicus chr. 16<br>SPFH domain family, member 2 (predicted)	EGRF-SP1F	476 - 456	(-)
**GXP_1406943** [GXP_1406943] (1 - 768)<br>**Sh3md2**, GXL_90363, GeneID: 306417, Rattus norvegicus chr. 16<br>putative scaffolding protein POSH	EGRF-SP1F	594 - 572	(-)
**GXP_108117** [GXP_108117] (1 - 601)<br>**B3gnt3_predicted**, GXL_90367, GeneID: 290638, Rattus norvegicus chr. 16<br>UDP-GlcNAc:betaGal beta-1, 3-N-acetylglucosaminyltransferase 3 (predicted)	EGRF-SP1F<br>EGRF-SP1F<br>EGRF-SP1F	201 - 181<br>443 - 423<br>449 - 427	(-)<br>(-)<br>(-)
**GXP_1406725** [GXP_1406725] (1 - 967)<br>**Wnt5a**, GXL_90368, GeneID: 64566, Rattus norvegicus chr. 16<br>wingless-type MMTV integration site 5A	EGRF-SP1F	433 - 457	(+)
**GXP_108131** [GXP_108131] (1 - 745)<br>**Sh3bp5**, GXL_90381, GeneID: 117186, Rattus norvegicus chr. 16<br>SH3-domain binding protein 5 (BTK-associated)	EGRF-SP1F	35 - 55	(+)
**GXP_1406767** [GXP_1406767] (1 - 620)<br>**Sh3bp5**, GXL_90381, GeneID: 117186, Rattus norvegicus chr. 16<br>SH3-domain binding protein 5 (BTK-associated)	EGRF-SP1F	37 - 16	(-)
**GXP_108137** [GXP_108137] (1 - 1150)<br>**Nr2f6**, GXL_90387, GeneID: 245980, Rattus norvegicus chr. 16<br>nuclear receptor subfamily 2, group F, member 6	EGRF-SP1F<br>EGRF-SP1F<br>EGRF-SP1F	159 - 136<br>360 - 382<br>475 - 498	(-)<br>(+)<br>(+)
**GXP_1153595** [GXP_1153595] (1 - 601)<br>**Nr2f6**, GXL_90387, GeneID: 245980, Rattus norvegicus chr. 16<br>nuclear receptor subfamily 2, group F, member 6	EGRF-SP1F<br>EGRF-SP1F	71 - 51<br>80 - 59	(-)<br>(-)
**GXP_108154** [GXP_108154] (1 - 1099)<br>**Glt8d1**, GXL_90404, GeneID: 306253, Rattus norvegicus chr. 16<br>glycosyltransferase 8 domain containing 1	EGRF-SP1F<br>EGRF-SP1F<br>EGRF-SP1F	953 - 931<br>959 - 936<br>961 - 941	(-)<br>(-)<br>(-)
**GXP_108156** [GXP_108156] (1 - 728)<br>**Aadat**, GXL_90406, GeneID: 29416, Rattus norvegicus chr. 16<br>aminoadipate aminotransferase	EGRF-SP1F	398 - 374	(-)
**GXP_1407005** [GXP_1407005] (1 - 616)<br>**Argbp2**, GXL_90408, GeneID: 114901, Rattus norvegicus chr. 16<br>Arg/Abl-interacting protein ArgBP2	EGRF-SP1F	133 - 113	(-)
**GXP_1406873** [GXP_1406873] (1 - 602)<br>**Rab3a**, GXL_90422, GeneID: 25531, Rattus norvegicus chr. 16<br>RAB3A, member RAS oncogene family	EGRF-SP1F<br>EGRF-SP1F	22 - 1<br>26 - 5	(-)<br>(-)

**GXP_114847** [GXP_114847] (1 - 1087)<br>**Edn1**, GXL_96186, GeneID: 24323, Rattus norvegicus chr. 17<br>endothelin 1	EGRF-SP1F<br>EGRF-SP1F<br>EGRF-SP1F	178 - 198<br>304 - 327<br>306 - 329	(+)<br>(+)<br>(+)
**GXP_1407411** [GXP_1407411] (1 - 602)<br>**Tcfap2a_predicted**, GXL_96212, GeneID: 306862, Rattus norvegicus chr. 17<br>transcription factor AP-2, alpha (predicted)	EGRF-SP1F	421 - 443	(+)
**GXP_114900** [GXP_114900] (1 - 606)<br>**Kif27**, GXL_96239, GeneID: 246209, Rattus norvegicus chr. 17<br>kinesin family member 27	EGRF-SP1F	116 - 96	(-)
**GXP_114905** [GXP_114905] (1 - 772)<br>**Smad5**, GXL_96244, GeneID: 59328, Rattus norvegicus chr. 17<br>MAD homolog 5 (Drosophila)	EGRF-SP1F	352 - 332	(-)
**GXP_1407270** [GXP_1407270] (1 - 669)<br>**Smad5**, GXL_96244, GeneID: 59328, Rattus norvegicus chr. 17<br>MAD homolog 5 (Drosophila)	EGRF-SP1F<br>EGRF-SP1F<br>EGRF-SP1F	437 - 458<br>446 - 467<br>450 - 472	(+)<br>(+)<br>(+)
**GXP_114917** [GXP_114917] (1 - 601)<br>**Srd5a1**, GXL_96256, GeneID: 24950, Rattus norvegicus chr. 17<br>steroid 5 alpha-reductase 1	EGRF-SP1F	490 - 470	(-)
**GXP_1154201** [GXP_1154201] (1 - 601)<br>**Srd5a1**, GXL_96256, GeneID: 24950, Rattus norvegicus chr. 17<br>steroid 5 alpha-reductase 1	EGRF-SP1F	402 - 382	(-)
**GXP_1154202** [GXP_1154202] (1 - 1216)<br>**Srd5a1**, GXL_96256, GeneID: 24950, Rattus norvegicus chr. 17<br>steroid 5 alpha-reductase 1	EGRF-SP1F<br>EGRF-SP1F<br>EGRF-SP1F<br>EGRF-SP1F<br>EGRF-SP1F	62 - 85<br>68 - 88<br>87 - 107<br>292 - 316<br>565 - 541	(+)<br>(+)<br>(+)<br>(+)<br>(-)
**GXP_114924** [GXP_114924] (1 - 660)<br>**Mak10**, GXL_96263, GeneID: 64472, Rattus norvegicus chr. 17<br>MAK10 homolog, amino-acid N-acetyltransferase subunit, (S. cerevisiae)	EGRF-SP1F	571 - 547	(-)
**GXP_114935** [GXP_114935] (1 - 748)<br>**Sephs1**, GXL_96274, GeneID: 291314, Rattus norvegicus chr. 17<br>selenophosphate synthetase 1	EGRF-SP1F	423 - 446	(+)
**GXP_114947** [GXP_114947] (1 - 793)<br>**Sncb**, GXL_96286, GeneID: 113893, Rattus norvegicus chr. 17<br>synuclein, beta	EGRF-SP1F	453 - 474	(+)
**GXP_1407332** [GXP_1407332] (1 - 602)<br>**Nfil3**, GXL_96293, GeneID: 114519, Rattus norvegicus chr. 17<br>nuclear factor, interleukin 3 regulated	EGRF-SP1F<br>EGRF-SP1F	402 - 380<br>407 - 385	(-)<br>(-)
**GXP_114992** [GXP_114992] (1 - 673)<br>**Dek**, GXL_96330, GeneID: 306817, Rattus norvegicus chr. 17	EGRF-SP1F	347 - 367	(+)

DEK oncogene (DNA binding)	EGRF-SP1F	408 - 428	(+)
**GXP_1407365** [GXP_1407365] (1 - 609)			
**Dek**, GXL_96330, GeneID: 306817, Rattus norvegicus chr. 17	EGRF-SP1F	542 - 565	(+)
DEK oncogene (DNA binding)			
**GXP_115021** [GXP_115021] (1 - 647)			
**Nedd9**, GXL_96359, GeneID: 291044, Rattus norvegicus chr. 17	EGRF-SP1F	427 - 404	(-)
neural precursor cell expressed, developmentally down-regulated gene 9			
**GXP_1407401** [GXP_1407401] (1 - 603)			
**Nedd9**, GXL_96359, GeneID: 291044, Rattus norvegicus chr. 17	EGRF-SP1F	2 - 22	(+)
neural precursor cell expressed, developmentally down-regulated gene 9			
**GXP_115023** [GXP_115023] (1 - 918)	EGRF-SP1F	560 - 537	(-)
**Bicd2**, GXL_96361, GeneID: 306809, Rattus norvegicus chr. 17			
bicaudal D homolog 2 (Drosophila)	EGRF-SP1F	563 - 542	(-)
**GXP_115032** [GXP_115032] (1 - 999)	EGRF-SP1F	537 - 557	(+)
**Barx1_predicted**, GXL_96369, GeneID: 364680, Rattus norvegicus chr. 17			
BarH-like homeobox 1 (predicted)	EGRF-SP1F	539 - 562	(+)
**GXP_1407671** [GXP_1407671] (1 - 731)			
**Nmt2**, GXL_96381, GeneID: 291318, Rattus norvegicus chr. 17	EGRF-SP1F	466 - 446	(-)
N-myristoyltransferase 2			
	EGRF-SP1F	444 - 421	(-)
**GXP_115063** [GXP_115063] (1 - 675)			
**Mxd3**, GXL_96400, GeneID: 252915, Rattus norvegicus chr. 17	EGRF-SP1F	455 - 432	(-)
Max dimerization protein 3	EGRF-SP1F	460 - 437	(-)
	EGRF-SP1F	469 - 448	(-)
**GXP_115066** [GXP_115066] (1 - 601)			
**Ddx46**, GXL_96403, GeneID: 245957, Rattus norvegicus chr. 17	EGRF-SP1F	83 - 105	(+)
DEAD (Asp-Glu-Ala-Asp) box polypeptide 46			
**GXP_115096** [GXP_115096] (1 - 601)			
**Neurod3**, GXL_96433, GeneID: 29410, Rattus norvegicus chr. 17	EGRF-SP1F	326 - 303	(-)
neurogenic differentiation 3			
**GXP_115097** [GXP_115097] (1 - 601)	EGRF-SP1F	365 - 344	(-)
**Arid4b**, GXL_96434, GeneID: 84481, Rattus norvegicus chr. 17			
AT rich interactive domain 4B (Rbp1 like)	EGRF-SP1F	445 - 425	(-)
**GXP_1407262** [GXP_1407262] (1 - 602)			
**Gkap1**, GXL_96435, GeneID: 361202, Rattus norvegicus chr. 17	EGRF-SP1F	472 - 494	(+)
G kinase anchoring protein 1			
**GXP_115122** [GXP_115122] (1 - 758)			
**Gprk6**, GXL_96459, GeneID: 59076, Rattus norvegicus chr. 17	EGRF-SP1F	469 - 447	(-)
G protein-coupled receptor kinase 6			
**GXP_115125** [GXP_115125] (1 - 756)	EGRF-SP1F	176 - 200	(+)

**Hk3**, GXL_96462, GeneID: 25060, Rattus norvegicus chr. 17<br>hexokinase 3	EGRF-SP1F	182 - 202	(+)
**GXP_115131** [GXP_115131] (1 - 601)<br>**Ctsl**, GXL_96468, GeneID: 25697, Rattus norvegicus chr. 17<br>cathepsin L	EGRF-SP1F	562 - 585	(+)
**GXP_1407507** [GXP_1407507] (1 - 816)<br>**Inhba**, GXL_96480, GeneID: 29200, Rattus norvegicus chr. 17<br>inhibin beta-A	EGRF-SP1F	261 - 282	(+)
**GXP_115144** [GXP_115144] (1 - 684)<br>**Serpinb6b**, GXL_96481, GeneID: 364705, Rattus norvegicus chr. 17<br>serine (or cysteine) proteinase inhibitor, clade B, member 6b	EGRF-SP1F	498 - 521	(+)
**GXP_1154243** [GXP_1154243] (1 - 794)<br>**Fbp1**, GXL_96520, GeneID: 24362, Rattus norvegicus chr. 17<br>fructose-1,6- biphosphatase 1	EGRF-SP1F	276 - 255	(-)
**GXP_115189** [GXP_115189] (1 - 601)<br>**Hist1h1t**, GXL_96526, GeneID: 24438, Rattus norvegicus chr. 17<br>histone cluster 1, H1t	EGRF-SP1F	438 - 417	(-)
**GXP_115194** [GXP_115194] (1 - 1225)<br>**St8sia6**, GXL_96531, GeneID: 291325, Rattus norvegicus chr. 17<br>ST8 alpha-N-acetyl-neuraminide alpha-2, 8-sialyltransferase 6	EGRF-SP1F<br>EGRF-SP1F	1017 - 995<br>1197 - 1221	(-)<br>(+)
**GXP_115212** [GXP_115212] (1 - 667)<br>**Exoc2**, GXL_96549, GeneID: 171455, Rattus norvegicus chr. 17<br>exocyst complex component 2	EGRF-SP1F	461 - 482	(+)
**GXP_115217** [GXP_115217] (1 - 662)<br>**Uqcrfs1**, GXL_96554, GeneID: 291103, Rattus norvegicus chr. 17<br>ubiquinol-cytochrome c reductase, Rieske iron-sulfur polypeptide 1	EGRF-SP1F	379 - 399	(+)
**GXP_1407220** [GXP_1407220] (1 - 739)<br>**Ptch1**, GXL_96558, GeneID: 89830, Rattus norvegicus chr. 17<br>patched homolog 1 (Drosophila)	EGRF-SP1F	232 - 252	(+)
**GXP_1407362** [GXP_1407362] (1 - 603)<br>**Id4**, GXL_96561, GeneID: 291023, Rattus norvegicus chr. 17<br>inhibitor of DNA binding 4	EGRF-SP1F<br>EGRF-SP1F	401 - 379<br>407 - 385	(-)<br>(-)
**GXP_115225** [GXP_115225] (1 - 669)<br>**Ninj1**, GXL_96562, GeneID: 25338, Rattus norvegicus chr. 17<br>ninjurin 1	EGRF-SP1F<br>EGRF-SP1F<br>EGRF-SP1F	235 - 255<br>488 - 466<br>499 - 476	(+)<br>(-)<br>(-)
**GXP_1407300** [GXP_1407300] (1 - 602)<br>**Dbn1**, GXL_96581, GeneID: 81653, Rattus norvegicus chr. 17<br>drebrin 1	EGRF-SP1F	276 - 299	(+)
**GXP_115901** [GXP_115901] (1 - 608)<br>**Il12b**, GXL_97158, GeneID: 64546, Rattus norvegicus chr. 10	EGRF-SP1F	472 - 492	(+)

interleukin 12b

**GXP_115908** [GXP_115908] (1 - 970)<br>**Kcnmb1**, GXL_97165, GeneID: 29747, Rattus norvegicus chr. 10<br>potassium large conductance calcium-activated channel, subfamily M, beta member 1	EGRF-SP1F	863 - 843	(-)
	EGRF-SP1F	869 - 849	(-)
**GXP_115915** [GXP_115915] (1 - 896)<br>**Timp2**, GXL_97172, GeneID: 29543, Rattus norvegicus chr. 10<br>tissue inhibitor of metalloproteinase 2	EGRF-SP1F	317 - 294	(-)
	EGRF-SP1F	323 - 299	(-)
**GXP_115916** [GXP_115916] (1 - 601)<br>**Neurod2**, GXL_97173, GeneID: 54276, Rattus norvegicus chr. 10<br>neurogenic differentiation 2	EGRF-SP1F	226 - 246	(+)
	EGRF-SP1F	430 - 410	(-)
**GXP_115928** [GXP_115928] (1 - 851)<br>**Axin1**, GXL_97185, GeneID: 79257, Rattus norvegicus chr. 10<br>axin 1	EGRF-SP1F	360 - 383	(+)
**GXP_1402311** [GXP_1402311] (1 - 606)<br>**Tmem8_predicted**, GXL_97192, GeneID: 303004, Rattus norvegicus chr. 10<br>transmembrane protein 8 (five membrane-spanning domains) (predicted)	EGRF-SP1F	186 - 209	(+)
**GXP_1402313** [GXP_1402313] (1 - 602)<br>**Tmem8_predicted**, GXL_97192, GeneID: 303004, Rattus norvegicus chr. 10<br>transmembrane protein 8 (five membrane-spanning domains) (predicted)	EGRF-SP1F	329 - 353	(+)
**GXP_115938** [GXP_115938] (1 - 601)<br>**Prm3**, GXL_97195, GeneID: 442921, Rattus norvegicus chr. 10<br>protamine 3	EGRF-SP1F	219 - 197	(-)
	EGRF-SP1F	522 - 500	(-)
**GXP_115941** [GXP_115941] (1 - 1044)<br>**Cygb**, GXL_97198, GeneID: 170520, Rattus norvegicus chr. 10<br>cytoglobin	EGRF-SP1F	837 - 859	(+)
	EGRF-SP1F	853 - 875	(+)
**GXP_115960** [GXP_115960] (1 - 881)<br>**Cacng4**, GXL_97217, GeneID: 140725, Rattus norvegicus chr. 10<br>calcium channel, voltage-dependent, gamma subunit 4	EGRF-SP1F	742 - 720	(-)
**GXP_1403228** [GXP_1403228] (1 - 602)<br>**Dnajc7**, GXL_97248, GeneID: 303536, Rattus norvegicus chr. 10<br>DnaJ (Hsp40) homolog, subfamily C, member 7	EGRF-SP1F	454 - 474	(+)
**GXP_115992** [GXP_115992] (1 - 818)<br>**Sfrs2**, GXL_97249, GeneID: 494445, Rattus norvegicus chr. 10<br>similar to splicing factor, arginine/serine-rich 2	EGRF-SP1F	130 - 107	(-)
**GXP_116006** [GXP_116006] (1 - 858)<br>**Snx11**, GXL_97263, GeneID: 303493, Rattus norvegicus chr. 10<br>sorting nexin 11	EGRF-SP1F	843 - 823	(-)
**GXP_1403327** [GXP_1403327] (1 - 694)<br>**Arhgap27**, GXL_97280, GeneID: 303583, Rattus norvegicus chr. 10<br>Rho GTPase activating protein 27	EGRF-SP1F	310 - 333	(+)

Sequence	Matrix	Position	Strand
**GXP_116034** [GXP_116034] (1 - 674)<br>**Scpep1**, GXL_97290, GeneID: 114861, Rattus norvegicus chr. 10<br>serine carboxypeptidase 1	EGRF-SP1F	480 - 503	(+)
	EGRF-SP1F	491 - 514	(+)
**GXP_116057** [GXP_116057] (1 - 643)<br>**Sost**, GXL_97313, GeneID: 80722, Rattus norvegicus chr. 10<br>sclerostin	EGRF-SP1F	265 - 289	(+)
**GXP_116064** [GXP_116064] (1 - 709)<br>**Prm2**, GXL_97320, GeneID: 25345, Rattus norvegicus chr. 10<br>protamine 2	EGRF-SP1F	483 - 507	(+)
**GXP_116066** [GXP_116066] (1 - 956)<br>**Cltc**, GXL_97322, GeneID: 54241, Rattus norvegicus chr. 10<br>clathrin, heavy polypeptide (Hc)	EGRF-SP1F	157 - 135	(-)
**GXP_116071** [GXP_116071] (1 - 601)<br>**Cacnb1**, GXL_97327, GeneID: 50688, Rattus norvegicus chr. 10<br>calcium channel, voltage-dependent, beta 1 subunit	EGRF-SP1F	323 - 300	(-)
	EGRF-SP1F	329 - 305	(-)
	EGRF-SP1F	340 - 317	(-)
	EGRF-SP1F	370 - 349	(-)
**GXP_116101** [GXP_116101] (1 - 601)<br>**Abca5**, GXL_97357, GeneID: 286970, Rattus norvegicus chr. 10<br>ATP-binding cassette, sub-family A (ABC1), member 5	EGRF-SP1F	291 - 268	(-)
**GXP_1403423** [GXP_1403423] (1 - 699)<br>**Abca5**, GXL_97357, GeneID: 286970, Rattus norvegicus chr. 10<br>ATP-binding cassette, sub-family A (ABC1), member 5	EGRF-SP1F	496 - 473	(-)
**GXP_1403370** [GXP_1403370] (1 - 762)<br>**Smarcd2**, GXL_97365, GeneID: 83833, Rattus norvegicus chr. 10<br>SWI/SNF related, matrix associated, actin dependent regulator of chromatin, subfamily d, member 2	EGRF-SP1F	102 - 82	(-)
	EGRF-SP1F	515 - 535	(+)
**GXP_1146883** [GXP_1146883] (1 - 650)<br>**Emp2**, GXL_97367, GeneID: 360468, Rattus norvegicus chr. 10<br>epithelial membrane protein 2	EGRF-SP1F	206 - 226	(+)
**GXP_116130** [GXP_116130] (1 - 846)<br>**Zfp403**, GXL_97386, GeneID: 360584, Rattus norvegicus chr. 10<br>zinc finger protein 403	EGRF-SP1F	14 - 36	(+)
	EGRF-SP1F	436 - 414	(-)
	EGRF-SP1F	440 - 418	(-)
	EGRF-SP1F	453 - 431	(-)
	EGRF-SP1F	558 - 578	(+)
**GXP_1402974** [GXP_1402974] (1 - 683)<br>**Zfp403**, GXL_97386, GeneID: 360584, Rattus norvegicus chr. 10<br>zinc finger protein 403	EGRF-SP1F	517 - 541	(+)
	EGRF-SP1F	591 - 613	(+)
**GXP_116162** [GXP_116162] (1 - 601)<br>**Grb2**, GXL_97418, GeneID: 81504, Rattus norvegicus chr. 10<br>growth factor receptor bound protein 2	EGRF-SP1F	345 - 323	(-)

**GXP_1403481** [GXP_1403481] (1 - 601)<br>**Grb2**, GXL_97418, GeneID: 81504, Rattus norvegicus chr. 10<br>growth factor receptor bound protein 2	EGRF-SP1F	300 - 280	(-)
**GXP_116168** [GXP_116168] (1 - 649)<br>**Mafg**, GXL_97424, GeneID: 64188, Rattus norvegicus chr. 10<br>v-maf musculoaponeurotic fibrosarcoma oncogene family, protein G (avian)	EGRF-SP1F	69 - 47	(-)
	EGRF-SP1F	72 - 52	(-)
**GXP_116174** [GXP_116174] (1 - 959)<br>**Hcrt**, GXL_97430, GeneID: 25723, Rattus norvegicus chr. 10<br>hypocretin	EGRF-SP1F	703 - 723	(+)
**GXP_116179** [GXP_116179] (1 - 601)<br>**Ubtf**, GXL_97435, GeneID: 25574, Rattus norvegicus chr. 10<br>upstream binding transcription factor, RNA polymerase I	EGRF-SP1F	578 - 557	(-)
**GXP_1148410** [GXP_1148410] (1 - 1302)<br>**Ubtf**, GXL_97435, GeneID: 25574, Rattus norvegicus chr. 10<br>upstream binding transcription factor, RNA polymerase I	EGRF-SP1F	542 - 563	(+)
	EGRF-SP1F	548 - 568	(+)
**GXP_1148413** [GXP_1148413] (1 - 899)<br>**Ubtf**, GXL_97435, GeneID: 25574, Rattus norvegicus chr. 10<br>upstream binding transcription factor, RNA polymerase I	EGRF-SP1F	75 - 54	(-)
**GXP_1148414** [GXP_1148414] (1 - 601)<br>**Ubtf**, GXL_97435, GeneID: 25574, Rattus norvegicus chr. 10<br>upstream binding transcription factor, RNA polymerase I	EGRF-SP1F	170 - 190	(+)
	EGRF-SP1F	339 - 318	(-)
**GXP_116197** [GXP_116197] (1 - 1476)<br>**Kpnb1**, GXL_97453, GeneID: 24917, Rattus norvegicus chr. 10<br>karyopherin (importin) beta 1	EGRF-SP1F	730 - 750	(+)
	EGRF-SP1F	732 - 755	(+)
	EGRF-SP1F	737 - 760	(+)
	EGRF-SP1F	954 - 932	(-)
	EGRF-SP1F	1165 - 1185	(+)
**GXP_116227** [GXP_116227] (1 - 1085)<br>**Nptx1**, GXL_97482, GeneID: 266777, Rattus norvegicus chr. 10<br>neuronal pentraxin 1	EGRF-SP1F	390 - 366	(-)
**GXP_116233** [GXP_116233] (1 - 936)<br>**Nxph3**, GXL_97488, GeneID: 59315, Rattus norvegicus chr. 10<br>neurexophilin 3	EGRF-SP1F	461 - 441	(-)
	EGRF-SP1F	682 - 661	(-)
	EGRF-SP1F	808 - 787	(-)
**GXP_116235** [GXP_116235] (1 - 918)<br>**Npepps**, GXL_97490, GeneID: 50558, Rattus norvegicus chr. 10<br>aminopeptidase puromycin sensitive	EGRF-SP1F	453 - 474	(+)
	EGRF-SP1F	462 - 484	(+)
	EGRF-SP1F	638 - 662	(+)
**GXP_116248** [GXP_116248] (1 - 729)<br>**Snip**, GXL_97503, GeneID: 56029, Rattus norvegicus chr. 10<br>SNAP25-interacting protein	EGRF-SP1F	430 - 408	(-)
	EGRF-SP1F	444 - 464	(+)

**GXP_1403150** [GXP_1403150] (1 - 601)<br>**Snip**, GXL_97503, GeneID: 56029, Rattus norvegicus chr. 10<br>SNAP25-interacting protein	EGRF-SP1F	344 - 322	(-)
**GXP_116249** [GXP_116249] (1 - 1210)<br>**Rpl23**, GXL_97504, GeneID: 29282, Rattus norvegicus chr. 10<br>ribosomal protein L23	EGRF-SP1F	849 - 825	(-)
**GXP_1403161** [GXP_1403161] (1 - 602)<br>**Rpl23**, GXL_97504, GeneID: 29282, Rattus norvegicus chr. 10<br>ribosomal protein L23	EGRF-SP1F	597 - 573	(-)
**GXP_116253** [GXP_116253] (1 - 1087)<br>**Cacna1g**, GXL_97508, GeneID: 29717, Rattus norvegicus chr. 10<br>calcium channel, voltage-dependent, T type, alpha 1G subunit	EGRF-SP1F	353 - 377	(+)
	EGRF-SP1F	476 - 496	(+)
	EGRF-SP1F	480 - 502	(+)
**GXP_116256** [GXP_116256] (1 - 602)<br>**Grin2a**, GXL_97511, GeneID: 24409, Rattus norvegicus chr. 10<br>glutamate receptor, ionotropic, N-methyl D-aspartate 2A	EGRF-SP1F	148 - 169	(+)
	EGRF-SP1F	150 - 171	(+)
**GXP_116262** [GXP_116262] (1 - 886)<br>**Atp5g1**, GXL_97517, GeneID: 29754, Rattus norvegicus chr. 10<br>ATP synthase, H+ transporting, mitochondrial F0 complex, subunit c (subunit 9), isoform 1	EGRF-SP1F	828 - 808	(-)
**GXP_1148172** [GXP_1148172] (1 - 601)<br>**Atp5g1**, GXL_97517, GeneID: 29754, Rattus norvegicus chr. 10<br>ATP synthase, H+ transporting, mitochondrial F0 complex, subunit c (subunit 9), isoform 1	EGRF-SP1F	511 - 487	(-)
**GXP_1403113** [GXP_1403113] (1 - 655)<br>**Atp5g1**, GXL_97517, GeneID: 29754, Rattus norvegicus chr. 10<br>ATP synthase, H+ transporting, mitochondrial F0 complex, subunit c (subunit 9), isoform 1	EGRF-SP1F	34 - 14	(-)
**GXP_116265** [GXP_116265] (1 - 981)<br>**Igf2bp1**, GXL_97520, GeneID: 303477, Rattus norvegicus chr. 10<br>insulin-like growth factor 2, binding protein 1	EGRF-SP1F	286 - 263	(-)
	EGRF-SP1F	815 - 837	(+)
**GXP_1403225** [GXP_1403225] (1 - 619)<br>**Acly**, GXL_97531, GeneID: 24159, Rattus norvegicus chr. 10<br>ATP citrate lyase	EGRF-SP1F	71 - 91	(+)
**GXP_1402365** [GXP_1402365] (1 - 602)<br>**Gabrb2**, GXL_97537, GeneID: 25451, Rattus norvegicus chr. 10<br>gamma-aminobutyric acid (GABA-A) receptor, subunit beta 2	EGRF-SP1F	428 - 451	(+)
**GXP_116285** [GXP_116285] (1 - 1258)<br>**H3f3b**, GXL_97540, GeneID: 117056, Rattus norvegicus chr. 10<br>H3 histone, family 3B	EGRF-SP1F	1062 - 1040	(-)
	EGRF-SP1F	1070 - 1046	(-)
**GXP_116301** [GXP_116301] (1 - 601)<br>**Mgat1**, GXL_97556, GeneID: 81519, Rattus norvegicus chr. 10<br>mannoside acetylglucosaminyltransferase 1	EGRF-SP1F	490 - 468	(-)
	EGRF-SP1F	455 - 435	(-)

**GXP_116313** [GXP_116313] (1 - 757)<br>**Krt25d**, GXL_97568, GeneID: 360623, Rattus norvegicus chr. 10<br>keratin 25D			
**GXP_1402247** [GXP_1402247] (1 - 696)<br>**Rnps1**, GXL_97579, GeneID: 287113, Rattus norvegicus chr. 10<br>ribonucleic acid binding protein S1	EGRF-SP1F	35 - 12	(-)
**GXP_116339** [GXP_116339] (1 - 1464)<br>**Pip5k2b**, GXL_97594, GeneID: 89812, Rattus norvegicus chr. 10<br>phosphatidylinositol-4-phosphate 5-kinase, type II, beta	EGRF-SP1F	1176 - 1154	(-)
	EGRF-SP1F	1337 - 1361	(+)
**GXP_116340** [GXP_116340] (1 - 684)<br>**Clcn7**, GXL_97595, GeneID: 29233, Rattus norvegicus chr. 10<br>chloride channel 7	EGRF-SP1F	48 - 68	(+)
	EGRF-SP1F	108 - 130	(+)
**GXP_116344** [GXP_116344] (1 - 937)<br>**Kcnh4**, GXL_97599, GeneID: 114032, Rattus norvegicus chr. 10<br>potassium voltage-gated channel, subfamily H (eag-related), member 4	EGRF-SP1F	651 - 628	(-)
	EGRF-SP1F	654 - 633	(-)
**GXP_116346** [GXP_116346] (1 - 707)<br>**Lkap**, GXL_97601, GeneID: 170946, Rattus norvegicus chr. 10<br>limkain b1	EGRF-SP1F	314 - 291	(-)
	EGRF-SP1F	513 - 491	(-)
	EGRF-SP1F	604 - 584	(-)
	EGRF-SP1F	608 - 586	(-)
**GXP_116367** [GXP_116367] (1 - 690)<br>**Pctp**, GXL_97622, GeneID: 29510, Rattus norvegicus chr. 10<br>phosphatidylcholine transfer protein	EGRF-SP1F	440 - 463	(+)
**GXP_116375** [GXP_116375] (1 - 880)<br>**Mrpl38**, GXL_97630, GeneID: 303685, Rattus norvegicus chr. 10<br>mitochondrial ribosomal protein L38	EGRF-SP1F	504 - 482	(-)
	EGRF-SP1F	509 - 487	(-)

Analysis terminated because more than 1000 matches were found.

A total of 1264 matches (1000 non-redundant matches) was found in 659 sequences.

Sequences searched: 4615 (3096183 bp).

Evaluation of results

Model: EGRF-SP1F

Number of input genes: 553

GO category "biological_process"

Number of genes annotated in GO: 426
Number of significant GO groups found: 90

GO group	p-value	# genes (observed)	# genes (expected)	list of genes	GeneIDs
localization	2.74207e-09	138	87.45	Arrb1, Ap2a2, Cd81, Picalm, Slc28a1, Abhd2_predicted, Pde3b, Doc2a, Gab2, Fgf15, Cdw92, Rab2, Sdcbp, Faf1, Clta, Kif1b, Clcnk1, Pdpn, Unc13b, Nrcam, Slc5a6, Foxg1, Npy, Cav2, Zfp384, Arf5, Met, Syn2, Kcnh2, Akap3, Cxcl12, Reln, Slc6a11, Slc4a5, Abp1, Crabp2, Ppp3ca, Accn5, Tspan2, Syt6, Pitx2, Unc5c, Kcnd3, Plcg1, Slc32a1, Src, Arfgef2, Dnmt3b, Psap, Cd24, Gja1, Gyltl1b, Atp5g3, Kcnh7, Cacnb4, Arl5a, Grin1, Cacna1b, Stxbp1, Slc34a3, Acvr1, Tra1_predicted, Hcn2, Ywhaz, Fstl3, Dgat1, Kcnq3, Rab21, Ascl1, Capzb, Slc2a5, Rims3, Nppb, Grik3, Atp2a2, Dynll1, Erp29, Ptpn11, Trpv4, Vps33a, Alcam, Slc12a8, Gsk3b, Clcn2, Ap2m1, Clic6, Kcne1, Cd47, Lphn1, Cd97, Bcar1, Gnao, Vps4a, Pou4f2, Glg1, Mbtps1, Slc7a5, Tomm20, Bcap31, Cacna1f, Gdi1, Tsc22d3, L1cam, Slc7a3, Atp6ap1, Slc38a5, Slc25a1, Dnai2, Slc16a6, Vegfc, Myo9b, Cacna2d3, Arf4, Slc27a1, Vps36_predicted, Rab3a, Edn1, Kif27, Bicd2, Inhba, Exoc2, Kcnmb1, Cygb, Cacng4, Arhgap27, Cltc, Cacnb1, Abca5, Hcrt, Kpnb1, Nptx1, Snip, Rpl23, Cacna1g, Igf2bp1, Gabrb2, Kcnh4, Pctp	25387, 81637, 25621, 89816, 116642, 293050, 29516, 65031, 84477, 170582, 85254, 65158, 83841, 140657, 83800, 117548, 79425, 54320, 64830, 497815, 170551, 24370, 24604, 363425, 171018, 79117, 24553, 29179, 117018, 312720, 24772, 24718, 79213, 297386, 65029, 29563, 24674, 63866, 64521, 60565, 54284, 362049, 65195, 25738, 83612, 83805, 296380, 444985, 25524, 25145, 24392, 311202, 114630, 170739, 58942, 117050, 24408, 257648, 25558, 246234, 79558, 362862, 114244, 25578, 114031, 84497, 29682, 299799, 64186, 298584, 65197, 65025, 25105, 298521, 29693, 58945, 117030, 25622, 66026, 65081, 79559, 266733, 84027, 29232, 116563, 304081, 25471, 29364, 65096, 361383, 25414, 50664, 246772, 171355, 29476, 89842, 50719, 266601, 293852, 114493, 25183, 83514, 50687, 29485, 83615, 192208, 29743, 360654, 303772, 114111, 25486, 306243, 79120, 94172, 290851, 25531, 24323, 246209, 306809, 29200, 171455, 29747, 170520, 140725, 303583, 54241, 50688, 286970, 25723, 24917, 266777, 56029, 29282, 29717, 303477, 25451, 114032, 29510
neuron differentiation	2.11756e-07	34	12.96	Igf1r, Pak1, Nrcam, Foxg1, Cxcl12, Reln, S100a6, Unc5c, Ss18l1, Gja1, Stxbp1, Rtn4rl2, Notch3, Socs2, Ascl1, Id3, Ptpn11, Mbp, Alcam, Dgkg, Ncam2, Rtn4r, Gnao, Pou4f2, L1cam, Lzts1, Fgfr1, Casp3, Nr2f6, Neurod3, Id4, Dbn1, Timp2, Neurod2	25718, 29431, 497815, 24370, 24772, 24718, 85247, 362049, 192352, 24392, 25558, 311169, 56761, 84607, 64186, 25585, 25622, 24547, 79559, 25666, 288280, 113912, 50664, 171355, 50687, 266711, 79114, 25402, 245980, 29410, 291023, 81653, 29543, 54276
establishment of localization	4.25185e-07	112	72.18	Arrb1, Ap2a2, Picalm, Slc28a1, Pde3b, Doc2a, Cdw92, Rab2, Sdcbp, Faf1, Clta, Kif1b, Clcnk1, Unc13b, Slc5a6, Npy, Cav2, Zfp384, Arf5, Syn2, Kcnh2, Slc6a11, Slc4a5, Abp1, Crabp2, Ppp3ca, Accn5, Syt6, Kcnd3, Plcg1, Slc32a1, Src, Arfgef2, Psap, Cd24, Gja1, Gyltl1b, Atp5g3, Kcnh7, Cacnb4, Arl5a, Grin1, Cacna1b, Stxbp1, Slc34a3, Tra1_predicted, Hcn2, Ywhaz, Fstl3, Dgat1, Kcnq3, Rab21, Slc2a5, Rims3, Nppb, Grik3, Atp2a2, Dynll1, Erp29, Ptpn11, Trpv4, Vps33a, Slc12a8, Gsk3b, Clcn2, Ap2m1, Clic6, Kcne1, Cd47, Lphn1, Vps4a, Glg1, Mbtps1, Slc7a5, Tomm20, Bcap31, Cacna1f, Gdi1, Tsc22d3, Slc7a3, Atp6ap1, Slc38a5, Slc25a1, Slc16a6, Vegfc, Myo9b, Cacna2d3, Arf4,	25387, 81637, 89816, 116642, 29516, 65031, 85254, 65158, 83841, 140657, 83800, 117548, 79425, 64830, 170551, 24604, 363425, 171018, 79117, 29179, 117018, 79213, 297386, 65029, 29563, 24674, 63866, 60565, 65195, 25738, 83612, 83805, 296380, 25524, 25145, 24392, 311202, 114630, 170739, 58942, 117050, 24408, 257648, 25558, 246234, 362862, 114244, 25578, 114031, 84497, 29682, 299799, 65197, 65025, 25105, 298521, 29693, 58945, 117030, 25622, 66026, 65081, 266733, 84027, 29232, 116563, 304081, 25471, 29364, 65096, 246772, 29476, 89842, 50719, 266601, 293852,

				Slc27a1, Vps36_predicted, Rab3a, Edn1, Kif27, Bicd2, Inhba, Exoc2, Kcnmb1, Cygb, Cacng4, Arhgap27, Cltc, Cacnb1, Abca5, Hcrt, Kpnb1, Nptx1, Snip, Rpl23, Cacna1g, Gabrb2, Kcnh4, Pctp	114493, 25183, 83514, 29485, 83615, 192208, 29743, 303772, 114111, 25486, 306243, 79120, 94172, 290851, 25531, 24323, 246209, 306809, 29200, 171455, 29747, 170520, 140725, 303583, 54241, 50688, 286970, 25723, 24917, 266777, 56029, 29282, 29717, 25451, 114032, 29510
transport	1.31818e-06	106	68.72	Arrb1, Ap2a2, Picalm, Slc28a1, Pde3b, Doc2a, Cdw92, Rab2, Sdcbp, Faf1, Clta, Kif1b, Unc13b, Slc5a6, Npy, Cav2, Zfp384, Arf5, Syn2, Kcnh2, Slc6a11, Slc4a5, Abp1, Crabp2, Ppp3ca, Accn5, Syt6, Kcnd3, Plcg1, Slc32a1, Src, Arfgef2, Psap, Cd24, Gja1, Gyltl1b, Atp5g3, Kcnh7, Cacnb4, Arl5a, Grin1, Cacna1b, Stxbp1, Slc34a3, Tra1_predicted, Hcn2, Ywhaz, Fstl3, Dgat1, Kcnq3, Rab21, Slc2a5, Rims3, Grik3, Atp2a2, Dynll1, Erp29, Ptpn11, Trpv4, Vps33a, Slc12a8, Gsk3b, Clcn2, Ap2m1, Clic6, Kcne1, Cd47, Lphn1, Vps4a, Glg1, Mbtps1, Slc7a5, Tomm20, Bcap31, Cacna1f, Gdi1, Slc7a3, Atp6ap1, Slc38a5, Slc25a1, Slc16a6, Myo9b, Cacna2d3, Arf4, Slc27a1, Vps36_predicted, Rab3a, Edn1, Kif27, Bicd2, Exoc2, Kcnmb1, Cygb, Cacng4, Arhgap27, Cltc, Cacnb1, Abca5, Hcrt, Kpnb1, Nptx1, Rpl23, Cacna1g, Gabrb2, Kcnh4, Pctp	25387, 81637, 89816, 116642, 29516, 65031, 85254, 65158, 83841, 140657, 83800, 117548, 64830, 170551, 24604, 363425, 171018, 79117, 29179, 117018, 79213, 297386, 65029, 29563, 24674, 63866, 60565, 65195, 25738, 83612, 83805, 296380, 25524, 25145, 24392, 311202, 114630, 170739, 58942, 117050, 24408, 257648, 25558, 246234, 362862, 114244, 25578, 114031, 84497, 29682, 299799, 65197, 65025, 298521, 29693, 58945, 117030, 25622, 66026, 65081, 266733, 84027, 29232, 116563, 304081, 25471, 29364, 65096, 246772, 29476, 89842, 50719, 266601, 293852, 114493, 25183, 29485, 83615, 192208, 29743, 303772, 25486, 306243, 79120, 94172, 290851, 25531, 24323, 246209, 306809, 171455, 29747, 170520, 140725, 303583, 54241, 50688, 286970, 25723, 24917, 266777, 29282, 29717, 25451, 114032, 29510
generation of neurons	1.83036e-06	35	14.85	Igf1r, Pak1, Nrcam, Foxg1, Met, Cxcl12, Reln, S100a6, Unc5c, Ss18l1, Gja1, Stxbp1, Rtn4rl2, Notch3, Socs2, Ascl1, Id3, Ptpn11, Mbp, Alcam, Dgkg, Ncam2, Rtn4r, Gnao, Pou4f2, L1cam, Lzts1, Fgfr1, Casp3, Nr2f6, Neurod3, Id4, Dbn1, Timp2, Neurod2	25718, 29431, 497815, 24370, 24553, 24772, 24718, 85247, 362049, 192352, 24392, 25558, 311169, 56761, 84607, 64186, 25585, 25622, 24547, 79559, 25666, 288280, 113912, 50664, 171355, 50687, 266711, 79114, 25402, 245980, 29410, 291023, 81653, 29543, 54276
cell differentiation	1.86495e-06	94	59.22	Fgf3, Igf1r, Tbx6_predicted, Pak1, Bag3, Mgmt, Fgf15, Faf1, Tmod1, Tnfrsf1b, Unc13b, Nrcam, Foxg1, Chn2, Met, Casp2, Cxcl12, Pparg, Reln, Foxm1, Bcl2l13_predicted, Gclm, S100a10, S100a6, Tspan2, Unc5c, Sep15, Notch2, Tcfap2c, Ss18l1, Src, Gja1, Neurog3, Lcn2, Stxbp1, Rtn4rl2, Acvr1, Dlx2, Aes, Zbtb7a, Cand1, Plec1, Tra1_predicted, Notch3, Socs2, Ascl1, Id3, Pou3f1, Wnt4, Pafah2, Casp9, Diablo, Ptpn11, Vps33a, Lnk, Mbp, Dscr1, Alcam, Dgkg, Ncam2, Gsk3b, Kcne1, Rtn4r, Tradd, Bcar1, Gnao, Pou4f2, Timp1, Sh3kbp1, Birc4, Tsc22d3, L1cam, Axin2, Prkcd, Bmpr1a, Lzts1, Ppp2cb, Fgfr1, Casp3, Sh3md2, Nr2f6, Edn1, Tcfap2a_predicted, Smad5, Barx1_predicted, Neurod3, Inhba, Id4, Dbn1, Timp2, Neurod2, Axin1, Prm2, Mafg	170633, 25718, 365371, 29431, 293524, 25332, 170582, 140657, 25566, 156767, 64830, 497815, 24370, 84031, 24553, 64314, 24772, 25664, 24718, 58921, 312682, 29739, 81778, 85247, 64521, 362049, 113922, 29492, 362280, 192352, 83805, 24392, 60329, 170496, 25558, 311169, 79558, 296499, 29466, 117107, 117152, 64204, 362862, 56761, 84607, 64186, 25585, 192110, 84426, 313611, 58918, 288753, 25622, 65081, 58838, 24547, 266766, 79559, 25666, 288280, 84027, 25471, 113912, 246756, 25414, 50664, 171355, 116510, 84357, 63879, 83514, 50687, 29134, 170538, 81507, 266711, 24673, 79114, 25402, 306417, 245980, 24323, 306862, 59328, 364680, 29410, 29200, 291023, 81653, 29543, 54276, 79257, 25345, 64188
cellular developmental	1.86495e-06	94	59.22	Fgf3, Igf1r, Tbx6_predicted, Pak1, Bag3, Mgmt, Fgf15, Faf1, Tmod1, Tnfrsf1b, Unc13b, Nrcam, Foxg1, Chn2, Met, Casp2,	170633, 25718, 365371, 29431, 293524, 25332, 170582, 140657, 25566, 156767, 64830, 497815, 24370, 84031,

process				Cxcl12, Pparg, Reln, Foxm1, Bcl2l13_predicted, Gclm, S100a10, S100a6, Tspan2, Unc5c, Sep15, Notch2, Tcfap2c, Ss18l1, Src, Gja1, Neurog3, Lcn2, Stxbp1, Rtn4rl2, Acvr1, Dlx2, Aes, Zbtb7a, Cand1, Plec1, Tra1_predicted, Notch3, Socs2, Ascl1, Id3, Pou3f1, Wnt4, Pafah2, Casp9, Diablo, Ptpn11, Vps33a, Lnk, Mbp, Dscr1, Alcam, Dgkg, Ncam2, Gsk3b, Kcne1, Rtn4r, Tradd, Bcar1, Gnao, Pou4f2, Timp1, Sh3kbp1, Birc4, Tsc22d3, L1cam, Axin2, Prkcd, Bmpr1a, Lzts1, Ppp2cb, Fgfr1, Casp3, Sh3md2, Nr2f6, Edn1, Tcfap2a_predicted, Smad5, Barx1_predicted, Neurod3, Inhba, Id4, Dbn1, Timp2, Neurod2, Axin1, Prm2, Mafg	24553, 64314, 24772, 25664, 24718, 58921, 312682, 29739, 81778, 85247, 64521, 362049, 113922, 29492, 362280, 192352, 83805, 24392, 60329, 170496, 25558, 311169, 79558, 296499, 29466, 117107, 117152, 64204, 362862, 56761, 84607, 64186, 25585, 192110, 84426, 313611, 58918, 288753, 25622, 65081, 58838, 24547, 266766, 79559, 25666, 288280, 84027, 25471, 113912, 246756, 25414, 50664, 171355, 116510, 84357, 63879, 83514, 50687, 29134, 170538, 81507, 266711, 24673, 79114, 25402, 306417, 245980, 24323, 306862, 59328, 364680, 29410, 29200, 291023, 81653, 29543, 54276, 79257, 25345, 64188
neurogenesis	2.06607e-06	37	16.24	Igf1r, Pak1, Nrcam, Foxg1, Met, Cxcl12, Reln, S100a6, Tspan2, Unc5c, Ss18l1, Gja1, Stxbp1, Rtn4rl2, Notch3, Socs2, Ascl1, Id3, Pou3f1, Ptpn11, Mbp, Alcam, Dgkg, Ncam2, Rtn4r, Gnao, Pou4f2, L1cam, Lzts1, Fgfr1, Casp3, Nr2f6, Neurod3, Id4, Dbn1, Timp2, Neurod2	25718, 29431, 497815, 24370, 24553, 24772, 24718, 85247, 64521, 362049, 192352, 24392, 25558, 311169, 56761, 84607, 64186, 25585, 192110, 25622, 24547, 79559, 25666, 288280, 113912, 50664, 171355, 50687, 266711, 79114, 25402, 245980, 29410, 291023, 81653, 29543, 54276
neuron development	4.21799e-06	26	9.73	Igf1r, Pak1, Nrcam, Foxg1, Cxcl12, Reln, S100a6, Unc5c, Ss18l1, Gja1, Stxbp1, Rtn4rl2, Ptpn11, Mbp, Alcam, Dgkg, Ncam2, Rtn4r, Gnao, Pou4f2, L1cam, Lzts1, Fgfr1, Nr2f6, Dbn1, Neurod2	25718, 29431, 497815, 24370, 24772, 24718, 85247, 362049, 192352, 24392, 25558, 311169, 25622, 24547, 79559, 25666, 288280, 113912, 50664, 171355, 50687, 266711, 79114, 245980, 81653, 54276
developmental process	6.09135e-06	149	108.85	Fgf3, Pde3b, Igf1r, Mesdc2, Doc2a, Agc1, Tbx6_predicted, Pak1, Bag3, Mgmt, Fgf15, Sdcbp, Faf1, Tmod1, Nr4a3, Tnfrsf1b, Ugcg, Pdpn, Unc13b, Nrcam, Crip2, Rhoj, Cgref1, Foxg1, Yy1, Chn2, Sftpb, Met, Bhlhb2, C1galt1, Casp2, Cxcl12, Pparg, Reln, Ptpn12, Foxm1, Bcl2l13_predicted, Gclm, Crabp2, Lmo4, Pdgfc, S100a10, Pde5a, S100a6, Tspan2, Pitx2, Unc5c, Sep15, Notch2, Plcg1, Tcfap2c, Ss18l1, Src, Cd164, Gja1, Neurog3, Fnbp1, Nfe2l2, Lcn2, Stxbp1, Calcrl, Atf2, Rtn4rl2, Cd44, Acvr1, Dlx2, Aes, Zbtb7a, Matk, Cand1, Cart1, Plec1, Tra1_predicted, Notch3, Dgat1, Socs2, Ascl1, Capzb, Id3, Heyl_predicted, Pou3f1, Wnt4, Pafah2, Nppb, Nppa, Casp9, Dynll1, Diablo, Ptpn11, Vps33a, Lnk, Mbp, Dscr1, Txnrd2, Alcam, Dgkg, Ncam2, Cxadr, Gsk3b, Clcn2, Kcne1, Rtn4r, Tradd, Bcar1, Dok4_predicted, Gnao, Pou4f2, Siah1a, Timp1, Mid1, Sh3kbp1, Birc4, Morf4l2, Rbbp7, Tsc22d3, L1cam, Gpm6b, Dgcr2, Axin2, Vegfc, Prkcd, Bmpr1a, Lzts1, Ppp2cb, Fgfr1, Sncg, Casp3, Mustn1, Sh3md2, Wnt5a, Nr2f6, Edn1, Tcfap2a_predicted, Smad5, Srd5a1, Barx1_predicted, Neurod3, Inhba, Ptch1, Id4, Ninj1, Dbn1, Timp2, Neurod2, Axin1, Sost, Prm2, Mafg, Nptx1	170633, 29516, 25718, 308796, 65031, 58968, 365371, 29431, 293524, 25332, 170582, 83841, 140657, 25566, 58853, 156767, 83626, 54320, 64830, 497815, 338401, 299145, 245918, 24370, 24919, 84031, 192155, 24553, 79431, 65044, 64314, 24772, 25664, 24718, 117255, 58921, 312682, 29739, 29563, 362051, 79429, 81778, 171115, 85247, 64521, 54284, 362049, 113922, 29492, 25738, 362280, 192352, 83805, 83689, 24392, 60329, 192348, 83619, 170496, 25558, 25029, 81647, 311169, 25406, 79558, 296499, 29466, 117107, 60450, 117152, 25401, 64204, 362862, 56761, 84497, 84607, 64186, 298584, 25585, 313575, 192110, 84426, 313611, 25105, 24602, 58918, 58945, 288753, 25622, 65081, 58838, 24547, 266766, 50551, 79559, 25666, 288280, 89843, 84027, 29232, 25471, 113912, 246756, 25414, 361364, 50664, 171355, 140941, 116510, 54252, 84357, 63879, 317413, 83712, 83514, 50687, 192179, 360742, 29134, 114111, 170538, 81507, 266711, 24673, 79114, 64347, 25402, 290553, 306417, 64566, 245980, 24323, 306862, 59328, 24950, 364680, 29410, 29200, 89830, 291023, 25338, 81653, 29543, 54276, 79257, 80722, 25345,

					64188, 266777
anatomical structure morphogenesis	8.2438e-06	70	41.74	Fgf3, Igf1r, Tbx6_predicted, Pak1, Sdcbp, Nr4a3, Pdpn, Nrcam, Rhoj, Cgref1, Foxg1, Chn2, Sftpb, C1galt1, Cxcl12, Pparg, Reln, Ptpn12, Foxm1, Crabp2, Lmo4, Pdgfc, S100a10, S100a6, Pitx2, Unc5c, Notch2, Ss18l1, Gja1, Neurog3, Stxbp1, Rtn4rl2, Cd44, Aes, Plec1, Notch3, Socs2, Capzb, Id3, Wnt4, Nppb, Nppa, Dynll1, Ptpn11, Mbp, Alcam, Ncam2, Gsk3b, Kcne1, Rtn4r, Bcar1, Gnao, Pou4f2, Mid1, Morf4l2, L1cam, Dgcr2, Vegfc, Bmpr1a, Lzts1, Ppp2cb, Fgfr1, Mustn1, Wnt5a, Edn1, Barx1_predicted, Neurod3, Ptch1, Dbn1, Mafg	170633, 25718, 365371, 29431, 83841, 58853, 54320, 497815, 299145, 245918, 24370, 84031, 192155, 65044, 24772, 25664, 24718, 117255, 58921, 29563, 362051, 79429, 81778, 85247, 54284, 362049, 29492, 192352, 24392, 60329, 25558, 311169, 25406, 29466, 64204, 56761, 84607, 298584, 25585, 84426, 25105, 24602, 58945, 25622, 24547, 79559, 288280, 84027, 25471, 113912, 25414, 50664, 171355, 54252, 317413, 50687, 360742, 114111, 81507, 266711, 24673, 79114, 290553, 64566, 24323, 364680, 29410, 89830, 81653, 64188
cell development	1.12781e-05	75	46.12	Igf1r, Pak1, Bag3, Mgmt, Fgf15, Faf1, Tmod1, Tnfrsf1b, Unc13b, Nrcam, Foxg1, Chn2, Met, Casp2, Cxcl12, Reln, Bcl2l13_predicted, Gclm, S100a6, Tspan2, Unc5c, Sep15, Notch2, Ss18l1, Src, Gja1, Lcn2, Stxbp1, Rtn4rl2, Acvr1, Tra1_predicted, Notch3, Socs2, Ascl1, Id3, Pou3f1, Wnt4, Pafah2, Casp9, Diablo, Ptpn11, Mbp, Alcam, Dgkg, Ncam2, Gsk3b, Kcne1, Rtn4r, Tradd, Bcar1, Gnao, Pou4f2, Timp1, Sh3kbp1, Birc4, Tsc22d3, L1cam, Axin2, Prkcd, Lzts1, Ppp2cb, Fgfr1, Casp3, Sh3md2, Nr2f6, Edn1, Tcfap2a_predicted, Neurod3, Inhba, Id4, Dbn1, Timp2, Neurod2, Axin1, Prm2	25718, 29431, 293524, 25332, 170582, 140657, 25566, 156767, 64830, 497815, 24370, 84031, 24553, 64314, 24772, 24718, 312682, 29739, 85247, 64521, 362049, 113922, 29492, 192352, 83805, 24392, 170496, 25558, 311169, 79558, 362862, 56761, 84607, 64186, 25585, 192110, 84426, 313611, 58918, 288753, 25622, 24547, 79559, 25666, 288280, 84027, 25471, 113912, 246756, 25414, 50664, 171355, 116510, 84357, 63879, 83514, 50687, 29134, 170538, 266711, 24673, 79114, 25402, 306417, 245980, 24323, 306862, 29410, 29200, 291023, 81653, 29543, 54276, 79257, 25345
neurite development	1.30937e-05	23	8.53	Igf1r, Pak1, Nrcam, Foxg1, Cxcl12, Reln, S100a6, Unc5c, Ss18l1, Gja1, Stxbp1, Rtn4rl2, Ptpn11, Mbp, Alcam, Ncam2, Rtn4r, Gnao, Pou4f2, L1cam, Lzts1, Fgfr1, Dbn1	25718, 29431, 497815, 24370, 24772, 24718, 85247, 362049, 192352, 24392, 25558, 311169, 25622, 24547, 79559, 288280, 113912, 50664, 171355, 50687, 266711, 79114, 81653
anatomical structure development	1.42885e-05	117	81.87	Fgf3, Pde3b, Igf1r, Mesdc2, Doc2a, Agc1, Tbx6_predicted, Pak1, Fgf15, Sdcbp, Tmod1, Nr4a3, Ugcg, Pdpn, Nrcam, Crip2, Rhoj, Cgref1, Foxg1, Chn2, Sftpb, Met, Bhlhb2, C1galt1, Cxcl12, Pparg, Reln, Ptpn12, Foxm1, Crabp2, Lmo4, Pdgfc, S100a10, Pde5a, S100a6, Tspan2, Pitx2, Unc5c, Notch2, Ss18l1, Src, Gja1, Neurog3, Fnbp1, Stxbp1, Calcrl, Atf2, Rtn4rl2, Cd44, Acvr1, Dlx2, Aes, Zbtb7a, Matk, Cart1, Plec1, Notch3, Dgat1, Socs2, Ascl1, Capzb, Id3, Heyl_predicted, Pou3f1, Wnt4, Nppb, Nppa, Dynll1, Ptpn11, Vps33a, Lnk, Mbp, Dscr1, Txnrd2, Alcam, Dgkg, Ncam2, Cxadr, Gsk3b, Clcn2, Kcne1, Rtn4r, Bcar1, Dok4_predicted, Gnao, Pou4f2, Mid1, Morf4l2, L1cam, Gpm6b, Dgcr2, Axin2, Vegfc, Bmpr1a, Lzts1, Ppp2cb, Fgfr1, Sncg, Casp3, Mustn1, Wnt5a, Nr2f6, Edn1, Tcfap2a_predicted, Smad5, Barx1_predicted, Neurod3, Inhba, Ptch1, Id4, Ninj1, Dbn1, Timp2, Neurod2, Sost, Mafg, Nptx1	170633, 29516, 25718, 308796, 65031, 58968, 365371, 29431, 170582, 83841, 25566, 58853, 83626, 54320, 497815, 338401, 299145, 245918, 24370, 84031, 192155, 24553, 79431, 65044, 24772, 25664, 24718, 117255, 58921, 29563, 362051, 79429, 81778, 171115, 85247, 64521, 54284, 362049, 29492, 192352, 83805, 24392, 60329, 192348, 25558, 25029, 81647, 311169, 25406, 79558, 296499, 29466, 117107, 60450, 25401, 64204, 56761, 84497, 84607, 64186, 298584, 25585, 313575, 192110, 84426, 25105, 24602, 58945, 25622, 65081, 58838, 24547, 266766, 50551, 79559, 25666, 288280, 89843, 84027, 29232, 25471, 113912, 25414, 361364, 50664, 171355, 54252, 317413, 50687, 192179, 360742, 29134, 114111, 81507, 266711, 24673, 79114, 64347, 25402, 290553, 64566, 245980, 24323, 306862, 59328, 364680, 29410, 29200, 89830, 291023, 25338, 81653, 29543, 54276, 80722, 64188, 266777

nervous system development	2.01446e-05	56	31.87	Igf1r, Doc2a, Agc1, Pak1, Nrcam, Foxg1, Met, Bhlhb2, Cxcl12, Reln, Lmo4, Pde5a, S100a6, Tspan2, Unc5c, Notch2, Ss18l1, Src, Gja1, Fnbp1, Stxbp1, Atf2, Rtn4rl2, Dlx2, Cart1, Notch3, Socs2, Ascl1, Id3, Heyl_predicted, Pou3f1, Ptpn11, Mbp, Dscr1, Alcam, Dgkg, Ncam2, Rtn4r, Dok4_predicted, Gnao, Pou4f2, L1cam, Gpm6b, Lzts1, Fgfr1, Sncg, Casp3, Nr2f6, Neurod3, Inhba, Id4, Ninj1, Dbn1, Timp2, Neurod2, Nptx1	25718, 65031, 58968, 29431, 497815, 24370, 24553, 79431, 24772, 24718, 362051, 171115, 85247, 64521, 362049, 29492, 192352, 83805, 24392, 192348, 25558, 81647, 311169, 296499, 25401, 56761, 84607, 64186, 25585, 313575, 192110, 25622, 24547, 266766, 79559, 25666, 288280, 113912, 361364, 50664, 171355, 50687, 192179, 266711, 79114, 64347, 25402, 245980, 29410, 29200, 291023, 25338, 81653, 29543, 54276, 266777
cell morphogenesis	2.16899e-05	38	18.68	Igf1r, Pak1, Sdcbp, Nrcam, Rhoj, Cgref1, Foxg1, Cxcl12, Reln, S100a10, S100a6, Unc5c, Notch2, Ss18l1, Gja1, Stxbp1, Rtn4rl2, Cd44, Socs2, Capzb, Nppb, Nppa, Dynll1, Ptpn11, Mbp, Alcam, Ncam2, Gsk3b, Rtn4r, Bcar1, Gnao, Pou4f2, Morf4l2, L1cam, Lzts1, Ppp2cb, Fgfr1, Dbn1	25718, 29431, 83841, 497815, 299145, 245918, 24370, 24772, 24718, 81778, 85247, 362049, 29492, 192352, 24392, 25558, 311169, 25406, 84607, 298584, 25105, 24602, 58945, 25622, 24547, 79559, 288280, 84027, 113912, 25414, 50664, 171355, 317413, 50687, 266711, 24673, 79114, 81653
cell-cell signaling	3.0283e-05	51	28.50	Fgf3, Pde3b, Doc2a, Sdcbp, Gabrd, Unc13b, Nrcam, Npy, Slc6a1, Cav2, Bhlhb2, Syn2, S100a6, A, Cd24, Gja1, Grin1, Cacna1b, Lynx1_predicted, Hcn2, Dgat1, Kcnq3, Pou3f1, Wnt4, Grik3, Trpv4, Mbp, Fgf12, Lphn1, Cd97, Hsd11b2, Cacna1f, Sh3kbp1, Gabra3, Lzts1, Fgfr1, Wnt5a, Rab3a, Edn1, Srd5a1, Sncb, Barx1_predicted, Inhba, Dbn1, Kcnmb1, Cacng4, Grb2, Hcrt, Nptx1, Grin2a, Gabrb2	170633, 29516, 65031, 83841, 29689, 64830, 497815, 24604, 79212, 363425, 79431, 29179, 85247, 24152, 25145, 24392, 24408, 257648, 300018, 114244, 84497, 29682, 192110, 84426, 298521, 66026, 24547, 170630, 65096, 361383, 25117, 114493, 84357, 24947, 266711, 79114, 64566, 25531, 24323, 24950, 113893, 364680, 29200, 81653, 29747, 140725, 81504, 25723, 266777, 24409, 25451
regulation of developmental process	3.40856e-05	26	10.93	Nrcam, Rhoj, Foxg1, Pparg, S100a10, Notch2, Nfe2l2, Zbtb7a, Notch3, Socs2, Id3, Pou3f1, Wnt4, Dynll1, Vps33a, Mbp, Dscr1, Rtn4r, Axin2, Lzts1, Ppp2cb, Inhba, Id4, Dbn1, Timp2, Mafg	497815, 299145, 24370, 25664, 81778, 29492, 83619, 117107, 56761, 84607, 25585, 192110, 84426, 58945, 65081, 24547, 266766, 113912, 29134, 266711, 24673, 29200, 291023, 81653, 29543, 64188
biological regulation	4.36921e-05	162	124.72	Arrb1, Cd81, Abhd2_predicted, Pde3b, Igf1r, Doc2a, Tbx6_predicted, Bag3, Mgmt, Ncoa2, Rab2, Faf1, Anp32b, Tnfrsf1b, Nfia, Pdpn, Unc13b, Nrcam, Crip2, Rhoj, Cgref1, Foxg1, Ppm1a, Yy1, Sfrs5, Adcy3, Cav2, Prkag2, Met, Bhlhb2, Cyp51, Syn2, Casp2, Kcnh2, Cxcl12, Pparg, Reln, Foxm1, Bcl2l13_predicted, Prkab2, Gclm, Cxxc4, Crabp2, Pdgfc, S100a10, Pde5a, S100a6, Pitx2, Unc5c, Notch2, Pofut1, Tcfap2c, Hnf4a, Src, Dnmt3b, Cd164, Gja1, Neurog3, Nfe2l2, Kcnh7, Cdk9, Grin1, Cacna1b, Calcrl, Serping1, Cd44, Dlx2, Aes, Hrsp12, Zbtb7a, Matk, Cand1, Cart1, Tra1_predicted, Tmpo, Fstl3, Notch3, Tdg, Yeats4_predicted, Socs2, Ascl1, Cnot2, Capzb, Id3, Heyl_predicted, Pou3f1, Mad2l2, Wnt4, Pafah2, Nppb, Nppa, Casp9, Atp2a2, Dynll1, Tcf1, Diablo, Ptpn11, Eif2b1, Trpv4, Vps33a, Mbp, Dscr1, Masp1, Dgkg, Tnk2, Gsk3b, Kcne1, Cd47, Rtn4r, Lphn1, Slc12a4, Ctcf, Bcar1, Gnao, Pou4f2, Mbtps1, Maf, Pkn1, Dnaja2, Timp1, Cacna1f, Mid1, Sh3kbp1, Birc4, Maged1, Morf4l2, Rbbp7, Mig12, Tsc22d3, Apln, Axin2, Vegfc, Prkcd,	25387, 25621, 293050, 29516, 25718, 65031, 365371, 293524, 25332, 83724, 65158, 140657, 170724, 156767, 25492, 54320, 64830, 497815, 338401, 299145, 245918, 24370, 24666, 24919, 29667, 64508, 363425, 373545, 24553, 79431, 25427, 29179, 64314, 117018, 24772, 25664, 24718, 58921, 312682, 64562, 29739, 83824, 29563, 79429, 81778, 171115, 85247, 54284, 362049, 29492, 311551, 362280, 25735, 83805, 444985, 83689, 24392, 60329, 83619, 170739, 362110, 24408, 257648, 25029, 295703, 25406, 296499, 29466, 65151, 117107, 60450, 117152, 25401, 362862, 25359, 114031, 56761, 114521, 299810, 84607, 64186, 299805, 298584, 25585, 313575, 192110, 313702, 84426, 313611, 25105, 24602, 58918, 29693, 58945, 24817, 288753, 25622, 64514, 66026, 65081, 24547, 266766, 64023, 25666, 303882, 84027, 25471, 29364, 113912, 65096, 29501, 83726, 25414, 50664, 171355, 89842, 54267, 29355, 84026,

				Bmpr1a, Lzts1, Ppp2cb, Fgfr1, Casp3, Sh3md2, Rab3a, Edn1, Tcfap2a_predicted, Smad5, Sncb, Nfil3, Barx1_predicted, Neurod3, Gprk6, Inhba, Exoc2, Ptch1, Id4, Dbn1, Timp2, Neurod2, Axin1, Sost, Smarcd2, Mafg, Hcrt, Ubtf, Snip	116510, 114493, 54252, 84357, 63879, 84469, 317413, 83712, 404280, 83514, 58812, 29134, 114111, 170538, 81507, 266711, 24673, 79114, 25402, 306417, 25531, 24323, 306862, 59328, 113893, 114519, 364680, 29410, 59076, 29200, 171455, 89830, 291023, 81653, 29543, 54276, 79257, 80722, 83833, 64188, 25723, 25574, 56029
regulation of biological process	4.50759e-05	159	122.00	Arrb1, Cd81, Abhd2_predicted, Pde3b, Igf1r, Doc2a, Tbx6_predicted, Bag3, Mgmt, Ncoa2, Rab2, Faf1, Anp32b, Tnfrsf1b, Nfia, Pdpn, Unc13b, Nrcam, Crip2, Rhoj, Cgref1, Foxg1, Ppm1a, Yy1, Sfrs5, Adcy3, Cav2, Met, Bhlhb2, Cyp51, Syn2, Casp2, Kcnh2, Cxcl12, Pparg, Reln, Foxm1, Bcl2l13_predicted, Gclm, Cxxc4, Crabp2, Pdgfc, S100a10, S100a6, Pitx2, Unc5c, Notch2, Pofut1, Tcfap2c, Hnf4a, Src, Dnmt3b, Cd164, Gja1, Neurog3, Nfe2l2, Kcnh7, Cdk9, Grin1, Cacna1b, Calcrl, Serping1, Cd44, Dlx2, Aes, Hrsp12, Zbtb7a, Matk, Cand1, Cart1, Tra1_predicted, Tmpo, Fstl3, Notch3, Tdg, Yeats4_predicted, Socs2, Ascl1, Cnot2, Capzb, Id3, Heyl_predicted, Pou3f1, Mad2l2, Wnt4, Pafah2, Nppb, Nppa, Casp9, Atp2a2, Dynll1, Tcf1, Diablo, Ptpn11, Eif2b1, Trpv4, Vps33a, Mbp, Dscr1, Masp1, Dgkg, Tnk2, Gsk3b, Kcne1, Cd47, Rtn4r, Lphn1, Slc12a4, Ctcf, Bcar1, Gnao, Pou4f2, Mbtps1, Maf, Pkn1, Dnaja2, Timp1, Cacna1f, Mid1, Sh3kbp1, Birc4, Maged1, Morf4l2, Rbbp7, Mig12, Tsc22d3, Apln, Axin2, Vegfc, Prkcd, Bmpr1a, Lzts1, Ppp2cb, Fgfr1, Casp3, Sh3md2, Rab3a, Edn1, Tcfap2a_predicted, Smad5, Sncb, Nfil3, Barx1_predicted, Neurod3, Gprk6, Inhba, Exoc2, Ptch1, Id4, Dbn1, Timp2, Neurod2, Axin1, Sost, Smarcd2, Mafg, Hcrt, Ubtf, Snip	25387, 25621, 293050, 29516, 25718, 65031, 365371, 293524, 25332, 83724, 65158, 140657, 170724, 156767, 25492, 54320, 64830, 497815, 338401, 299145, 245918, 24370, 24666, 24919, 29667, 64508, 363425, 24553, 79431, 25427, 29179, 64314, 117018, 24772, 25664, 24718, 58921, 312682, 29739, 83824, 29563, 79429, 81778, 85247, 54284, 362049, 29492, 311551, 362280, 25735, 83805, 444985, 83689, 24392, 60329, 83619, 170739, 362110, 24408, 257648, 25029, 295703, 25406, 296499, 29466, 65151, 117107, 60450, 117152, 25401, 362862, 25359, 114031, 56761, 114521, 299810, 84607, 64186, 299805, 298584, 25585, 313575, 192110, 313702, 84426, 313611, 25105, 24602, 58918, 29693, 58945, 24817, 288753, 25622, 64514, 66026, 65081, 24547, 266766, 64023, 25666, 303882, 84027, 25471, 29364, 113912, 65096, 29501, 83726, 25414, 50664, 171355, 89842, 54267, 29355, 84026, 116510, 114493, 54252, 84357, 63879, 84469, 317413, 83712, 404280, 83514, 58812, 29134, 114111, 170538, 81507, 266711, 24673, 79114, 25402, 306417, 25531, 24323, 306862, 59328, 113893, 114519, 364680, 29410, 59076, 29200, 171455, 89830, 291023, 81653, 29543, 54276, 79257, 80722, 83833, 64188, 25723, 25574, 56029
cell projection organization and biogenesis	5.2296e-05	25	10.56	Igf1r, Pak1, Sdcbp, Nrcam, Foxg1, Cxcl12, Reln, S100a6, Unc5c, Ss18l1, Gja1, Stxbp1, Rtn4rl2, Capzb, Ptpn11, Mbp, Alcam, Ncam2, Rtn4r, Gnao, Pou4f2, L1cam, Lzts1, Fgfr1, Dbn1	25718, 29431, 83841, 497815, 24370, 24772, 24718, 85247, 362049, 192352, 24392, 25558, 311169, 298584, 25622, 24547, 79559, 288280, 113912, 50664, 171355, 50687, 266711, 79114, 81653
cell projection morphogenesis	5.2296e-05	25	10.56	Igf1r, Pak1, Sdcbp, Nrcam, Foxg1, Cxcl12, Reln, S100a6, Unc5c, Ss18l1, Gja1, Stxbp1, Rtn4rl2, Capzb, Ptpn11, Mbp, Alcam, Ncam2, Rtn4r, Gnao, Pou4f2, L1cam, Lzts1, Fgfr1, Dbn1	25718, 29431, 83841, 497815, 24370, 24772, 24718, 85247, 362049, 192352, 24392, 25558, 311169, 298584, 25622, 24547, 79559, 288280, 113912, 50664, 171355, 50687, 266711, 79114, 81653
multicellular organismal development	5.97878e-05	113	80.90	Fgf3, Pde3b, Igf1r, Mesdc2, Doc2a, Agc1, Tbx6_predicted, Pak1, Fgf15, Tmod1, Nr4a3, Ugcg, Pdpn, Nrcam, Crip2, Foxg1, Yy1, Sftpb, Met, Bhlhb2, C1galt1, Cxcl12, Reln, Ptpn12, Foxm1, Crabp2, Lmo4, Pdgfc, Pde5a, S100a6, Tspan2, Pitx2, Unc5c, Notch2, Plcg1, Tcfap2c, Ss18l1, Src, Cd164, Gja1, Neurog3,	170633, 29516, 25718, 308796, 65031, 58968, 365371, 29431, 170582, 25566, 58853, 83626, 54320, 497815, 338401, 24370, 24919, 192155, 24553, 79431, 65044, 24772, 24718, 117255, 58921, 29563, 362051, 79429, 171115, 85247, 64521, 54284, 362049, 29492, 25738,

				Fnbp1, Nfe2l2, Stxbp1, Calcrl, Atf2, Rtn4rl2, Cd44, Acvr1, Dlx2, Aes, Zbtb7a, Matk, Cart1, Notch3, Dgat1, Socs2, Ascl1, Id3, Heyl_predicted, Pou3f1, Wnt4, Nppb, Ptpn11, Vps33a, Lnk, Mbp, Dscr1, Txnrd2, Alcam, Dgkg, Ncam2, Cxadr, Gsk3b, Clcn2, Rtn4r, Dok4_predicted, Gnao, Pou4f2, Siah1a, Timp1, Mid1, Rbbp7, L1cam, Gpm6b, Dgcr2, Axin2, Vegfc, Bmpr1a, Lzts1, Fgfr1, Sncg, Casp3, Mustn1, Wnt5a, Nr2f6, Edn1, Tcfap2a_predicted, Smad5, Srd5a1, Barx1_predicted, Neurod3, Inhba, Ptch1, Id4, Ninj1, Dbn1, Timp2, Neurod2, Axin1, Sost, Mafg, Nptx1	362280, 192352, 83805, 83689, 24392, 60329, 192348, 83619, 25558, 25029, 81647, 311169, 25406, 79558, 296499, 29466, 117107, 60450, 25401, 56761, 84497, 84607, 64186, 25585, 313575, 192110, 84426, 25105, 25622, 65081, 58838, 24547, 266766, 50551, 79559, 25666, 288280, 89843, 84027, 29232, 113912, 361364, 50664, 171355, 140941, 116510, 54252, 83712, 50687, 192179, 360742, 29134, 114111, 81507, 266711, 79114, 64347, 25402, 290553, 64566, 245980, 24323, 306862, 59328, 24950, 364680, 29410, 29200, 89830, 291023, 25338, 81653, 29543, 54276, 79257, 80722, 64188, 266777
system development	0.000112509	101	71.54	Fgf3, Pde3b, Igf1r, Mesdc2, Doc2a, Agc1, Tbx6_predicted, Pak1, Fgf15, Tmod1, Nr4a3, Ugcg, Pdpn, Nrcam, Crip2, Foxg1, Sftpb, Met, Bhlhb2, C1galt1, Cxcl12, Reln, Ptpn12, Foxm1, Crabp2, Lmo4, Pdgfc, Pde5a, S100a6, Tspan2, Pitx2, Unc5c, Notch2, Ss18l1, Src, Gja1, Fnbp1, Stxbp1, Calcrl, Atf2, Rtn4rl2, Cd44, Acvr1, Dlx2, Aes, Zbtb7a, Matk, Cart1, Notch3, Dgat1, Socs2, Ascl1, Id3, Heyl_predicted, Pou3f1, Wnt4, Nppb, Ptpn11, Vps33a, Lnk, Mbp, Dscr1, Txnrd2, Alcam, Dgkg, Ncam2, Cxadr, Gsk3b, Clcn2, Rtn4r, Dok4_predicted, Gnao, Pou4f2, L1cam, Gpm6b, Dgcr2, Axin2, Vegfc, Bmpr1a, Lzts1, Fgfr1, Sncg, Casp3, Mustn1, Wnt5a, Nr2f6, Edn1, Tcfap2a_predicted, Smad5, Barx1_predicted, Neurod3, Inhba, Ptch1, Id4, Ninj1, Dbn1, Timp2, Neurod2, Sost, Mafg, Nptx1	170633, 29516, 25718, 308796, 65031, 58968, 365371, 29431, 170582, 25566, 58853, 83626, 54320, 497815, 338401, 24370, 192155, 24553, 79431, 65044, 24772, 24718, 117255, 58921, 29563, 362051, 79429, 171115, 85247, 64521, 54284, 362049, 29492, 192352, 83805, 24392, 192348, 25558, 25029, 81647, 311169, 25406, 79558, 296499, 29466, 117107, 60450, 25401, 56761, 84497, 84607, 64186, 25585, 313575, 192110, 84426, 25105, 25622, 65081, 58838, 24547, 266766, 50551, 79559, 25666, 288280, 89843, 84027, 29232, 113912, 361364, 50664, 171355, 50687, 192179, 360742, 29134, 114111, 81507, 266711, 79114, 64347, 25402, 290553, 64566, 245980, 24323, 306862, 59328, 364680, 29410, 29200, 89830, 291023, 25338, 81653, 29543, 54276, 80722, 64188, 266777
regulation of cellular process	0.000161678	133	100.83	Arrb1, Cd81, Abhd2_predicted, Igf1r, Tbx6_predicted, Bag3, Mgmt, Ncoa2, Rab2, Faf1, Anp32b, Tnfrsf1b, Nfia, Pdpn, Unc13b, Nrcam, Crip2, Cgref1, Foxg1, Ppm1a, Yy1, Sfrs5, Cav2, Bhlhb2, Cyp51, Casp2, Kcnh2, Cxcl12, Pparg, Reln, Foxm1, Bcl2l13_predicted, Gclm, Cxxc4, Crabp2, Pdgfc, S100a10, S100a6, Pitx2, Unc5c, Notch2, Pofut1, Tcfap2c, Hnf4a, Src, Cd164, Gja1, Neurog3, Nfe2l2, Kcnh7, Cdk9, Calcrl, Cd44, Dlx2, Aes, Hrsp12, Zbtb7a, Matk, Cand1, Cart1, Tra1_predicted, Tmpo, Fstl3, Notch3, Tdg, Yeats4_predicted, Socs2, Ascl1, Cnot2, Capzb, Id3, Heyl_predicted, Pou3f1, Mad2l2, Wnt4, Pafah2, Nppb, Nppa, Casp9, Tcf1, Diablo, Ptpn11, Eif2b1, Dscr1, Tnk2, Gsk3b, Cd47, Slc12a4, Ctcf, Bcar1, Pou4f2, Mbtps1, Maf, Dnaja2, Timp1, Mid1, Sh3kbp1, Birc4, Maged1, Morf4l2, Rbbp7, Mig12, Tsc22d3, Apln, Axin2, Vegfc, Prkcd, Bmpr1a, Lzts1, Ppp2cb, Fgfr1, Casp3, Sh3md2, Rab3a, Edn1, Tcfap2a_predicted, Smad5, Nfil3, Barx1_predicted, Neurod3, Gprk6, Inhba, Exoc2, Ptch1, Id4,	25387, 25621, 293050, 25718, 365371, 293524, 25332, 83724, 65158, 140657, 170724, 156767, 25492, 54320, 64830, 497815, 338401, 245918, 24370, 24666, 24919, 29667, 363425, 79431, 25427, 64314, 117018, 24772, 25664, 24718, 58921, 312682, 29739, 83824, 29563, 79429, 81778, 85247, 54284, 362049, 29492, 311551, 362280, 25735, 83805, 83689, 24392, 60329, 83619, 170739, 362110, 25029, 25406, 296499, 29466, 65151, 117107, 60450, 117152, 25401, 362862, 25359, 114031, 56761, 114521, 299810, 84607, 64186, 299805, 298584, 25585, 313575, 192110, 313702, 84426, 313611, 25105, 24602, 58918, 24817, 288753, 25622, 64514, 266766, 303882, 84027, 29364, 29501, 83726, 25414, 171355, 89842, 54267, 84026, 116510, 54252, 84357, 63879, 84469, 317413, 83712, 404280, 83514, 58812, 29134, 114111, 170538, 81507, 266711, 24673, 79114, 25402,

				Timp2, Neurod2, Axin1, Sost, Smarcd2, Mafg, Ubtf, Snip	306417, 25531, 24323, 306862, 59328, 114519, 364680, 29410, 59076, 29200, 171455, 89830, 291023, 29543, 54276, 79257, 80722, 83833, 64188, 25574, 56029
regulation of cell differentiation	0.000318119	18	7.24	Nrcam, Foxg1, Pparg, S100a10, Notch2, Zbtb7a, Notch3, Socs2, Id3, Pou3f1, Wnt4, Dscr1, Axin2, Ppp2cb, Inhba, Id4, Timp2, Mafg	497815, 24370, 25664, 81778, 29492, 117107, 56761, 84607, 25585, 192110, 84426, 266766, 29134, 24673, 29200, 291023, 29543, 64188
cell organization and biogenesis	0.000338709	97	70.11	Arrb1, Ap2a2, Picalm, Igf1r, Pak1, Prc1_predicted, Rab2, Sdcbp, Faf1, Tmod1, Clta, Kif1b, Eps15, Nrcam, Rhoj, Cgref1, Foxg1, Rock2, Sfrs5, Cav2, Zfp384, Arf5, Dctn1, Cxcl12, Reln, Ppp3ca, S100a10, S100a6, Syt6, Unc5c, Sep15, Notch2, Ss18l1, Cd24, Gja1, Gyltl1b, Arl5a, Stxbp1, Rtn4rl2, Cd44, Akap8, Hrsp12, Ywhaz, Tmpo, Vezt, Socs2, Capzb, Ebna1bp2, Nppb, Nppa, Dynll1, Erp29, Ptpn11, Eif2b1, Mbp, Alcam, Ncam2, Tnk2, Cxadr, Gsk3b, Ap2m1, Cd47, Rtn4r, MAST1, Bcar1, Gnao, Pou4f2, Glg1, Mbtps1, Tomm20, Bcap31, Mid1, Morf4l2, Rbbp7, Mig12, L1cam, Slc25a1, Myo9b, Lzts1, Ppp2cb, Arf4, Fgfr1, Casp3, Vps36_predicted, Rab3a, Kif27, Bicd2, Hist1h1t, Exoc2, Dbn1, Arhgap27, Prm2, Cltc, Smarcd2, Kpnb1, Rpl23, H3f3b	25387, 81637, 89816, 25718, 29431, 308761, 65158, 83841, 140657, 25566, 83800, 117548, 313474, 497815, 299145, 245918, 24370, 25537, 29667, 363425, 171018, 79117, 29167, 24772, 24718, 24674, 81778, 85247, 60565, 362049, 113922, 29492, 192352, 25145, 24392, 311202, 117050, 25558, 311169, 25406, 116633, 65151, 25578, 25359, 299738, 84607, 298584, 114021, 25105, 24602, 58945, 117030, 25622, 64514, 24547, 79559, 288280, 303882, 89843, 84027, 116563, 29364, 113912, 353118, 25414, 50664, 171355, 29476, 89842, 266601, 293852, 54252, 317413, 83712, 404280, 50687, 29743, 25486, 266711, 24673, 79120, 79114, 25402, 290851, 25531, 246209, 306809, 24438, 171455, 81653, 303583, 25345, 54241, 83833, 24917, 29282, 117056
pattern specification process	0.000386352	17	6.73	Fgf3, Tbx6_predicted, Foxg1, Yy1, Cxcl12, Pitx2, Notch2, Acvr1, Ascl1, Mid1, Axin2, Bmpr1a, Fgfr1, Edn1, Smad5, Ptch1, Axin1	170633, 365371, 24370, 24919, 24772, 54284, 29492, 79558, 64186, 54252, 29134, 81507, 79114, 24323, 59328, 89830, 79257
neurite morphogenesis	0.000467362	18	7.47	Nrcam, Foxg1, Cxcl12, Reln, S100a6, Unc5c, Gja1, Stxbp1, Rtn4rl2, Ptpn11, Mbp, Alcam, Ncam2, Rtn4r, Gnao, Pou4f2, L1cam, Lzts1	497815, 24370, 24772, 24718, 85247, 362049, 24392, 25558, 311169, 25622, 24547, 79559, 288280, 113912, 50664, 171355, 50687, 266711
neuron morphogenesis during differentiation	0.000467362	18	7.47	Nrcam, Foxg1, Cxcl12, Reln, S100a6, Unc5c, Gja1, Stxbp1, Rtn4rl2, Ptpn11, Mbp, Alcam, Ncam2, Rtn4r, Gnao, Pou4f2, L1cam, Lzts1	497815, 24370, 24772, 24718, 85247, 362049, 24392, 25558, 311169, 25622, 24547, 79559, 288280, 113912, 50664, 171355, 50687, 266711
positive regulation of biological process	0.000682018	66	44.69	Arrb1, Cd81, Igf1r, Tbx6_predicted, Ncoa2, Faf1, Pdpn, Unc13b, Nrcam, Crip2, Foxg1, Ppm1a, Adcy3, Met, Casp2, Cxcl12, Pparg, Reln, Foxm1, Bcl2l13_predicted, Gclm, Pdgfc, S100a6, Notch2, Hnf4a, Src, Gja1, Neurog3, Nfe2l2, Calcrl, Serping1, Dlx2, Matk, Cand1, Socs2, Id3, Casp9, Tcf1, Diablo, Ptpn11, Masp1, Dgkg, Tnk2, Gsk3b, Cd47, Ctcf, Bcar1, Maf, Pkn1, Dnaja2, Timp1, Apln, Vegfc, Prkcd, Bmpr1a, Ppp2cb, Fgfr1, Casp3, Sh3md2, Edn1, Tcfap2a_predicted, Smad5, Inhba, Exoc2, Id4, Timp2	25387, 25621, 25718, 365371, 83724, 140657, 54320, 64830, 497815, 338401, 24370, 24666, 64508, 24553, 64314, 24772, 25664, 24718, 58921, 312682, 29739, 79429, 85247, 29492, 25735, 83805, 24392, 60329, 83619, 25029, 295703, 296499, 60450, 117152, 84607, 25585, 58918, 24817, 288753, 25622, 64023, 25666, 303882, 84027, 29364, 83726, 25414, 54267, 29355, 84026, 116510, 58812, 114111, 170538, 81507, 24673, 79114, 25402, 306417, 24323, 306862, 59328, 29200, 171455, 291023, 29543
generation of a signal involved in	0.000863313	13	4.75	Pde3b, Doc2a, Unc13b, Syn2, Cacna1b, Dgat1, Trpv4, Lphn1, Cacna1f, Rab3a, Edn1, Inhba, Hcrt	29516, 65031, 64830, 29179, 257648, 84497, 66026, 65096, 114493, 25531, 24323, 29200, 25723

cell-cell signaling					
positive regulation of cellular process	0.000898809	56	36.81	Cd81, Igf1r, Tbx6_predicted, Ncoa2, Faf1, Pdpn, Unc13b, Nrcam, Crip2, Foxg1, Ppm1a, Casp2, Cxcl12, Pparg, Foxm1, Bcl2l13_predicted, Pdgfc, S100a6, Notch2, Hnf4a, Src, Gja1, Neurog3, Nfe2l2, Calcrl, Dlx2, Matk, Cand1, Socs2, Id3, Casp9, Tcf1, Diablo, Tnk2, Gsk3b, Cd47, Ctcf, Bcar1, Maf, Dnaja2, Timp1, Apln, Vegfc, Prkcd, Bmpr1a, Ppp2cb, Fgfr1, Casp3, Sh3md2, Edn1, Tcfap2a_predicted, Smad5, Inhba, Exoc2, Id4, Timp2	25621, 25718, 365371, 83724, 140657, 54320, 64830, 497815, 338401, 24370, 24666, 64314, 24772, 25664, 58921, 312682, 79429, 85247, 29492, 25735, 83805, 24392, 60329, 83619, 25029, 296499, 60450, 117152, 84607, 25585, 58918, 24817, 288753, 303882, 84027, 29364, 83726, 25414, 54267, 84026, 116510, 58812, 114111, 170538, 81507, 24673, 79114, 25402, 306417, 24323, 306862, 59328, 29200, 171455, 291023, 29543
platelet-derived growth factor receptor signaling pathway	0.000908702	3	0.23	Pdgfc, Src, Bcar1	79429, 83805, 25414
axonogenesis	0.00110502	16	6.73	Nrcam, Foxg1, Cxcl12, Reln, S100a6, Unc5c, Stxbp1, Rtn4rl2, Ptpn11, Mbp, Alcam, Ncam2, Rtn4r, Gnao, Pou4f2, L1cam	497815, 24370, 24772, 24718, 85247, 362049, 25558, 311169, 25622, 24547, 79559, 288280, 113912, 50664, 171355, 50687
metal ion transport	0.00115141	23	11.48	Npy, Kcnh2, Ppp3ca, Accn5, Kcnd3, Plcg1, Kcnh7, Cacnb4, Grin1, Cacna1b, Slc34a3, Hcn2, Kcnq3, Grik3, Atp2a2, Kcne1, Cacna1f, Cacna2d3, Kcnmb1, Cacng4, Cacnb1, Cacna1g, Kcnh4	24604, 117018, 24674, 63866, 65195, 25738, 170739, 58942, 24408, 257648, 246234, 114244, 29682, 298521, 29693, 25471, 114493, 306243, 29747, 140725, 50688, 29717, 114032
calcium ion transport	0.00115823	11	3.74	Npy, Ppp3ca, Plcg1, Cacnb4, Cacna1b, Atp2a2, Cacna1f, Cacna2d3, Cacng4, Cacnb1, Cacna1g	24604, 24674, 25738, 58942, 257648, 29693, 114493, 306243, 140725, 50688, 29717
transmission of nerve impulse	0.00131716	32	18.22	Doc2a, Sdcbp, Gabrd, Unc13b, Nrcam, Npy, Slc6a1, Cav2, Bhlhb2, Syn2, Cd24, Grin1, Cacna1b, Lynx1_predicted, Kcnq3, Pou3f1, Grik3, Mbp, Lphn1, Cacna1f, Gabra3, Lzts1, Rab3a, Edn1, Sncb, Dbn1, Kcnmb1, Cacng4, Hcrt, Nptx1, Grin2a, Gabrb2	65031, 83841, 29689, 64830, 497815, 24604, 79212, 363425, 79431, 29179, 25145, 24408, 257648, 300018, 29682, 192110, 298521, 24547, 65096, 114493, 24947, 266711, 25531, 24323, 113893, 81653, 29747, 140725, 25723, 266777, 24409, 25451
negative regulation of cellular process	0.00135224	55	36.62	Arrb1, Abhd2_predicted, Igf1r, Bag3, Ncoa2, Faf1, Cgref1, Foxg1, Yy1, Cav2, Bhlhb2, Pparg, Gclm, Cxxc4, Notch2, Cd164, Gja1, Aes, Zbtb7a, Tra1_predicted, Fstl3, Notch3, Tdg, Capzb, Id3, Pou3f1, Wnt4, Pafah2, Nppb, Nppa, Tcf1, Eif2b1, Dscr1, Gsk3b, Ctcf, Pou4f2, Timp1, Mid1, Birc4, Rbbp7, Mig12, Tsc22d3, Axin2, Ppp2cb, Fgfr1, Casp3, Edn1, Tcfap2a_predicted, Barx1_predicted, Inhba, Id4, Timp2, Axin1, Sost, Snip	25387, 293050, 25718, 293524, 83724, 140657, 245918, 24370, 24919, 363425, 79431, 25664, 29739, 83824, 29492, 83689, 24392, 29466, 117107, 362862, 114031, 56761, 114521, 298584, 25585, 192110, 84426, 313611, 25105, 24602, 24817, 64514, 266766, 84027, 83726, 171355, 116510, 54252, 63879, 83712, 404280, 83514, 29134, 24673, 79114, 25402, 24323, 306862, 364680, 29200, 291023, 29543, 79257, 80722, 56029
cellular morphogenesis during differentiation	0.0014169	18	8.21	Nrcam, Foxg1, Cxcl12, Reln, S100a6, Unc5c, Gja1, Stxbp1, Rtn4rl2, Ptpn11, Mbp, Alcam, Ncam2, Rtn4r, Gnao, Pou4f2, L1cam, Lzts1	497815, 24370, 24772, 24718, 85247, 362049, 24392, 25558, 311169, 25622, 24547, 79559, 288280, 113912, 50664, 171355, 50687, 266711
negative regulation of	0.00141983	11	3.83	Foxg1, Notch2, Notch3, Id3, Wnt4, Mbp, Dscr1, Rtn4r, Axin2, Inhba, Id4	24370, 29492, 56761, 25585, 84426, 24547, 266766, 113912, 29134, 29200, 291023

developmental process					
synaptic transmission	0.00163234	29	16.19	Doc2a, Sdcbp, Gabrd, Unc13b, Npy, Slc6a1, Cav2, Bhlhb2, Syn2, Cd24, Grin1, Cacna1b, Lynx1_predicted, Kcnq3, Grik3, Mbp, Lphn1, Cacna1f, Gabra3, Lzts1, Rab3a, Edn1, Sncb, Dbn1, Kcnmb1, Hcrt, Nptx1, Grin2a, Gabrb2	65031, 83841, 29689, 64830, 24604, 79212, 363425, 79431, 29179, 25145, 24408, 257648, 300018, 29682, 298521, 24547, 65096, 114493, 24947, 266711, 25531, 24323, 113893, 81653, 29747, 25723, 266777, 24409, 25451
nerve growth factor receptor signaling pathway	0.00175446	3	0.28	Src, Ptpn11, Bcar1	83805, 25622, 25414
embryonic development	0.00187565	24	12.64	Tbx6_predicted, Nr4a3, Cxcl12, Crabp2, Lmo4, Pitx2, Notch2, Plcg1, Tcfap2c, Gja1, Nfe2l2, Acvr1, Wnt4, Lnk, Axin2, Vegfc, Fgfr1, Mustn1, Wnt5a, Edn1, Smad5, Neurod3, Ptch1, Mafg	365371, 58853, 24772, 29563, 362051, 54284, 29492, 25738, 362280, 24392, 83619, 79558, 84426, 58838, 29134, 114111, 79114, 290553, 64566, 24323, 59328, 29410, 89830, 64188
negative regulation of cell differentiation	0.00200038	9	2.86	Foxg1, Notch2, Notch3, Id3, Wnt4, Dscr1, Axin2, Inhba, Id4	24370, 29492, 56761, 25585, 84426, 266766, 29134, 29200, 291023
heart development	0.00209547	12	4.61	Fgf15, Pitx2, Gja1, Calcrl, Acvr1, Id3, Nppb, Txnrd2, Cxadr, Bmpr1a, Casp3, Edn1	170582, 54284, 24392, 25029, 79558, 25585, 25105, 50551, 89843, 81507, 25402, 24323
regulation of Wnt receptor signaling pathway	0.00224577	5	0.97	Cxxc4, Axin2, Ppp2cb, Barx1_predicted, Axin1	83824, 29134, 24673, 364680, 79257
epidermal growth factor receptor signaling pathway	0.00224577	5	0.97	Eps15, Src, Bcar1, Arf4, Grb2	313474, 83805, 25414, 79120, 81504
cell fate commitment	0.00269323	10	3.55	Fgf3, Tbx6_predicted, Pparg, Notch2, Aes, Notch3, Ascl1, Fgfr1, Casp3, Neurod3	170633, 365371, 25664, 29492, 29466, 56761, 64186, 79114, 25402, 29410
cation transport	0.00283757	26	14.53	Npy, Kcnh2, Ppp3ca, Accn5, Kcnd3, Plcg1, Atp5g3, Kcnh7, Cacnb4, Grin1, Cacna1b, Slc34a3, Hcn2, Kcnq3, Grik3, Atp2a2, Trpv4, Kcne1, Cacna1f, Atp6ap1, Cacna2d3, Kcnmb1, Cacng4, Cacnb1, Cacna1g, Kcnh4	24604, 117018, 24674, 63866, 65195, 25738, 114630, 170739, 58942, 24408, 257648, 246234, 114244, 29682, 298521, 29693, 66026, 25471, 114493, 83615, 306243, 29747, 140725, 50688, 29717, 114032
positive regulation of neuron differentiation	0.00296356	3	0.32	Nrcam, Socs2, Timp2	497815, 84607, 29543
negative regulation of Wnt receptor signaling pathway	0.00308655	4	0.65	Cxxc4, Axin2, Barx1_predicted, Axin1	83824, 29134, 364680, 79257
fluid secretion	0.00308655	4	0.65	Clcnk1, Nppb, Tsc22d3, Edn1	79425, 25105, 83514, 24323
	0.0035551	29	17.07		

protein amino acid phosphorylation				Cd81, Phkg2, Igf1r, Pak1, Pink1_predicted, Rock2, Prkch, Met, Reln, Pdgfc, Src, Cdk9, Hipk3, Stk39, Acvr1, Matk, Csnk1g2, Tnk2, Gsk3b, Tssk2, MAST1, Pkn1, Btk, Vegfc, Prkcd, Ppp2cb, Fgfr1, Gprk6, Il12b	25621, 140671, 25718, 29431, 298575, 25537, 81749, 24553, 24718, 79429, 83805, 362110, 83617, 54348, 79558, 60450, 65278, 303882, 84027, 304181, 353118, 29355, 367901, 114111, 170538, 24673, 79114, 59076, 64546
epithelial cell differentiation	0.00368476	7	2.03	Pparg, Gja1, Neurog3, Plec1, Id3, Kcne1, Barx1_predicted	25664, 24392, 60329, 64204, 25585, 25471, 364680
tissue development	0.00410288	24	13.42	Mesdc2, Agc1, Tbx6_predicted, Nr4a3, Ugcg, Ptpn12, Crabp2, Notch2, Gja1, Rtn4rl2, Zbtb7a, Matk, Cart1, Notch3, Alcam, Axin2, Bmpr1a, Mustn1, Edn1, Tcfap2a_predicted, Smad5, Barx1_predicted, Sost, Mafg	308796, 58968, 365371, 58853, 83626, 117255, 29563, 29492, 24392, 311169, 117107, 60450, 25401, 56761, 79559, 29134, 81507, 290553, 24323, 306862, 59328, 364680, 80722, 64188
phosphate metabolic process	0.00415479	38	24.40	Cd81, Phkg2, Igf1r, Pak1, Pink1_predicted, Rock2, Ppm1a, Prkch, Met, Reln, Ptpn12, Ppp3ca, Pdgfc, Ptpn1, Src, Ndufs3_predicted, Cdk9, Hipk3, Stk39, Acvr1, Phpt1_predicted, Matk, Csnk1g2, Tnk2, Gsk3b, Tssk2, MAST1, Pkn1, Pdp2, Btk, Vegfc, Prkcd, Ppp2cb, Fgfr1, Dusp4, Gprk6, Inhba, Il12b	25621, 140671, 25718, 29431, 298575, 25537, 24666, 81749, 24553, 24718, 117255, 24674, 79429, 24697, 83805, 295923, 362110, 83617, 54348, 79558, 296571, 60450, 65278, 303882, 84027, 304181, 353118, 29355, 246311, 367901, 114111, 170538, 24673, 79114, 60587, 59076, 29200, 64546
phosphorus metabolic process	0.00415479	38	24.40	Cd81, Phkg2, Igf1r, Pak1, Pink1_predicted, Rock2, Ppm1a, Prkch, Met, Reln, Ptpn12, Ppp3ca, Pdgfc, Ptpn1, Src, Ndufs3_predicted, Cdk9, Hipk3, Stk39, Acvr1, Phpt1_predicted, Matk, Csnk1g2, Tnk2, Gsk3b, Tssk2, MAST1, Pkn1, Pdp2, Btk, Vegfc, Prkcd, Ppp2cb, Fgfr1, Dusp4, Gprk6, Inhba, Il12b	25621, 140671, 25718, 29431, 298575, 25537, 24666, 81749, 24553, 24718, 117255, 24674, 79429, 24697, 83805, 295923, 362110, 83617, 54348, 79558, 296571, 60450, 65278, 303882, 84027, 304181, 353118, 29355, 246311, 367901, 114111, 170538, 24673, 79114, 60587, 59076, 29200, 64546
regulation of neuron differentiation	0.00415929	6	1.57	Nrcam, Foxg1, Notch3, Socs2, Id4, Timp2	497815, 24370, 56761, 84607, 291023, 29543
negative regulation of biological process	0.00432734	58	41.19	Arrb1, Abhd2_predicted, Igf1r, Bag3, Ncoa2, Faf1, Cgref1, Foxg1, Yy1, Cav2, Bhlhb2, Pparg, Gclm, Cxxc4, Notch2, Cd164, Gja1, Aes, Zbtb7a, Tra1_predicted, Fstl3, Notch3, Tdg, Capzb, Id3, Pou3f1, Wnt4, Pafah2, Nppb, Nppa, Tcf1, Eif2b1, Mbp, Dscr1, Gsk3b, Rtn4r, Ctcf, Pou4f2, Timp1, Mid1, Birc4, Rbbp7, Mig12, Tsc22d3, Axin2, Ppp2cb, Fgfr1, Casp3, Edn1, Tcfap2a_predicted, Barx1_predicted, Inhba, Ptch1, Id4, Timp2, Axin1, Sost, Snip	25387, 293050, 25718, 293524, 83724, 140657, 245918, 24370, 24919, 363425, 79431, 25664, 29739, 83824, 29492, 83689, 24392, 29466, 117107, 362862, 114031, 56761, 114521, 298584, 25585, 192110, 84426, 313611, 25105, 24602, 24817, 64514, 24547, 266766, 84027, 113912, 83726, 171355, 116510, 54252, 63879, 83712, 404280, 83514, 29134, 24673, 79114, 25402, 24323, 306862, 364680, 29200, 89830, 291023, 29543, 79257, 80722, 56029
cell migration	0.00434393	22	11.99	Abhd2_predicted, Gab2, Fgf15, Sdcbp, Pdpn, Nrcam, Foxg1, Met, Cxcl12, Reln, Pitx2, Unc5c, Gja1, Acvr1, Ascl1, Alcam, Cd47, Bcar1, Gnao, Pou4f2, L1cam, Vegfc	293050, 84477, 170582, 83841, 54320, 497815, 24370, 24553, 24772, 24718, 54284, 362049, 24392, 79558, 64186, 79559, 29364, 25414, 50664, 171355, 50687, 114111
small GTPase mediated signal	0.00461694	15	7.06	Pak1, Sdcbp, Rhoj, Reln, RSA-14-44, Notch2, Arl5a, Tnk2, Rasd2, Gdi1, Myo9b, Ppp2cb, Rab3a, Timp2, Grb2	29431, 83841, 299145, 24718, 297173, 29492, 117050, 303882, 171099, 25183, 25486, 24673, 25531, 29543,

transduction					81504
localization of cell	0.00497237	28	16.70	Abhd2_predicted, Gab2, Fgf15, Sdcbp, Pdpn, Nrcam, Foxg1, Npy, Met, Akap3, Cxcl12, Reln, Tspan2, Pitx2, Unc5c, Gja1, Acvr1, Ascl1, Capzb, Alcam, Cd47, Cd97, Bcar1, Gnao, Pou4f2, L1cam, Dnai2, Vegfc	293050, 84477, 170582, 83841, 54320, 497815, 24370, 24604, 24553, 312720, 24772, 24718, 64521, 54284, 362049, 24392, 79558, 64186, 298584, 79559, 29364, 361383, 25414, 50664, 171355, 50687, 360654, 114111
cell motility	0.00497237	28	16.70	Abhd2_predicted, Gab2, Fgf15, Sdcbp, Pdpn, Nrcam, Foxg1, Npy, Met, Akap3, Cxcl12, Reln, Tspan2, Pitx2, Unc5c, Gja1, Acvr1, Ascl1, Capzb, Alcam, Cd47, Cd97, Bcar1, Gnao, Pou4f2, L1cam, Dnai2, Vegfc	293050, 84477, 170582, 83841, 54320, 497815, 24370, 24604, 24553, 312720, 24772, 24718, 64521, 54284, 362049, 24392, 79558, 64186, 298584, 79559, 29364, 361383, 25414, 50664, 171355, 50687, 360654, 114111
cAMP metabolic process	0.00503666	5	1.15	Arrb1, Adcy3, Adcy5, Edn1, Timp2	25387, 64508, 64532, 24323, 29543
glutamate signaling pathway	0.00503666	5	1.15	Grid2, Grin1, Grik3, Homer3, Grin2a	79220, 24408, 298521, 29548, 24409
positive regulation of cell migration	0.00521392	4	0.74	Pdpn, Cxcl12, Bcar1, Vegfc	54320, 24772, 25414, 114111
morphogenesis of an epithelium	0.00531612	11	4.52	Pparg, Lmo4, Notch2, Gja1, Neurog3, Plec1, Id3, Wnt4, Kcne1, Vegfc, Barx1_predicted	25664, 362051, 29492, 24392, 60329, 64204, 25585, 84426, 25471, 114111, 364680
limb morphogenesis	0.00537136	7	2.17	Crabp2, Notch2, Bmpr1a, Fgfr1, Mustn1, Wnt5a, Ptch1	29563, 29492, 81507, 79114, 290553, 64566, 89830
appendage morphogenesis	0.00537136	7	2.17	Crabp2, Notch2, Bmpr1a, Fgfr1, Mustn1, Wnt5a, Ptch1	29563, 29492, 81507, 79114, 290553, 64566, 89830
appendage development	0.00537136	7	2.17	Crabp2, Notch2, Bmpr1a, Fgfr1, Mustn1, Wnt5a, Ptch1	29563, 29492, 81507, 79114, 290553, 64566, 89830
cell proliferation	0.00573655	44	29.84	Cd81, Igf1r, Anp32b, Tnfrsf1b, Eps15, Crip2, Cgref1, Npy, Cav2, Met, Cxcl12, Pparg, Foxm1, Tspan5, Pdgfc, S100a6, Tspan2, Pitx2, Notch2, Src, Cd164, Gja1, Cdk9, Calcrl, Matk, Ascl1, Id3, Gsk3b, Bcar1, Dnaja2, Timp1, Rbbp7, Apln, Axin2, Vegfc, Prkcd, Bmpr1a, Fgfr1, Casp3, Edn1, Ptch1, Id4, Timp2, Mafg	25621, 25718, 170724, 156767, 313474, 338401, 245918, 24604, 363425, 24553, 24772, 25664, 58921, 362048, 79429, 85247, 64521, 54284, 29492, 83805, 83689, 24392, 362110, 25029, 60450, 64186, 25585, 84027, 25414, 84026, 116510, 83712, 58812, 29134, 114111, 170538, 81507, 79114, 25402, 24323, 89830, 291023, 29543, 64188
spermidine metabolic process	0.00607554	2	0.14	Srm, Dhps	84596, 288923
epithelial cell development	0.00607554	2	0.14	Gja1, Kcne1	24392, 25471
di-, tri-valent inorganic anion homeostasis	0.00607554	2	0.14	Slc34a3, Slc9a3r1	246234, 59114
ribosomal protein import into nucleus	0.00607554	2	0.14	Kpnb1, Rpl23	24917, 29282

negative regulation of Ras protein signal transduction	0.00607554	2	0.14	Ppp2cb, Timp2	24673, 29543
epithelial cell maturation	0.00607554	2	0.14	Gja1, Kcne1	24392, 25471
phosphate ion homeostasis	0.00607554	2	0.14	Slc34a3, Slc9a3r1	246234, 59114
growth	0.00626116	19	10.15	Cd81, Cgref1, Ptpn12, S100a10, Notch2, Gja1, Rtn4rl2, Cd44, Aes, Notch3, Socs2, Nppb, Nppa, Bcar1, Morf4l2, Ppp2cb, Fgfr1, Mustn1, Ptch1	25621, 245918, 117255, 81778, 29492, 24392, 311169, 25406, 29466, 56761, 84607, 25105, 24602, 25414, 317413, 24673, 79114, 290553, 89830
cartilage development	0.00709478	5	1.25	Agc1, Zbtb7a, Cart1, Bmpr1a, Edn1	58968, 117107, 25401, 81507, 24323
protein transport	0.00739005	25	14.85	Ap2a2, Rab2, Sdcbp, Faf1, Clta, Arf5, Ppp3ca, Gyltl1b, Arl5a, Tra1_predicted, Ywhaz, Erp29, Ptpn11, Gsk3b, Ap2m1, Glg1, Mbtps1, Tomm20, Bcap31, Gdi1, Arf4, Rab3a, Cltc, Kpnb1, Rpl23	81637, 65158, 83841, 140657, 83800, 79117, 24674, 311202, 117050, 362862, 25578, 117030, 25622, 84027, 116563, 29476, 89842, 266601, 293852, 25183, 79120, 25531, 54241, 24917, 29282
protein localization	0.00764172	29	18.03	Ap2a2, Cd81, Rab2, Sdcbp, Faf1, Clta, Arf5, Akap3, Ppp3ca, Dnmt3b, Gyltl1b, Arl5a, Tra1_predicted, Ywhaz, Erp29, Ptpn11, Gsk3b, Ap2m1, Glg1, Mbtps1, Tomm20, Bcap31, Gdi1, Vegfc, Arf4, Rab3a, Cltc, Kpnb1, Rpl23	81637, 25621, 65158, 83841, 140657, 83800, 79117, 312720, 24674, 444985, 311202, 117050, 362862, 25578, 117030, 25622, 84027, 116563, 29476, 89842, 266601, 293852, 25183, 114111, 79120, 25531, 54241, 24917, 29282
tube development	0.00809569	12	5.44	Pdpn, Cxcl12, Lmo4, Pitx2, Gja1, Cd44, Wnt4, Clcn2, Bmpr1a, Fgfr1, Wnt5a, Edn1	54320, 24772, 362051, 54284, 24392, 25406, 84426, 29232, 81507, 79114, 64566, 24323
phosphorylation	0.00825698	31	19.74	Cd81, Phkg2, Igf1r, Pak1, Pink1_predicted, Rock2, Prkch, Met, Reln, Pdgfc, Src, Ndufs3_predicted, Cdk9, Hipk3, Stk39, Acvr1, Matk, Csnk1g2, Tnk2, Gsk3b, Tssk2, MAST1, Pkn1, Btk, Vegfc, Prkcd, Ppp2cb, Fgfr1, Gprk6, Inhba, Il12b	25621, 140671, 25718, 29431, 298575, 25537, 81749, 24553, 24718, 79429, 83805, 295923, 362110, 83617, 54348, 79558, 60450, 65278, 303882, 84027, 304181, 353118, 29355, 367901, 114111, 170538, 24673, 79114, 59076, 29200, 64546
potassium ion transport	0.00826756	10	4.15	Kcnh2, Kcnd3, Kcnh7, Grin1, Hcn2, Kcnq3, Grik3, Kcne1, Kcnmb1, Kcnh4	117018, 65195, 170739, 24408, 114244, 29682, 298521, 25471, 29747, 114032
cyclic nucleotide metabolic process	0.00831604	6	1.80	Arrb1, Adcy3, Pde5a, Adcy5, Edn1, Timp2	25387, 64508, 171115, 64532, 24323, 29543
ion transport	0.0089354	30	19.05	Npy, Kcnh2, Slc4a5, Ppp3ca, Accn5, Kcnd3, Plcg1, Atp5g3, Kcnh7, Cacnb4, Grin1, Cacna1b, Slc34a3, Hcn2, Kcnq3, Grik3, Atp2a2, Trpv4, Clcn2, Clic6, Kcne1, Cacna1f, Atp6ap1, Cacna2d3, Kcnmb1, Cacng4, Cacnb1, Cacna1g, Gabrb2, Kcnh4	24604, 117018, 297386, 24674, 63866, 65195, 25738, 114630, 170739, 58942, 24408, 257648, 246234, 114244, 29682, 298521, 29693, 66026, 29232, 304081, 25471, 114493, 83615, 306243, 29747, 140725, 50688, 29717, 25451, 114032
protein kinase cascade	0.00913949	23	13.56	Arrb1, Cd81, Pak1, Faf1, Ppm1a, Met, Cr16, Gja1, Plekhg5, Ptpn11, Ifnar1_predicted, MAST1, Dok4_predicted, Pkn1, Btk,	25387, 25621, 29431, 140657, 24666, 24553, 259242, 24392, 310999, 25622, 288264, 353118, 361364, 29355,

				Ppp2cb, Fgfr1, Sh3md2, Wnt5a, Sh3bp5, Edn1, Timp2, Grb2	367901, 24673, 79114, 306417, 64566, 117186, 24323, 29543, 81504
chromosome condensation	0.00917094	3	0.46	Rock2, Akap8, Prm2	25537, 116633, 25345

GO category "molecular_function"

Number of genes annotated in GO: 422
Number of significant GO groups found: 40

GO group	p-value	# genes (observed)	# genes (expected)	list of genes	GeneIDs
protein binding	6.00066e-09	218	161.07	Arrb1, Itgad, Ap2a2, Cd81, Picalm, Ric8a, Igf1r, Agc1, Pak1, Gab2, Bag3, Trpc2, Prc1_predicted, Fgf15, Ncoa2, Faah, Sdcbp, Klhl17, Faf1, Tmod1, Kif1b, Fbxo6b, Tesk1, Tnfrsf1b, Atp6v1g1_predicted, Msc_predicted, Eps15, Tdrd7, Nrcam, Crip2, Rhoj, Rock2, Dtnb, Yy1, Sfrs5, Begain, Adcy3, Chn2, Rtkn, Npy, Cav2, Prkag2, Zfp384, Met, Bhlhb2, Grid2, Dctn1, Syn2, Casp2, Cr16, Akap3, Cxcl12, Pparg, Prkab2, Gclm, Cxxc4, Pias3, Sf3b4, Ppp3ca, Plrg1, Lmo4, Pdgfc, Cnn3, S100a6, Syt6, Pitx2, Unc5c, Kcnd3, Sep15, Notch2, Map2k1ip1, Ptpn1, Plcg1, Tcfap2c, A, Epb4.1l1, Ss18l1, Trp53rk_predicted, Src, Dlgap4, Arfgef2, Dnmt3b, Gja1, Fnbp1, Nfe2l2, Atp5g3, Cdk9, Grin1, Cacna1b, Stk39, Stxbp1, Atf2, Chn1, Cd44, Acvr1, Sh3glb2, Dlx2, Akap8, Aes, Zbtb7a, Vps28_predicted, Matk, Lynx1_predicted, Ptbp1, Cand1, Cart1, Plec1, Tra1_predicted, Ywhaz, Tmpo, Fstl3, Tdg, Vezt, Nxph4, Fbxo7, Yeats4_predicted, Socs2, Ascl1, Capzb, Id3, Mad2l2, Ebna1bp2, Hpcal4, Wnt4, Nppb, Dlgap3, Nppa, Casp9, Grik3, Dynll1, Tcf1, Ptpn11, Eif2b1, Trpv4, Lnk, Fgf12, Dscr1, Txnrd2, Alcam, Tnk2, Cxadr, Gsk3b, Clcn2, Ap2m1, Clic6, Ifnar1_predicted, Kcne1, Cd47, Rtn4r, Tradd, Lphn1, Cd97, Ctcf, Bcar1, Vps4a, Glg1, Siah1a, Maf, Pkn1, Tomm20, Bcap31, Timp1, Mid1, Sh3kbp1, Gdi1, Maged1, Morf4l2, Rbbp7, Mig12, Apln, Btk, Axin2, Vegfc, Myo9b, Upf3a, Prkcd, Lzts1, Ppp2cb, Arf4, Fgfr1, Vps36_predicted, Sh3md2, Wnt5a, Sh3bp5, Rab3a, Edn1, Tcfap2a_predicted, Smad5, Nfil3, Bicd2, Mxd3, Gkap1, Ctsl, Inhba, Fbp1, Exoc2, Ptch1, Id4, Dbn1, Il12b, Timp2, Axin1, Dnajc7, Sfrs2, Sost, Cltc, Cacnb1, Smarcd2, Emp2, Grb2, Hcrt, Ubtf, Kpnb1, Nxph3, Snip, Grin2a, Gabrb2, Pip5k2b	25387, 64350, 81637, 25621, 89816, 293614, 25718, 58968, 29431, 84477, 293524, 64573, 308761, 170582, 83724, 29347, 83841, 246757, 140657, 25566, 117548, 192351, 29460, 156767, 298103, 312897, 313474, 85425, 497815, 338401, 299145, 25537, 362715, 24919, 29667, 79146, 64508, 84031, 297383, 24604, 363425, 373545, 171018, 24553, 79431, 79220, 29167, 29179, 64314, 259242, 312720, 24772, 25664, 64562, 29739, 83824, 83614, 295270, 24674, 60376, 362051, 79429, 54321, 85247, 60565, 54284, 362049, 65195, 113922, 29492, 362045, 24697, 25738, 362280, 24152, 59317, 192352, 362272, 83805, 286930, 296380, 444985, 24392, 192348, 83619, 114630, 362110, 24408, 257648, 54348, 25558, 81647, 84030, 25406, 79558, 311848, 296499, 116633, 29466, 117107, 300052, 60450, 300018, 29497, 117152, 25401, 64204, 362862, 25578, 25359, 114031, 114521, 299738, 59316, 366854, 299810, 84607, 64186, 298584, 25585, 313702, 114021, 50872, 84426, 25105, 286923, 24602, 58918, 298521, 58945, 24817, 25622, 64514, 66026, 58838, 170630, 266766, 50551, 79559, 303882, 89843, 84027, 29232, 116563, 304081, 288264, 25471, 29364, 113912, 246756, 65096, 361383, 83726, 25414, 246772, 29476, 140941, 54267, 29355, 266601, 293852, 116510, 54252, 84357, 25183, 84469, 317413, 83712, 404280, 58812, 367901, 29134, 114111, 25486, 361176, 170538, 266711, 24673, 79120, 79114, 290851, 306417, 64566, 117186,

					25531, 24323, 306862, 59328, 114519, 306809, 252915, 361202, 25697, 29200, 24362, 171455, 89830, 291023, 81653, 64546, 29543, 79257, 303536, 494445, 80722, 54241, 50688, 83833, 360468, 81504, 25723, 25574, 24917, 59315, 56029, 24409, 25451, 89812
binding	3.56405e-07	287	237.78	Arrb1, Itgad, Ap2a2, Cd81, Picalm, Phkg2, Ric8a, Igf1r, Doc2a, Agc1, Tbx6_predicted, Pak1, Gab2, Bag3, Trpc2, Mgmt, Prc1_predicted, Fgf15, Ncoa2, Rab2, Faah, Sdcbp, Klhl17, Faf1, Tmod1, Kif1b, Fbxo6b, Nr4a3, Tesk1, Tnfrsf1b, Atp6v1g1_predicted, Msc_predicted, Nfia, Eps15, Ssbp3, Tdrd7, Nrcam, Crip2, Rhoj, Smoc1, Rock2, Hpcal1, Dtnb, Yy1, Sfrs5, Begain, Adcy3, Chn2, Rtkn, Npy, Cav2, Prkag2, Zfp384, Arf5, Met, Bhlhb2, Grid2, Cyp51, Tacr1, Dctn1, Syn2, Casp2, Cr16, Akap3, Cxcl12, Pparg, Slc6a11, Abp1, Foxm1, Prkab2, Gclm, Cxxc4, Pias3, Adar, Crabp2, Sf3b4, Ppp3ca, Plrg1, Lmo4, Pdgfc, Cnn3, Pde5a, S100a6, Syt6, Pitx2, Unc5c, Kcnd3, Sep15, Notch2, Map2k1ip1, Ptpn1, Plcg1, Tcfap2c, A, Epb4.1l1, Hnf4a, Ss18l1, Trp53rk_predicted, Src, Bpi, Dlgap4, Arfgef2, Top1, Dnmt3b, Psap, Fabp7, Gja1, Neurog3, Fnbp1, Nfe2l2, Tmx2, Atp5g3, Lcn2, Cacnb4, Cdk9, Arl5a, Grin1, Cacna1b, Stk39, Stxbp1, Atf2, Chn1, Cd44, Acvr1, Rbms1, Dgkz, Sh3glb2, Dlx2, Akap8, Aes, Zbtb7a, Vps28_predicted, Matk, Lynx1_predicted, Ptbp1, Cand1, Cart1, Plec1, Tra1_predicted, Shmt2, Ywhaz, Tmpo, Fstl3, Suox, Notch3, Tdg, Siahbp1, Vezt, Nxph4, Fbxo7, Yeats4_predicted, Socs2, Ascl1, Cyp4f6, Capzb, Id3, Heyl_predicted, Pou3f1, Mad2l2, Ebna1bp2, Hpcal4, Wnt4, Pafah2, Nppb, Dlgap3, Nppa, Casp9, Grik3, Padi2, Acads, Abcb9, Atp2a2, Dynll1, Gtpbp6_predicted, Tcf1, Ptpn11, Eif2b1, Trpv4, Lnk, Fgf12, Dscr1, Txnrd2, Alcam, Tnk2, Cxadr, Gsk3b, Clcn2, Ap2m1, Sfrs10, Clic6, Ifnar1_predicted, Kcne1, Cd47, Rtn4r, Tradd, Lphn1, Cd97, Ctcf, MAST1, Bcar1, Gnao, Vps4a, Pou4f2, Glg1, Siah1a, Maf, Mmaa_predicted, Pkn1, Pdp2, Rasd2, Tomm20, Bcap31, Timp1, Otc, Mid1, Sh3kbp1, Gdi1, Maged1, Morf4l2, Rbbp7, Mig12, Apln, Gabra3, Btk, Slc25a1, Sept9, Axin2, Vegfc, Myo9b, Upf3a, Prkcd, Lzts1, Ppp2cb, Arf4, Fgfr1, Itih3, Vps36_predicted, Sh3md2, Wnt5a, Sh3bp5, Rab3a, Edn1, Tcfap2a_predicted, Smad5, Sephs1, Nfil3, Dek, Bicd2, Barx1_predicted, Mxd3, Neurod3, Gkap1, Hk3, Ctsl, Inhba, Fbp1, Hist1h1t, Exoc2, Ptch1, Id4, Dbn1, Il12b, Timp2, Neurod2, Axin1, Dnajc7, Sfrs2, Sost, Prm2, Cltc, Cacnb1, Abca5, Smarcd2, Emp2, Grb2, Mafg, Hcrt, Ubtf, Kpnb1, Nxph3, Snip, Grin2a, Igf2bp1, Gabrb2, H3f3b, Rnps1, Pip5k2b	25387, 64350, 81637, 25621, 89816, 140671, 293614, 25718, 65031, 58968, 365371, 29431, 84477, 293524, 64573, 25332, 308761, 170582, 83724, 65158, 29347, 83841, 246757, 140657, 25566, 117548, 192351, 58853, 29460, 156767, 298103, 312897, 25492, 313474, 84354, 85425, 497815, 338401, 299145, 314280, 25537, 50871, 362715, 24919, 29667, 79146, 64508, 84031, 297383, 24604, 363425, 373545, 171018, 79117, 24553, 79431, 79220, 25427, 24807, 29167, 29179, 64314, 259242, 312720, 24772, 25664, 79213, 65029, 58921, 64562, 29739, 83824, 83614, 81635, 29563, 295270, 24674, 60376, 362051, 79429, 54321, 171115, 85247, 60565, 54284, 362049, 65195, 113922, 29492, 362045, 24697, 25738, 362280, 24152, 59317, 25735, 192352, 362272, 83805, 296321, 286930, 296380, 64550, 444985, 25524, 80841, 24392, 60329, 192348, 83619, 295701, 114630, 170496, 58942, 362110, 117050, 24408, 257648, 54348, 25558, 81647, 84030, 25406, 79558, 362138, 81821, 311848, 296499, 116633, 29466, 117107, 300052, 60450, 300018, 29497, 117152, 25401, 64204, 362862, 299857, 25578, 25359, 114031, 81805, 56761, 114521, 84401, 299738, 59316, 366854, 299810, 84607, 64186, 266689, 298584, 25585, 313575, 192110, 313702, 114021, 50872, 84426, 313611, 25105, 286923, 24602, 58918, 298521, 29511, 64304, 63886, 29693, 58945, 363931, 24817, 25622, 64514, 66026, 58838, 170630, 266766, 50551, 79559, 303882, 89843, 84027, 29232, 116563, 117259, 304081, 288264, 25471, 29364, 113912, 246756, 65096, 361383, 83726, 353118, 25414, 50664, 246772, 171355, 29476, 140941, 54267, 291939, 29355, 246311, 171099, 266601, 293852, 116510, 25611, 54252, 84357, 25183, 84469, 317413, 83712, 404280, 58812, 24947, 367901, 29743, 83788, 29134,

					114111, 25486, 361176, 170538, 266711, 24673, 79120, 79114, 50693, 290851, 306417, 64566, 117186, 25531, 24323, 306862, 59328, 291314, 114519, 306817, 306809, 364680, 252915, 29410, 361202, 25060, 25697, 29200, 24362, 24438, 171455, 89830, 291023, 81653, 64546, 29543, 54276, 79257, 303536, 494445, 80722, 25345, 54241, 50688, 286970, 83833, 360468, 81504, 64188, 25723, 25574, 24917, 59315, 56029, 24409, 303477, 25451, 117056, 287113, 89812
voltage-gated ion channel activity	1.18685e-05	17	5.11	Clcnkb, Clcnk1, Kcnh2, Kcnd3, Kcnh7, Cacnb4, Cacna1b, Kcnq3, Clcn2, Clic6, Kcne1, Cacna1f, Cacna2d3, Cacng4, Cacnb1, Cacna1g, Kcnh4	79430, 79425, 117018, 65195, 170739, 58942, 257648, 29682, 29232, 304081, 25471, 114493, 306243, 140725, 50688, 29717, 114032
ion transporter activity	1.34959e-05	43	21.80	Slc28a1, Trpc2, Clcnkb, Clcnk1, Atp6v1g1_predicted, Slc5a6, Slc6a1, Kcnh2, Slc6a11, Slc4a5, Abp1, S100a6, Accn5, Kcnd3, Slc32a1, Gja1, Atp5g3, Kcnh7, Cacnb4, Grin1, Cacna1b, Slc34a3, Hcn2, Kcnq3, Grik3, Atp2a2, Trpv4, P2rxl1, Clcn2, Clic6, Kcne1, Slc12a4, Atp6v0d1, Cacna1f, Slc7a3, Cacna2d3, Uqcrfs1, Kcnmb1, Cacng4, Cacnb1, Cacna1g, Gabrb2, Kcnh4	116642, 64573, 79430, 79425, 298103, 170551, 79212, 117018, 79213, 297386, 65029, 85247, 63866, 65195, 83612, 24392, 114630, 170739, 58942, 24408, 257648, 246234, 114244, 29682, 298521, 29693, 66026, 25041, 29232, 304081, 25471, 29501, 291969, 114493, 29485, 306243, 291103, 29747, 140725, 50688, 29717, 25451, 114032
transporter activity	1.64463e-05	67	40.19	Slc28a1, Doc2a, Trpc2, Clcnkb, Cdw92, Clcnk1, Atp6v1g1_predicted, Slc5a6, Slc6a1, Arf5, Kcnh2, Slc6a11, Slc4a5, Abp1, S100a6, Accn5, Syt6, Kcnd3, Slc32a1, Gja1, Atp5g3, Kcnh7, Cacnb4, Grin1, Cacna1b, Slc34a3, Hcn2, Kcnq3, Slc2a5, Grik3, Abcb9, Atp2a2, Trpv4, P2rxl1, Slc12a8, Atp5j, Clcn2, Ap2m1, Clic6, Kcne1, Slc12a4, Atp6v0d1, Cyb5b, Slc7a5, Tomm20, Cacna1f, Slc7a3, Atp6ap1, Slc38a5, Slc25a1, Slc16a6, Upf3a, Cacna2d3, Arf4, Slc27a1, Rab3a, Uqcrfs1, Kcnmb1, Cygb, Cacng4, Cacnb1, Abca5, Kpnb1, Cacna1g, Gabrb2, Kcnh4, Pctp	116642, 65031, 64573, 79430, 85254, 79425, 298103, 170551, 79212, 79117, 117018, 79213, 297386, 65029, 85247, 63866, 60565, 65195, 83612, 24392, 114630, 170739, 58942, 24408, 257648, 246234, 114244, 29682, 65197, 298521, 63886, 29693, 66026, 25041, 266733, 94271, 29232, 116563, 304081, 25471, 29501, 291969, 80773, 50719, 266601, 114493, 29485, 83615, 192208, 29743, 303772, 361176, 306243, 79120, 94172, 25531, 291103, 29747, 170520, 140725, 50688, 286970, 24917, 29717, 25451, 114032, 29510
ion channel activity	3.5081e-05	27	11.58	Trpc2, Clcnkb, Clcnk1, Kcnh2, Abp1, Accn5, Kcnd3, Kcnh7, Cacnb4, Grin1, Cacna1b, Hcn2, Kcnq3, Grik3, Trpv4, P2rxl1, Clcn2, Clic6, Kcne1, Cacna1f, Cacna2d3, Kcnmb1, Cacng4, Cacnb1, Cacna1g, Gabrb2, Kcnh4	64573, 79430, 79425, 117018, 65029, 63866, 65195, 170739, 58942, 24408, 257648, 114244, 29682, 298521, 66026, 25041, 29232, 304081, 25471, 114493, 306243, 29747, 140725, 50688, 29717, 25451, 114032
cation transporter activity	7.18828e-05	35	17.56	Slc28a1, Trpc2, Atp6v1g1_predicted, Slc5a6, Slc6a1, Kcnh2, Slc6a11, Abp1, Accn5, Kcnd3, Slc32a1, Atp5g3, Kcnh7, Cacnb4, Grin1, Cacna1b, Slc34a3, Hcn2, Kcnq3, Grik3, Atp2a2, Trpv4, P2rxl1, Kcne1, Slc12a4, Atp6v0d1, Cacna1f, Slc7a3, Cacna2d3, Uqcrfs1, Kcnmb1, Cacng4, Cacnb1, Cacna1g, Kcnh4	116642, 64573, 298103, 170551, 79212, 117018, 79213, 65029, 63866, 65195, 83612, 114630, 170739, 58942, 24408, 257648, 246234, 114244, 29682, 298521, 29693, 66026, 25041, 25471, 29501, 291969, 114493, 29485, 306243, 291103, 29747,

					140725, 50688, 29717, 114032
alpha-type channel activity	7.45837e-05	28	12.76	Trpc2, Clcnkb, Clcnk1, Kcnh2, Abp1, Accn5, Kcnd3, Gja1, Kcnh7, Cacnb4, Grin1, Cacna1b, Hcn2, Kcnq3, Grik3, Trpv4, P2rxl1, Clcn2, Clic6, Kcne1, Cacna1f, Cacna2d3, Kcnmb1, Cacng4, Cacnb1, Cacna1g, Gabrb2, Kcnh4	64573, 79430, 79425, 117018, 65029, 63866, 65195, 24392, 170739, 58942, 24408, 257648, 114244, 29682, 298521, 66026, 25041, 29232, 304081, 25471, 114493, 306243, 29747, 140725, 50688, 29717, 25451, 114032
channel or pore class transporter activity	8.40552e-05	28	12.84	Trpc2, Clcnkb, Clcnk1, Kcnh2, Abp1, Accn5, Kcnd3, Gja1, Kcnh7, Cacnb4, Grin1, Cacna1b, Hcn2, Kcnq3, Grik3, Trpv4, P2rxl1, Clcn2, Clic6, Kcne1, Cacna1f, Cacna2d3, Kcnmb1, Cacng4, Cacnb1, Cacna1g, Gabrb2, Kcnh4	64573, 79430, 79425, 117018, 65029, 63866, 65195, 24392, 170739, 58942, 24408, 257648, 114244, 29682, 298521, 66026, 25041, 29232, 304081, 25471, 114493, 306243, 29747, 140725, 50688, 29717, 25451, 114032
phorbol ester receptor activity	9.5368e-05	5	0.52	Unc13b, Prkch, Chn2, Chn1, Prkcd	64830, 81749, 84031, 84030, 170538
cation channel activity	0.000174179	22	9.39	Trpc2, Kcnh2, Abp1, Accn5, Kcnd3, Kcnh7, Cacnb4, Grin1, Cacna1b, Hcn2, Kcnq3, Grik3, Trpv4, P2rxl1, Kcne1, Cacna1f, Cacna2d3, Kcnmb1, Cacng4, Cacnb1, Cacna1g, Kcnh4	64573, 117018, 65029, 63866, 65195, 170739, 58942, 24408, 257648, 114244, 29682, 298521, 66026, 25041, 25471, 114493, 306243, 29747, 140725, 50688, 29717, 114032
voltage-gated calcium channel activity	0.000195305	7	1.27	Cacnb4, Cacna1b, Cacna1f, Cacna2d3, Cacng4, Cacnb1, Cacna1g	58942, 257648, 114493, 306243, 140725, 50688, 29717
cyclic-nucleotide phosphodiesterase activity	0.000275238	6	0.96	Pde3b, Pde2a, Pde8a, Pde4b, Pde5a, Pde11a	29516, 81743, 308776, 24626, 171115, 140928
protein domain specific binding	0.000382747	18	7.34	Faf1, Crip2, Zfp384, Cr16, Cxxc4, Dlgap4, Gja1, Ywhaz, Tdg, Vezt, Id3, Hpcal4, Dlgap3, Clic6, Bcar1, Morf4l2, Ppp2cb, Sh3bp5	140657, 338401, 171018, 259242, 83824, 286930, 24392, 25578, 114521, 299738, 25585, 50872, 286923, 304081, 25414, 317413, 24673, 117186
voltage-gated chloride channel activity	0.000611095	4	0.44	Clcnkb, Clcnk1, Clcn2, Clic6	79430, 79425, 29232, 304081
DNA-methyltransferase activity	0.00077492	3	0.22	Mgmt, Dnmt3a, Dnmt3b	25332, 444984, 444985
L-gamma-aminobutyric acid transporter activity	0.00077492	3	0.22	Slc6a1, Slc6a11, Slc32a1	79212, 79213, 83612
calcium channel activity	0.000843403	9	2.53	Trpc2, Cacnb4, Cacna1b, Trpv4, Cacna1f, Cacna2d3, Cacng4, Cacnb1, Cacna1g	64573, 58942, 257648, 66026, 114493, 306243, 140725, 50688, 29717
transcriptional repressor activity	0.00105981	18	7.99	Ncoa2, Msc_predicted, Yy1, Bhlhb2, Pparg, Pias3, Plrg1, Aes, Zbtb7a, Cart1, Tdg, Id3, Tcf1, Ctcf, Rbbp7, Nfil3, Id4, Sfrs2	83724, 312897, 24919, 79431, 25664, 83614, 60376, 29466, 117107, 25401, 114521, 25585, 24817, 83726, 83712, 114519, 291023, 494445
3',5'-cyclic-nucleotide phosphodiesterase activity	0.00139597	5	0.87	Pde3b, Pde2a, Pde8a, Pde4b, Pde5a	29516, 81743, 308776, 24626, 171115
	0.00162884	24	12.49		

transcription factor binding				Ncoa2, Faf1, Msc_predicted, Yy1, Pias3, Plrg1, Lmo4, Pitx2, Ss18l1, Cdk9, Atf2, Aes, Cart1, Tdg, Id3, Gsk3b, Ctcf, Maged1, Morf4l2, Tcfap2a_predicted, Nfil3, Id4, Sfrs2, Smarcd2	83724, 140657, 312897, 24919, 83614, 60376, 362051, 54284, 192352, 362110, 81647, 29466, 25401, 114521, 25585, 84027, 83726, 84469, 317413, 306862, 114519, 291023, 494445, 83833
transcription corepressor activity	0.00164011	11	3.89	Msc_predicted, Yy1, Pias3, Plrg1, Aes, Cart1, Id3, Ctcf, Nfil3, Id4, Sfrs2	312897, 24919, 83614, 60376, 29466, 25401, 25585, 83726, 114519, 291023, 494445
phosphotransferase activity, alcohol group as acceptor	0.00192443	34	20.18	Bckdk, Phkg2, Igf1r, Pak1, Tesk1, Pink1_predicted, Rock2, Prkch, Prkag2, Met, Kcnh2, Prkab2, Src, Cdk9, Hipk3, Stk39, Acvr1, Dgkz, Matk, Csnk1g2, Dgkg, Tnk2, Gsk3b, Tssk2, MAST1, Pkn1, Btk, Prkcd, Bmpr1a, Fgfr1, Gprk6, Hk3, Il12b, Pip5k2b	29603, 140671, 25718, 29431, 29460, 298575, 25537, 81749, 373545, 24553, 117018, 64562, 83805, 362110, 83617, 54348, 79558, 81821, 60450, 65278, 25666, 303882, 84027, 304181, 353118, 29355, 367901, 170538, 81507, 79114, 59076, 25060, 64546, 89812
receptor binding	0.00205802	39	24.20	Arrb1, Ncoa2, Faah, Sdcbp, Faf1, Npy, Cxcl12, Pdgfc, Ptpn1, Plcg1, A, Arfgef2, Grin1, Tra1_predicted, Tmpo, Nxph4, Socs2, Wnt4, Nppb, Nppa, Fgf12, Alcam, Clic6, Tradd, Glg1, Bcap31, Apln, Vegfc, Arf4, Wnt5a, Edn1, Mxd3, Inhba, Il12b, Timp2, Emp2, Grb2, Hcrt, Nxph3	25387, 83724, 29347, 83841, 140657, 24604, 24772, 79429, 24697, 25738, 24152, 296380, 24408, 362862, 25359, 59316, 84607, 84426, 25105, 24602, 170630, 79559, 304081, 246756, 29476, 293852, 58812, 114111, 79120, 64566, 24323, 252915, 29200, 64546, 29543, 360468, 81504, 25723, 59315
protein kinase activity	0.00210586	30	17.21	Bckdk, Phkg2, Igf1r, Pak1, Tesk1, Pink1_predicted, Rock2, Prkch, Prkag2, Met, Kcnh2, Prkab2, Src, Cdk9, Hipk3, Stk39, Acvr1, Matk, Csnk1g2, Tnk2, Gsk3b, Tssk2, MAST1, Pkn1, Btk, Prkcd, Bmpr1a, Fgfr1, Gprk6, Il12b	29603, 140671, 25718, 29431, 29460, 298575, 25537, 81749, 373545, 24553, 117018, 64562, 83805, 362110, 83617, 54348, 79558, 60450, 65278, 303882, 84027, 304181, 353118, 29355, 367901, 170538, 81507, 79114, 59076, 64546
channel regulator activity	0.0039976	7	2.05	Npy, Pias3, Lynx1_predicted, Hpcal4, Kcne1, Cacna2d3, Kcnmb1	24604, 83614, 300018, 50872, 25471, 306243, 29747
kinase activity	0.00403022	35	21.93	Bckdk, Phkg2, Igf1r, Pak1, Tesk1, Pink1_predicted, Rock2, Prkch, Prkag2, Met, Kcnh2, Prkab2, Src, Cdk9, Hipk3, Stk39, Acvr1, Dgkz, Matk, Csnk1g2, Dgkg, Tnk2, Gsk3b, Tssk2, MAST1, Pkn1, Btk, Prkcd, Bmpr1a, Fgfr1, Sephs1, Gprk6, Hk3, Il12b, Pip5k2b	29603, 140671, 25718, 29431, 29460, 298575, 25537, 81749, 373545, 24553, 117018, 64562, 83805, 362110, 83617, 54348, 79558, 81821, 60450, 65278, 25666, 303882, 84027, 304181, 353118, 29355, 367901, 170538, 81507, 79114, 291314, 59076, 25060, 64546, 89812
phosphoric ester hydrolase activity	0.00428906	19	9.79	Pde3b, Pde2a, Pde8a, Pde4b, Alpl, Ppm1a, Ptpn12, Ppp3ca, Pde5a, Ptpn1, Plcg1, Pde11a, Phpt1_predicted, Nt5c1a_predicted, Ptpn11, Pdp2, Ppp2cb, Dusp4, Fbp1	29516, 81743, 308776, 24626, 25586, 24666, 117255, 24674, 171115, 24697, 25738, 140928, 296571, 313574, 25622, 246311, 24673, 60587, 24362
bHLH transcription factor binding	0.00546383	2	0.13	Cdk9, Id3	362110, 25585
Hsp70/Hsc70 protein regulator activity	0.00546383	2	0.13	Bag3, Faf1	293524, 140657
SH3/SH2 adaptor activity	0.00565451	5	1.18	Gab2, Chn2, Chn1, Sh3bp5, Grb2	84477, 84031, 84030, 117186, 81504

carboxylic acid transporter activity	0.00565641	9	3.32	Slc6a1, Slc6a11, Slc32a1, Slc12a8, Slc7a5, Slc7a3, Slc38a5, Slc25a1, Slc16a6	79212, 79213, 83612, 266733, 50719, 29485, 192208, 29743, 303772
3',5'-cyclic-AMP phosphodiesterase activity	0.00570902	3	0.39	Pde3b, Pde8a, Pde4b	29516, 308776, 24626
organic acid transporter activity	0.00616508	9	3.36	Slc6a1, Slc6a11, Slc32a1, Slc12a8, Slc7a5, Slc7a3, Slc38a5, Slc25a1, Slc16a6	79212, 79213, 83612, 266733, 50719, 29485, 192208, 29743, 303772
auxiliary transport protein activity	0.00633787	7	2.23	Npy, Pias3, Lynx1_predicted, Hpcal4, Kcne1, Cacna2d3, Kcnmb1	24604, 83614, 300018, 50872, 25471, 306243, 29747
amino acid-polyamine transporter activity	0.00664153	5	1.22	Slc6a1, Slc6a11, Slc32a1, Slc12a8, Slc7a3	79212, 79213, 83612, 266733, 29485
polyamine transporter activity	0.00664153	5	1.22	Slc6a1, Slc6a11, Slc32a1, Slc12a8, Slc7a3	79212, 79213, 83612, 266733, 29485
potassium channel activity	0.00775144	10	4.11	Kcnh2, Kcnd3, Kcnh7, Grin1, Hcn2, Kcnq3, Grik3, Kcne1, Kcnmb1, Kcnh4	117018, 65195, 170739, 24408, 114244, 29682, 298521, 25471, 29747, 114032
cation:amino acid symporter activity	0.00789334	3	0.44	Slc6a1, Slc6a11, Slc32a1	79212, 79213, 83612
amino acid transporter activity	0.00957145	7	2.40	Slc6a1, Slc6a11, Slc32a1, Slc12a8, Slc7a5, Slc7a3, Slc38a5	79212, 79213, 83612, 266733, 50719, 29485, 192208

GO category "cellular_component"

Number of genes annotated in GO: 391
Number of significant GO groups found: 30

GO group	p-value	# genes (observed)	# genes (expected)	list of genes	GeneIDs
plasma membrane part	1.17736e-05	84	53.66	Arrb1, Itgad, Ap2a2, Cd81, Picalm, Slc28a1, Igf1r, Pak1, Clcnkb, Sdcbp, Faf1, Clta, Clcnk1, Gabrd, Eps15, Nrcam, Slc5a6, Adcy3, Cav2, Zfp384, Met, Grid2, Kcnh2, Negr1, Kcnd3, Notch2, Slc32a1, Vapb, Cd164, Cd24, Gja1, Cacnb4, Grin1, Cacna1b, Stk39, Calcrl, Rtn4rl2, Cd44, Slc34a3, Acvr1, Plec1, Hcn2, Vezt, Kcnq3, Has2, Grik3, Usp48, Mbp, P2rxl1, Alcam, Cxadr, Clcn2, Ifnar1_predicted, Kcne1, Cd47, Cd97, Slc12a4, Has3, Bcar1, Gnao, Bcap31, Cacna1f, Igsf1, Atp1b4, Gabra3, Slc9a3r1, Slc16a6, Lamp1, Cacna2d3, Lzts1, Fgfr1, B3gnt3_predicted, Ptch1, Axin1, Tmem8_predicted, Cacng4, Cltc, Cacnb1, Emp2, Cacna1g, Grin2a, Gabrb2, Clcn7, Kcnh4	25387, 64350, 81637, 25621, 89816, 116642, 25718, 29431, 79430, 83841, 140657, 83800, 79425, 29689, 313474, 497815, 170551, 64508, 363425, 171018, 24553, 79220, 117018, 59318, 65195, 29492, 83612, 60431, 83689, 25145, 24392, 58942, 24408, 257648, 54348, 25029, 311169, 25406, 246234, 79558, 64204, 114244, 299738, 29682, 25694, 298521, 362636, 24547, 25041, 79559, 89843, 29232, 288264, 25471, 29364, 361383, 29501, 266805, 25414, 50664, 293852, 114493, 302822, 84396, 24947, 59114, 303772, 25328, 306243, 266711, 79114, 290638, 89830, 79257, 303004, 140725, 54241, 50688, 360468, 29717, 24409, 25451, 29233,

					114032
cell fraction	3.05354e-05	59	34.73	Arrb1, Slc28a1, Clcnkb, Faah, Pde4b, Clcnk1, Ugcg, Slc5a6, Begain, Slc6a1, Met, Grid2, Kcnh2, Ppp3ca, Dlgap4, Cd164, Ndufs3_predicted, Ptges, Atp5g3, Lcn2, Cacnb4, Grin1, Stk39, Matk, Lynx1_predicted, Plec1, Tra1_predicted, Hcn2, Dgat1, Kcnq3, Cyp4f6, Ebna1bp2, Pafah2, Dlgap3, Casp9, Grik3, Atp2a2, Eif2b1, Alcam, Atp5j, Clcn2, Adcy5, Slc12a4, Bcar1, Gnao, Cyb5b, Gdi1, Slc16a6, Lamp1, Myo9b, Ppp2cb, Fgfr1, Edn1, Srd5a1, Sncb, Fbp1, Cacnb1, Cacna1g, Clcn7	25387, 116642, 79430, 29347, 24626, 79425, 83626, 170551, 79146, 79212, 24553, 79220, 117018, 24674, 286930, 83689, 295923, 59103, 114630, 170496, 58942, 24408, 54348, 60450, 300018, 64204, 362862, 114244, 84497, 29682, 266689, 114021, 313611, 286923, 58918, 298521, 29693, 64514, 79559, 94271, 29232, 64532, 29501, 25414, 50664, 80773, 25183, 303772, 25328, 25486, 24673, 79114, 24323, 24950, 113893, 24362, 50688, 29717, 29233
plasma membrane	6.09072e-05	98	68.07	Arrb1, Itgad, Ap2a2, Cd81, Picalm, Slc28a1, Igf1r, Pak1, Clcnkb, Sdcbp, Faf1, Clta, Clcnk1, Gabrd, Eps15, Pdpn, Nrcam, Slc5a6, Adcy3, Cav2, Zfp384, Arf5, Met, Grid2, Tacr1, Kcnh2, Negr1, Unc5c, Kcnd3, Notch2, Slc32a1, Vapb, Cd164, Cd24, Gja1, Cacnb4, Grin1, Cacna1b, Stk39, Stxbp1, Calcrl, Rtn4rl2, Cd44, Slc34a3, Acvr1, Lynx1_predicted, Plec1, Hcn2, Vezt, Kcnq3, Has2, Slc2a5, Grik3, Usp48, Trpv4, Mbp, P2rxl1, Alcam, Cxadr, Clcn2, Clic6, Ifnar1_predicted, Kcne1, Cd47, Adcy5, Rtn4r, Cd97, Slc12a4, Has3, Bcar1, Gnao, Slc7a5, Bcap31, Cacna1f, Igsf1, Atp1b4, Gabra3, Slc9a3r1, Slc16a6, Lamp1, Cacna2d3, Lzts1, Arf4, Fgfr1, Slc27a1, B3gnt3_predicted, Ptch1, Axin1, Tmem8_predicted, Cacng4, Cltc, Cacnb1, Emp2, Cacna1g, Grin2a, Gabrb2, Clcn7, Kcnh4	25387, 64350, 81637, 25621, 89816, 116642, 25718, 29431, 79430, 83841, 140657, 83800, 79425, 29689, 313474, 54320, 497815, 170551, 64508, 363425, 171018, 79117, 24553, 79220, 24807, 117018, 59318, 362049, 65195, 29492, 83612, 60431, 83689, 25145, 24392, 58942, 24408, 257648, 54348, 25558, 25029, 311169, 25406, 246234, 79558, 300018, 64204, 114244, 299738, 29682, 25694, 65197, 298521, 362636, 66026, 24547, 25041, 79559, 89843, 29232, 304081, 288264, 25471, 29364, 64532, 113912, 361383, 29501, 266805, 25414, 50664, 50719, 293852, 114493, 302822, 84396, 24947, 59114, 303772, 25328, 306243, 266711, 79120, 79114, 94172, 290638, 89830, 79257, 303004, 140725, 54241, 50688, 360468, 29717, 24409, 25451, 29233, 114032
voltage-gated calcium channel complex	8.49617e-05	7	1.12	Cacnb4, Cacna1b, Cacna1f, Cacna2d3, Cacng4, Cacnb1, Cacna1g	58942, 257648, 114493, 306243, 140725, 50688, 29717
membrane fraction	8.86912e-05	47	26.66	Arrb1, Slc28a1, Clcnkb, Faah, Clcnk1, Ugcg, Slc5a6, Begain, Slc6a1, Met, Grid2, Kcnh2, Dlgap4, Cd164, Ndufs3_predicted, Ptges, Atp5g3, Cacnb4, Grin1, Stk39, Lynx1_predicted, Tra1_predicted, Hcn2, Dgat1, Kcnq3, Cyp4f6, Ebna1bp2, Dlgap3, Grik3, Atp2a2, Alcam, Atp5j, Clcn2, Adcy5, Slc12a4, Bcar1, Gnao, Cyb5b, Gdi1, Slc16a6, Lamp1, Fgfr1, Srd5a1, Sncb, Cacnb1, Cacna1g, Clcn7	25387, 116642, 79430, 29347, 79425, 83626, 170551, 79146, 79212, 24553, 79220, 117018, 286930, 83689, 295923, 59103, 114630, 58942, 24408, 54348, 300018, 362862, 114244, 84497, 29682, 266689, 114021, 286923, 298521, 29693, 79559, 94271, 29232, 64532, 29501, 25414, 50664, 80773, 25183, 303772, 25328, 79114, 24950, 113893, 50688, 29717, 29233
cell projection	0.000155013	30	14.71	Pak1, Nrcam, Slc5a6, Slc6a1, Met, Akap3, Negr1, S100a6, Kcnd3, Plcg1, Fabp7, Grin1, Cacna1b, Slc34a3, Capzb, Dlgap3, Alcam, Tnk2, Clcn2, Bcar1, L1cam, Dnai2, Myo9b, Lzts1, Sncg, Sh3md2, Dbn1, Timp2, Cygb, Igf2bp1	29431, 497815, 170551, 79212, 24553, 312720, 59318, 85247, 65195, 25738, 80841, 24408, 257648, 246234, 298584, 286923, 79559, 303882, 29232, 25414, 50687, 360654, 25486, 266711, 64347,

					306417, 81653, 29543, 170520, 303477
intracellular part	0.000324736	222	188.55	Arrb1, Ap2a2, Bckdk, Ppp2r2d, Doc2a, Tbx6_predicted, Pak1, Bag3, Trpc2, Mgmt, Prc1_predicted, Ncoa2, Rab2, Sdcbp, Faf1, Tmod1, Clta, Kif1b, Pde4b, Anp32b, Atp6v1g1_predicted, Msc_predicted, Nfia, Ssbp3, Ugcg, Unc13b, Tdrd7, Slc5a6, Rock2, Glrx5_predicted, Yy1, Cav2, Ppp4r2_predicted, Prkag2, Zfp384, Arf5, Bhlhb2, Dctn1, Syn2, Casp2, Kcnh2, Akap3, Mrps35_predicted, Pparg, Reln, Abp1, Ptpn12, Bcl2l13_predicted, Prkab2, Gclm, Cxxc4, Cct3, Pias3, Ubqln4_predicted, Adar, Crabp2, Sf3b4, Ppp3ca, Plrg1, Lmo4, Pla2g12a_predicted, Cnn3, S100a6, Syt6, Pitx2, Sep15, Notch2, Plcg1, Tcfap2c, Hnf4a, Bpi, Arfgef2, Top1, Dnmt3b, Psap, Cd164, Prep, Gja1, Neurog3, Ndufs3_predicted, Ptges, Nfe2l2, Tmx2, Atp5g3, Lcn2, Cacnb4, Ubadc1, Cdk9, Psmd5_predicted, Hipk3, Grin1, Rpl35, Stk39, Stxbp1, Rbms1, Dgkz, Phpt1_predicted, Dlx2, Akap8, Aes, Hrsp12, Zbtb7a, Vps28_predicted, Ptbp1, Cand1, Cart1, Plec1, Tra1_predicted, Shmt2, Ywhaz, Tmpo, Fstl3, Suox, Fbxo7, Yeats4_predicted, Socs2, Ascl1, Cnot2, Cyp4f6, Capzb, Id3, Pou3f1, Ebna1bp2, Wnt4, Pafah2, Nt5c1a_predicted, Nppa, Casp9, Acot7, Acads, Atp2a2, Pxmp2, Dynll1, Tcf1, Erp29, Diablo, Eif2b1, Dscr1, Txnrd2, Bdh1, Tnk2, Gsk3b, Atp5j, Clcn2, Ap2m1, Sfrs10, Adcy5, Ctcf, Bcar1, Egln1, Glg1, Cyb5b, Siah1a, Mbtps1, Maf, Pkn1, Pdp2, Tomm20, Bcap31, Otc, Mid1, Birc4, Morf4l2, Rbbp7, Mig12, Atp1b4, Rgn, Btk, Slc25a1, Dnai2, Lamp1, Myo9b, Upf3a, Prkcd, Chdh, Lzts1, Ppp2cb, Arf4, Sncg, Slc27a1, Casp3, Mustn1, Vps36_predicted, Sh3bp5, Aadat, Rab3a, Tcfap2a_predicted, Kif27, Smad5, Sncb, Nfil3, Dek, Barx1_predicted, Nmt2, Ddx46, Gkap1, Ctsl, Fbp1, Hist1h1t, Exoc2, Uqcrfs1, Id4, Dbn1, Timp2, Axin1, Cygb, Sfrs2, Scpep1, Prm2, Cltc, Abca5, Smarcd2, Grb2, Hcrt, Ubtf, Kpnb1, Snip, Rpl23, Atp5g1, Acly, H3f3b, Pctp	25387, 81637, 29603, 246255, 65031, 365371, 29431, 293524, 64573, 25332, 308761, 83724, 65158, 83841, 140657, 25566, 83800, 117548, 24626, 170724, 298103, 312897, 25492, 84354, 83626, 64830, 85425, 170551, 25537, 362776, 24919, 363425, 297486, 373545, 171018, 79117, 79431, 29167, 29179, 64314, 117018, 312720, 297727, 25664, 24718, 65029, 117255, 312682, 64562, 29739, 83824, 295230, 83614, 310633, 81635, 29563, 295270, 24674, 60376, 362051, 362039, 54321, 85247, 60565, 54284, 113922, 29492, 25738, 362280, 25735, 296321, 296380, 64550, 444985, 25524, 83689, 83471, 24392, 60329, 295923, 59103, 83619, 295701, 114630, 170496, 58942, 362087, 362110, 296651, 83617, 24408, 296709, 54348, 25558, 362138, 81821, 296571, 296499, 116633, 29466, 65151, 117107, 300052, 29497, 117152, 25401, 64204, 362862, 299857, 25578, 25359, 114031, 81805, 366854, 299810, 84607, 64186, 299805, 266689, 298584, 25585, 192110, 114021, 84426, 313611, 313574, 24602, 58918, 26759, 64304, 29693, 29533, 58945, 24817, 117030, 288753, 64514, 266766, 50551, 117099, 303882, 84027, 94271, 29232, 116563, 117259, 64532, 83726, 25414, 308913, 29476, 80773, 140941, 89842, 54267, 29355, 246311, 266601, 293852, 25611, 54252, 63879, 317413, 83712, 404280, 84396, 25106, 367901, 29743, 360654, 25328, 25486, 361176, 170538, 290551, 266711, 24673, 79120, 64347, 94172, 25402, 290553, 290851, 117186, 29416, 25531, 306862, 246209, 59328, 113893, 114519, 306817, 364680, 291318, 245957, 361202, 25697, 24362, 24438, 171455, 291103, 291023, 81653, 29543, 79257, 170520, 494445, 114861, 25345, 54241, 286970, 83833, 81504, 25723, 25574, 24917, 56029, 29282, 29754, 24159, 117056, 29510
neuron projection	0.000325642	18	7.25	Pak1, Nrcam, Slc6a1, Negr1, Kcnd3, Plcg1, Grin1, Cacna1b, Dlgap3, Alcam, Tnk2, L1cam, Lzts1, Sncg, Dbn1, Timp2, Cygb, Igf2bp1	29431, 497815, 79212, 59318, 65195, 25738, 24408, 257648, 286923, 79559, 303882, 50687, 266711, 64347, 81653, 29543, 170520, 303477
protein complex	0.000831802	77	54.53	P4ha3, Arrb1, Itgad, Ap2a2, Bckdk, Ppp2r2d, Sdcbp, Faf1, Atp6v1g1_predicted, Yy1, Prkag2, Dctn1, Kcnh2,	361612, 25387, 64350, 81637, 29603, 246255, 83841, 140657, 298103, 24919, 373545, 29167,

				Mrps35_predicted, Pparg, Prkab2, Gclm, Cct3, Sf3b4, Ppp3ca, Lmo4, Pitx2, Tcfap2c, Hnf4a, Gja1, Ndufs3_predicted, Atp5g3, Cacnb4, Ubadc1, Cdk9, Psmd5_predicted, Grin1, Rpl35, Cacna1b, Cd44, Acvr1, Ptbp1, Hcn2, Fbxo7, Kcnq3, Capzb, Grik3, Abcb9, Dynll1, Tcf1, Eif2b1, Kcne1, Gnao, Pdp2, Tomm20, Cacna1f, Otc, Mid1, Morf4l2, Rbbp7, Atp6ap1, Dnai2, Cacna2d3, Ppp2cb, Tcfap2a_predicted, Hist1h1t, Exoc2, Uqcrfs1, Il12b, Cacng4, Sfrs2, Prm2, Cacnb1, Abca5, Smarcd2, Kpnb1, Rpl23, Cacna1g, Atp5g1, Acly, H3f3b, Kcnh4	117018, 297727, 25664, 64562, 29739, 295230, 295270, 24674, 362051, 54284, 362280, 25735, 24392, 295923, 114630, 58942, 362087, 362110, 296651, 24408, 296709, 257648, 25406, 79558, 29497, 114244, 366854, 29682, 298584, 298521, 63886, 58945, 24817, 64514, 25471, 50664, 246311, 266601, 114493, 25611, 54252, 317413, 83712, 83615, 360654, 306243, 24673, 306862, 24438, 171455, 291103, 64546, 140725, 494445, 25345, 50688, 286970, 83833, 24917, 29282, 29717, 29754, 24159, 117056, 114032
intrinsic to plasma membrane	0.000908178	57	37.70	Itgad, Cd81, Slc28a1, Igf1r, Clcnkb, Sdcbp, Clcnk1, Gabrd, Nrcam, Slc5a6, Adcy3, Cav2, Met, Grid2, Kcnh2, Negr1, Kcnd3, Notch2, Slc32a1, Vapb, Cd164, Cd24, Gja1, Grin1, Calcrl, Rtn4rl2, Acvr1, Hcn2, Vezt, Kcnq3, Has2, Grik3, P2rxl1, Alcam, Clcn2, Ifnar1_predicted, Kcne1, Cd47, Cd97, Slc12a4, Has3, Bcap31, Igsf1, Atp1b4, Gabra3, Slc16a6, Lamp1, Fgfr1, B3gnt3_predicted, Ptch1, Tmem8_predicted, Cacng4, Emp2, Grin2a, Gabrb2, Clcn7, Kcnh4	64350, 25621, 116642, 25718, 79430, 83841, 79425, 29689, 497815, 170551, 64508, 363425, 24553, 79220, 117018, 59318, 65195, 29492, 83612, 60431, 83689, 25145, 24392, 24408, 25029, 311169, 79558, 114244, 299738, 29682, 25694, 298521, 25041, 79559, 29232, 288264, 25471, 29364, 361383, 29501, 266805, 293852, 302822, 84396, 24947, 303772, 25328, 79114, 290638, 89830, 303004, 140725, 360468, 24409, 25451, 29233, 114032
coated pit	0.00102449	5	0.82	Ap2a2, Picalm, Clta, Eps15, Cltc	81637, 89816, 83800, 313474, 54241
cell soma	0.00108534	10	3.15	Negr1, Kcnd3, Fabp7, Cacna1b, Alcam, Tnk2, Cacna1f, Sncg, Timp2, Cygb	59318, 65195, 80841, 257648, 79559, 303882, 114493, 64347, 29543, 170520
intracellular	0.00125081	223	193.30	Arrb1, Ap2a2, Bckdk, Ppp2r2d, Doc2a, Tbx6_predicted, Pak1, Bag3, Trpc2, Mgmt, Prc1_predicted, Ncoa2, Rab2, Sdcbp, Faf1, Tmod1, Clta, Kif1b, Pde4b, Anp32b, Atp6v1g1_predicted, Msc_predicted, Nfia, Ssbp3, Ugcg, Unc13b, Tdrd7, Slc5a6, Rock2, Glrx5_predicted, Yy1, Cav2, Ppp4r2_predicted, Prkag2, Zfp384, Arf5, Bhlhb2, Dctn1, Syn2, Casp2, Kcnh2, Akap3, Mrps35_predicted, Pparg, Reln, Abp1, Ptpn12, Bcl2l13_predicted, Prkab2, Gclm, Cxxc4, Cct3, Pias3, Ubqln4_predicted, Adar, Crabp2, Sf3b4, Ppp3ca, Plrg1, Lmo4, Pla2g12a_predicted, Cnn3, S100a6, Syt6, Pitx2, Sep15, Notch2, Plcg1, Tcfap2c, Hnf4a, Bpi, Arfgef2, Top1, Dnmt3b, Psap, Cd164, Prep, Gja1, Neurog3, Ndufs3_predicted, Ptges, Nfe2l2, Tmx2, Atp5g3, Lcn2, Cacnb4, Ubadc1, Cdk9, Psmd5_predicted, Hipk3, Grin1, Rpl35, Stk39, Stxbp1, Rbms1, Dgkz, Phpt1_predicted, Dlx2, Akap8, Aes, Hrsp12, Zbtb7a, Vps28_predicted, Ptbp1, Cand1, Cart1, Plec1, Tra1_predicted, Shmt2, Ywhaz, Tmpo, Fstl3, Suox, Fbxo7, Yeats4_predicted, Socs2, Ascl1, Cnot2, Cyp4f6, Capzb, Id3, Pou3f1, Ebna1bp2, Wnt4, Pafah2, Nt5c1a_predicted, Nppa, Casp9, Acot7, Acads, Atp2a2, Pxmp2, Dynll1, Tcf1, Erp29, Diablo, Eif2b1, Dscr1, Txnrd2, Lrrc15, Bdh1, Tnk2, Gsk3b, Atp5j, Clcn2,	25387, 81637, 29603, 246255, 65031, 365371, 29431, 293524, 64573, 25332, 308761, 83724, 65158, 83841, 140657, 25566, 83800, 117548, 24626, 170724, 298103, 312897, 25492, 84354, 83626, 64830, 85425, 170551, 25537, 362776, 24919, 363425, 297486, 373545, 171018, 79117, 79431, 29167, 29179, 64314, 117018, 312720, 297727, 25664, 24718, 65029, 117255, 312682, 64562, 29739, 83824, 295230, 83614, 310633, 81635, 29563, 295270, 24674, 60376, 362051, 362039, 54321, 85247, 60565, 54284, 113922, 29492, 25738, 362280, 25735, 296321, 296380, 64550, 444985, 25524, 83689, 83471, 24392, 60329, 295923, 59103, 83619, 295701, 114630, 170496, 58942, 362087, 362110, 296651, 83617, 24408, 296709, 54348, 25558, 362138, 81821, 296571, 296499, 116633, 29466, 65151, 117107, 300052, 29497, 117152, 25401, 64204, 362862, 299857, 25578, 25359, 114031, 81805, 366854, 299810, 84607, 64186, 299805, 266689, 298584, 25585,

				Ap2m1, Sfrs10, Adcy5, Ctcf, Bcar1, Egln1, Glg1, Cyb5b, Siah1a, Mbtps1, Maf, Pkn1, Pdp2, Tomm20, Bcap31, Otc, Mid1, Birc4, Morf4l2, Rbbp7, Mig12, Atp1b4, Rgn, Btk, Slc25a1, Dnai2, Lamp1, Myo9b, Upf3a, Prkcd, Chdh, Lzts1, Ppp2cb, Arf4, Sncg, Slc27a1, Casp3, Mustn1, Vps36_predicted, Sh3bp5, Aadat, Rab3a, Tcfap2a_predicted, Kif27, Smad5, Sncb, Nfil3, Dek, Barx1_predicted, Nmt2, Ddx46, Gkap1, Ctsl, Fbp1, Hist1h1t, Exoc2, Uqcrfs1, Id4, Dbn1, Timp2, Axin1, Cygb, Sfrs2, Scpep1, Prm2, Cltc, Abca5, Smarcd2, Grb2, Hcrt, Ubtf, Kpnb1, Snip, Rpl23, Atp5g1, Acly, H3f3b, Pctp	192110, 114021, 84426, 313611, 313574, 24602, 58918, 26759, 64304, 29693, 29533, 58945, 24817, 117030, 288753, 64514, 266766, 50551, 246296, 117099, 303882, 84027, 94271, 29232, 116563, 117259, 64532, 83726, 25414, 308913, 29476, 80773, 140941, 89842, 54267, 29355, 246311, 266601, 293852, 25611, 54252, 63879, 317413, 83712, 404280, 84396, 25106, 367901, 29743, 360654, 25328, 25486, 361176, 170538, 290551, 266711, 24673, 79120, 64347, 94172, 25402, 290553, 290851, 117186, 29416, 25531, 306862, 246209, 59328, 113893, 114519, 306817, 364680, 291318, 245957, 361202, 25697, 24362, 24438, 171455, 291103, 291023, 81653, 29543, 79257, 170520, 494445, 114861, 25345, 54241, 286970, 83833, 81504, 25723, 25574, 24917, 56029, 29282, 29754, 24159, 117056, 29510
membrane-bound organelle	0.00231531	161	134.20	Ap2a2, Bckdk, Doc2a, Tbx6_predicted, Pak1, Trpc2, Mgmt, Prc1_predicted, Ncoa2, Rab2, Sdcbp, Faf1, Tmod1, Clta, Kif1b, Anp32b, Atp6v1g1_predicted, Msc_predicted, Nfia, Ssbp3, Unc13b, Tdrd7, Slc5a6, Glrx5_predicted, Yy1, Zfp384, Arf5, Bhlhb2, Syn2, Casp2, Kcnh2, Akap3, Mrps35_predicted, Pparg, Abp1, Bcl2l13_predicted, Cxxc4, Pias3, Ubqln4_predicted, Adar, Sf3b4, Plrg1, Lmo4, Pla2g12a_predicted, S100a6, Syt6, Pitx2, Sep15, Notch2, Tcfap2c, Hnf4a, Arfgef2, Top1, Dnmt3b, Psap, Cd164, Prep, Gja1, Neurog3, Ndufs3_predicted, Ptges, Nfe2l2, Tmx2, Atp5g3, Cdk9, Hipk3, Grin1, Stk39, Stxbp1, Rbms1, Dgkz, Dlx2, Akap8, Aes, Hrsp12, Zbtb7a, Vps28_predicted, Ptbp1, Cand1, Cart1, Tra1_predicted, Shmt2, Ywhaz, Tmpo, Fstl3, Suox, Yeats4_predicted, Ascl1, Cnot2, Cyp4f6, Id3, Pou3f1, Ebna1bp2, Wnt4, Nppa, Casp9, Acads, Atp2a2, Pxmp2, Tcf1, Erp29, Diablo, Dscr1, Txnrd2, Bdh1, Gsk3b, Atp5j, Ap2m1, Sfrs10, Ctcf, Glg1, Cyb5b, Siah1a, Mbtps1, Maf, Pkn1, Pdp2, Tomm20, Bcap31, Otc, Morf4l2, Rbbp7, Atp1b4, Rgn, Slc25a1, Dnai2, Lamp1, Upf3a, Chdh, Lzts1, Ppp2cb, Arf4, Mustn1, Vps36_predicted, Sh3bp5, Aadat, Rab3a, Tcfap2a_predicted, Smad5, Sncb, Nfil3, Dek, Barx1_predicted, Ddx46, Gkap1, Ctsl, Hist1h1t, Id4, Timp2, Axin1, Sfrs2, Prm2, Cltc, Abca5, Smarcd2, Grb2, Hcrt, Ubtf, Kpnb1, Rpl23, Atp5g1	81637, 29603, 65031, 365371, 29431, 64573, 25332, 308761, 83724, 65158, 83841, 140657, 25566, 83800, 117548, 170724, 298103, 312897, 25492, 84354, 64830, 85425, 170551, 362776, 24919, 171018, 79117, 79431, 29179, 64314, 117018, 312720, 297727, 25664, 65029, 312682, 83824, 83614, 310633, 81635, 295270, 60376, 362051, 362039, 85247, 60565, 54284, 113922, 29492, 362280, 25735, 296380, 64550, 444985, 25524, 83689, 83471, 24392, 60329, 295923, 59103, 83619, 295701, 114630, 362110, 83617, 24408, 54348, 25558, 362138, 81821, 296499, 116633, 29466, 65151, 117107, 300052, 29497, 117152, 25401, 362862, 299857, 25578, 25359, 114031, 81805, 299810, 64186, 299805, 266689, 25585, 192110, 114021, 84426, 24602, 58918, 64304, 29693, 29533, 24817, 117030, 288753, 266766, 50551, 117099, 84027, 94271, 116563, 117259, 83726, 29476, 80773, 140941, 89842, 54267, 29355, 246311, 266601, 293852, 25611, 317413, 83712, 84396, 25106, 29743, 360654, 25328, 361176, 290551, 266711, 24673, 79120, 290553, 290851, 117186, 29416, 25531, 306862, 59328, 113893, 114519, 306817, 364680, 245957, 361202, 25697, 24438, 291023, 29543, 79257, 494445, 25345, 54241, 286970, 83833, 81504, 25723, 25574, 24917, 29282,

					29754
synapse	0.00249997	12	4.70	Arrb1, Dtnb, Syn2, Slc32a1, Dlgap4, Grin1, Dlgap3, Usp48, L1cam, Lzts1, Axin1, Grin2a	25387, 362715, 29179, 83612, 286930, 24408, 286923, 362636, 50687, 266711, 79257, 24409
postsynaptic membrane	0.00254957	6	1.42	Arrb1, Dlgap4, Grin1, Dlgap3, Usp48, Grin2a	25387, 286930, 24408, 286923, 362636, 24409
integral to plasma membrane	0.00296523	54	37.14	Itgad, Cd81, Slc28a1, Igf1r, Clcnkb, Sdcbp, Clcnk1, Gabrd, Nrcam, Slc5a6, Adcy3, Cav2, Met, Grid2, Kcnh2, Kcnd3, Notch2, Slc32a1, Vapb, Cd164, Gja1, Grin1, Calcrl, Acvr1, Hcn2, Vezt, Kcnq3, Has2, Grik3, P2rxl1, Alcam, Clcn2, Ifnar1_predicted, Kcne1, Cd47, Cd97, Slc12a4, Has3, Bcap31, Igsf1, Atp1b4, Gabra3, Slc16a6, Lamp1, Fgfr1, B3gnt3_predicted, Ptch1, Tmem8_predicted, Cacng4, Emp2, Grin2a, Gabrb2, Clcn7, Kcnh4	64350, 25621, 116642, 25718, 79430, 83841, 79425, 29689, 497815, 170551, 64508, 363425, 24553, 79220, 117018, 65195, 29492, 83612, 60431, 83689, 24392, 24408, 25029, 79558, 114244, 299738, 29682, 25694, 298521, 25041, 79559, 29232, 288264, 25471, 29364, 361383, 29501, 266805, 293852, 302822, 84396, 24947, 303772, 25328, 79114, 290638, 89830, 303004, 140725, 360468, 24409, 25451, 29233, 114032
clathrin-coated vesicle	0.00320752	11	4.23	Ap2a2, Doc2a, Clta, Syn2, Syt6, Grin1, Ap2m1, Siah1a, Rab3a, Cltc, Hcrt	81637, 65031, 83800, 29179, 60565, 24408, 116563, 140941, 25531, 54241, 25723
clathrin coat of coated pit	0.00379289	3	0.35	Ap2a2, Clta, Cltc	81637, 83800, 54241
organelle lumen	0.00379578	35	21.87	Bckdk, Tdrd7, Yy1, Mrps35_predicted, Pparg, Lmo4, Pitx2, Sep15, Tcfap2c, Hnf4a, Ptges, Cdk9, Akap8, Ptbp1, Tra1_predicted, Suox, Yeats4_predicted, Ebna1bp2, Acads, Tcf1, Erp29, Bdh1, Atp5j, Mbtps1, Pdp2, Otc, Morf4l2, Rbbp7, Mustn1, Tcfap2a_predicted, Sfrs2, Prm2, Smarcd2, Ubtf, Rpl23	29603, 85425, 24919, 297727, 25664, 362051, 54284, 113922, 362280, 25735, 59103, 362110, 116633, 29497, 362862, 81805, 299810, 114021, 64304, 24817, 117030, 117099, 94271, 89842, 246311, 25611, 317413, 83712, 290553, 306862, 494445, 25345, 83833, 25574, 29282
membrane-enclosed lumen	0.00379578	35	21.87	Bckdk, Tdrd7, Yy1, Mrps35_predicted, Pparg, Lmo4, Pitx2, Sep15, Tcfap2c, Hnf4a, Ptges, Cdk9, Akap8, Ptbp1, Tra1_predicted, Suox, Yeats4_predicted, Ebna1bp2, Acads, Tcf1, Erp29, Bdh1, Atp5j, Mbtps1, Pdp2, Otc, Morf4l2, Rbbp7, Mustn1, Tcfap2a_predicted, Sfrs2, Prm2, Smarcd2, Ubtf, Rpl23	29603, 85425, 24919, 297727, 25664, 362051, 54284, 113922, 362280, 25735, 59103, 362110, 116633, 29497, 362862, 81805, 299810, 114021, 64304, 24817, 117030, 117099, 94271, 89842, 246311, 25611, 317413, 83712, 290553, 306862, 494445, 25345, 83833, 25574, 29282
intracellular membrane-bound organelle	0.00416968	159	134.03	Ap2a2, Bckdk, Doc2a, Tbx6_predicted, Pak1, Trpc2, Mgmt, Prc1_predicted, Ncoa2, Rab2, Sdcbp, Faf1, Tmod1, Clta, Kif1b, Anp32b, Atp6v1g1_predicted, Msc_predicted, Nfia, Ssbp3, Unc13b, Tdrd7, Glrx5_predicted, Yy1, Zfp384, Arf5, Bhlhb2, Syn2, Casp2, Kcnh2, Akap3, Mrps35_predicted, Pparg, Abp1, Bcl2l13_predicted, Cxxc4, Pias3, Ubqln4_predicted, Adar, Sf3b4, Plrg1, Lmo4, Pla2g12a_predicted, S100a6, Syt6, Pitx2, Sep15, Notch2, Tcfap2c, Hnf4a, Arfgef2, Top1, Dnmt3b, Psap, Cd164, Prep, Gja1, Neurog3, Ndufs3_predicted, Ptges, Nfe2l2, Tmx2, Atp5g3, Cdk9, Hipk3, Grin1, Stk39, Stxbp1, Rbms1, Dgkz, Dlx2, Akap8, Aes, Hrsp12, Zbtb7a, Vps28_predicted, Ptbp1, Cand1,	81637, 29603, 65031, 365371, 29431, 64573, 25332, 308761, 83724, 65158, 83841, 140657, 25566, 83800, 117548, 170724, 298103, 312897, 25492, 84354, 64830, 85425, 362776, 24919, 171018, 79117, 79431, 29179, 64314, 117018, 312720, 297727, 25664, 65029, 312682, 83824, 83614, 310633, 81635, 295270, 60376, 362051, 362039, 85247, 60565, 54284, 113922, 29492, 362280, 25735, 296380, 64550, 444985, 25524, 83689, 83471, 24392, 60329, 295923, 59103, 83619, 295701, 114630, 362110, 83617, 24408, 54348,

				Cart1, Tra1_predicted, Shmt2, Ywhaz, Tmpo, Fstl3, Suox, Yeats4_predicted, Ascl1, Cnot2, Cyp4f6, Id3, Pou3f1, Ebna1bp2, Wnt4, Nppa, Casp9, Acads, Atp2a2, Pxmp2, Tcf1, Erp29, Diablo, Dscr1, Txnrd2, Bdh1, Gsk3b, Atp5j, Ap2m1, Sfrs10, Ctcf, Glg1, Cyb5b, Siah1a, Mbtps1, Maf, Pkn1, Pdp2, Tomm20, Bcap31, Otc, Morf4l2, Rbbp7, Atp1b4, Rgn, Slc25a1, Dnai2, Lamp1, Upf3a, Chdh, Lzts1, Ppp2cb, Arf4, Mustn1, Vps36_predicted, Sh3bp5, Aadat, Rab3a, Tcfap2a_predicted, Smad5, Sncb, Nfil3, Dek, Barx1_predicted, Ddx46, Gkap1, Ctsl, Hist1h1t, Id4, Timp2, Axin1, Sfrs2, Prm2, Cltc, Abca5, Smarcd2, Hcrt, Ubtf, Kpnb1, Rpl23, Atp5g1	25558, 362138, 81821, 296499, 116633, 29466, 65151, 117107, 300052, 29497, 117152, 25401, 362862, 299857, 25578, 25359, 114031, 81805, 299810, 64186, 299805, 266689, 25585, 192110, 114021, 84426, 24602, 58918, 64304, 29693, 29533, 24817, 117030, 288753, 266766, 50551, 117099, 84027, 94271, 116563, 117259, 83726, 29476, 80773, 140941, 89842, 54267, 29355, 246311, 266601, 293852, 25611, 317413, 83712, 84396, 25106, 29743, 360654, 25328, 361176, 290551, 266711, 24673, 79120, 290553, 290851, 117186, 29416, 25531, 306862, 59328, 113893, 114519, 306817, 364680, 245957, 361202, 25697, 24438, 291023, 29543, 79257, 494445, 25345, 54241, 286970, 83833, 25723, 25574, 24917, 29282, 29754
cytoplasmic part	0.00422755	110	87.79	Arrb1, Ap2a2, Bckdk, Ppp2r2d, Doc2a, Pak1, Bag3, Trpc2, Rab2, Faf1, Tmod1, Clta, Kif1b, Pde4b, Atp6v1g1_predicted, Unc13b, Tdrd7, Glrx5_predicted, Cav2, Ppp4r2_predicted, Arf5, Syn2, Mrps35_predicted, Pparg, Abp1, Ptpn12, Bcl2l13_predicted, Gclm, Cxxc4, Cct3, Ubqln4_predicted, Ppp3ca, Pla2g12a_predicted, Syt6, Sep15, Arfgef2, Psap, Cd164, Prep, Gja1, Ndufs3_predicted, Atp5g3, Cacnb4, Ubadc1, Grin1, Rpl35, Stxbp1, Phpt1_predicted, Vps28_predicted, Plec1, Tra1_predicted, Shmt2, Ywhaz, Fstl3, Suox, Cyp4f6, Capzb, Wnt4, Nt5c1a_predicted, Nppa, Casp9, Acot7, Acads, Atp2a2, Pxmp2, Erp29, Diablo, Eif2b1, Txnrd2, Bdh1, Gsk3b, Atp5j, Ap2m1, Egln1, Glg1, Cyb5b, Siah1a, Mbtps1, Pkn1, Pdp2, Tomm20, Bcap31, Otc, Btk, Slc25a1, Lamp1, Myo9b, Chdh, Ppp2cb, Arf4, Slc27a1, Casp3, Vps36_predicted, Sh3bp5, Aadat, Rab3a, Sncb, Gkap1, Ctsl, Fbp1, Exoc2, Axin1, Scpep1, Cltc, Abca5, Hcrt, Rpl23, Atp5g1, Acly, Pctp	25387, 81637, 29603, 246255, 65031, 29431, 293524, 64573, 65158, 140657, 25566, 83800, 117548, 24626, 298103, 64830, 85425, 362776, 363425, 297486, 79117, 29179, 297727, 25664, 65029, 117255, 312682, 29739, 83824, 295230, 310633, 24674, 362039, 60565, 113922, 296380, 25524, 83689, 83471, 24392, 295923, 114630, 58942, 362087, 24408, 296709, 25558, 296571, 300052, 64204, 362862, 299857, 25578, 114031, 81805, 266689, 298584, 84426, 313574, 24602, 58918, 26759, 64304, 29693, 29533, 117030, 288753, 64514, 50551, 117099, 84027, 94271, 116563, 308913, 29476, 80773, 140941, 89842, 29355, 246311, 266601, 293852, 25611, 367901, 29743, 25328, 25486, 290551, 24673, 79120, 94172, 25402, 290851, 117186, 29416, 25531, 113893, 361202, 25697, 24362, 171455, 79257, 114861, 54241, 286970, 25723, 29282, 29754, 24159, 29510
insoluble fraction	0.0053564	5	1.16	Faah, Pde4b, Ppp3ca, Plec1, Clcn2	29347, 24626, 24674, 64204, 29232
intracellular organelle	0.00590317	178	153.57	Ap2a2, Bckdk, Doc2a, Tbx6_predicted, Pak1, Trpc2, Mgmt, Prc1_predicted, Ncoa2, Rab2, Sdcbp, Faf1, Tmod1, Clta, Kif1b, Anp32b, Atp6v1g1_predicted, Msc_predicted, Nfia, Ssbp3, Unc13b, Tdrd7, Slc5a6, Rock2, Glrx5_predicted, Yy1, Ppp4r2_predicted, Zfp384, Arf5, Bhlhb2, Dctn1, Syn2, Casp2, Kcnh2, Akap3, Mrps35_predicted, Pparg, Abp1, Bcl2l13_predicted, Cxxc4, Pias3, Ubqln4_predicted, Adar, Sf3b4, Plrg1, Lmo4, Pla2g12a_predicted, Cnn3, S100a6, Syt6, Pitx2, Sep15, Notch2, Tcfap2c, Hnf4a, Arfgef2, Top1, Dnmt3b, Psap, Cd164, Prep, Gja1,	81637, 29603, 65031, 365371, 29431, 64573, 25332, 308761, 83724, 65158, 83841, 140657, 25566, 83800, 117548, 170724, 298103, 312897, 25492, 84354, 64830, 85425, 170551, 25537, 362776, 24919, 297486, 171018, 79117, 79431, 29167, 29179, 64314, 117018, 312720, 297727, 25664, 65029, 312682, 83824, 83614, 310633, 81635, 295270, 60376, 362051, 362039, 54321, 85247, 60565, 54284, 113922, 29492, 362280, 25735,

				Neurog3, Ndufs3_predicted, Ptges, Nfe2l2, Tmx2, Atp5g3, Cdk9, Hipk3, Grin1, Rpl35, Stk39, Stxbp1, Rbms1, Dgkz, Dlx2, Akap8, Aes, Hrsp12, Zbtb7a, Vps28_predicted, Ptbp1, Cand1, Cart1, Tra1_predicted, Shmt2, Ywhaz, Tmpo, Fstl3, Suox, Yeats4_predicted, Ascl1, Cnot2, Cyp4f6, Capzb, Id3, Pou3f1, Ebna1bp2, Wnt4, Nppa, Casp9, Acads, Atp2a2, Pxmp2, Dynll1, Tcf1, Erp29, Diablo, Dscr1, Txnrd2, Bdh1, Gsk3b, Atp5j, Clcn2, Ap2m1, Sfrs10, Ctcf, Glg1, Cyb5b, Siah1a, Mbtps1, Maf, Pkn1, Pdp2, Tomm20, Bcap31, Otc, Mid1, Morf4l2, Rbbp7, Mig12, Atp1b4, Rgn, Btk, Slc25a1, Dnai2, Lamp1, Myo9b, Upf3a, Chdh, Lzts1, Ppp2cb, Arf4, Slc27a1, Mustn1, Vps36_predicted, Sh3bp5, Aadat, Rab3a, Tcfap2a_predicted, Kif27, Smad5, Sncb, Nfil3, Dek, Barx1_predicted, Ddx46, Gkap1, Ctsl, Hist1h1t, Id4, Dbn1, Timp2, Axin1, Sfrs2, Prm2, Cltc, Abca5, Smarcd2, Grb2, Hcrt, Ubtf, Kpnb1, Snip, Rpl23, Atp5g1, H3f3b	296380, 64550, 444985, 25524, 83689, 83471, 24392, 60329, 295923, 59103, 83619, 295701, 114630, 362110, 83617, 24408, 296709, 54348, 25558, 362138, 81821, 296499, 116633, 29466, 65151, 117107, 300052, 29497, 117152, 25401, 362862, 299857, 25578, 25359, 114031, 81805, 299810, 64186, 299805, 266689, 298584, 25585, 192110, 114021, 84426, 24602, 58918, 64304, 29693, 29533, 58945, 24817, 117030, 288753, 266766, 50551, 117099, 84027, 94271, 29232, 116563, 117259, 83726, 29476, 80773, 140941, 89842, 54267, 29355, 246311, 266601, 293852, 25611, 54252, 317413, 83712, 404280, 84396, 25106, 367901, 29743, 360654, 25328, 25486, 361176, 290551, 266711, 24673, 79120, 94172, 290553, 290851, 117186, 29416, 25531, 306862, 246209, 59328, 113893, 114519, 306817, 364680, 245957, 361202, 25697, 24438, 291023, 81653, 29543, 79257, 494445, 25345, 54241, 286970, 83833, 81504, 25723, 25574, 24917, 56029, 29282, 29754, 117056
synapse part	0.00591349	7	2.20	Arrb1, Dlgap4, Grin1, Dlgap3, Usp48, L1cam, Grin2a	25387, 286930, 24408, 286923, 362636, 50687, 24409
organelle	0.00622147	178	153.74	Ap2a2, Bckdk, Doc2a, Tbx6_predicted, Pak1, Trpc2, Mgmt, Prc1_predicted, Ncoa2, Rab2, Sdcbp, Faf1, Tmod1, Clta, Kif1b, Anp32b, Atp6v1g1_predicted, Msc_predicted, Nfia, Ssbp3, Unc13b, Tdrd7, Slc5a6, Rock2, Glrx5_predicted, Yy1, Ppp4r2_predicted, Zfp384, Arf5, Bhlhb2, Dctn1, Syn2, Casp2, Kcnh2, Akap3, Mrps35_predicted, Pparg, Abp1, Bcl2l13_predicted, Cxxc4, Pias3, Ubqln4_predicted, Adar, Sf3b4, Plrg1, Lmo4, Pla2g12a_predicted, Cnn3, S100a6, Syt6, Pitx2, Sep15, Notch2, Tcfap2c, Hnf4a, Arfgef2, Top1, Dnmt3b, Psap, Cd164, Prep, Gja1, Neurog3, Ndufs3_predicted, Ptges, Nfe2l2, Tmx2, Atp5g3, Cdk9, Hipk3, Grin1, Rpl35, Stk39, Stxbp1, Rbms1, Dgkz, Dlx2, Akap8, Aes, Hrsp12, Zbtb7a, Vps28_predicted, Ptbp1, Cand1, Cart1, Tra1_predicted, Shmt2, Ywhaz, Tmpo, Fstl3, Suox, Yeats4_predicted, Ascl1, Cnot2, Cyp4f6, Capzb, Id3, Pou3f1, Ebna1bp2, Wnt4, Nppa, Casp9, Acads, Atp2a2, Pxmp2, Dynll1, Tcf1, Erp29, Diablo, Dscr1, Txnrd2, Bdh1, Gsk3b, Atp5j, Clcn2, Ap2m1, Sfrs10, Ctcf, Glg1, Cyb5b, Siah1a, Mbtps1, Maf, Pkn1, Pdp2, Tomm20, Bcap31, Otc, Mid1, Morf4l2, Rbbp7, Mig12, Atp1b4, Rgn, Btk, Slc25a1, Dnai2, Lamp1, Myo9b, Upf3a, Chdh, Lzts1, Ppp2cb, Arf4, Slc27a1, Mustn1, Vps36_predicted, Sh3bp5,	81637, 29603, 65031, 365371, 29431, 64573, 25332, 308761, 83724, 65158, 83841, 140657, 25566, 83800, 117548, 170724, 298103, 312897, 25492, 84354, 64830, 85425, 170551, 25537, 362776, 24919, 297486, 171018, 79117, 79431, 29167, 29179, 64314, 117018, 312720, 297727, 25664, 65029, 312682, 83824, 83614, 310633, 81635, 295270, 60376, 362051, 362039, 54321, 85247, 60565, 54284, 113922, 29492, 362280, 25735, 296380, 64550, 444985, 25524, 83689, 83471, 24392, 60329, 295923, 59103, 83619, 295701, 114630, 362110, 83617, 24408, 296709, 54348, 25558, 362138, 81821, 296499, 116633, 29466, 65151, 117107, 300052, 29497, 117152, 25401, 362862, 299857, 25578, 25359, 114031, 81805, 299810, 64186, 299805, 266689, 298584, 25585, 192110, 114021, 84426, 24602, 58918, 64304, 29693, 29533, 58945, 24817, 117030, 288753, 266766, 50551, 117099, 84027, 94271, 29232, 116563, 117259, 83726, 29476, 80773, 140941,

				Aadat, Rab3a, Tcfap2a_predicted, Kif27, Smad5, Sncb, Nfil3, Dek, Barx1_predicted, Ddx46, Gkap1, Ctsl, Hist1h1t, Id4, Dbn1, Timp2, Axin1, Sfrs2, Prm2, Cltc, Abca5, Smarcd2, Grb2, Hcrt, Ubtf, Kpnb1, Snip, Rpl23, Atp5g1, H3f3b	89842, 54267, 29355, 246311, 266601, 293852, 25611, 54252, 317413, 83712, 404280, 84396, 25106, 367901, 29743, 360654, 25328, 25486, 361176, 290551, 266711, 24673, 79120, 94172, 290553, 290851, 117186, 29416, 25531, 306862, 246209, 59328, 113893, 114519, 306817, 364680, 245957, 361202, 25697, 24438, 291023, 81653, 29543, 79257, 494445, 25345, 54241, 286970, 83833, 81504, 25723, 25574, 24917, 56029, 29282, 29754, 117056
endoplasmic reticulum lumen	0.00644652	4	0.78	Sep15, Tra1_predicted, Erp29, Mbtps1	113922, 362862, 117030, 89842
vesicle	0.00767562	20	11.09	Ap2a2, Doc2a, Clta, Slc5a6, Syn2, Cxxc4, Syt6, Grin1, Fstl3, Erp29, Ap2m1, Siah1a, Pkn1, Btk, Slc27a1, Rab3a, Axin1, Cltc, Grb2, Hcrt	81637, 65031, 83800, 170551, 29179, 83824, 60565, 24408, 114031, 117030, 116563, 140941, 29355, 367901, 94172, 25531, 79257, 54241, 81504, 25723
cytosol	0.00904637	28	17.47	Arrb1, Ppp2r2d, Pak1, Bag3, Faf1, Pde4b, Pparg, Ptpn12, Cct3, Ubqln4_predicted, Ppp3ca, Arfgef2, Prep, Stxbp1, Phpt1_predicted, Vps28_predicted, Tra1_predicted, Suox, Nt5c1a_predicted, Casp9, Acot7, Gsk3b, Egln1, Ppp2cb, Casp3, Fbp1, Scpep1, Pctp	25387, 246255, 29431, 293524, 140657, 24626, 25664, 117255, 295230, 310633, 24674, 296380, 83471, 25558, 296571, 300052, 362862, 81805, 313574, 58918, 26759, 84027, 308913, 24673, 25402, 24362, 114861, 29510

Extraction Options

Sequence Extraction

Selected elements with start/end ± bp
Complete sequence reverse complement
in FASTA format GenBank format

GeneID Extraction

Excel Extraction

Compare results

Enter GeneIDs

(separated by spaces, returns, or commas)

Further Evaluation of Matches

Search PubMed for

("gene name") AND
(where "gene name" is automatically extracted from the description lines)
Note: This works only for annotated genomic DNA sequences from eukaryotes!

For comments, questions, or bug reports, please contact support@genomatix.de.

© Genomatix Software GmbH 1998-2008 - All rights reserved.

GEMS Launcher 4.6.1

License Agreement
