# Supplementary material for: Comparison of Gene Expression Profile in Embryonic Mesencephalon and Neuronal Primary Cultures
Source: PLoS One. 2009 Mar 23;4(3):e4977. doi: 10.1371/journal.pone.0004977 (PMC2654915; doi:10.1371/journal.pone.0004977)
Supplement: Table S5 — Summary of the Genomatix ModelInspector analysis for V$NEUR-V$NR2F. Rattus norvegicus promoter sequences showing the V$NEUR-V$NR2F module. For each match, the sequence annotation, the position of the module, and the strand are indicated. Over-represented Geneontology terms are also shown starting from page 57. (0.35 MB PDF) [file pone.0004977.s006.pdf]

GEMS Launcher Task: *ModelInspector*: Search for user-defined models
working on Rat Promoters

Output overview of ModelInspector matches (788 matches)

go to:[Output overview] [Detailed output] [Statistics]

ModelInspector Release 5.5 February 2008 **Wed May 28 11:10:54 2008**

Solution parameters:

**Sequence file:**	Rat Promoters
**Models:**	User-defined/neur-nr2f.model
**Strand(s) searched:**	both strands
**Threshold for number of elements:**	100.0 % (2 of 2 elements)
**Output sorted by:**	match positions on the sequences
**Maximum number of matches:**	1000

Match List:

Sequence	Model Name	Position	Strand	Select Match
**GXP_1410473** [GXP_1410473] (1 - 657)<br>**Mrgprf**, GXL_703, GeneID: 266762, Rattus norvegicus chr. 1<br>MAS-related GPR, member F	neur-nr2f	6 - 120	(+)	
**GXP_1098** [GXP_1098] (1 - 602)<br>**Tacc2**, GXL_836, GeneID: 309025, Rattus norvegicus chr. 1<br>transforming, acidic coiled-coil containing protein 2	neur-nr2f	209 - 336	(+)	
**GXP_1115** [GXP_1115] (1 - 602)<br>**Spon1**, GXL_853, GeneID: 64456, Rattus norvegicus chr. 1	neur-nr2f	399 - 282	(-)	

spondin 1

**GXP_1409941** [GXP_1409941] (1 - 604)<br>**Pde2a**, GXL_980, GeneID: 81743, Rattus norvegicus chr. 1<br>phosphodiesterase 2A, cGMP-stimulated	neur-nr2f	303 - 440	(+)
**GXP_1287** [GXP_1287] (1 - 1290)<br>**Tbx6_predicted**, GXL_1023, GeneID: 365371, Rattus norvegicus chr. 1<br>T-box 6 (predicted)	neur-nr2f	878 - 736	(-)
**GXP_1410268** [GXP_1410268] (1 - 602)<br>**Hsd3b7**, GXL_1036, GeneID: 246211, Rattus norvegicus chr. 1<br>hydroxy-delta-5-steroid dehydrogenase, 3 beta- and steroid delta-isomerase 7	neur-nr2f	460 - 326	(-)
**GXP_1392** [GXP_1392] (1 - 760)<br>**Ctsc**, GXL_1127, GeneID: 25423, Rattus norvegicus chr. 1<br>cathepsin C	neur-nr2f	585 - 731	(+)
**GXP_517844** [GXP_517844] (1 - 601)<br>**Clcn6_predicted**, GXL_1194, GeneID: 295586, Rattus norvegicus chr. 5<br>chloride channel 6 (predicted)	neur-nr2f	553 - 427	(-)
**GXP_1509** [GXP_1509] (1 - 601)<br>**Spink4**, GXL_1242, GeneID: 408233, Rattus norvegicus chr. 5<br>Kazal type serine protease inhibitor 4	neur-nr2f	406 - 547	(+)
**GXP_1570** [GXP_1570] (1 - 1176)<br>**Ttc22_predicted**, GXL_1300, GeneID: 298300, Rattus norvegicus chr. 5<br>tetratricopeptide repeat domain 22 (predicted)	neur-nr2f	308 - 158	(-)
**GXP_1416132** [GXP_1416132] (1 - 972)<br>**Pde4b**, GXL_1405, GeneID: 24626, Rattus norvegicus chr. 5<br>phosphodiesterase 4B, cAMP specific	neur-nr2f	780 - 658	(-)
**GXP_1416712** [GXP_1416712] (1 - 601)<br>**Tnfrsf1b**, GXL_1489, GeneID: 156767, Rattus norvegicus chr. 5<br>tumor necrosis factor receptor superfamily, member 1b	neur-nr2f	205 - 59	(-)
**GXP_1416247** [GXP_1416247] (1 - 602)<br>**Pomgnt1**, GXL_1501, GeneID: 362567, Rattus norvegicus chr. 5<br>protein O-linked mannose beta1, 2-N-acetylglucosaminyltransferase	neur-nr2f	585 - 457	(-)
**GXP_1417140** [GXP_1417140] (1 - 602)<br>**Myt1l**, GXL_10329, GeneID: 116668, Rattus norvegicus chr. 6<br>myelin transcription factor 1-like	neur-nr2f	125 - 243	(+)
**GXP_1417109** [GXP_1417109] (1 - 602)<br>**Rock2**, GXL_10344, GeneID: 25537, Rattus norvegicus chr. 6<br>Rho-associated coiled-coil forming kinase 2	neur-nr2f	221 - 80	(-)
**GXP_1417114** [GXP_1417114] (1 - 1184)<br>**Hpcal1**, GXL_10377, GeneID: 50871, Rattus norvegicus chr. 6<br>hippocalcin-like 1	neur-nr2f	299 - 449	(+)

**GXP_1417023** [GXP_1417023] (1 - 1012)<br>**Zfp513**, GXL_10482, GeneID: 313913, Rattus norvegicus chr. 6<br>zinc finger protein 513	neur-nr2f	996 - 863	(-)
**GXP_1415315** [GXP_1415315] (1 - 676)<br>**M6pr**, GXL_15685, GeneID: 312689, Rattus norvegicus chr. 4<br>mannose-6-phosphate receptor, cation dependent	neur-nr2f	445 - 562	(+)
**GXP_19472** [GXP_19472] (1 - 602)<br>**Cntn4**, GXL_15731, GeneID: 116658, Rattus norvegicus chr. 4<br>contactin 4	neur-nr2f	121 - 272	(+)
**GXP_1415355** [GXP_1415355] (1 - 602)<br>**Zfp384**, GXL_15781, GeneID: 171018, Rattus norvegicus chr. 4<br>zinc finger protein 384	neur-nr2f	144 - 10	(-)
**GXP_19668** [GXP_19668] (1 - 601)<br>**Fzd1**, GXL_15923, GeneID: 58868, Rattus norvegicus chr. 4<br>frizzled homolog 1 (Drosophila)	neur-nr2f	161 - 18	(-)
**GXP_1415164** [GXP_1415164] (1 - 602)<br>**Itpr1**, GXL_16011, GeneID: 25262, Rattus norvegicus chr. 4<br>inositol 1,4,5-triphosphate receptor 1	neur-nr2f	327 - 192	(-)
**GXP_1414838** [GXP_1414838] (1 - 602)<br>**Scospondin**, GXL_16058, GeneID: 474348, Rattus norvegicus chr. 4<br>subcommissural organ spondin	neur-nr2f	17 - 135	(+)
**GXP_1414941** [GXP_1414941] (1 - 602)<br>**Eif2ak3**, GXL_16068, GeneID: 29702, Rattus norvegicus chr. 4<br>eukaryotic translation initiation factor 2 alpha kinase 3	neur-nr2f	293 - 167	(-)
**GXP_1415331** [GXP_1415331] (1 - 637)<br>**C1rl**, GXL_16081, GeneID: 408246, Rattus norvegicus chr. 4<br>complement component 1, r subcomponent-like	neur-nr2f	420 - 294	(-)
**GXP_19832** [GXP_19832] (1 - 637)<br>**Spsb2**, GXL_16086, GeneID: 297592, Rattus norvegicus chr. 4<br>splA/ryanodine receptor domain and SOCS box containing 2	neur-nr2f	243 - 116	(-)
**GXP_1415343** [GXP_1415343] (1 - 1155)<br>**Spsb2**, GXL_16086, GeneID: 297592, Rattus norvegicus chr. 4<br>splA/ryanodine receptor domain and SOCS box containing 2	neur-nr2f	879 - 757	(-)
**GXP_22689** [GXP_22689] (1 - 1279)<br>**St7l**, GXL_18704, GeneID: 295344, Rattus norvegicus chr. 2<br>suppression of tumorigenicity 7-like	neur-nr2f	1167 - 1052	(-)
**GXP_22786** [GXP_22786] (1 - 605)<br>**S100a11**, GXL_18800, GeneID: 445415, Rattus norvegicus chr. 2<br>S100 calcium binding protein A11 (calizzarin)	neur-nr2f	360 - 476	(+)
	neur-nr2f	395 - 250	(-)

**GXP_1412808** [GXP_1412808] (1 - 634)			
**Lmo4**, GXL_18834, GeneID: 362051, Rattus norvegicus chr. 2			
LIM domain only 4			
**GXP_1412562** [GXP_1412562] (1 - 602)			
**Kcnd3**, GXL_19014, GeneID: 65195, Rattus norvegicus chr. 2	neur-nr2f	236 - 369	(+)
potassium voltage gated channel, Shal-related family, member 3			
**GXP_1412480** [GXP_1412480] (1 - 602)			
**Notch2**, GXL_19017, GeneID: 29492, Rattus norvegicus chr. 2	neur-nr2f	328 - 473	(+)
notch gene homolog 2 (Drosophila)			
**GXP_1412321** [GXP_1412321] (1 - 685)			
**Arhgef2**, GXL_19023, GeneID: 310635, Rattus norvegicus chr. 2	neur-nr2f	351 - 471	(+)
rho/rac guanine nucleotide exchange factor (GEF) 2			
**GXP_25339** [GXP_25339] (1 - 601)			
**Smgb**, GXL_20988, GeneID: 54303, Rattus norvegicus chr. 3	neur-nr2f	24 - 170	(+)
neonatal submandibular gland protein B			
**GXP_25362** [GXP_25362] (1 - 1338)			
**Slc32a1**, GXL_21010, GeneID: 83612, Rattus norvegicus chr. 3	neur-nr2f	1167 - 1038	(-)
solute carrier family 32 (GABA vesicular transporter), member 1			
**GXP_25430** [GXP_25430] (1 - 733)			
**Zgpat**, GXL_21078, GeneID: 296478, Rattus norvegicus chr. 3	neur-nr2f	244 - 397	(+)
zinc finger, CCCH-type with G patch domain			
**GXP_25462** [GXP_25462] (1 - 929)			
**Bwk1**, GXL_21110, GeneID: 296466, Rattus norvegicus chr. 3	neur-nr2f	501 - 626	(+)
Bwk1 leukemia-related gene			
**GXP_1414187** [GXP_1414187] (1 - 675)			
**Dlgap4**, GXL_21115, GeneID: 286930, Rattus norvegicus chr. 3	neur-nr2f	255 - 399	(+)
discs, large homolog-associated protein 4 (Drosophila)			
**GXP_1413623** [GXP_1413623] (1 - 610)			
**Rcn1_predicted**, GXL_37424, GeneID: 362182, Rattus norvegicus chr. 3	neur-nr2f	351 - 466	(+)
reticulocalbin 1 (predicted)			
**GXP_45322** [GXP_45322] (1 - 726)			
**Pscdbp**, GXL_37470, GeneID: 311047, Rattus norvegicus chr. 3	neur-nr2f	515 - 371	(-)
pleckstrin homology, Sec7 and coiled-coil domains, binding protein			
**GXP_45359** [GXP_45359] (1 - 601)			
**Olr662_predicted**, GXL_37506, GeneID: 405948, Rattus norvegicus chr. 3	neur-nr2f	249 - 96	(-)
olfactory receptor 662 (predicted)			
**GXP_45387** [GXP_45387] (1 - 1053)			
**C8g_predicted**, GXL_37533, GeneID: 296545, Rattus norvegicus chr. 3	neur-nr2f	850 - 725	(-)
complement component 8, gamma polypeptide (predicted)			
	neur-nr2f	84 - 217	(+)

**GXP_1412908** [GXP_1412908] (1 - 602)<br>**Slc34a3**, GXL_37660, GeneID: 246234, Rattus norvegicus chr. 3<br>solute carrier family 34 (sodium phosphate), member 3			
**GXP_1413233** [GXP_1413233] (1 - 602)<br>**Acvr1**, GXL_37664, GeneID: 79558, Rattus norvegicus chr. 3<br>activin A receptor, type 1	neur-nr2f	543 - 404	(-)
**GXP_1413535** [GXP_1413535] (1 - 602)<br>**Cry2**, GXL_37674, GeneID: 170917, Rattus norvegicus chr. 3<br>cryptochrome 2 (photolyase-like)	neur-nr2f	471 - 589	(+)
**GXP_45580** [GXP_45580] (1 - 706)<br>**Ptgds**, GXL_37726, GeneID: 25526, Rattus norvegicus chr. 3<br>prostaglandin D2 synthase	neur-nr2f	234 - 88	(-)
	neur-nr2f	646 - 517	(-)
**GXP_45606** [GXP_45606] (1 - 601)<br>**Olr552_predicted**, GXL_37752, GeneID: 405943, Rattus norvegicus chr. 3<br>olfactory receptor 552 (predicted)	neur-nr2f	353 - 233	(-)
**GXP_52118** [GXP_52118] (1 - 601)<br>**Olr1078**, GXL_42857, GeneID: 299593, Rattus norvegicus chr. 7<br>olfactory receptor 1078	neur-nr2f	507 - 381	(-)
**GXP_1169854** [GXP_1169854] (1 - 601)<br>**Map2k2**, GXL_42896, GeneID: 58960, Rattus norvegicus chr. 7<br>mitogen activated protein kinase kinase 2	neur-nr2f	309 - 177	(-)
**GXP_1418378** [GXP_1418378] (1 - 602)<br>**Lynx1_predicted**, GXL_42911, GeneID: 300018, Rattus norvegicus chr. 7<br>Ly6/neurotoxin 1 (predicted)	neur-nr2f	328 - 449	(+)
**GXP_1169756** [GXP_1169756] (1 - 601)<br>**Mbc2**, GXL_43000, GeneID: 29579, Rattus norvegicus chr. 7<br>membrane bound C2 domain containing protein	neur-nr2f	108 - 238	(+)
**GXP_52269** [GXP_52269] (1 - 825)<br>**Myf6**, GXL_43006, GeneID: 25714, Rattus norvegicus chr. 7<br>myogenic factor 6	neur-nr2f	122 - 273	(+)
**GXP_52299** [GXP_52299] (1 - 601)<br>**Olr962_predicted**, GXL_43036, GeneID: 302045, Rattus norvegicus chr. 7<br>olfactory receptor 962 (predicted)	neur-nr2f	195 - 346	(+)
**GXP_1417813** [GXP_1417813] (1 - 603)<br>**Eef2**, GXL_43134, GeneID: 29565, Rattus norvegicus chr. 7<br>eukaryotic translation elongation factor 2	neur-nr2f	353 - 503	(+)
**GXP_52406** [GXP_52406] (1 - 601)<br>**Olr951_predicted**, GXL_43143, GeneID: 288879, Rattus norvegicus chr. 7<br>olfactory receptor 951 (predicted)	neur-nr2f	195 - 346	(+)
	neur-nr2f	166 - 294	(+)

**GXP_52409** [GXP_52409] (1 - 601)<br>**Giot1**, GXL_43146, GeneID: 171090, Rattus norvegicus chr. 7<br>gonadotropin inducible ovarian transcription factor 1			
**GXP_52430** [GXP_52430] (1 - 606)<br>**Fgf22**, GXL_43167, GeneID: 170579, Rattus norvegicus chr. 7<br>fibroblast growth factor 22	neur-nr2f	33 - 154	(+)
**GXP_1418278** [GXP_1418278] (1 - 611)<br>**Klf10**, GXL_43186, GeneID: 81813, Rattus norvegicus chr. 7<br>Kruppel-like factor 10	neur-nr2f	486 - 371	(-)
**GXP_52471** [GXP_52471] (1 - 603)<br>**Rdh7**, GXL_43208, GeneID: 360420, Rattus norvegicus chr. 7<br>retinol dehydrogenase 7	neur-nr2f	540 - 423	(-)
**GXP_52531** [GXP_52531] (1 - 721)<br>**Olr908_predicted**, GXL_43268, GeneID: 288806, Rattus norvegicus chr. 7<br>olfactory receptor 908 (predicted)	neur-nr2f	673 - 524	(-)
**GXP_1417835** [GXP_1417835] (1 - 602)<br>**Csnk1g2**, GXL_43324, GeneID: 65278, Rattus norvegicus chr. 7<br>casein kinase 1, gamma 2	neur-nr2f	360 - 207	(-)
**GXP_1416319** [GXP_1416319] (1 - 601)<br>**Slc2a1**, GXL_48346, GeneID: 24778, Rattus norvegicus chr. 5<br>solute carrier family 2 (facilitated glucose transporter), member 1	neur-nr2f	67 - 220	(+)
**GXP_59052** [GXP_59052] (1 - 1110)<br>**Pla2g2a**, GXL_48373, GeneID: 29692, Rattus norvegicus chr. 5<br>phospholipase A2, group IIA (platelets, synovial fluid)	neur-nr2f	701 - 566	(-)
**GXP_1416352** [GXP_1416352] (1 - 602)<br>**Rims3**, GXL_48441, GeneID: 65025, Rattus norvegicus chr. 5<br>regulating synaptic membrane exocytosis 3	neur-nr2f	297 - 159	(-)
**GXP_1416731** [GXP_1416731] (1 - 602)<br>**Frap1**, GXL_48481, GeneID: 56718, Rattus norvegicus chr. 5<br>FK506 binding protein 12-rapamycin associated protein 1	neur-nr2f	233 - 104	(-)
**GXP_59178** [GXP_59178] (1 - 679)<br>**Nt5c1a_predicted**, GXL_48497, GeneID: 313574, Rattus norvegicus chr. 5<br>5'-nucleotidase, cytosolic IA (predicted)	neur-nr2f	143 - 279	(+)
**GXP_59197** [GXP_59197] (1 - 601)<br>**Olr858_predicted**, GXL_48516, GeneID: 366457, Rattus norvegicus chr. 5<br>olfactory receptor 858 (predicted)	neur-nr2f	56 - 176	(+)
**GXP_59212** [GXP_59212] (1 - 683)<br>**Rpl22**, GXL_48531, GeneID: 81768, Rattus norvegicus chr. 5<br>ribosomal protein L22	neur-nr2f	234 - 383	(+)
	neur-nr2f	50 - 180	(+)

**GXP_59216** [GXP_59216] (1 - 813)<br>**Casp9**, GXL_48535, GeneID: 58918, Rattus norvegicus chr. 5<br>caspase 9			
**GXP_59262** [GXP_59262] (1 - 722)<br>**Hes5**, GXL_48581, GeneID: 79225, Rattus norvegicus chr. 5<br>hairy and enhancer of split 5 (Drosophila)	neur-nr2f	18 - 171	(+)
**GXP_59275** [GXP_59275] (1 - 601)<br>**Htr1d**, GXL_48594, GeneID: 25323, Rattus norvegicus chr. 5<br>5-hydroxytryptamine (serotonin) receptor 1D	neur-nr2f	255 - 391	(+)
**GXP_1150227** [GXP_1150227] (1 - 836)<br>**Camkk2**, GXL_50823, GeneID: 83506, Rattus norvegicus chr. 12<br>calcium/calmodulin-dependent protein kinase kinase 2, beta	neur-nr2f	12 - 159	(+)
**GXP_1404535** [GXP_1404535] (1 - 602)<br>**Zcchc8_predicted**, GXL_50844, GeneID: 288661, Rattus norvegicus chr. 12<br>zinc finger, CCHC domain containing 8 (predicted)	neur-nr2f	348 - 501	(+)
**GXP_61722** [GXP_61722] (1 - 601)<br>**Dynll1**, GXL_50869, GeneID: 58945, Rattus norvegicus chr. 12<br>dynein light chain LC8-type 1	neur-nr2f	47 - 184	(+)
**GXP_62880** [GXP_62880] (1 - 740)<br>**Cyb5**, GXL_52013, GeneID: 64001, Rattus norvegicus chr. 18<br>cytochrome b-5	neur-nr2f	512 - 633	(+)
**GXP_1404013** [GXP_1404013] (1 - 625)<br>**Hrasls_predicted**, GXL_60599, GeneID: 288025, Rattus norvegicus chr. 11<br>HRAS-like suppressor (predicted)	neur-nr2f	531 - 408	(-)
**GXP_73072** [GXP_73072] (1 - 601)<br>**Tagln3**, GXL_60758, GeneID: 63837, Rattus norvegicus chr. 11<br>transgelin 3	neur-nr2f	290 - 411	(+)
**GXP_852696** [GXP_852696] (1 - 1236)<br>**Tagln3**, GXL_60758, GeneID: 63837, Rattus norvegicus chr. 11<br>transgelin 3	neur-nr2f	925 - 1046	(+)
**GXP_73168** [GXP_73168] (1 - 955)<br>**Tnk2**, GXL_60853, GeneID: 303882, Rattus norvegicus chr. 11<br>tyrosine kinase, non-receptor, 2	neur-nr2f	20 - 146	(+)
**GXP_73215** [GXP_73215] (1 - 839)<br>**Clcn2**, GXL_60900, GeneID: 29232, Rattus norvegicus chr. 11<br>chloride channel 2	neur-nr2f	454 - 310	(-)
**GXP_1404086** [GXP_1404086] (1 - 602)<br>**Ap2m1**, GXL_60928, GeneID: 116563, Rattus norvegicus chr. 11<br>adaptor-related protein complex 2, mu 1 subunit	neur-nr2f	107 - 254	(+)
	neur-nr2f	131 - 280	(+)

**GXP_73264** [GXP_73264] (1 - 601)<br>**Kcnj6**, GXL_60949, GeneID: 25743, Rattus norvegicus chr. 11<br>potassium inwardly-rectifying channel, subfamily J, member 6			
**GXP_73282** [GXP_73282] (1 - 1260)<br>**Apg3l**, GXL_60967, GeneID: 171415, Rattus norvegicus chr. 11<br>APG3 autophagy 3-like (S. cerevisiae)	neur-nr2f	233 - 109	(-)
**GXP_1408669** [GXP_1408669] (1 - 601)<br>**Ldhd**, GXL_64768, GeneID: 307858, Rattus norvegicus chr. 19<br>lactate dehydrogenase D	neur-nr2f	105 - 219	(+)
**GXP_77867** [GXP_77867] (1 - 666)<br>**Mvd**, GXL_64813, GeneID: 81726, Rattus norvegicus chr. 19<br>mevalonate (diphospho) decarboxylase	neur-nr2f	278 - 128	(-)
**GXP_1408249** [GXP_1408249] (1 - 601)<br>**Gpr56**, GXL_64827, GeneID: 260326, Rattus norvegicus chr. 19<br>G protein-coupled receptor 56	neur-nr2f	107 - 229	(+)
**GXP_1156620** [GXP_1156620] (1 - 601)<br>**Abcb10**, GXL_65061, GeneID: 361439, Rattus norvegicus chr. 19<br>ATP-binding cassette, sub-family B (MDR/TAP), member 10	neur-nr2f	420 - 541	(+)
**GXP_1420866** [GXP_1420866] (1 - 632)<br>**Rbm10**, GXL_69286, GeneID: 64510, Rattus norvegicus chr. X<br>RNA binding motif protein 10	neur-nr2f	196 - 347	(+)
**GXP_1421012** [GXP_1421012] (1 - 943)<br>**Mid1**, GXL_69368, GeneID: 54252, Rattus norvegicus chr. X<br>midline 1	neur-nr2f	565 - 703	(+)
**GXP_1420933** [GXP_1420933] (1 - 601)<br>**Syp**, GXL_69377, GeneID: 24804, Rattus norvegicus chr. X<br>synaptophysin	neur-nr2f	317 - 164	(-)
**GXP_83490** [GXP_83490] (1 - 648)<br>**Phb**, GXL_69423, GeneID: 25344, Rattus norvegicus chr. X<br>prohibitin	neur-nr2f	324 - 448	(+)
**GXP_83532** [GXP_83532] (1 - 818)<br>**Taf9l**, GXL_69464, GeneID: 171152, Rattus norvegicus chr. X<br>TAF9-like RNA polymerase II, TATA box binding protein (TBP)-associated factor, 31kDa	neur-nr2f	587 - 469	(-)
**GXP_83685** [GXP_83685] (1 - 941)<br>**Gyk**, GXL_69616, GeneID: 79223, Rattus norvegicus chr. X<br>glycerol kinase	neur-nr2f	428 - 279	(-)
**GXP_1404122** [GXP_1404122] (1 - 602)<br>**Dgcr2**, GXL_86337, GeneID: 360742, Rattus norvegicus chr. 11<br>DiGeorge syndrome critical region gene 2	neur-nr2f	424 - 550	(+)
	neur-nr2f	56 - 204	(+)

**GXP_107850** [GXP_107850] (1 - 601)<br>**Ppp1r3b**, GXL_90105, GeneID: 192280, Rattus norvegicus chr. 16<br>protein phosphatase 1, regulatory (inhibitor) subunit 3B			
**GXP_1406866** [GXP_1406866] (1 - 601)<br>**Bpy2ip1_predicted**, GXL_90266, GeneID: 290640, Rattus norvegicus chr. 16<br>BPY2 interacting protein 1 (predicted)	neur-nr2f	513 - 386	(-)
**GXP_1407186** [GXP_1407186] (1 - 602)<br>**Myr8**, GXL_90330, GeneID: 192253, Rattus norvegicus chr. 16<br>myosin heavy chain Myr 8	neur-nr2f	197 - 321	(+)
**GXP_108116** [GXP_108116] (1 - 659)<br>**F10**, GXL_90366, GeneID: 29243, Rattus norvegicus chr. 16<br>coagulation factor X	neur-nr2f	124 - 244	(+)
**GXP_855793** [GXP_855793] (1 - 695)<br>**F10**, GXL_90366, GeneID: 29243, Rattus norvegicus chr. 16<br>coagulation factor X	neur-nr2f	547 - 667	(+)
**GXP_855343** [GXP_855343] (1 - 601)<br>**Wnt5a**, GXL_90368, GeneID: 64566, Rattus norvegicus chr. 16<br>wingless-type MMTV integration site 5A	neur-nr2f	108 - 222	(+)
**GXP_1406873** [GXP_1406873] (1 - 602)<br>**Rab3a**, GXL_90422, GeneID: 25531, Rattus norvegicus chr. 16<br>RAB3A, member RAS oncogene family	neur-nr2f	507 - 362	(-)
**GXP_1407422** [GXP_1407422] (1 - 608)<br>**Ctag3**, GXL_96153, GeneID: 306872, Rattus norvegicus chr. 17<br>cancer/testis antigen 3	neur-nr2f	98 - 212	(+)
**GXP_1154638** [GXP_1154638] (1 - 603)<br>**Scgn**, GXL_96238, GeneID: 306942, Rattus norvegicus chr. 17<br>secretagogin, EF-hand calcium binding protein	neur-nr2f	378 - 492	(+)
**GXP_114959** [GXP_114959] (1 - 632)<br>**Testin**, GXL_96298, GeneID: 286916, Rattus norvegicus chr. 17<br>testin gene	neur-nr2f	7 - 160	(+)
**GXP_1154315** [GXP_1154315] (1 - 601)<br>**H2afy**, GXL_96319, GeneID: 29384, Rattus norvegicus chr. 17<br>H2A histone family, member Y	neur-nr2f	520 - 403	(-)
**GXP_1407672** [GXP_1407672] (1 - 601)<br>**Nmt2**, GXL_96381, GeneID: 291318, Rattus norvegicus chr. 17<br>N-myristoyltransferase 2	neur-nr2f	531 - 388	(-)
**GXP_1407254** [GXP_1407254] (1 - 1053)<br>**Ntrk2**, GXL_96436, GeneID: 25054, Rattus norvegicus chr. 17<br>neurotrophic tyrosine kinase, receptor, type 2	neur-nr2f	247 - 375	(+)
	neur-nr2f	134 - 268	(+)

**GXP_856110** [GXP_856110] (1 - 601)<br>**Inhba**, GXL_96480, GeneID: 29200, Rattus norvegicus chr. 17<br>inhibin beta-A			
**GXP_115183** [GXP_115183] (1 - 602)<br>**Fbp1**, GXL_96520, GeneID: 24362, Rattus norvegicus chr. 17<br>fructose-1,6- biphosphatase 1	neur-nr2f	371 - 506	(+)
**GXP_115906** [GXP_115906] (1 - 746)<br>**Stat3**, GXL_97163, GeneID: 25125, Rattus norvegicus chr. 10<br>signal transducer and activator of transcription 3	neur-nr2f	464 - 341	(-)
**GXP_1403244** [GXP_1403244] (1 - 602)<br>**Stat3**, GXL_97163, GeneID: 25125, Rattus norvegicus chr. 10<br>signal transducer and activator of transcription 3	neur-nr2f	383 - 233	(-)
**GXP_115908** [GXP_115908] (1 - 970)<br>**Kcnmb1**, GXL_97165, GeneID: 29747, Rattus norvegicus chr. 10<br>potassium large conductance calcium-activated channel, subfamily M, beta member 1	neur-nr2f	779 - 642	(-)
**GXP_115915** [GXP_115915] (1 - 896)<br>**Timp2**, GXL_97172, GeneID: 29543, Rattus norvegicus chr. 10<br>tissue inhibitor of metalloproteinase 2	neur-nr2f	405 - 264	(-)
**GXP_115973** [GXP_115973] (1 - 617)<br>**Nme3**, GXL_97230, GeneID: 85269, Rattus norvegicus chr. 10<br>non-metastatic cell expressed protein 3	neur-nr2f	204 - 76	(-)
**GXP_115994** [GXP_115994] (1 - 631)<br>**Cd7_predicted**, GXL_97251, GeneID: 303747, Rattus norvegicus chr. 10<br>CD7 antigen (predicted)	neur-nr2f	326 - 203	(-)
**GXP_116049** [GXP_116049] (1 - 601)<br>**Olr1391_predicted**, GXL_97305, GeneID: 405983, Rattus norvegicus chr. 10<br>olfactory receptor 1391 (predicted)	neur-nr2f	78 - 213	(+)
**GXP_116064** [GXP_116064] (1 - 709)<br>**Prm2**, GXL_97320, GeneID: 25345, Rattus norvegicus chr. 10<br>protamine 2	neur-nr2f	536 - 394	(-)
**GXP_116181** [GXP_116181] (1 - 601)<br>**Olr1389_predicted**, GXL_97437, GeneID: 287240, Rattus norvegicus chr. 10<br>olfactory receptor 1389 (predicted)	neur-nr2f	109 - 251	(+)
**GXP_1403149** [GXP_1403149] (1 - 1302)<br>**Snip**, GXL_97503, GeneID: 56029, Rattus norvegicus chr. 10<br>SNAP25-interacting protein	neur-nr2f	279 - 164	(-)
**GXP_1154930** [GXP_1154930] (1 - 611)<br>**Sec61a2_predicted**, GXL_113296, GeneID: 361273, Rattus norvegicus chr. 17<br>Sec61, alpha subunit 2 (S. cerevisiae) (predicted)	neur-nr2f	223 - 352	(+)
	neur-nr2f	263 - 381	(+)

**GXP_1407649** [GXP_1407649] (1 - 774)<br>**Optn**, GXL_113321, GeneID: 246294, Rattus norvegicus chr. 17<br>optineurin			
**GXP_498959** [GXP_498959] (1 - 601)<br>**Gabrg2**, GXL_123469, GeneID: 29709, Rattus norvegicus chr. 10<br>gamma-aminobutyric acid A receptor, gamma 2	neur-nr2f	336 - 479	(+)
**GXP_1402422** [GXP_1402422] (1 - 601)<br>**Maml1_predicted**, GXL_123472, GeneID: 303101, Rattus norvegicus chr. 10<br>mastermind like 1 (Drosophila) (predicted)	neur-nr2f	431 - 289	(-)
**GXP_1402638** [GXP_1402638] (1 - 602)<br>**Ntn1**, GXL_123533, GeneID: 114523, Rattus norvegicus chr. 10<br>netrin 1	neur-nr2f	154 - 31	(-)
**GXP_148227** [GXP_148227] (1 - 937)<br>**Rasd1**, GXL_123816, GeneID: 64455, Rattus norvegicus chr. 10<br>RAS, dexamethasone-induced 1	neur-nr2f	384 - 269	(-)
**GXP_1402681** [GXP_1402681] (1 - 1229)<br>**Atp1b2**, GXL_123823, GeneID: 24214, Rattus norvegicus chr. 10<br>ATPase, Na+/K+ transporting, beta 2 polypeptide	neur-nr2f	675 - 799	(+)
**GXP_148248** [GXP_148248] (1 - 625)<br>**Tekt1**, GXL_123837, GeneID: 85270, Rattus norvegicus chr. 10<br>tektin 1	neur-nr2f	246 - 394	(+)
**GXP_1402798** [GXP_1402798] (1 - 604)<br>**Srr**, GXL_123849, GeneID: 303306, Rattus norvegicus chr. 10<br>serine racemase	neur-nr2f	554 - 407	(-)
**GXP_1149704** [GXP_1149704] (1 - 721)<br>**Lnx2_predicted**, GXL_126533, GeneID: 360761, Rattus norvegicus chr. 12<br>ligand of numb-protein X 2 (predicted)	neur-nr2f	345 - 195	(-)
**GXP_1404249** [GXP_1404249] (1 - 611)<br>**Zfp655**, GXL_126553, GeneID: 360764, Rattus norvegicus chr. 12<br>zinc finger protein 655	neur-nr2f	391 - 516	(+)
**GXP_1404659** [GXP_1404659] (1 - 601)<br>**Pxn**, GXL_126584, GeneID: 360820, Rattus norvegicus chr. 12<br>paxillin	neur-nr2f	65 - 195	(+)
**GXP_1404421** [GXP_1404421] (1 - 602)<br>**Stx1a**, GXL_126642, GeneID: 116470, Rattus norvegicus chr. 12<br>syntaxin 1A (brain)	neur-nr2f	325 - 459	(+)
**GXP_1404431** [GXP_1404431] (1 - 609)<br>**Gtf2ird1**, GXL_126645, GeneID: 246770, Rattus norvegicus chr. 12<br>general transcription factor II I repeat domain-containing 1	neur-nr2f	387 - 521	(+)
	neur-nr2f	191 - 48	(-)

**GXP_1404341** [GXP_1404341] (1 - 649)<br>**Zfand2a**, GXL_126777, GeneID: 360772, Rattus norvegicus chr. 12<br>zinc finger, AN1-type domain 2A			
**GXP_151955** [GXP_151955] (1 - 1260)<br>**Arhgef18_predicted**, GXL_126814, GeneID: 304193, Rattus norvegicus chr. 12<br>rho/rac guanine nucleotide exchange factor (GEF) 18 (predicted)	neur-nr2f	535 - 654	(+)
**GXP_1404383** [GXP_1404383] (1 - 602)<br>**Serpine1**, GXL_126862, GeneID: 24617, Rattus norvegicus chr. 12<br>serine (or cysteine) peptidase inhibitor, clade E, member 1	neur-nr2f	513 - 383	(-)
**GXP_1403286** [GXP_1403286] (1 - 602)<br>**G6pc3**, GXL_135137, GeneID: 303565, Rattus norvegicus chr. 10<br>glucose 6 phosphatase, catalytic, 3	neur-nr2f	65 - 193	(+)
**GXP_1148361** [GXP_1148361] (1 - 601)<br>**Ramp2**, GXL_135154, GeneID: 58966, Rattus norvegicus chr. 10<br>receptor (calcitonin) activity modifying protein 2	neur-nr2f	288 - 404	(+)
**GXP_1403085** [GXP_1403085] (1 - 602)<br>**Lrrc59**, GXL_135159, GeneID: 287633, Rattus norvegicus chr. 10<br>leucine rich repeat containing 59	neur-nr2f	421 - 276	(-)
**GXP_162553** [GXP_162553] (1 - 695)<br>**Ccl11**, GXL_135197, GeneID: 29397, Rattus norvegicus chr. 10<br>chemokine (C-C motif) ligand 11	neur-nr2f	415 - 281	(-)
**GXP_162564** [GXP_162564] (1 - 601)<br>**Olr1408_predicted**, GXL_135208, GeneID: 405063, Rattus norvegicus chr. 10<br>olfactory receptor 1408 (predicted)	neur-nr2f	250 - 134	(-)
**GXP_1402852** [GXP_1402852] (1 - 602)<br>**Flot2**, GXL_135246, GeneID: 83764, Rattus norvegicus chr. 10<br>flotillin 2	neur-nr2f	374 - 260	(-)
**GXP_162606** [GXP_162606] (1 - 601)<br>**Olr1439_predicted**, GXL_135250, GeneID: 287328, Rattus norvegicus chr. 10<br>olfactory receptor 1439 (predicted)	neur-nr2f	573 - 438	(-)
**GXP_162629** [GXP_162629] (1 - 768)<br>**Col23a1**, GXL_135273, GeneID: 353303, Rattus norvegicus chr. 10<br>procollagen, type XXIII, alpha 1	neur-nr2f	12 - 131	(+)
**GXP_1402710** [GXP_1402710] (1 - 602)<br>**Dlgh4**, GXL_135280, GeneID: 29495, Rattus norvegicus chr. 10<br>discs, large homolog 4 (Drosophila)	neur-nr2f	88 - 234	(+)
**GXP_162705** [GXP_162705] (1 - 816)<br>**Ccdc42_predicted**, GXL_135349, GeneID: 303234, Rattus norvegicus chr. 10<br>coiled-coil domain containing 42 (predicted)	neur-nr2f	329 - 198	(-)
	neur-nr2f	161 - 32	(-)

**GXP_162809** [GXP_162809] (1 - 601)<br>**Olr1515_predicted**, GXL_135453, GeneID: 405994, Rattus norvegicus chr. 10<br>olfactory receptor 1515 (predicted)			
**GXP_162824** [GXP_162824] (1 - 601)<br>**Aoc3**, GXL_135468, GeneID: 29473, Rattus norvegicus chr. 10<br>amine oxidase, copper containing 3	neur-nr2f	202 - 67	(-)
**GXP_168380** [GXP_168380] (1 - 1433)<br>**Lin7a**, GXL_140016, GeneID: 85327, Rattus norvegicus chr. 7<br>lin-7 homolog a (C. elegans)	neur-nr2f	985 - 862	(-)
**GXP_1418845** [GXP_1418845] (1 - 601)<br>**Nfe2**, GXL_140075, GeneID: 366998, Rattus norvegicus chr. 7<br>nuclear factor, erythroid derived 2	neur-nr2f	359 - 210	(-)
**GXP_168462** [GXP_168462] (1 - 607)<br>**Olr908_predicted**, GXL_140095, GeneID: 288806, Rattus norvegicus chr. 7<br>olfactory receptor 908 (predicted)	neur-nr2f	553 - 404	(-)
**GXP_168463** [GXP_168463] (1 - 763)<br>**Wnt7b**, GXL_140096, GeneID: 315196, Rattus norvegicus chr. 7<br>wingless-related MMTV integration site 7B	neur-nr2f	272 - 150	(-)
**GXP_168520** [GXP_168520] (1 - 601)<br>**Olr954_predicted**, GXL_140150, GeneID: 404938, Rattus norvegicus chr. 7<br>olfactory receptor 954 (predicted)	neur-nr2f	553 - 404	(-)
**GXP_168538** [GXP_168538] (1 - 742)<br>**Gns**, GXL_140168, GeneID: 299825, Rattus norvegicus chr. 7<br>glucosamine (N-acetyl)-6-sulfatase	neur-nr2f	363 - 240	(-)
**GXP_168588** [GXP_168588] (1 - 632)<br>**Ela1**, GXL_140217, GeneID: 24331, Rattus norvegicus chr. 7<br>elastase 1, pancreatic	neur-nr2f	589 - 437	(-)
**GXP_1418161** [GXP_1418161] (1 - 601)<br>**Grip1**, GXL_140323, GeneID: 84016, Rattus norvegicus chr. 7<br>glutamate receptor interacting protein 1	neur-nr2f	279 - 429	(+)
**GXP_1169939** [GXP_1169939] (1 - 601)<br>**Prg-2**, GXL_140324, GeneID: 314614, Rattus norvegicus chr. 7<br>plasticity-related protein PRG-2	neur-nr2f	314 - 432	(+)
**GXP_1170976** [GXP_1170976] (1 - 601)<br>**Naga**, GXL_140329, GeneID: 315165, Rattus norvegicus chr. 7<br>N-acetyl galactosaminidase, alpha	neur-nr2f	165 - 34	(-)
**GXP_168714** [GXP_168714] (1 - 625)<br>**Cyp2d22**, GXL_140343, GeneID: 171522, Rattus norvegicus chr. 7<br>cytochrome P450, family 2, subfamily d, polypeptide 22	neur-nr2f	110 - 248	(+)
	neur-nr2f	381 - 249	(-)

**GXP_864217** [GXP_864217] (1 - 601)			
**Faim2**, GXL_140350, GeneID: 246274, Rattus norvegicus chr. 7			
Fas apoptotic inhibitory molecule 2			
**GXP_1421410** [GXP_1421410] (1 - 602)			
**Avpr2**, GXL_142347, GeneID: 25108, Rattus norvegicus chr. X	neur-nr2f	450 - 579	(+)
arginine vasopressin receptor 2			
**GXP_171217** [GXP_171217] (1 - 686)			
**Acsl4**, GXL_142383, GeneID: 113976, Rattus norvegicus chr. X	neur-nr2f	58 - 172	(+)
acyl-CoA synthetase long-chain family member 4			
**GXP_171347** [GXP_171347] (1 - 717)			
**Msn**, GXL_142511, GeneID: 81521, Rattus norvegicus chr. X	neur-nr2f	291 - 158	(-)
moesin			
**GXP_171438** [GXP_171438] (1 - 667)			
**Fhl1**, GXL_142602, GeneID: 25177, Rattus norvegicus chr. X	neur-nr2f	480 - 614	(+)
four and a half LIM domains 1			
**GXP_180104** [GXP_180104] (1 - 601)			
**Olr392_predicted**, GXL_150703, GeneID: 405227, Rattus norvegicus chr. 2	neur-nr2f	253 - 391	(+)
olfactory receptor 392 (predicted)			
**GXP_180116** [GXP_180116] (1 - 601)			
**Abcd3**, GXL_150715, GeneID: 25270, Rattus norvegicus chr. 2	neur-nr2f	112 - 237	(+)
ATP-binding cassette, sub-family D (ALD), member 3			
**GXP_180158** [GXP_180158] (1 - 685)			
**Tpo1**, GXL_150756, GeneID: 170907, Rattus norvegicus chr. 2	neur-nr2f	168 - 21	(-)
developmentally regulated protein TPO1			
**GXP_180201** [GXP_180201] (1 - 755)			
**Gstm1**, GXL_150799, GeneID: 24423, Rattus norvegicus chr. 2	neur-nr2f	63 - 194	(+)
glutathione S-transferase, mu 1			
**GXP_180409** [GXP_180409] (1 - 648)			
**Lhx8_predicted**, GXL_151005, GeneID: 365963, Rattus norvegicus chr. 2	neur-nr2f	451 - 585	(+)
LIM homeobox protein 8 (predicted)			
**GXP_182612** [GXP_182612] (1 - 1071)			
**Kcnk4**, GXL_152771, GeneID: 116489, Rattus norvegicus chr. 1	neur-nr2f	226 - 366	(+)
potassium channel, subfamily K, member 4			
**GXP_182701** [GXP_182701] (1 - 862)			
**Pola2**, GXL_152857, GeneID: 85242, Rattus norvegicus chr. 1	neur-nr2f	788 - 638	(-)
polymerase (DNA directed), alpha 2			
**GXP_1410960** [GXP_1410960] (1 - 601)			
**Ubtd1**, GXL_152913, GeneID: 309373, Rattus norvegicus chr. 1	neur-nr2f	413 - 271	(-)
ubiquitin domain containing 1			
	neur-nr2f	315 - 166	(-)

**GXP_182907** [GXP_182907] (1 - 692)<br>**Rps6kb2**, GXL_153056, GeneID: 361696, Rattus norvegicus chr. 1<br>ribosomal protein S6 kinase, polypeptide 2			
**GXP_199062** [GXP_199062] (1 - 601)			
**Fut4**, GXL_166082, GeneID: 60670, Rattus norvegicus chr. 8<br>fucosyltransferase 4	neur-nr2f	345 - 226	(-)
**GXP_1419165** [GXP_1419165] (1 - 602)			
**Tagln**, GXL_166283, GeneID: 25123, Rattus norvegicus chr. 8<br>transgelin	neur-nr2f	126 - 6	(-)
**GXP_199366** [GXP_199366] (1 - 1150)			
**Rbm7_predicted**, GXL_166382, GeneID: 315634, Rattus norvegicus chr. 8<br>RNA binding motif protein 7 (predicted)	neur-nr2f	139 - 266	(+)
**GXP_199384** [GXP_199384] (1 - 778)			
**Htr3b**, GXL_166400, GeneID: 58963, Rattus norvegicus chr. 8<br>5-hydroxytryptamine (serotonin) receptor 3b	neur-nr2f	443 - 295	(-)
**GXP_199485** [GXP_199485] (1 - 730)			
**Mapkapk3**, GXL_166500, GeneID: 315994, Rattus norvegicus chr. 8<br>mitogen-activated protein kinase-activated protein kinase 3	neur-nr2f	155 - 40	(-)
**GXP_1417322** [GXP_1417322] (1 - 607)			
**Pygl**, GXL_168677, GeneID: 64035, Rattus norvegicus chr. 6<br>liver glycogen phosphorylase	neur-nr2f	449 - 600	(+)
**GXP_1417437** [GXP_1417437] (1 - 638)			
**Slc8a3**, GXL_168702, GeneID: 140448, Rattus norvegicus chr. 6<br>solute carrier family 8 (sodium/calcium exchanger), member 3	neur-nr2f	392 - 538	(+)
**GXP_202183** [GXP_202183] (1 - 758)			
**Abhd1**, GXL_168777, GeneID: 313917, Rattus norvegicus chr. 6<br>abhydrolase domain containing 1	neur-nr2f	735 - 615	(-)
**GXP_1417595** [GXP_1417595] (1 - 1140)			
**Asb2**, GXL_168796, GeneID: 299266, Rattus norvegicus chr. 6<br>ankyrin repeat and SOCS box-containing protein 2	neur-nr2f	948 - 1079	(+)
**GXP_210792** [GXP_210792] (1 - 754)			
**Atp4b**, GXL_175723, GeneID: 24217, Rattus norvegicus chr. 16<br>ATPase, H+/K+ exchanging, beta polypeptide	neur-nr2f	221 - 374	(+)
**GXP_210890** [GXP_210890] (1 - 765)			
**Fnta**, GXL_175819, GeneID: 25318, Rattus norvegicus chr. 16<br>farnesyltransferase, CAAX box, alpha	neur-nr2f	201 - 86	(-)
**GXP_1409792** [GXP_1409792] (1 - 639)			
**Fsd2_predicted**, GXL_182573, GeneID: 308779, Rattus norvegicus chr. 1<br>fibronectin type III and SPRY domain containing 2 (predicted)	neur-nr2f	232 - 117	(-)
	neur-nr2f	193 - 46	(-)

**GXP_1409571** [GXP_1409571] (1 - 611)			
**Emp3**, GXL_182635, GeneID: 81505, Rattus norvegicus chr. 1			
epithelial membrane protein 3			
**GXP_1409789** [GXP_1409789] (1 - 705)			
**Ap3b2_predicted**, GXL_182715, GeneID: 308777, Rattus norvegicus chr. 1	neur-nr2f	240 - 117	(-)
adaptor-related protein complex 3, beta 2 subunit (predicted)			
**GXP_218744** [GXP_218744] (1 - 602)			
**Grwd1**, GXL_182799, GeneID: 308592, Rattus norvegicus chr. 1	neur-nr2f	348 - 481	(+)
glutamate-rich WD repeat containing 1			
**GXP_218811** [GXP_218811] (1 - 836)			
**Anpep**, GXL_182864, GeneID: 81641, Rattus norvegicus chr. 1	neur-nr2f	201 - 342	(+)
alanyl (membrane) aminopeptidase			
**GXP_218845** [GXP_218845] (1 - 601)			
**Csrp3**, GXL_182898, GeneID: 117505, Rattus norvegicus chr. 1	neur-nr2f	281 - 165	(-)
cysteine and glycine-rich protein 3			
**GXP_218869** [GXP_218869] (1 - 874)			
**Dbx1**, GXL_182921, GeneID: 292934, Rattus norvegicus chr. 1	neur-nr2f	62 - 195	(+)
developing brain homeobox 1			
**GXP_1409615** [GXP_1409615] (1 - 601)			
**Dbx1**, GXL_182921, GeneID: 292934, Rattus norvegicus chr. 1	neur-nr2f	105 - 238	(+)
developing brain homeobox 1			
**GXP_218877** [GXP_218877] (1 - 621)			
**Inppl1**, GXL_182929, GeneID: 65038, Rattus norvegicus chr. 1	neur-nr2f	515 - 370	(-)
inositol polyphosphate phosphatase-like 1			
**GXP_1410215** [GXP_1410215] (1 - 602)			
**Cln3**, GXL_182936, GeneID: 293485, Rattus norvegicus chr. 1	neur-nr2f	350 - 482	(+)
ceroid lipofuscinosis, neuronal 3, juvenile (Batten, Spielmeyer-Vogt disease)			
**GXP_218894** [GXP_218894] (1 - 616)			
**Cuzd1**, GXL_182946, GeneID: 117179, Rattus norvegicus chr. 1	neur-nr2f	392 - 257	(-)
CUB and zona pellucida-like domains 1			
**GXP_218944** [GXP_218944] (1 - 710)			
**Prss8**, GXL_182996, GeneID: 192107, Rattus norvegicus chr. 1	neur-nr2f	273 - 138	(-)
protease, serine, 8 (prostasin)			
**GXP_224056** [GXP_224056] (1 - 601)			
**H2-T18**, GXL_186879, GeneID: 406194, Rattus norvegicus chr. 20	neur-nr2f	384 - 255	(-)
histocompatibility 2, T region locus 18			
**GXP_224152** [GXP_224152] (1 - 602)			
**Dpcr1**, GXL_186971, GeneID: 406199, Rattus norvegicus chr. 20	neur-nr2f	22 - 155	(+)
diffuse panbronchiolitis critical region gene 1			
	neur-nr2f	467 - 314	(-)

**GXP_1411172** [GXP_1411172] (1 - 601)<br>**Bat5**, GXL_187001, GeneID: 361796, Rattus norvegicus chr. 20<br>HLA-B associated transcript 5			
**GXP_1160295** [GXP_1160295] (1 - 601)<br>**Notch4**, GXL_187032, GeneID: 406162, Rattus norvegicus chr. 20<br>Notch homolog 4	neur-nr2f	58 - 184	(+)
**GXP_224244** [GXP_224244] (1 - 601)<br>**RT1-O**, GXL_187058, GeneID: 24751, Rattus norvegicus chr. 20<br>RT1 class Ib, locus H2-Q-like, grc region	neur-nr2f	388 - 272	(-)
**GXP_224249** [GXP_224249] (1 - 1009)<br>**Gpsm3**, GXL_187063, GeneID: 406163, Rattus norvegicus chr. 20<br>G-protein signalling modulator 3 (AGS3-like, C. elegans)	neur-nr2f	58 - 184	(+)
**GXP_224335** [GXP_224335] (1 - 685)<br>**Ltb**, GXL_187145, GeneID: 361795, Rattus norvegicus chr. 20<br>lymphotoxin B	neur-nr2f	174 - 288	(+)
**GXP_864492** [GXP_864492] (1 - 601)<br>**Cdon**, GXL_188732, GeneID: 50938, Rattus norvegicus chr. 8<br>cell adhesion molecule-related/down-regulated by oncogenes	neur-nr2f	405 - 264	(-)
**GXP_226220** [GXP_226220] (1 - 601)<br>**Xtrp3**, GXL_188747, GeneID: 113918, Rattus norvegicus chr. 8<br>X transporter protein 3	neur-nr2f	320 - 451	(+)
**GXP_226284** [GXP_226284] (1 - 601)<br>**Olr1320_predicted**, GXL_188811, GeneID: 300621, Rattus norvegicus chr. 8<br>olfactory receptor 1320 (predicted)	neur-nr2f	308 - 181	(-)
**GXP_1419268** [GXP_1419268] (1 - 603)<br>**Ireb2**, GXL_188845, GeneID: 64831, Rattus norvegicus chr. 8<br>iron responsive element binding protein 2	neur-nr2f	286 - 403	(+)
**GXP_1419105** [GXP_1419105] (1 - 881)<br>**H2afx**, GXL_188941, GeneID: 300668, Rattus norvegicus chr. 8<br>dolichyl-phosphate (UDP-N-acetylglucosamine) N-acetylglucosaminephosphotransferase 1 (GlcNAc-1-P transferase)	neur-nr2f	144 - 24	(-)
**GXP_229064** [GXP_229064] (1 - 601)<br>**Tgfb2**, GXL_190945, GeneID: 81809, Rattus norvegicus chr. 13<br>transforming growth factor, beta 2	neur-nr2f	174 - 317	(+)
**GXP_1404764** [GXP_1404764] (1 - 627)<br>**Serpinb2**, GXL_190950, GeneID: 60325, Rattus norvegicus chr. 13<br>serine (or cysteine) proteinase inhibitor, clade B, member 2	neur-nr2f	200 - 48	(-)
**GXP_229139** [GXP_229139] (1 - 996)<br>**Dpt_predicted**, GXL_191017, GeneID: 289178, Rattus norvegicus chr. 13<br>dermatopontin (predicted)	neur-nr2f	499 - 375	(-)
	neur-nr2f	449 - 315	(-)

**GXP_229149** [GXP_229149] (1 - 749)<br>**Fvt1_predicted**, GXL_191027, GeneID: 360833, Rattus norvegicus chr. 13<br>follicular lymphoma variant translocation 1 (predicted)			
**GXP_1404933** [GXP_1404933] (1 - 622)<br>**Csrp1**, GXL_191030, GeneID: 29276, Rattus norvegicus chr. 13<br>cysteine and glycine-rich protein 1	neur-nr2f	429 - 575	(+)
**GXP_1405337** [GXP_1405337] (1 - 615)<br>**Lbr**, GXL_191066, GeneID: 89789, Rattus norvegicus chr. 13<br>lamin B receptor	neur-nr2f	430 - 576	(+)
**GXP_1404916** [GXP_1404916] (1 - 601)<br>**Ptprv**, GXL_191067, GeneID: 64576, Rattus norvegicus chr. 13<br>protein tyrosine phosphatase, receptor type, V	neur-nr2f	179 - 28	(-)
**GXP_1404899** [GXP_1404899] (1 - 602)<br>**Klhl12**, GXL_191190, GeneID: 266772, Rattus norvegicus chr. 13<br>kelch-like 12 (Drosophila)	neur-nr2f	82 - 220	(+)
**GXP_1404957** [GXP_1404957] (1 - 602)<br>**Lhx9**, GXL_191240, GeneID: 289048, Rattus norvegicus chr. 13<br>LIM homeobox 9	neur-nr2f	450 - 307	(-)
**GXP_1404917** [GXP_1404917] (1 - 1137)<br>**Ptpn7**, GXL_191273, GeneID: 246781, Rattus norvegicus chr. 13<br>protein tyrosine phosphatase, non-receptor type 7	neur-nr2f	98 - 249	(+)
**GXP_229415** [GXP_229415] (1 - 975)<br>**Hsd11b1**, GXL_191291, GeneID: 25116, Rattus norvegicus chr. 13<br>hydroxysteroid 11-beta dehydrogenase 1	neur-nr2f	5 - 148	(+)
**GXP_237776** [GXP_237776] (1 - 766)<br>**Fkbp1a**, GXL_198133, GeneID: 25639, Rattus norvegicus chr. 3<br>FK506 binding protein 1a	neur-nr2f	495 - 618	(+)
**GXP_237793** [GXP_237793] (1 - 601)<br>**Olr754_predicted**, GXL_198150, GeneID: 296005, Rattus norvegicus chr. 3<br>olfactory receptor 754 (predicted)	neur-nr2f	428 - 563	(+)
**GXP_1413175** [GXP_1413175] (1 - 602)<br>**Nek6**, GXL_198194, GeneID: 360161, Rattus norvegicus chr. 3<br>NIMA (never in mitosis gene a)-related expressed kinase 6	neur-nr2f	507 - 383	(-)
**GXP_237896** [GXP_237896] (1 - 645)<br>**Lin7c**, GXL_198252, GeneID: 60442, Rattus norvegicus chr. 3<br>lin-7 homolog C (C. elegans)	neur-nr2f	361 - 227	(-)
**GXP_1414060** [GXP_1414060] (1 - 601)<br>**Entpd6**, GXL_198266, GeneID: 85260, Rattus norvegicus chr. 3<br>ectonucleoside triphosphate diphosphohydrolase 6	neur-nr2f	553 - 425	(-)
	neur-nr2f	170 - 29	(-)

**GXP_237933** [GXP_237933] (1 - 1043)<br>**Chgb**, GXL_198289, GeneID: 24259, Rattus norvegicus chr. 3<br>chromogranin B			
**GXP_237993** [GXP_237993] (1 - 601)<br>**Olr562_predicted**, GXL_198349, GeneID: 404869, Rattus norvegicus chr. 3<br>olfactory receptor 562 (predicted)	neur-nr2f	342 - 212	(-)
**GXP_1413593** [GXP_1413593] (1 - 602)<br>**Abtb2**, GXL_198420, GeneID: 171440, Rattus norvegicus chr. 3<br>ankyrin repeat and BTB (POZ) domain containing 2	neur-nr2f	211 - 74	(-)
**GXP_1413493** [GXP_1413493] (1 - 603)<br>**Acp2**, GXL_198444, GeneID: 24162, Rattus norvegicus chr. 3<br>acid phosphatase 2, lysosomal	neur-nr2f	494 - 377	(-)
**GXP_238124** [GXP_238124] (1 - 612)<br>**Ptpra**, GXL_198479, GeneID: 25167, Rattus norvegicus chr. 3<br>protein tyrosine phosphatase, receptor type, A	neur-nr2f	232 - 360	(+)
**GXP_1413331** [GXP_1413331] (1 - 602)<br>**Cybrd1**, GXL_198578, GeneID: 295669, Rattus norvegicus chr. 3<br>cytochrome b reductase 1	neur-nr2f	108 - 242	(+)
**GXP_1406154** [GXP_1406154] (1 - 603)<br>**Fut11**, GXL_198718, GeneID: 286971, Rattus norvegicus chr. 15<br>fucosyltransferase 11	neur-nr2f	346 - 221	(-)
**GXP_238408** [GXP_238408] (1 - 730)<br>**Ptk2b**, GXL_198760, GeneID: 50646, Rattus norvegicus chr. 15<br>protein tyrosine kinase 2 beta	neur-nr2f	372 - 220	(-)
**GXP_1406258** [GXP_1406258] (1 - 602)<br>**Tep1**, GXL_198788, GeneID: 64523, Rattus norvegicus chr. 15<br>telomerase associated protein 1	neur-nr2f	530 - 396	(-)
**GXP_1406320** [GXP_1406320] (1 - 601)<br>**Prmt5_predicted**, GXL_198840, GeneID: 364382, Rattus norvegicus chr. 15<br>protein arginine N-methyltransferase 5 (predicted)	neur-nr2f	383 - 268	(-)
**GXP_1406510** [GXP_1406510] (1 - 602)<br>**Pdlim2**, GXL_198850, GeneID: 290354, Rattus norvegicus chr. 15<br>PDZ and LIM domain 2	neur-nr2f	599 - 458	(-)
**GXP_1406571** [GXP_1406571] (1 - 601)<br>**Cog3**, GXL_198933, GeneID: 361073, Rattus norvegicus chr. 15<br>component of oligomeric golgi complex 3	neur-nr2f	515 - 369	(-)
**GXP_1406268** [GXP_1406268] (1 - 602)<br>**Ndrg2**, GXL_198981, GeneID: 171114, Rattus norvegicus chr. 15<br>N-myc downstream regulated gene 2	neur-nr2f	314 - 182	(-)
	neur-nr2f	301 - 177	(-)

**GXP_241360** [GXP_241360] (1 - 705)<br>**Slc22a7**, GXL_201121, GeneID: 89776, Rattus norvegicus chr. 9<br>solute carrier family 22 (organic anion transporter), member 7			
**GXP_241418** [GXP_241418] (1 - 1047)<br>**Igfbp5**, GXL_201176, GeneID: 25285, Rattus norvegicus chr. 9<br>insulin-like growth factor binding protein 5	neur-nr2f	814 - 683	(-)
**GXP_1173060** [GXP_1173060] (1 - 601)<br>**Vegfa**, GXL_201205, GeneID: 83785, Rattus norvegicus chr. 9<br>vascular endothelial growth factor A	neur-nr2f	278 - 144	(-)
**GXP_241485** [GXP_241485] (1 - 650)<br>**Twsg1_predicted**, GXL_201241, GeneID: 363294, Rattus norvegicus chr. 9<br>twisted gastrulation homolog 1 (Drosophila) (predicted)	neur-nr2f	399 - 248	(-)
**GXP_1420538** [GXP_1420538] (1 - 609)<br>**Tuba4a**, GXL_201284, GeneID: 316531, Rattus norvegicus chr. 9<br>tubulin, alpha 4A	neur-nr2f	387 - 530	(+)
**GXP_1420540** [GXP_1420540] (1 - 602)<br>**Tuba4a**, GXL_201284, GeneID: 316531, Rattus norvegicus chr. 9<br>tubulin, alpha 4A	neur-nr2f	363 - 490	(+)
**GXP_1420541** [GXP_1420541] (1 - 601)<br>**Tuba4a**, GXL_201284, GeneID: 316531, Rattus norvegicus chr. 9<br>tubulin, alpha 4A	neur-nr2f	404 - 531	(+)
**GXP_1420374** [GXP_1420374] (1 - 623)<br>**Orc2l**, GXL_201287, GeneID: 301430, Rattus norvegicus chr. 9<br>origin recognition complex, subunit 2-like (S. cerevisiae)	neur-nr2f	269 - 397	(+)
**GXP_1420553** [GXP_1420553] (1 - 1468)<br>**D1bwg1363e**, GXL_201311, GeneID: 316533, Rattus norvegicus chr. 9<br>chondroitin polymerizing factor	neur-nr2f	1175 - 1050	(-)
**GXP_1420679** [GXP_1420679] (1 - 601)<br>**Per2**, GXL_201351, GeneID: 63840, Rattus norvegicus chr. 9<br>period homolog 2 (Drosophila)	neur-nr2f	507 - 361	(-)
**GXP_1420101** [GXP_1420101] (1 - 603)<br>**Hspcb**, GXL_201364, GeneID: 301252, Rattus norvegicus chr. 9<br>heat shock 90kDa protein 1, beta	neur-nr2f	426 - 289	(-)
**GXP_1173099** [GXP_1173099] (1 - 837)<br>**Mep1a**, GXL_201410, GeneID: 25684, Rattus norvegicus chr. 9<br>meprin 1 alpha	neur-nr2f	462 - 314	(-)
**GXP_241755** [GXP_241755] (1 - 601)<br>**Lrrc30_predicted**, GXL_201499, GeneID: 301711, Rattus norvegicus chr. 9<br>leucine rich repeat containing 30 (predicted)	neur-nr2f	342 - 459	(+)
	neur-nr2f	102 - 240	(+)

**GXP_241783** [GXP_241783] (1 - 601)<br>**Gpr1**, GXL_201527, GeneID: 25457, Rattus norvegicus chr. 9<br>G protein-coupled receptor 1			
**GXP_241881** [GXP_241881] (1 - 601)<br>**Adm2**, GXL_201623, GeneID: 399475, Rattus norvegicus chr. 7<br>adrenomedullin 2	neur-nr2f	90 - 237	(+)
**GXP_1418400** [GXP_1418400] (1 - 601)<br>**Mapk15**, GXL_201655, GeneID: 286997, Rattus norvegicus chr. 7<br>mitogen-activated protein kinase 15	neur-nr2f	447 - 563	(+)
**GXP_241919** [GXP_241919] (1 - 601)<br>**Rdh2**, GXL_201660, GeneID: 299511, Rattus norvegicus chr. 7<br>retinol dehydrogenase 2	neur-nr2f	540 - 423	(-)
**GXP_520637** [GXP_520637] (1 - 601)<br>**Csf2rb1**, GXL_201687, GeneID: 171081, Rattus norvegicus chr. 7<br>colony stimulating factor 2 receptor, beta 1, low-affinity (granulocyte-macrophage)	neur-nr2f	457 - 326	(-)
**GXP_1418565** [GXP_1418565] (1 - 608)<br>**Ttll1**, GXL_201728, GeneID: 362969, Rattus norvegicus chr. 7<br>tubulin tyrosine ligase-like 1	neur-nr2f	186 - 61	(-)
**GXP_1418239** [GXP_1418239] (1 - 601)<br>**Tac2**, GXL_201742, GeneID: 29191, Rattus norvegicus chr. 7<br>tachykinin 2	neur-nr2f	415 - 567	(+)
**GXP_1418668** [GXP_1418668] (1 - 602)<br>**Cntn1**, GXL_201812, GeneID: 117258, Rattus norvegicus chr. 7<br>contactin 1	neur-nr2f	174 - 295	(+)
**GXP_1172635** [GXP_1172635] (1 - 601)<br>**Mst1**, GXL_203448, GeneID: 24566, Rattus norvegicus chr. 8<br>Macrophage stimulating 1 (hepatocyte growth factor-like)	neur-nr2f	171 - 55	(-)
**GXP_1172680** [GXP_1172680] (1 - 601)<br>**Smarcc1_predicted**, GXL_203497, GeneID: 301020, Rattus norvegicus chr. 8<br>SWI/SNF related, matrix associated, actin dependent regulator of chromatin, subfamily c, member 1 (predicted)	neur-nr2f	174 - 311	(+)
**GXP_253144** [GXP_253144] (1 - 657)<br>**C3ar1**, GXL_211893, GeneID: 84007, Rattus norvegicus chr. 4<br>complement component 3a receptor 1	neur-nr2f	257 - 127	(-)
**GXP_1414717** [GXP_1414717] (1 - 615)<br>**Slc35b4_predicted**, GXL_211948, GeneID: 296969, Rattus norvegicus chr. 4<br>solute carrier family 35, member B4 (predicted)	neur-nr2f	119 - 258	(+)
**GXP_1414673** [GXP_1414673] (1 - 601)<br>**Opn1sw**, GXL_211995, GeneID: 81644, Rattus norvegicus chr. 4<br>opsin 1 (cone pigments), short-wave-sensitive (color blindness, tritan)	neur-nr2f	213 - 349	(+)
	neur-nr2f	224 - 361	(+)

**GXP_253264** [GXP_253264] (1 - 715)<br>**Oldlr1**, GXL_212001, GeneID: 140914, Rattus norvegicus chr. 4<br>oxidized low density lipoprotein (lectin-like) receptor 1			
**GXP_253326** [GXP_253326] (1 - 643)<br>**Mrpl35_predicted**, GXL_212062, GeneID: 297334, Rattus norvegicus chr. 4<br>mitochondrial ribosomal protein L35 (predicted)	neur-nr2f	548 - 428	(-)
**GXP_253368** [GXP_253368] (1 - 1416)<br>**Tspan9_predicted**, GXL_212104, GeneID: 312728, Rattus norvegicus chr. 4<br>tetraspanin 9 (predicted)	neur-nr2f	71 - 189	(+)
**GXP_1414655** [GXP_1414655] (1 - 601)<br>**Grm8**, GXL_212130, GeneID: 60590, Rattus norvegicus chr. 4<br>glutamate receptor, metabotropic 8	neur-nr2f	373 - 516	(+)
**GXP_1414475** [GXP_1414475] (1 - 602)<br>**Nos3**, GXL_212140, GeneID: 24600, Rattus norvegicus chr. 4<br>nitric oxide synthase 3, endothelial cell	neur-nr2f	575 - 434	(-)
**GXP_1415382** [GXP_1415382] (1 - 641)<br>**Ntf3**, GXL_212141, GeneID: 81737, Rattus norvegicus chr. 4<br>neurotrophin 3	neur-nr2f	462 - 342	(-)
**GXP_253486** [GXP_253486] (1 - 611)<br>**Trh**, GXL_212219, GeneID: 25569, Rattus norvegicus chr. 4<br>thyrotropin releasing hormone	neur-nr2f	169 - 285	(+)
**GXP_1415330** [GXP_1415330] (1 - 618)<br>**Clstn3**, GXL_212309, GeneID: 171393, Rattus norvegicus chr. 4<br>calsyntenin 3	neur-nr2f	188 - 312	(+)
**GXP_258838** [GXP_258838] (1 - 601)<br>**Olr1_predicted**, GXL_216794, GeneID: 365170, Rattus norvegicus chr. 1<br>olfactory receptor 1 (predicted)	neur-nr2f	507 - 386	(-)
**GXP_258882** [GXP_258882] (1 - 1440)<br>**Lgi4**, GXL_216836, GeneID: 361549, Rattus norvegicus chr. 1<br>leucine-rich repeat LGI family, member 4	neur-nr2f	402 - 251	(-)
**GXP_258910** [GXP_258910] (1 - 700)<br>**Lypd3**, GXL_216864, GeneID: 60378, Rattus norvegicus chr. 1<br>Ly6/Plaur domain containing 3	neur-nr2f	674 - 546	(-)
**GXP_1157968** [GXP_1157968] (1 - 601)<br>**Syt3**, GXL_216876, GeneID: 25731, Rattus norvegicus chr. 1<br>synaptotagmin III	neur-nr2f	468 - 594	(+)
**GXP_258937** [GXP_258937] (1 - 1035)<br>**Nphs1**, GXL_216890, GeneID: 64563, Rattus norvegicus chr. 1<br>nephrosis 1 homolog, nephrin (human)	neur-nr2f	368 - 245	(-)
	neur-nr2f	543 - 406	(-)

**GXP_1409476** [GXP_1409476] (1 - 618)			
**Kcnc3**, GXL_216903, GeneID: 117101, Rattus norvegicus chr. 1			
potassium voltage gated channel, Shaw-related subfamily, member 3			
**GXP_258983** [GXP_258983] (1 - 601)			
**Ceacam3**, GXL_216933, GeneID: 24926, Rattus norvegicus chr. 1	neur-nr2f	334 - 211	(-)
carcinoembryonic antigen-related cell adhesion molecule 3			
**GXP_1409196** [GXP_1409196] (1 - 714)			
**Ckm**, GXL_217103, GeneID: 24265, Rattus norvegicus chr. 1	neur-nr2f	706 - 589	(-)
creatine kinase, muscle			
**GXP_259196** [GXP_259196] (1 - 601)			
**Pglyrp1**, GXL_217139, GeneID: 84387, Rattus norvegicus chr. 1	neur-nr2f	137 - 284	(+)
peptidoglycan recognition protein 1			
**GXP_259200** [GXP_259200] (1 - 1140)			
**Slc27a5**, GXL_217143, GeneID: 79111, Rattus norvegicus chr. 1	neur-nr2f	375 - 236	(-)
solute carrier family 27 (fatty acid transporter), member 5			
**GXP_1409528** [GXP_1409528] (1 - 601)			
**Ntf5**, GXL_217255, GeneID: 25730, Rattus norvegicus chr. 1	neur-nr2f	425 - 307	(-)
neurotrophin 5			
**GXP_1410549** [GXP_1410549] (1 - 602)			
**B3gnt6_predicted**, GXL_232973, GeneID: 293667, Rattus norvegicus chr. 1	neur-nr2f	507 - 366	(-)
UDP-GlcNAc:betaGal beta-1, 3-N-acetylglucosaminyltransferase 6 (predicted)			
**GXP_1411004** [GXP_1411004] (1 - 601)			
**Nolc1**, GXL_233143, GeneID: 64896, Rattus norvegicus chr. 1	neur-nr2f	237 - 389	(+)
nucleolar and coiled-body phosphoprotein 1			
**GXP_1410548** [GXP_1410548] (1 - 601)			
**Slc29a2**, GXL_233171, GeneID: 65194, Rattus norvegicus chr. 1	neur-nr2f	450 - 566	(+)
solute carrier family 29 (nucleoside transporters), member 2			
**GXP_1420287** [GXP_1420287] (1 - 668)			
**Il1rl1**, GXL_233349, GeneID: 25556, Rattus norvegicus chr. 9	neur-nr2f	613 - 499	(-)
interleukin 1 receptor-like 1			
**GXP_1420700** [GXP_1420700] (1 - 601)			
**Ngep**, GXL_233396, GeneID: 367318, Rattus norvegicus chr. 9	neur-nr2f	312 - 444	(+)
new gene expressed in prostate			
**GXP_865917** [GXP_865917] (1 - 601)			
**Ugt1a1**, GXL_233421, GeneID: 24861, Rattus norvegicus chr. 9	neur-nr2f	523 - 375	(-)
UDP glycosyltransferase 1 family, polypeptide A1			
**GXP_278591** [GXP_278591] (1 - 601)			
**Rpl37a_predicted**, GXL_233475, GeneID: 363248, Rattus norvegicus chr. 9	neur-nr2f	146 - 266	(+)
ribosomal protein L37a (predicted)			
	neur-nr2f	243 - 113	(-)

**GXP_1173524** [GXP_1173524] (1 - 889)			
**Abi2**, GXL_233484, GeneID: 286928, Rattus norvegicus chr. 9			
abl-interactor 2			
**GXP_1420509** [GXP_1420509] (1 - 1314)			
**Bcs1l**, GXL_233532, GeneID: 301514, Rattus norvegicus chr. 9	neur-nr2f	799 - 667	(-)
BCS1-like (yeast)			
**GXP_1420777** [GXP_1420777] (1 - 601)			
**Dlgap1**, GXL_233554, GeneID: 65040, Rattus norvegicus chr. 9	neur-nr2f	163 - 288	(+)
discs, large (Drosophila) homolog-associated protein 1			
**GXP_1415550** [GXP_1415550] (1 - 608)			
**Rdh10**, GXL_241568, GeneID: 353252, Rattus norvegicus chr. 5	neur-nr2f	307 - 186	(-)
retinol dehydrogenase 10 (all-trans)			
**GXP_287412** [GXP_287412] (1 - 601)			
**Dio1**, GXL_241575, GeneID: 25430, Rattus norvegicus chr. 5	neur-nr2f	306 - 437	(+)
deiodinase, iodothyronine, type I			
**GXP_287522** [GXP_287522] (1 - 601)			
**Olr866_predicted**, GXL_241681, GeneID: 298475, Rattus norvegicus chr. 5	neur-nr2f	306 - 434	(+)
olfactory receptor 866 (predicted)			
**GXP_1416241** [GXP_1416241] (1 - 1447)			
**Lrrc41**, GXL_241693, GeneID: 362566, Rattus norvegicus chr. 5	neur-nr2f	505 - 626	(+)
leucine rich repeat containing 41			
**GXP_1416175** [GXP_1416175] (1 - 602)			
**Mrpl37**, GXL_241818, GeneID: 56281, Rattus norvegicus chr. 5	neur-nr2f	539 - 413	(-)
mitochondrial ribosomal protein L37			
**GXP_1416021** [GXP_1416021] (1 - 601)			
**Mpdz**, GXL_241859, GeneID: 29365, Rattus norvegicus chr. 5	neur-nr2f	555 - 435	(-)
multiple PDZ domain protein			
**GXP_1416303** [GXP_1416303] (1 - 602)			
**Ptprf**, GXL_241910, GeneID: 360406, Rattus norvegicus chr. 5	neur-nr2f	390 - 275	(-)
protein tyrosine phosphatase, receptor type, F			
**GXP_287852** [GXP_287852] (1 - 655)			
**Dbccr1**, GXL_242008, GeneID: 140610, Rattus norvegicus chr. 5	neur-nr2f	642 - 525	(-)
deleted in bladder cancer chromosome region candidate 1 (human)			
**GXP_287857** [GXP_287857] (1 - 1062)			
**Atpbd1b_predicted**, GXL_242013, GeneID: 362614, Rattus norvegicus chr. 5	neur-nr2f	397 - 280	(-)
ATP binding domain 1 family, member B (predicted)			
**GXP_288196** [GXP_288196] (1 - 711)			
**Cog6**, GXL_242272, GeneID: 310411, Rattus norvegicus chr. 2	neur-nr2f	354 - 469	(+)
component of oligomeric golgi complex 6			
	neur-nr2f	159 - 40	(-)

**GXP_288228** [GXP_288228] (1 - 955)<br>**Pfn2**, GXL_242302, GeneID: 81531, Rattus norvegicus chr. 2<br>profilin 2			
**GXP_1411678** [GXP_1411678] (1 - 602)<br>**Sv2c**, GXL_242333, GeneID: 29643, Rattus norvegicus chr. 2<br>synaptic vesicle glycoprotein 2c	neur-nr2f	204 - 78	(-)
**GXP_355553** [GXP_355553] (1 - 601)<br>**Lmna**, GXL_242362, GeneID: 60374, Rattus norvegicus chr. 2<br>lamin A	neur-nr2f	359 - 490	(+)
**GXP_288367** [GXP_288367] (1 - 1085)<br>**Efna1**, GXL_242438, GeneID: 94268, Rattus norvegicus chr. 2<br>ephrin A1	neur-nr2f	45 - 179	(+)
**GXP_1412475** [GXP_1412475] (1 - 601)<br>**Pde4dip**, GXL_242523, GeneID: 64183, Rattus norvegicus chr. 2<br>phosphodiesterase 4D interacting protein (myomegalin)	neur-nr2f	484 - 349	(-)
**GXP_288509** [GXP_288509] (1 - 881)<br>**Pelo**, GXL_242579, GeneID: 294754, Rattus norvegicus chr. 2<br>pelota homolog	neur-nr2f	212 - 73	(-)
**GXP_1411671** [GXP_1411671] (1 - 613)<br>**Pde8b**, GXL_242650, GeneID: 309962, Rattus norvegicus chr. 2<br>phosphodiesterase 8B	neur-nr2f	2 - 118	(+)
**GXP_1412319** [GXP_1412319] (1 - 643)<br>**Rab25_predicted**, GXL_242656, GeneID: 310632, Rattus norvegicus chr. 2<br>RAB25, member RAS oncogene family (predicted)	neur-nr2f	195 - 325	(+)
**GXP_1414259** [GXP_1414259] (1 - 601)<br>**Svs2**, GXL_246083, GeneID: 25013, Rattus norvegicus chr. 3<br>seminal vesicle protein, secretion 2	neur-nr2f	430 - 287	(-)
**GXP_1414260** [GXP_1414260] (1 - 601)<br>**Svs2**, GXL_246083, GeneID: 25013, Rattus norvegicus chr. 3<br>seminal vesicle protein, secretion 2	neur-nr2f	432 - 289	(-)
**GXP_292703** [GXP_292703] (1 - 1219)<br>**Arfrp1**, GXL_246111, GeneID: 117051, Rattus norvegicus chr. 3<br>ADP-ribosylation factor related protein 1	neur-nr2f	712 - 559	(-)
**GXP_1412938** [GXP_1412938] (1 - 602)<br>**Kcnt1**, GXL_246117, GeneID: 60444, Rattus norvegicus chr. 3<br>potassium channel, subfamily T, member 1	neur-nr2f	153 - 287	(+)
**GXP_292721** [GXP_292721] (1 - 1030)<br>**Rgs19**, GXL_246129, GeneID: 59293, Rattus norvegicus chr. 3<br>regulator of G-protein signaling 19	neur-nr2f	924 - 784	(-)
	neur-nr2f	463 - 310	(-)

GXP_292790 [GXP_292790] (1 - 875)
Angptl2, GXL_246197, GeneID: 171100, Rattus norvegicus chr. 3
angiopoietin-like 2

GXP_1413981 [GXP_1413981] (1 - 1365)
Mkks, GXL_246314, GeneID: 311456, Rattus norvegicus chr. 3 — neur-nr2f 31 - 183 (+)
McKusick-Kaufman syndrome protein

GXP_1164811 [GXP_1164811] (1 - 601)
Stau1, GXL_246370, GeneID: 84496, Rattus norvegicus chr. 3 — neur-nr2f 405 - 260 (-)
staufen RNA binding protein homolog 1 (Drosophila)

GXP_1413705 [GXP_1413705] (1 - 602)
Plcb2, GXL_246397, GeneID: 85240, Rattus norvegicus chr. 3 — neur-nr2f 460 - 321 (-)
phospholipase C, beta 2

GXP_1164895 [GXP_1164895] (1 - 629)
Sycp2, GXL_246438, GeneID: 83820, Rattus norvegicus chr. 3 — neur-nr2f 101 - 250 (+)
synaptonemal complex protein 2

GXP_1405648 [GXP_1405648] (1 - 1064)
Pdgfra, GXL_247065, GeneID: 25267, Rattus norvegicus chr. 14 — neur-nr2f 900 - 1044 (+)
platelet derived growth factor receptor, alpha polypeptide

GXP_293856 [GXP_293856] (1 - 601)
Rpl5, GXL_247132, GeneID: 81763, Rattus norvegicus chr. 14 — neur-nr2f 121 - 3 (-)
ribosomal protein L5

GXP_1405574 [GXP_1405574] (1 - 626)
Ereg, GXL_247162, GeneID: 59325, Rattus norvegicus chr. 14 — neur-nr2f 261 - 387 (+)
epiregulin

GXP_1405491 [GXP_1405491] (1 - 602)
Arhgap24, GXL_247228, GeneID: 305156, Rattus norvegicus chr. 14 — neur-nr2f 169 - 311 (+)
Rho GTPase activating protein 24

GXP_293981 [GXP_293981] (1 - 601)
Ccng2_predicted, GXL_247255, GeneID: 29157, Rattus norvegicus chr. 14 — neur-nr2f 360 - 496 (+)
cyclin G2 (predicted)

GXP_302445 [GXP_302445] (1 - 601)
Olr7_predicted, GXL_254115, GeneID: 405043, Rattus norvegicus chr. 1 — neur-nr2f 525 - 378 (-)
olfactory receptor 7 (predicted)

GXP_309296 [GXP_309296] (1 - 710)
Spink1, GXL_260107, GeneID: 24833, Rattus norvegicus chr. 18 — neur-nr2f 521 - 654 (+)
serine protease inhibitor, Kazal type 1

GXP_1408105 [GXP_1408105] (1 - 602)
Stard6, GXL_260132, GeneID: 291527, Rattus norvegicus chr. 18 — neur-nr2f 332 - 189 (-)
STAR-related lipid transfer (START) domain containing protein 6

neur-nr2f 162 - 24 (-)

**GXP_309383** [GXP_309383] (1 - 898)<br>**Atp5a1**, GXL_260179, GeneID: 65262, Rattus norvegicus chr. 18<br>ATP synthase, H+ transporting, mitochondrial F1 complex, alpha subunit, isoform 1			
**GXP_1407874** [GXP_1407874] (1 - 621)<br>**Cxxc5**, GXL_260256, GeneID: 291670, Rattus norvegicus chr. 18<br>CXXC finger 5	neur-nr2f<br>neur-nr2f	459 - 579<br>472 - 342	(+)<br>(-)
**GXP_1408192** [GXP_1408192] (1 - 602)<br>**Cndp2**, GXL_260267, GeneID: 291394, Rattus norvegicus chr. 18<br>CNDP dipeptidase 2 (metallopeptidase M20 family)	neur-nr2f	140 - 279	(+)
**GXP_309558** [GXP_309558] (1 - 832)<br>**Txnl1**, GXL_260349, GeneID: 140922, Rattus norvegicus chr. 18<br>thioredoxin-like 1	neur-nr2f	125 - 244	(+)
**GXP_1407863** [GXP_1407863] (1 - 602)<br>**Catna1**, GXL_260411, GeneID: 307505, Rattus norvegicus chr. 18<br>catenin (cadherin-associated protein), alpha 1	neur-nr2f	278 - 152	(-)
**GXP_1408128** [GXP_1408128] (1 - 602)<br>**Acaa2**, GXL_260536, GeneID: 170465, Rattus norvegicus chr. 18<br>acetyl-Coenzyme A acyltransferase 2 (mitochondrial 3-oxoacyl-Coenzyme A thiolase)	neur-nr2f	537 - 412	(-)
**GXP_312760** [GXP_312760] (1 - 950)<br>**Sucla2_predicted**, GXL_262999, GeneID: 361071, Rattus norvegicus chr. 15<br>succinate-Coenzyme A ligase, ADP-forming, beta subunit (predicted)	neur-nr2f	808 - 665	(-)
**GXP_1406598** [GXP_1406598] (1 - 601)<br>**Tdrd3**, GXL_263094, GeneID: 306066, Rattus norvegicus chr. 15<br>tudor domain containing 3	neur-nr2f	521 - 405	(-)
**GXP_312933** [GXP_312933] (1 - 679)<br>**Nufip1**, GXL_263172, GeneID: 364430, Rattus norvegicus chr. 15<br>nuclear fragile X mental retardation protein interacting protein 1	neur-nr2f	510 - 363	(-)
**GXP_313057** [GXP_313057] (1 - 601)<br>**Udpgtr2**, GXL_263281, GeneID: 286954, Rattus norvegicus chr. 14<br>liver UDP-glucuronosyltransferase, phenobarbital-inducible form	neur-nr2f	279 - 149	(-)
**GXP_1406047** [GXP_1406047] (1 - 1424)<br>**Vps54**, GXL_263293, GeneID: 286932, Rattus norvegicus chr. 14<br>vacuolar protein sorting 54 (yeast)	neur-nr2f	579 - 453	(-)
**GXP_313107** [GXP_313107] (1 - 601)<br>**Tmem17**, GXL_263330, GeneID: 360985, Rattus norvegicus chr. 14<br>transmembrane protein 17	neur-nr2f	573 - 434	(-)
**GXP_1405979** [GXP_1405979] (1 - 601)<br>**Spergen2**, GXL_263336, GeneID: 305491, Rattus norvegicus chr. 14<br>spermatogenic cell-specific gene 2	neur-nr2f<br>neur-nr2f	7 - 147<br>373 - 249	(+)<br>(-)

**GXP_313217** [GXP_313217] (1 - 601)			
**Ugt2a1**, GXL_263438, GeneID: 63867, Rattus norvegicus chr. 14			
UDP glucuronosyltransferase 2 family, polypeptide A1			
**GXP_1405602** [GXP_1405602] (1 - 672)			
**Csn2**, GXL_263442, GeneID: 29173, Rattus norvegicus chr. 14	neur-nr2f	296 - 449	(+)
casein beta			
**GXP_313249** [GXP_313249] (1 - 601)			
**Gnrhr**, GXL_263468, GeneID: 81668, Rattus norvegicus chr. 14	neur-nr2f	312 - 426	(+)
gonadotropin releasing hormone receptor			
**GXP_1405940** [GXP_1405940] (1 - 603)			
**Sf3a1_predicted**, GXL_263482, GeneID: 305479, Rattus norvegicus chr. 14	neur-nr2f	341 - 468	(+)
splicing factor 3a, subunit 1 (predicted)			
**GXP_1405941** [GXP_1405941] (1 - 602)			
**Sf3a1_predicted**, GXL_263482, GeneID: 305479, Rattus norvegicus chr. 14	neur-nr2f	183 - 302	(+)
splicing factor 3a, subunit 1 (predicted)			
**GXP_313272** [GXP_313272] (1 - 666)			
**Htra3_predicted**, GXL_263491, GeneID: 360959, Rattus norvegicus chr. 14	neur-nr2f	355 - 230	(-)
HtrA serine peptidase 3 (predicted)			
**GXP_314996** [GXP_314996] (1 - 642)			
**Cd48**, GXL_264972, GeneID: 245962, Rattus norvegicus chr. 13	neur-nr2f	218 - 101	(-)
CD48 antigen			
**GXP_315038** [GXP_315038] (1 - 672)			
**Slamf9_predicted**, GXL_265013, GeneID: 289235, Rattus norvegicus chr. 13	neur-nr2f	294 - 169	(-)
SLAM family member 9 (predicted)			
**GXP_1157032** [GXP_1157032] (1 - 601)			
**Rps6ka2**, GXL_317448, GeneID: 117269, Rattus norvegicus chr. 1	neur-nr2f	120 - 265	(+)
ribosomal protein S6 kinase polypeptide 2			
**GXP_495515** [GXP_495515] (1 - 601)			
**Psg16_predicted**, GXL_317659, GeneID: 24257, Rattus norvegicus chr. 1	neur-nr2f	417 - 294	(-)
pregnancy specific glycoprotein 16 (predicted)			
**GXP_1409301** [GXP_1409301] (1 - 602)			
**Samd4b**, GXL_317747, GeneID: 308473, Rattus norvegicus chr. 1	neur-nr2f	155 - 24	(-)
sterile alpha motif domain containing 4B			
**GXP_495741** [GXP_495741] (1 - 822)			
**Map4k1_predicted**, GXL_317754, GeneID: 292763, Rattus norvegicus chr. 1	neur-nr2f	231 - 373	(+)
mitogen activated protein kinase kinase kinase kinase 1 (predicted)			
**GXP_495892** [GXP_495892] (1 - 602)			
**Gramd1a**, GXL_317805, GeneID: 361550, Rattus norvegicus chr. 1	neur-nr2f	130 - 1	(-)
GRAM domain containing 1A			
	neur-nr2f	469 - 324	(-)

**GXP_496190** [GXP_496190] (1 - 1317)<br>**Kdelr1**, GXL_317916, GeneID: 361577, Rattus norvegicus chr. 1<br>KDEL (Lys-Asp-Glu-Leu) endoplasmic reticulum protein retention receptor 1			
**GXP_1410073** [GXP_1410073] (1 - 601)<br>**Arntl**, GXL_318178, GeneID: 29657, Rattus norvegicus chr. 1<br>aryl hydrocarbon receptor nuclear translocator-like	neur-nr2f	202 - 349	(+)
**GXP_1410212** [GXP_1410212] (1 - 602)<br>**Eif3s8**, GXL_318245, GeneID: 293484, Rattus norvegicus chr. 1<br>eukaryotic translation initiation factor 3, subunit 8, 110kDa	neur-nr2f	335 - 221	(-)
**GXP_850539** [GXP_850539] (1 - 607)<br>**Tcirg1**, GXL_318406, GeneID: 293650, Rattus norvegicus chr. 1<br>T-cell, immune regulator 1	neur-nr2f	414 - 552	(+)
**GXP_497662** [GXP_497662] (1 - 602)<br>**Cnih2**, GXL_318424, GeneID: 361705, Rattus norvegicus chr. 1<br>cornichon homolog 2 (Drosophila)	neur-nr2f	32 - 171	(+)
**GXP_497843** [GXP_497843] (1 - 754)<br>**Taf6l_predicted**, GXL_318477, GeneID: 309194, Rattus norvegicus chr. 1<br>TAF6-like RNA polymerase II, p300/CBP-associated factor (PCAF)-associated factor (predicted)	neur-nr2f	233 - 382	(+)
**GXP_1410649** [GXP_1410649] (1 - 733)<br>**Taf6l_predicted**, GXL_318477, GeneID: 309194, Rattus norvegicus chr. 1<br>TAF6-like RNA polymerase II, p300/CBP-associated factor (PCAF)-associated factor (predicted)	neur-nr2f	323 - 191	(-)
**GXP_497852** [GXP_497852] (1 - 650)<br>**Ganab_predicted**, GXL_318481, GeneID: 293721, Rattus norvegicus chr. 1<br>alpha glucosidase 2 alpha neutral subunit (predicted)	neur-nr2f	48 - 178	(+)
**GXP_850705** [GXP_850705] (1 - 601)<br>**Ganab_predicted**, GXL_318481, GeneID: 293721, Rattus norvegicus chr. 1<br>alpha glucosidase 2 alpha neutral subunit (predicted)	neur-nr2f	354 - 484	(+)
**GXP_1410904** [GXP_1410904] (1 - 698)<br>**Cep55**, GXL_318610, GeneID: 294074, Rattus norvegicus chr. 1<br>centrosomal protein 55	neur-nr2f	545 - 427	(-)
**GXP_1410963** [GXP_1410963] (1 - 602)<br>**Cwf19l1_predicted**, GXL_318651, GeneID: 365465, Rattus norvegicus chr. 1<br>CWF19-like 1, cell cycle control (S. pombe) (predicted)	neur-nr2f	392 - 240	(-)
**GXP_498338** [GXP_498338] (1 - 601)<br>**Poll**, GXL_318669, GeneID: 361767, Rattus norvegicus chr. 1<br>polymerase (DNA directed), lambda	neur-nr2f	188 - 36	(-)
**GXP_498450** [GXP_498450] (1 - 739)<br>**Ins1**, GXL_318706, GeneID: 24505, Rattus norvegicus chr. 1<br>insulin 1	neur-nr2f	399 - 255	(-)
	neur-nr2f	386 - 246	(-)

**GXP_1402161** [GXP_1402161] (1 - 601)<br>**Zc3h7a_predicted**, GXL_318765, GeneID: 360466, Rattus norvegicus chr. 10<br>zinc finger CCCH type containing 7 A (predicted)			
**GXP_1147259** [GXP_1147259] (1 - 601)<br>**Sqstm1**, GXL_318931, GeneID: 113894, Rattus norvegicus chr. 10<br>sequestosome 1	neur-nr2f	307 - 444	(+)
**GXP_1147458** [GXP_1147458] (1 - 601)<br>**Map2k3**, GXL_319020, GeneID: 303200, Rattus norvegicus chr. 10<br>mitogen activated protein kinase kinase 3	neur-nr2f	144 - 260	(+)
**GXP_499296** [GXP_499296] (1 - 673)<br>**Mfap4**, GXL_319025, GeneID: 287382, Rattus norvegicus chr. 10<br>microfibrillar-associated protein 4	neur-nr2f	234 - 374	(+)
**GXP_1402796** [GXP_1402796] (1 - 602)<br>**Rutbc1_predicted**, GXL_319143, GeneID: 303304, Rattus norvegicus chr. 10<br>RUN and TBC1 domain containing 1 (predicted)	neur-nr2f	217 - 348	(+)
**GXP_499948** [GXP_499948] (1 - 671)<br>**Mks1**, GXL_319258, GeneID: 287612, Rattus norvegicus chr. 10<br>Meckel syndrome, type 1	neur-nr2f	344 - 220	(-)
**GXP_500185** [GXP_500185] (1 - 601)<br>**Thrap4**, GXL_319339, GeneID: 619436, Rattus norvegicus chr. 10<br>thyroid hormone receptor associated protein 4	neur-nr2f	223 - 83	(-)
**GXP_1403263** [GXP_1403263] (1 - 601)<br>**Vat1**, GXL_319378, GeneID: 287721, Rattus norvegicus chr. 10<br>vesicle amine transport protein 1 homolog (T californica)	neur-nr2f	190 - 63	(-)
**GXP_500389** [GXP_500389] (1 - 777)<br>**Slc4a1**, GXL_319400, GeneID: 24779, Rattus norvegicus chr. 10<br>solute carrier family 4, member 1	neur-nr2f	732 - 580	(-)
**GXP_500611** [GXP_500611] (1 - 601)<br>**Sox9**, GXL_319475, GeneID: 140586, Rattus norvegicus chr. 10<br>SRY-box containing gene 9	neur-nr2f	162 - 36	(-)
**GXP_500703** [GXP_500703] (1 - 686)<br>**Pcnt1**, GXL_319519, GeneID: 287830, Rattus norvegicus chr. 10<br>pericentrin 1	neur-nr2f	517 - 373	(-)
**GXP_1403479** [GXP_1403479] (1 - 601)<br>**Mif4gd**, GXL_319521, GeneID: 360659, Rattus norvegicus chr. 10<br>MIF4G domain containing	neur-nr2f	208 - 356	(+)
**GXP_1148706** [GXP_1148706] (1 - 601)<br>**Srp68_predicted**, GXL_319532, GeneID: 363707, Rattus norvegicus chr. 10<br>signal recognition particle 68 (predicted)	neur-nr2f	332 - 202	(-)
	neur-nr2f	242 - 363	(+)

**GXP_501064** [GXP_501064] (1 - 614)<br>**Mx1**, GXL_319662, GeneID: 24575, Rattus norvegicus chr. 11<br>myxovirus (influenza virus) resistance 1	neur-nr2f	379 - 496	(+)
**GXP_1404070** [GXP_1404070] (1 - 601)<br>**Chrd**, GXL_319834, GeneID: 117275, Rattus norvegicus chr. 11<br>chordin	neur-nr2f	181 - 302	(+)
**GXP_501696** [GXP_501696] (1 - 708)<br>**Lrrc8e**, GXL_319899, GeneID: 304203, Rattus norvegicus chr. 12<br>leucine rich repeat containing 8 family, member E	neur-nr2f	312 - 187	(-)
**GXP_502069** [GXP_502069] (1 - 1094)<br>**Mospd3**, GXL_320050, GeneID: 288557, Rattus norvegicus chr. 12<br>motile sperm domain containing 3	neur-nr2f	849 - 972	(+)
**GXP_502097** [GXP_502097] (1 - 855)<br>**Cldn15_predicted**, GXL_320067, GeneID: 304388, Rattus norvegicus chr. 12<br>claudin 15 (predicted)	neur-nr2f	175 - 303	(+)
**GXP_502242** [GXP_502242] (1 - 601)<br>**Ncor2_predicted**, GXL_320117, GeneID: 360801, Rattus norvegicus chr. 12<br>nuclear receptor co-repressor 2 (predicted)	neur-nr2f	209 - 343	(+)
**GXP_502370** [GXP_502370] (1 - 735)<br>**Fln29**, GXL_320155, GeneID: 114635, Rattus norvegicus chr. 12<br>FLN29 gene product	neur-nr2f	411 - 286	(-)
**GXP_1404979** [GXP_1404979] (1 - 601)<br>**Glrx2**, GXL_320365, GeneID: 114022, Rattus norvegicus chr. 13<br>glutaredoxin 2 (thioltransferase)	neur-nr2f	238 - 388	(+)
**GXP_503409** [GXP_503409] (1 - 982)<br>**Sccpdh**, GXL_320546, GeneID: 305021, Rattus norvegicus chr. 13<br>saccharopine dehydrogenase (putative)	neur-nr2f	68 - 216	(+)
**GXP_1152196** [GXP_1152196] (1 - 601)<br>**Tbc1d10a**, GXL_320906, GeneID: 360968, Rattus norvegicus chr. 14<br>TBC1 domain family, member 10a	neur-nr2f	174 - 288	(+)
**GXP_1406007** [GXP_1406007] (1 - 629)<br>**Upp1**, GXL_320941, GeneID: 289801, Rattus norvegicus chr. 14<br>uridine phosphorylase 1	neur-nr2f	357 - 510	(+)
**GXP_1406109** [GXP_1406109] (1 - 1187)<br>**Spnb2**, GXL_321003, GeneID: 305614, Rattus norvegicus chr. 14<br>spectrin beta 2	neur-nr2f	132 - 280	(+)
**GXP_1406325** [GXP_1406325] (1 - 602)<br>**Acin1**, GXL_321206, GeneID: 305884, Rattus norvegicus chr. 15<br>apoptotic chromatin condensation inducer 1	neur-nr2f	450 - 308	(-)
	neur-nr2f	413 - 533	(+)

**GXP_1406355** [GXP_1406355] (1 - 1059)<br>**Gmpr2**, GXL_321224, GeneID: 192357, Rattus norvegicus chr. 15<br>guanosine monophosphate reductase 2			
**GXP_505414** [GXP_505414] (1 - 601)<br>**Ppp3cc**, GXL_321338, GeneID: 171378, Rattus norvegicus chr. 15<br>protein phosphatase 3, catalytic subunit, gamma isoform	neur-nr2f	274 - 425	(+)
**GXP_1406512** [GXP_1406512] (1 - 602)<br>**Ppp3cc**, GXL_321338, GeneID: 171378, Rattus norvegicus chr. 15<br>protein phosphatase 3, catalytic subunit, gamma isoform	neur-nr2f	218 - 350	(+)
**GXP_505542** [GXP_505542] (1 - 1108)<br>**Wbp4**, GXL_321382, GeneID: 114765, Rattus norvegicus chr. 15<br>WW domain binding protein 4	neur-nr2f	362 - 243	(-)
**GXP_505737** [GXP_505737] (1 - 601)<br>**Actr8_predicted**, GXL_321459, GeneID: 361107, Rattus norvegicus chr. 16<br>ARP8 actin-related protein 8 homolog (S. cerevisiae) (predicted)	neur-nr2f	304 - 445	(+)
**GXP_1406822** [GXP_1406822] (1 - 601)<br>**Tspan14_predicted**, GXL_321535, GeneID: 306324, Rattus norvegicus chr. 16<br>tetraspanin 14 (predicted)	neur-nr2f	536 - 388	(-)
**GXP_506476** [GXP_506476] (1 - 635)<br>**Defb12**, GXL_321760, GeneID: 641643, Rattus norvegicus chr. 16<br>defensin beta 12	neur-nr2f	403 - 289	(-)
**GXP_507580** [GXP_507580] (1 - 1195)<br>**Nol4_predicted**, GXL_322266, GeneID: 307553, Rattus norvegicus chr. 18<br>nucleolar protein 4 (predicted)	neur-nr2f	153 - 306	(+)
**GXP_1407784** [GXP_1407784] (1 - 601)<br>**Nol4_predicted**, GXL_322266, GeneID: 307553, Rattus norvegicus chr. 18<br>nucleolar protein 4 (predicted)	neur-nr2f	437 - 590	(+)
**GXP_1407812** [GXP_1407812] (1 - 603)<br>**Wdr33_predicted**, GXL_322289, GeneID: 307524, Rattus norvegicus chr. 18<br>WD repeat domain 33 (predicted)	neur-nr2f	235 - 89	(-)
**GXP_507963** [GXP_507963] (1 - 817)<br>**Pde6a_predicted**, GXL_322424, GeneID: 307401, Rattus norvegicus chr. 18<br>phosphodiesterase 6A, cGMP-specific, rod, alpha (predicted)	neur-nr2f	4 - 134	(+)
**GXP_508193** [GXP_508193] (1 - 1037)<br>**Cbln2**, GXL_322517, GeneID: 291388, Rattus norvegicus chr. 18<br>cerebellin 2 precursor protein	neur-nr2f	418 - 553	(+)
**GXP_1408211** [GXP_1408211] (1 - 750)<br>**Cmtm3_predicted**, GXL_322532, GeneID: 291813, Rattus norvegicus chr. 19<br>CKLF-like MARVEL transmembrane domain containing 3 (predicted)	neur-nr2f	401 - 273	(-)
	neur-nr2f	486 - 358	(-)

**GXP_1408212** [GXP_1408212] (1 - 602)<br>**Cmtm3_predicted**, GXL_322532, GeneID: 291813, Rattus norvegicus chr. 19<br>CKLF-like MARVEL transmembrane domain containing 3 (predicted)			
**GXP_1408246** [GXP_1408246] (1 - 704)<br>**Katnb1**, GXL_322557, GeneID: 291852, Rattus norvegicus chr. 19<br>katanin p80 (WD40-containing) subunit B 1	neur-nr2f	669 - 549	(-)
**GXP_1408398** [GXP_1408398] (1 - 606)<br>**Rnaseh2a**, GXL_322635, GeneID: 364974, Rattus norvegicus chr. 19<br>ribonuclease H2, large subunit	neur-nr2f	229 - 109	(-)
**GXP_1408608** [GXP_1408608] (1 - 602)<br>**Txnl4b**, GXL_322725, GeneID: 292008, Rattus norvegicus chr. 19<br>thioredoxin-like 4B	neur-nr2f	182 - 63	(-)
**GXP_508945** [GXP_508945] (1 - 703)<br>**Aprt_predicted**, GXL_322795, GeneID: 292072, Rattus norvegicus chr. 19<br>adenine phosphoribosyl transferase (predicted)	neur-nr2f	240 - 382	(+)
**GXP_509340** [GXP_509340] (1 - 674)<br>**Ercc8_predicted**, GXL_322948, GeneID: 310071, Rattus norvegicus chr. 2<br>excision repair cross-complementing rodent repair deficiency, complementation group 8 (predicted)	neur-nr2f	280 - 154	(-)
**GXP_1411861** [GXP_1411861] (1 - 1180)<br>**Prlr**, GXL_323004, GeneID: 24684, Rattus norvegicus chr. 2<br>prolactin receptor	neur-nr2f	445 - 331	(-)
**GXP_1412129** [GXP_1412129] (1 - 602)<br>**Alg5**, GXL_323189, GeneID: 295051, Rattus norvegicus chr. 2<br>asparagine-linked glycosylation 5 homolog (yeast, dolichyl-phosphate beta-glucosyltransferase)	neur-nr2f	222 - 360	(+)
**GXP_1412173** [GXP_1412173] (1 - 601)<br>**Tiparp_predicted**, GXL_323223, GeneID: 310467, Rattus norvegicus chr. 2<br>TCDD-inducible poly(ADP-ribose) polymerase (predicted)	neur-nr2f	164 - 316	(+)
**GXP_510142** [GXP_510142] (1 - 1065)<br>**Insrr**, GXL_323280, GeneID: 60663, Rattus norvegicus chr. 2<br>insulin receptor-related receptor	neur-nr2f	247 - 374	(+)
**GXP_510390** [GXP_510390] (1 - 1157)<br>**Mllt11**, GXL_323369, GeneID: 295264, Rattus norvegicus chr. 2<br>myeloid/lymphoid or mixed-lineage leukemia (trithorax homolog, Drosophila), translocated to, 11	neur-nr2f	583 - 716	(+)
**GXP_1411125** [GXP_1411125] (1 - 603)<br>**Gabbr1**, GXL_323632, GeneID: 81657, Rattus norvegicus chr. 20<br>gamma-aminobutyric acid (GABA) B receptor 1	neur-nr2f	377 - 511	(+)
**GXP_511243** [GXP_511243] (1 - 638)<br>**Zfp297**, GXL_323657, GeneID: 309630, Rattus norvegicus chr. 20<br>zinc finger protein 297	neur-nr2f	362 - 224	(-)
	neur-nr2f	480 - 342	(-)

GXP_1160330 [GXP_1160330] (1 - 601)
Zfp297, GXL_323657, GeneID: 309630, Rattus norvegicus chr. 20
zinc finger protein 297

GXP_1411378 [GXP_1411378] (1 - 601)
Hrmt1l1, GXL_323724, GeneID: 499420, Rattus norvegicus chr. 20 — neur-nr2f 551 - 423 (-)
HMT1 hnRNP methyltransferase-like 1 (S. cerevisiae)

GXP_512408 [GXP_512408] (1 - 601)
Nr4a2, GXL_324134, GeneID: 54278, Rattus norvegicus chr. 3 — neur-nr2f 423 - 553 (+)
nuclear receptor subfamily 4, group A, member 2

GXP_1413494 [GXP_1413494] (1 - 601)
Pacsin3, GXL_324249, GeneID: 311187, Rattus norvegicus chr. 3 — neur-nr2f 198 - 83 (-)
protein kinase C and casein kinase substrate in neurons 3

GXP_1413497 [GXP_1413497] (1 - 602)
Pacsin3, GXL_324249, GeneID: 311187, Rattus norvegicus chr. 3 — neur-nr2f 65 - 205 (+)
protein kinase C and casein kinase substrate in neurons 3

GXP_1413558 [GXP_1413558] (1 - 602)
Alkbh3, GXL_324270, GeneID: 362169, Rattus norvegicus chr. 3 — neur-nr2f 383 - 534 (+)
alkB, alkylation repair homolog 3 (E. coli)

GXP_1413591 [GXP_1413591] (1 - 982)
Elf5_predicted, GXL_324293, GeneID: 366142, Rattus norvegicus chr. 3 — neur-nr2f 706 - 850 (+)
E74-like factor 5 (predicted)

GXP_1413691 [GXP_1413691] (1 - 602)
Eif2ak4_predicted, GXL_324352, GeneID: 114859, Rattus norvegicus chr. 3 — neur-nr2f 204 - 76 (-)
eukaryotic translation initiation factor 2 alpha kinase 4 (predicted)

GXP_513645 [GXP_513645] (1 - 642)
Rem1, GXL_324565, GeneID: 366232, Rattus norvegicus chr. 3 — neur-nr2f 546 - 405 (-)
rad and gem related GTP binding protein 1

GXP_1414173 [GXP_1414173] (1 - 647)
Rnpc2, GXL_324607, GeneID: 362251, Rattus norvegicus chr. 3 — neur-nr2f 31 - 146 (+)
RNA-binding region (RNP1, RRM) containing 2

GXP_1414275 [GXP_1414275] (1 - 723)
Tnnc2, GXL_324666, GeneID: 296369, Rattus norvegicus chr. 3 — neur-nr2f 151 - 279 (+)
troponin C type 2 (fast)

GXP_514209 [GXP_514209] (1 - 779)
Polr3k, GXL_324761, GeneID: 366277, Rattus norvegicus chr. 3 — neur-nr2f 371 - 512 (+)
polymerase (RNA) III (DNA directed) polypeptide K

GXP_514312 [GXP_514312] (1 - 789)
Lrrc17, GXL_324798, GeneID: 502715, Rattus norvegicus chr. 4 — neur-nr2f 368 - 482 (+)
leucine rich repeat containing 17

neur-nr2f 408 - 286 (-)

**GXP_514538** [GXP_514538] (1 - 662)			
**Wnt16**, GXL_324895, GeneID: 500047, Rattus norvegicus chr. 4			
wingless-related MMTV integration site 16			
**GXP_1415148** [GXP_1415148] (1 - 638)			
**Foxp1**, GXL_325320, GeneID: 297480, Rattus norvegicus chr. 4	neur-nr2f	200 - 345	(+)
forkhead box P1			
**GXP_1415185** [GXP_1415185] (1 - 601)			
**Cpne9**, GXL_325351, GeneID: 297516, Rattus norvegicus chr. 4	neur-nr2f	237 - 105	(-)
copine family member IX			
**GXP_1415278** [GXP_1415278] (1 - 1000)			
**Adipor2**, GXL_325391, GeneID: 312670, Rattus norvegicus chr. 4	neur-nr2f	388 - 521	(+)
adiponectin receptor 2			
**GXP_1415445** [GXP_1415445] (1 - 602)			
**Ddx47**, GXL_325502, GeneID: 297685, Rattus norvegicus chr. 4	neur-nr2f	272 - 413	(+)
DEAD (Asp-Glu-Ala-Asp) box polypeptide 47			
**GXP_1415558** [GXP_1415558] (1 - 603)			
**Lactb2**, GXL_325568, GeneID: 297768, Rattus norvegicus chr. 5	neur-nr2f	108 - 227	(+)
lactamase, beta 2			
**GXP_1415593** [GXP_1415593] (1 - 602)			
**Adhfe1**, GXL_325584, GeneID: 362474, Rattus norvegicus chr. 5	neur-nr2f	507 - 361	(-)
alcohol dehydrogenase, iron containing, 1			
**GXP_516324** [GXP_516324] (1 - 666)			
**Wwp1**, GXL_325656, GeneID: 297930, Rattus norvegicus chr. 5	neur-nr2f	167 - 48	(-)
WW domain containing E3 ubiquitin protein ligase 1			
**GXP_1415691** [GXP_1415691] (1 - 602)			
**Wwp1**, GXL_325656, GeneID: 297930, Rattus norvegicus chr. 5	neur-nr2f	331 - 209	(-)
WW domain containing E3 ubiquitin protein ligase 1			
**GXP_1415856** [GXP_1415856] (1 - 601)			
**Igfbpl1_predicted**, GXL_325744, GeneID: 366366, Rattus norvegicus chr. 5	neur-nr2f	289 - 164	(-)
insulin-like growth factor binding protein-like 1 (predicted)			
**GXP_519508** [GXP_519508] (1 - 601)			
**Olr991_predicted**, GXL_326881, GeneID: 500780, Rattus norvegicus chr. 7	neur-nr2f	551 - 404	(-)
olfactory receptor 991 (predicted)			
**GXP_1169886** [GXP_1169886] (1 - 601)			
**Rexo1**, GXL_326914, GeneID: 314630, Rattus norvegicus chr. 7	neur-nr2f	270 - 387	(+)
REX1, RNA exonuclease 1 homolog (S. cerevisiae)			
**GXP_1417947** [GXP_1417947] (1 - 602)			
**Tcp11l2**, GXL_327008, GeneID: 314683, Rattus norvegicus chr. 7	neur-nr2f	179 - 327	(+)
t-complex 11 (mouse) like 2			
	neur-nr2f	519 - 403	(-)

**GXP_1418049** [GXP_1418049] (1 - 601)			
**Dspg3_predicted**, GXL_327058, GeneID: 314772, Rattus norvegicus chr. 7			
dermatan sulphate proteoglycan 3 (predicted)			
**GXP_1418095** [GXP_1418095] (1 - 601)			
**Syt1**, GXL_327074, GeneID: 25716, Rattus norvegicus chr. 7	neur-nr2f	478 - 337	(-)
synaptotagmin I			
**GXP_1170496** [GXP_1170496] (1 - 601)			
**Ankrd46**, GXL_327167, GeneID: 299982, Rattus norvegicus chr. 7	neur-nr2f	88 - 241	(+)
ankyrin repeat domain 46			
**GXP_1170923** [GXP_1170923] (1 - 901)			
**Grap2**, GXL_327350, GeneID: 366962, Rattus norvegicus chr. 7	neur-nr2f	721 - 867	(+)
GRB2-related adaptor protein 2			
**GXP_1418569** [GXP_1418569] (1 - 602)			
**Pacsin2**, GXL_327374, GeneID: 124461, Rattus norvegicus chr. 7	neur-nr2f	283 - 426	(+)
protein kinase C and casein kinase substrate in neurons 2			
**GXP_521012** [GXP_521012] (1 - 601)			
**Hdac7a**, GXL_327444, GeneID: 84582, Rattus norvegicus chr. 7	neur-nr2f	261 - 147	(-)
histone deacetylase 7A			
**GXP_521605** [GXP_521605] (1 - 601)			
**Pvrl1**, GXL_327675, GeneID: 192183, Rattus norvegicus chr. 8	neur-nr2f	454 - 571	(+)
poliovirus receptor-related 1			
**GXP_1419250** [GXP_1419250] (1 - 628)			
**Slc35f2_predicted**, GXL_327738, GeneID: 300713, Rattus norvegicus chr. 8	neur-nr2f	169 - 320	(+)
solute carrier family 35, member F2 (predicted)			
**GXP_522431** [GXP_522431] (1 - 1468)			
**Dnajc13_predicted**, GXL_327962, GeneID: 363127, Rattus norvegicus chr. 8	neur-nr2f	1209 - 1070	(-)
DnaJ (Hsp40) homolog, subfamily C, member 13 (predicted)			
**GXP_1419732** [GXP_1419732] (1 - 661)			
**Pcbp4_predicted**, GXL_327974, GeneID: 363133, Rattus norvegicus chr. 8	neur-nr2f	357 - 234	(-)
poly(rC) binding protein 4 (predicted)			
**GXP_522593** [GXP_522593] (1 - 601)			
**Ihpk2**, GXL_328007, GeneID: 59268, Rattus norvegicus chr. 8	neur-nr2f	2 - 155	(+)
inositol hexaphosphate kinase 2			
**GXP_1419882** [GXP_1419882] (1 - 1158)			
**Dclk3_predicted**, GXL_328036, GeneID: 316023, Rattus norvegicus chr. 8	neur-nr2f	251 - 369	(+)
doublecortin-like kinase 3 (predicted)			
**GXP_522781** [GXP_522781] (1 - 601)			
**Xylb**, GXL_328071, GeneID: 316067, Rattus norvegicus chr. 8	neur-nr2f	471 - 341	(-)
xylulokinase homolog (H. influenzae)			
	neur-nr2f	157 - 38	(-)

**GXP_1172853** [GXP_1172853] (1 - 601)<br>**Gup1_predicted**, GXL_328085, GeneID: 301073, Rattus norvegicus chr. 8<br>Gup1, glycerol uptake/transporter homolog (yeast) (predicted)			
**GXP_1420678** [GXP_1420678] (1 - 612)<br>**Hes6**, GXL_328463, GeneID: 316626, Rattus norvegicus chr. 9<br>hairy and enhancer of split 6 (Drosophila)	neur-nr2f	517 - 370	(-)
**GXP_1420842** [GXP_1420842] (1 - 601)<br>**Dock11**, GXL_328563, GeneID: 313438, Rattus norvegicus chr. X<br>dedicator of cytokinesis 11	neur-nr2f	330 - 189	(-)
**GXP_1420874** [GXP_1420874] (1 - 602)<br>**Fundc1**, GXL_328587, GeneID: 363442, Rattus norvegicus chr. X<br>FUN14 domain containing 1	neur-nr2f	146 - 24	(-)
**GXP_1174372** [GXP_1174372] (1 - 601)<br>**Maged2**, GXL_328670, GeneID: 113947, Rattus norvegicus chr. X<br>melanoma antigen, family D, 2	neur-nr2f	177 - 40	(-)
**GXP_1421017** [GXP_1421017] (1 - 602)<br>**Msl31**, GXL_328689, GeneID: 317464, Rattus norvegicus chr. X<br>male-specific lethal-3 homolog 1 (Drosophila)	neur-nr2f	191 - 308	(+)
**GXP_1175246** [GXP_1175246] (1 - 601)<br>**Irak1_predicted**, GXL_329031, GeneID: 363520, Rattus norvegicus chr. X<br>interleukin-1 receptor-associated kinase 1 (predicted)	neur-nr2f	249 - 111	(-)
**GXP_1408994** [GXP_1408994] (1 - 602)<br>**Fndc1**, GXL_467842, GeneID: 308099, Rattus norvegicus chr. 1<br>fibronectin type III domain containing 1	neur-nr2f	426 - 547	(+)
**GXP_1409621** [GXP_1409621] (1 - 602)<br>**Nell1**, GXL_467904, GeneID: 81733, Rattus norvegicus chr. 1<br>NEL-like 1 (chicken)	neur-nr2f	256 - 120	(-)
**GXP_850097** [GXP_850097] (1 - 601)<br>**Olr233_predicted**, GXL_467936, GeneID: 293377, Rattus norvegicus chr. 1<br>olfactory receptor 233 (predicted)	neur-nr2f	325 - 173	(-)
**GXP_851427** [GXP_851427] (1 - 601)<br>**Olr1388_predicted**, GXL_468002, GeneID: 405066, Rattus norvegicus chr. 10<br>olfactory receptor 1388 (predicted)	neur-nr2f	106 - 251	(+)
**GXP_852869** [GXP_852869] (1 - 1101)<br>**Trp63**, GXL_468112, GeneID: 246334, Rattus norvegicus chr. 11<br>transformation related protein 63	neur-nr2f	777 - 928	(+)
**GXP_1404310** [GXP_1404310] (1 - 607)<br>**Kiaa0415**, GXL_468145, GeneID: 641386, Rattus norvegicus chr. 12<br>KIAA0415 protein	neur-nr2f	329 - 479	(+)
	neur-nr2f	752 - 635	(-)

**GXP_1404312** [GXP_1404312] (1 - 1111)<br>**Kiaa0415**, GXL_468145, GeneID: 641386, Rattus norvegicus chr. 12<br>KIAA0415 protein			
**GXP_1404867** [GXP_1404867] (1 - 601)<br>**Nfasc**, GXL_468187, GeneID: 116690, Rattus norvegicus chr. 13<br>neurofascin	neur-nr2f	535 - 406	(-)
**GXP_1405357** [GXP_1405357] (1 - 601)<br>**Rab3gap2**, GXL_468217, GeneID: 289350, Rattus norvegicus chr. 13<br>RAB3 GTPase activating protein subunit 2	neur-nr2f	203 - 61	(-)
**GXP_855159** [GXP_855159] (1 - 1068)<br>**Hr**, GXL_468319, GeneID: 60563, Rattus norvegicus chr. 15<br>hairless homolog (mouse)	neur-nr2f	777 - 923	(+)
**GXP_856343** [GXP_856343] (1 - 605)<br>**B4galt6**, GXL_468416, GeneID: 65196, Rattus norvegicus chr. 18<br>UDP-Gal:betaGlcNAc beta 1,4-galactosyltransferase, polypeptide 6	neur-nr2f	329 - 481	(+)
**GXP_856667** [GXP_856667] (1 - 601)<br>**Slc14a2**, GXL_468437, GeneID: 54302, Rattus norvegicus chr. 18<br>solute carrier family 14 (urea transporter), member 2	neur-nr2f	165 - 291	(+)
**GXP_1408700** [GXP_1408700] (1 - 601)<br>**Efcbp2**, GXL_468485, GeneID: 170928, Rattus norvegicus chr. 19<br>EF hand calcium binding protein 2	neur-nr2f	85 - 210	(+)
**GXP_858122** [GXP_858122] (1 - 1206)<br>**Magi3**, GXL_468542, GeneID: 245903, Rattus norvegicus chr. 2<br>membrane associated guanylate kinase, WW and PDZ domain containing 3	neur-nr2f	31 - 177	(+)
**GXP_859449** [GXP_859449] (1 - 601)<br>**Olr681_predicted**, GXL_468659, GeneID: 404824, Rattus norvegicus chr. 3<br>olfactory receptor 681 (predicted)	neur-nr2f	38 - 185	(+)
**GXP_1413488** [GXP_1413488] (1 - 601)<br>**Madd**, GXL_468665, GeneID: 94193, Rattus norvegicus chr. 3<br>MAP-kinase activating death domain	neur-nr2f	161 - 15	(-)
**GXP_862296** [GXP_862296] (1 - 697)<br>**Spata21**, GXL_468836, GeneID: 366491, Rattus norvegicus chr. 5<br>spermatogenesis associated 21	neur-nr2f	381 - 248	(-)
**GXP_1417517** [GXP_1417517] (1 - 602)<br>**Zdhhc22**, GXL_468867, GeneID: 299211, Rattus norvegicus chr. 6<br>zinc finger, DHHC-type containing 22	neur-nr2f	455 - 578	(+)
**GXP_1417816** [GXP_1417816] (1 - 601)<br>**Pias4**, GXL_468901, GeneID: 362827, Rattus norvegicus chr. 7<br>protein inhibitor of activated STAT, 4	neur-nr2f	53 - 205	(+)
	neur-nr2f	174 - 319	(+)

**GXP_865913** [GXP_865913] (1 - 858)<br>**Sag**, GXL_469070, GeneID: 25539, Rattus norvegicus chr. 9<br>retinal S-antigen			
**GXP_1402231** [GXP_1402231] (1 - 1455)<br>**Srrm2_predicted**, GXL_621399, GeneID: 302969, Rattus norvegicus chr. 10<br>serine/arginine repetitive matrix 2 (predicted)	neur-nr2f	905 - 1032	(+)
**GXP_1147398** [GXP_1147398] (1 - 661)<br>**Zfp39_predicted**, GXL_621557, GeneID: 303173, Rattus norvegicus chr. 10<br>zinc finger protein 39 (predicted)	neur-nr2f	399 - 547	(+)
**GXP_1147500** [GXP_1147500] (1 - 1020)<br>**Zfp287_predicted**, GXL_621598, GeneID: 303212, Rattus norvegicus chr. 10<br>zinc finger protein 287 (predicted)	neur-nr2f	464 - 344	(-)
**GXP_1147504** [GXP_1147504] (1 - 853)<br>**Trim16_predicted**, GXL_621600, GeneID: 303214, Rattus norvegicus chr. 10<br>tripartite motif protein 16 (predicted)	neur-nr2f	758 - 622	(-)
**GXP_1402653** [GXP_1402653] (1 - 602)<br>**Arhgef15_predicted**, GXL_621629, GeneID: 287418, Rattus norvegicus chr. 10<br>Rho guanine nucleotide exchange factor (GEF) 15 (predicted)	neur-nr2f	595 - 456	(-)
**GXP_1147661** [GXP_1147661] (1 - 668)<br>**Alox12_predicted**, GXL_621651, GeneID: 287454, Rattus norvegicus chr. 10<br>arachidonate 12-lipoxygenase (predicted)	neur-nr2f	141 - 23	(-)
**GXP_1147665** [GXP_1147665] (1 - 917)<br>**Tm4sf5_predicted**, GXL_621653, GeneID: 303256, Rattus norvegicus chr. 10<br>transmembrane 4 superfamily member 5 (predicted)	neur-nr2f	580 - 733	(+)
**GXP_1402812** [GXP_1402812] (1 - 668)<br>**Rpa1**, GXL_621694, GeneID: 287524, Rattus norvegicus chr. 10<br>replication protein A1	neur-nr2f	275 - 143	(-)
**GXP_1147786** [GXP_1147786] (1 - 709)<br>**Smyd4_predicted**, GXL_621695, GeneID: 287525, Rattus norvegicus chr. 10<br>SET and MYND domain containing 4 (predicted)	neur-nr2f	565 - 697	(+)
**GXP_1147943** [GXP_1147943] (1 - 791)<br>**Psmd11_predicted**, GXL_621742, GeneID: 303353, Rattus norvegicus chr. 10<br>proteasome (prosome, macropain) 26S subunit, non-ATPase, 11 (predicted)	neur-nr2f	496 - 357	(-)
**GXP_1403019** [GXP_1403019] (1 - 602)<br>**Tex14_predicted**, GXL_621783, GeneID: 287603, Rattus norvegicus chr. 10<br>testis expressed gene 14 (predicted)	neur-nr2f	565 - 412	(-)
**GXP_1403022** [GXP_1403022] (1 - 602)<br>**Tex14_predicted**, GXL_621783, GeneID: 287603, Rattus norvegicus chr. 10<br>testis expressed gene 14 (predicted)	neur-nr2f	530 - 395	(-)
	neur-nr2f	380 - 532	(+)

**GXP_1403032** [GXP_1403032] (1 - 602)<br>**Mpo**, GXL_621789, GeneID: 303413, Rattus norvegicus chr. 10<br>myeloperoxidase			
**GXP_1403095** [GXP_1403095] (1 - 601)<br>**Itga3_predicted**, GXL_621819, GeneID: 360606, Rattus norvegicus chr. 10<br>integrin alpha 3 (predicted)	neur-nr2f	161 - 43	(-)
**GXP_1403116** [GXP_1403116] (1 - 601)<br>**Ttll6_predicted**, GXL_621826, GeneID: 287646, Rattus norvegicus chr. 10<br>tubulin tyrosine ligase-like family, member 6 (predicted)	neur-nr2f	189 - 310	(+)
**GXP_1403139** [GXP_1403139] (1 - 712)<br>**Osbpl7_predicted**, GXL_621835, GeneID: 303497, Rattus norvegicus chr. 10<br>oxysterol binding protein-like 7 (predicted)	neur-nr2f	270 - 153	(-)
**GXP_1403198** [GXP_1403198] (1 - 681)<br>**Cdc6_predicted**, GXL_621855, GeneID: 360621, Rattus norvegicus chr. 10<br>cell division cycle 6 homolog (S. cerevisiae) (predicted)	neur-nr2f	434 - 580	(+)
**GXP_1403199** [GXP_1403199] (1 - 602)<br>**Cdc6_predicted**, GXL_621855, GeneID: 360621, Rattus norvegicus chr. 10<br>cell division cycle 6 homolog (S. cerevisiae) (predicted)	neur-nr2f	91 - 237	(+)
**GXP_1403395** [GXP_1403395] (1 - 639)<br>**Helz_predicted**, GXL_621941, GeneID: 287773, Rattus norvegicus chr. 10<br>helicase with zinc finger domain (predicted)	neur-nr2f	264 - 121	(-)
**GXP_1403435** [GXP_1403435] (1 - 627)<br>**Sdk2_predicted**, GXL_621962, GeneID: 360652, Rattus norvegicus chr. 10<br>sidekick homolog 2 (chicken) (predicted)	neur-nr2f	377 - 237	(-)
**GXP_1148637** [GXP_1148637] (1 - 602)<br>**Ush1g_predicted**, GXL_621983, GeneID: 287819, Rattus norvegicus chr. 10<br>Usher syndrome 1G homolog (human) (predicted)	neur-nr2f	355 - 486	(+)
**GXP_1403550** [GXP_1403550] (1 - 601)<br>**Usp36_predicted**, GXL_622018, GeneID: 303700, Rattus norvegicus chr. 10<br>ubiquitin specific protease 36 (predicted)	neur-nr2f	382 - 251	(-)
**GXP_1403678** [GXP_1403678] (1 - 601)<br>**Sfrs15**, GXL_622113, GeneID: 245924, Rattus norvegicus chr. 11<br>splicing factor, arginine/serine-rich 15	neur-nr2f	136 - 10	(-)
**GXP_1403764** [GXP_1403764] (1 - 727)<br>**Prdm15_predicted**, GXL_622141, GeneID: 304054, Rattus norvegicus chr. 11<br>PR domain containing 15 (predicted)	neur-nr2f	238 - 87	(-)
**GXP_1403767** [GXP_1403767] (1 - 602)<br>**Zfp295_predicted**, GXL_622143, GeneID: 304056, Rattus norvegicus chr. 11<br>zinc finger protein 295 (predicted)	neur-nr2f	345 - 483	(+)
	neur-nr2f	104 - 243	(+)

**GXP_1403919** [GXP_1403919] (1 - 607)<br>**Mylk_predicted**, GXL_622247, GeneID: 288057, Rattus norvegicus chr. 11<br>myosin, light polypeptide kinase (predicted)			
**GXP_1149546** [GXP_1149546] (1 - 601)<br>**Vpreb1**, GXL_622337, GeneID: 303782, Rattus norvegicus chr. 11<br>pre-B lymphocyte gene 1	neur-nr2f	199 - 61	(-)
**GXP_1404165** [GXP_1404165] (1 - 602)<br>**Lass4_predicted**, GXL_622360, GeneID: 304208, Rattus norvegicus chr. 12<br>longevity assurance homolog 4 (S. cerevisiae) (predicted)	neur-nr2f	428 - 300	(-)
**GXP_1404179** [GXP_1404179] (1 - 1379)<br>**Pcp2**, GXL_622379, GeneID: 304195, Rattus norvegicus chr. 12<br>Purkinje cell protein 2 (L7)	neur-nr2f	667 - 543	(-)
**GXP_1149855** [GXP_1149855] (1 - 665)<br>**Zfp68_predicted**, GXL_622474, GeneID: 304337, Rattus norvegicus chr. 12<br>zinc finger protein 68 (predicted)	neur-nr2f	379 - 498	(+)
**GXP_1150117** [GXP_1150117] (1 - 1090)<br>**Rimbp2**, GXL_622582, GeneID: 266780, Rattus norvegicus chr. 12<br>RIM binding protein 2	neur-nr2f	387 - 533	(+)
**GXP_1404548** [GXP_1404548] (1 - 601)<br>**Fbxl10**, GXL_622620, GeneID: 304495, Rattus norvegicus chr. 12<br>F-box and leucine-rich repeat protein 10	neur-nr2f	424 - 305	(-)
**GXP_1404552** [GXP_1404552] (1 - 602)<br>**Anapc5_predicted**, GXL_622621, GeneID: 288671, Rattus norvegicus chr. 12<br>anaphase-promoting complex subunit 5 (predicted)	neur-nr2f	141 - 17	(-)
**GXP_1150310** [GXP_1150310] (1 - 726)<br>**Fbxw8_predicted**, GXL_622645, GeneID: 304522, Rattus norvegicus chr. 12<br>F-box and WD-40 domain protein 8 (predicted)	neur-nr2f	590 - 449	(-)
**GXP_1150323** [GXP_1150323] (1 - 601)<br>**Rfc5_predicted**, GXL_622650, GeneID: 304528, Rattus norvegicus chr. 12<br>replication factor C (activator 1) 5 (predicted)	neur-nr2f	32 - 180	(+)
**GXP_1404695** [GXP_1404695] (1 - 601)<br>**Usp30_predicted**, GXL_622671, GeneID: 304579, Rattus norvegicus chr. 12<br>ubiquitin specific protease 30 (predicted)	neur-nr2f	395 - 535	(+)
**GXP_1150622** [GXP_1150622] (1 - 876)<br>**Elk4_predicted**, GXL_622774, GeneID: 304786, Rattus norvegicus chr. 13<br>ELK4, member of ETS oncogene family (predicted)	neur-nr2f	183 - 330	(+)
**GXP_1405068** [GXP_1405068] (1 - 601)<br>**Tdrd5_predicted**, GXL_622875, GeneID: 289129, Rattus norvegicus chr. 13<br>tudor domain containing 5 (predicted)	neur-nr2f	329 - 199	(-)
	neur-nr2f	808 - 663	(-)

**GXP_1151143** [GXP_1151143] (1 - 823)<br>**Slamf8_predicted**, GXL_622971, GeneID: 289237, Rattus norvegicus chr. 13<br>SLAM family member 8 (predicted)			
**GXP_1405815** [GXP_1405815] (1 - 946)<br>**Zfp509_predicted**, GXL_623347, GeneID: 305428, Rattus norvegicus chr. 14<br>zinc finger protein 509 (predicted)	neur-nr2f	764 - 635	(-)
**GXP_1152178** [GXP_1152178] (1 - 601)<br>**Smtn**, GXL_623388, GeneID: 289734, Rattus norvegicus chr. 14<br>smoothelin	neur-nr2f	462 - 340	(-)
**GXP_1405925** [GXP_1405925] (1 - 601)<br>**Smtn**, GXL_623388, GeneID: 289734, Rattus norvegicus chr. 14<br>smoothelin	neur-nr2f	369 - 223	(-)
**GXP_1152317** [GXP_1152317] (1 - 1111)<br>**Spred2**, GXL_623442, GeneID: 305539, Rattus norvegicus chr. 14<br>sprouty-related, EVH1 domain containing 2	neur-nr2f	13 - 144	(+)
**GXP_1406113** [GXP_1406113] (1 - 601)<br>**Psme4**, GXL_623488, GeneID: 498433, Rattus norvegicus chr. 14<br>proteasome (prosome, macropain) activator subunit 4	neur-nr2f	388 - 261	(-)
**GXP_1406114** [GXP_1406114] (1 - 601)<br>**Psme4**, GXL_623488, GeneID: 498433, Rattus norvegicus chr. 14<br>proteasome (prosome, macropain) activator subunit 4	neur-nr2f	385 - 258	(-)
**GXP_1406147** [GXP_1406147] (1 - 601)<br>**Ndst2_predicted**, GXL_623497, GeneID: 114002, Rattus norvegicus chr. 15<br>N-deacetylase/N-sulfotransferase (heparan glucosaminyl) 2 (predicted)	neur-nr2f	245 - 395	(+)
**GXP_1406339** [GXP_1406339] (1 - 850)<br>**Pck2_predicted**, GXL_623657, GeneID: 361042, Rattus norvegicus chr. 15<br>phosphoenolpyruvate carboxykinase 2 (mitochondrial) (predicted)	neur-nr2f	646 - 496	(-)
**GXP_1152860** [GXP_1152860] (1 - 601)<br>**Mcpt4l1**, GXL_623662, GeneID: 408208, Rattus norvegicus chr. 15<br>mast cell protease 4-like 1	neur-nr2f	96 - 230	(+)
**GXP_1406376** [GXP_1406376] (1 - 655)<br>**Adprtl1**, GXL_623681, GeneID: 361046, Rattus norvegicus chr. 15<br>ADP-ribosyltransferase (NAD+, poly (ADP-ribose) polymerase)-like 1	neur-nr2f	425 - 272	(-)
**GXP_1152997** [GXP_1152997] (1 - 704)<br>**Pbk_predicted**, GXL_623725, GeneID: 290326, Rattus norvegicus chr. 15<br>PDZ binding kinase (predicted)	neur-nr2f	361 - 491	(+)
**GXP_1406666** [GXP_1406666] (1 - 603)<br>**Dock9**, GXL_623860, GeneID: 259237, Rattus norvegicus chr. 15<br>dedicator of cytokinesis 9	neur-nr2f	105 - 232	(+)
	neur-nr2f	128 - 250	(+)

GXP_1406698 [GXP_1406698] (1 - 601)
Slmap_predicted, GXL_623881, GeneID: 290533, Rattus norvegicus chr. 16
sarcolemma associated protein (predicted)

GXP_1153440 [GXP_1153440] (1 - 668)
Sema3g, GXL_623906, GeneID: 290562, Rattus norvegicus chr. 16 — neur-nr2f — 630 - 506 — (-)
sema domain, immunoglobulin domain (Ig), short basic domain, secreted, (semaphorin) 3G

GXP_1153489 [GXP_1153489] (1 - 601)
Zfp488_predicted, GXL_623925, GeneID: 290571, Rattus norvegicus chr. 16 — neur-nr2f — 456 - 333 — (-)
zinc finger protein 488 (predicted)

GXP_1153557 [GXP_1153557] (1 - 884)
Sh2d4b_predicted, GXL_623966, GeneID: 290612, Rattus norvegicus chr. 16 — neur-nr2f — 381 - 256 — (-)
SH2 domain containing 4B (predicted)

GXP_1406841 [GXP_1406841] (1 - 602)
Ap1m1, GXL_623970, GeneID: 306332, Rattus norvegicus chr. 16 — neur-nr2f — 438 - 295 — (-)
adaptor-related protein complex AP-1, mu subunit 1

GXP_1153905 [GXP_1153905] (1 - 602)
F11, GXL_624109, GeneID: 290757, Rattus norvegicus chr. 16 — neur-nr2f — 425 - 566 — (+)
coagulation factor XI

GXP_1407037 [GXP_1407037] (1 - 601)
Sgcz_predicted, GXL_624130, GeneID: 364605, Rattus norvegicus chr. 16 — neur-nr2f — 419 - 299 — (-)
sarcoglycan zeta (predicted)

GXP_1154071 [GXP_1154071] (1 - 616)
Myst3, GXL_624182, GeneID: 306571, Rattus norvegicus chr. 16 — neur-nr2f — 201 - 332 — (+)
MYST histone acetyltransferase (monocytic leukemia) 3

GXP_1407119 [GXP_1407119] (1 - 687)
Thsd1_predicted, GXL_624187, GeneID: 364630, Rattus norvegicus chr. 16 — neur-nr2f — 44 - 191 — (+)
thrombospondin, type I, domain 1 (predicted)

GXP_1154097 [GXP_1154097] (1 - 601)
RatNP-3b, GXL_624193, GeneID: 498659, Rattus norvegicus chr. 16 — neur-nr2f — 314 - 175 — (-)
defensin RatNP-3 precursor

GXP_1154299 [GXP_1154299] (1 - 601)
Trpc7, GXL_624287, GeneID: 282822, Rattus norvegicus chr. 17 — neur-nr2f — 212 - 66 — (-)
transient receptor potential cation channel, subfamily C, member 7

GXP_1407296 [GXP_1407296] (1 - 601)
Dok3_predicted, GXL_624300, GeneID: 306760, Rattus norvegicus chr. 17 — neur-nr2f — 200 - 68 — (-)
docking protein 3 (predicted)

GXP_1154452 [GXP_1154452] (1 - 601)
Zfp169_predicted, GXL_624351, GeneID: 306816, Rattus norvegicus chr. 17 — neur-nr2f — 105 - 238 — (+)
zinc finger protein 169 (predicted)
neur-nr2f — 435 - 555 — (+)

**GXP_1407483** [GXP_1407483] (1 - 601)<br>**Lrrc16_predicted**, GXL_624439, GeneID: 306941, Rattus norvegicus chr. 17<br>leucine rich repeat containing 16 (predicted)			
**GXP_1407509** [GXP_1407509] (1 - 603)<br>**Gli3**, GXL_624494, GeneID: 140588, Rattus norvegicus chr. 17<br>GLI-Kruppel family member GLI3	neur-nr2f	218 - 343	(+)
**GXP_1407640** [GXP_1407640] (1 - 601)<br>**Usp6nl_predicted**, GXL_624591, GeneID: 291309, Rattus norvegicus chr. 17<br>USP6 N-terminal like (predicted)	neur-nr2f	157 - 32	(-)
**GXP_1407641** [GXP_1407641] (1 - 1018)<br>**Upf2_predicted**, GXL_624592, GeneID: 361271, Rattus norvegicus chr. 17<br>UPF2 regulator of nonsense transcripts homolog (yeast) (predicted)	neur-nr2f	291 - 411	(+)
**GXP_1155048** [GXP_1155048] (1 - 601)<br>**Gpr158_predicted**, GXL_624644, GeneID: 291352, Rattus norvegicus chr. 17<br>G protein-coupled receptor 158 (predicted)	neur-nr2f	419 - 302	(-)
**GXP_1155148** [GXP_1155148] (1 - 601)<br>**Dsc1_predicted**, GXL_624692, GeneID: 291759, Rattus norvegicus chr. 18<br>desmocollin 1 (predicted)	neur-nr2f	210 - 338	(+)
**GXP_1155345** [GXP_1155345] (1 - 607)<br>**Pcdhb18**, GXL_624773, GeneID: 548105, Rattus norvegicus chr. 18<br>protocadherin beta 18	neur-nr2f	429 - 294	(-)
**GXP_1407914** [GXP_1407914] (1 - 602)<br>**Arhgap26_predicted**, GXL_624790, GeneID: 307459, Rattus norvegicus chr. 18<br>Rho GTPase activating protein 26 (predicted)	neur-nr2f	130 - 270	(+)
**GXP_1155429** [GXP_1155429] (1 - 601)<br>**Dcp2_predicted**, GXL_624809, GeneID: 291604, Rattus norvegicus chr. 18<br>DCP2 decapping enzyme homolog (S. cerevisiae) (predicted)	neur-nr2f	448 - 330	(-)
**GXP_1155474** [GXP_1155474] (1 - 601)<br>**Tnfaip8_predicted**, GXL_624839, GeneID: 307428, Rattus norvegicus chr. 18<br>tumor necrosis factor, alpha-induced protein 8 (predicted)	neur-nr2f	445 - 571	(+)
**GXP_1407972** [GXP_1407972] (1 - 602)<br>**Sncaip_predicted**, GXL_624846, GeneID: 307309, Rattus norvegicus chr. 18<br>synuclein, alpha interacting protein (synphilin) (predicted)	neur-nr2f	117 - 2	(-)
**GXP_1155633** [GXP_1155633] (1 - 897)<br>**Spire1_predicted**, GXL_624909, GeneID: 307348, Rattus norvegicus chr. 18<br>spire homolog 1 (Drosophila) (predicted)	neur-nr2f	119 - 2	(-)
**GXP_1155821** [GXP_1155821] (1 - 601)<br>**Cd226_predicted**, GXL_624983, GeneID: 307199, Rattus norvegicus chr. 18<br>CD226 antigen (predicted)	neur-nr2f	321 - 202	(-)
	neur-nr2f	285 - 133	(-)

**GXP_1408337** [GXP_1408337] (1 - 605)			
**Nkd1_predicted**, GXL_625056, GeneID: 364952, Rattus norvegicus chr. 19			
naked cuticle 1 homolog (Drosophila) (predicted)			
**GXP_1156023** [GXP_1156023] (1 - 1072)			
**Brd7_predicted**, GXL_625057, GeneID: 361374, Rattus norvegicus chr. 19	neur-nr2f	507 - 375	(-)
bromodomain containing 7 (predicted)			
**GXP_1408393** [GXP_1408393] (1 - 601)			
**Tnpo2_predicted**, GXL_625105, GeneID: 304670, Rattus norvegicus chr. 19	neur-nr2f	131 - 254	(+)
transportin 2 (importin 3, karyopherin beta 2b) (predicted)			
**GXP_1408424** [GXP_1408424] (1 - 602)			
**Zswim4_predicted**, GXL_625113, GeneID: 304655, Rattus norvegicus chr. 19	neur-nr2f	12 - 154	(+)
zinc finger, SWIM domain containing 4 (predicted)			
**GXP_1408559** [GXP_1408559] (1 - 603)			
**Rbm35b_predicted**, GXL_625172, GeneID: 307810, Rattus norvegicus chr. 19	neur-nr2f	502 - 356	(-)
RNA binding motif protein 35b (predicted)			
**GXP_1408716** [GXP_1408716] (1 - 618)			
**Cotl1_predicted**, GXL_625234, GeneID: 361422, Rattus norvegicus chr. 19	neur-nr2f	392 - 521	(+)
coactosin-like 1 (Dictyostelium) (predicted)			
**GXP_1408741** [GXP_1408741] (1 - 602)			
**Snai3_predicted**, GXL_625250, GeneID: 307919, Rattus norvegicus chr. 19	neur-nr2f	374 - 514	(+)
snail homolog 3 (Drosophila) (predicted)			
**GXP_1156599** [GXP_1156599] (1 - 601)			
**Sult5a1_predicted**, GXL_625259, GeneID: 292077, Rattus norvegicus chr. 19	neur-nr2f	308 - 162	(-)
sulfotransferase family 5A, member 1 (predicted)			
**GXP_1156626** [GXP_1156626] (1 - 601)			
**Galnt2_predicted**, GXL_625271, GeneID: 292090, Rattus norvegicus chr. 19	neur-nr2f	33 - 185	(+)
UDP-N-acetyl-alpha-D-galactosamine:polypeptide N-acetylgalactosaminyltransferase 2 (predicted)			
**GXP_1408784** [GXP_1408784] (1 - 601)			
**Pgbd5_predicted**, GXL_625272, GeneID: 292098, Rattus norvegicus chr. 19	neur-nr2f	335 - 480	(+)
piggyBac transposable element derived 5 (predicted)			
**GXP_1408926** [GXP_1408926] (1 - 601)			
**Slc12a7**, GXL_625399, GeneID: 308069, Rattus norvegicus chr. 1	neur-nr2f	74 - 215	(+)
solute carrier family 12 (potassium/chloride transporters), member 7			
**GXP_1409018** [GXP_1409018] (1 - 601)			
**Rnaset2_predicted**, GXL_625455, GeneID: 292306, Rattus norvegicus chr. 1	neur-nr2f	9 - 130	(+)
ribonuclease T2 (predicted)			
**GXP_1157118** [GXP_1157118] (1 - 680)			
**Wdr27_predicted**, GXL_625523, GeneID: 308222, Rattus norvegicus chr. 1	neur-nr2f	409 - 260	(-)
WD repeat domain 27 (predicted)			
	neur-nr2f	105 - 251	(+)

**GXP_1409107** [GXP_1409107] (1 - 633)<br>**Ptprh**, GXL_625649, GeneID: 171125, Rattus norvegicus chr. 1<br>protein tyrosine phosphatase, receptor type, H			
**GXP_1157805** [GXP_1157805] (1 - 601)<br>**Etsrp71_predicted**, GXL_625853, GeneID: 361544, Rattus norvegicus chr. 1<br>ets related protein 71 (predicted)	neur-nr2f	456 - 589	(+)
**GXP_1409417** [GXP_1409417] (1 - 601)<br>**Ankrd27_predicted**, GXL_625880, GeneID: 361555, Rattus norvegicus chr. 1<br>ankyrin repeat domain 27 (VPS9 domain) (predicted)	neur-nr2f	506 - 360	(-)
**GXP_1157955** [GXP_1157955] (1 - 601)<br>**Klk5**, GXL_625921, GeneID: 408211, Rattus norvegicus chr. 1<br>kallikrein 5	neur-nr2f	425 - 289	(-)
**GXP_1409520** [GXP_1409520] (1 - 634)<br>**Trpm4**, GXL_625939, GeneID: 171143, Rattus norvegicus chr. 1<br>transient receptor potential cation channel, subfamily M, member 4	neur-nr2f	385 - 538	(+)
**GXP_1409526** [GXP_1409526] (1 - 601)<br>**Ppfia3**, GXL_625940, GeneID: 140591, Rattus norvegicus chr. 1<br>protein tyrosine phosphatase, receptor type, f polypeptide (PTPRF), interacting protein (liprin), alpha 3	neur-nr2f	574 - 443	(-)
**GXP_1409595** [GXP_1409595] (1 - 621)<br>**Tph1**, GXL_625955, GeneID: 24848, Rattus norvegicus chr. 1<br>tryptophan hydroxylase 1	neur-nr2f	376 - 513	(+)
**GXP_1409630** [GXP_1409630] (1 - 602)<br>**Tubgcp5_predicted**, GXL_625983, GeneID: 308663, Rattus norvegicus chr. 1<br>tubulin, gamma complex associated protein 5 (predicted)	neur-nr2f	335 - 459	(+)
**GXP_1158193** [GXP_1158193] (1 - 601)<br>**Mkrn3_predicted**, GXL_625996, GeneID: 292988, Rattus norvegicus chr. 1<br>makorin, ring finger protein, 3 (predicted)	neur-nr2f	523 - 371	(-)
**GXP_1409783** [GXP_1409783] (1 - 601)<br>**Nmb_predicted**, GXL_626062, GeneID: 308751, Rattus norvegicus chr. 1<br>neuromedin B (predicted)	neur-nr2f	377 - 525	(+)
**GXP_1409824** [GXP_1409824] (1 - 948)<br>**Eed_predicted**, GXL_626092, GeneID: 293104, Rattus norvegicus chr. 1<br>embryonic ectoderm development (predicted)	neur-nr2f	744 - 618	(-)
**GXP_1409937** [GXP_1409937] (1 - 619)<br>**Centd2**, GXL_626139, GeneID: 361617, Rattus norvegicus chr. 1<br>centaurin, delta 2	neur-nr2f	167 - 282	(+)
**GXP_1158688** [GXP_1158688] (1 - 629)<br>**Nalp10_predicted**, GXL_626184, GeneID: 293426, Rattus norvegicus chr. 1<br>NACHT, leucine rich repeat and PYD containing 10 (predicted)	neur-nr2f	232 - 364	(+)
	neur-nr2f	223 - 108	(-)

**GXP_1158698** [GXP_1158698] (1 - 602)<br>**St5_predicted**, GXL_626189, GeneID: 308944, Rattus norvegicus chr. 1<br>suppression of tumorigenicity 5 (predicted)			
**GXP_1410050** [GXP_1410050] (1 - 754)<br>**Mrvi1_predicted**, GXL_626198, GeneID: 308899, Rattus norvegicus chr. 1<br>MRV integration site 1 homolog (mouse) (predicted)	neur-nr2f	97 - 245	(+)
**GXP_1410222** [GXP_1410222] (1 - 601)<br>**Gdpd3_predicted**, GXL_626271, GeneID: 293490, Rattus norvegicus chr. 1<br>glycerophosphodiester phosphodiesterase domain containing 3 (predicted)	neur-nr2f	552 - 408	(-)
**GXP_1158984** [GXP_1158984] (1 - 601)<br>**Sephs2**, GXL_626279, GeneID: 308993, Rattus norvegicus chr. 1<br>selenophosphate synthetase 2	neur-nr2f	413 - 294	(-)
**GXP_1410449** [GXP_1410449] (1 - 619)<br>**Th**, GXL_626397, GeneID: 25085, Rattus norvegicus chr. 1<br>tyrosine hydroxylase	neur-nr2f	71 - 185	(+)
**GXP_1410557** [GXP_1410557] (1 - 601)<br>**Sf3b2_predicted**, GXL_626437, GeneID: 293671, Rattus norvegicus chr. 1<br>splicing factor 3b, subunit 2 (predicted)	neur-nr2f	207 - 67	(-)
**GXP_1159619** [GXP_1159619] (1 - 824)<br>**Vwce_predicted**, GXL_626496, GeneID: 309209, Rattus norvegicus chr. 1<br>von Willebrand factor C and EGF domains (predicted)	neur-nr2f	82 - 203	(+)
**GXP_1159640** [GXP_1159640] (1 - 601)<br>**Ms4a12_predicted**, GXL_626504, GeneID: 293741, Rattus norvegicus chr. 1<br>membrane-spanning 4-domains, subfamily A, member 12 (predicted)	neur-nr2f	326 - 449	(+)
**GXP_1410730** [GXP_1410730] (1 - 602)<br>**Dtx4**, GXL_626518, GeneID: 293774, Rattus norvegicus chr. 1<br>deltex 4 homolog (Drosophila)	neur-nr2f	158 - 276	(+)
**GXP_1410753** [GXP_1410753] (1 - 602)<br>**Trpm6**, GXL_626536, GeneID: 293874, Rattus norvegicus chr. 1<br>transient receptor potential cation channel, subfamily M, member 6	neur-nr2f	201 - 79	(-)
**GXP_1410767** [GXP_1410767] (1 - 632)<br>**Tmem2_predicted**, GXL_626545, GeneID: 309400, Rattus norvegicus chr. 1<br>transmembrane protein 2 (predicted)	neur-nr2f	68 - 187	(+)
**GXP_1410886** [GXP_1410886] (1 - 602)<br>**Tnks2_predicted**, GXL_626601, GeneID: 309512, Rattus norvegicus chr. 1<br>tankyrase, TRF1-interacting ankyrin-related ADP-ribose polymerase 2 (predicted)	neur-nr2f	566 - 413	(-)
**GXP_1159919** [GXP_1159919] (1 - 601)<br>**Cyp2c6**, GXL_626619, GeneID: 246070, Rattus norvegicus chr. 1<br>Cytochrome P450, subfamily IIC6	neur-nr2f	152 - 7	(-)
	neur-nr2f	25 - 150	(+)

GXP_1410968 [GXP_1410968] (1 - 602)
Wnt8b_predicted, GXL_626648, GeneID: 293990, Rattus norvegicus chr. 1
wingless related MMTV integration site 8b (predicted)

GXP_1161060 [GXP_1161060] (1 - 641)
Ptcd2_predicted, GXL_627077, GeneID: 310025, Rattus norvegicus chr. 2 — neur-nr2f 98 - 243 (+)
pentatricopeptide repeat domain 2 (predicted)

GXP_1411990 [GXP_1411990] (1 - 602)
Hps3_predicted, GXL_627266, GeneID: 310288, Rattus norvegicus chr. 2 — neur-nr2f 274 - 408 (+)
Hermansky-Pudlak syndrome 3 homolog (human) (predicted)

GXP_1412065 [GXP_1412065] (1 - 601)
Dcun1d1_predicted, GXL_627310, GeneID: 310324, Rattus norvegicus chr. 2 — neur-nr2f 149 - 266 (+)
DCN1, defective in cullin neddylation 1, domain containing 1 (S. cerevisiae) (predicted)

GXP_1161619 [GXP_1161619] (1 - 720)
Ccna2, GXL_627314, GeneID: 114494, Rattus norvegicus chr. 2 — neur-nr2f 280 - 433 (+)
cyclin A2

GXP_1412157 [GXP_1412157] (1 - 707)
Mbnl1, GXL_627393, GeneID: 282635, Rattus norvegicus chr. 2 — neur-nr2f 588 - 463 (-)
muscleblind-like 1 (Drosophila)

GXP_1162115 [GXP_1162115] (1 - 601)
Flg, GXL_627534, GeneID: 24641, Rattus norvegicus chr. 2 — neur-nr2f 207 - 65 (-)
filaggrin

GXP_1412499 [GXP_1412499] (1 - 601)
Igsf3_predicted, GXL_627624, GeneID: 295325, Rattus norvegicus chr. 2 — neur-nr2f 470 - 347 (-)
immunoglobulin superfamily, member 3 (predicted)

GXP_1412693 [GXP_1412693] (1 - 726)
Bcar3_predicted, GXL_627714, GeneID: 310838, Rattus norvegicus chr. 2 — neur-nr2f 97 - 240 (+)
breast cancer anti-estrogen resistance 3 (predicted)

GXP_1412694 [GXP_1412694] (1 - 602)
Bcar3_predicted, GXL_627714, GeneID: 310838, Rattus norvegicus chr. 2 — neur-nr2f 254 - 137 (-)
breast cancer anti-estrogen resistance 3 (predicted)

GXP_1412695 [GXP_1412695] (1 - 601)
Bcar3_predicted, GXL_627714, GeneID: 310838, Rattus norvegicus chr. 2 — neur-nr2f 408 - 523 (+)
breast cancer anti-estrogen resistance 3 (predicted)

GXP_1412763 [GXP_1412763] (1 - 602)
Bdh2_predicted, GXL_627763, GeneID: 295458, Rattus norvegicus chr. 2 — neur-nr2f 276 - 153 (-)
3-hydroxybutyrate dehydrogenase, type 2 (predicted)

GXP_1162729 [GXP_1162729] (1 - 833)
Clca2_predicted, GXL_627795, GeneID: 308016, Rattus norvegicus chr. 2 — neur-nr2f 650 - 771 (+)
chloride channel, calcium activated, family member 2 (predicted)

neur-nr2f 150 - 268 (+)

**GXP_1412988** [GXP_1412988] (1 - 611)<br>**Brd3_predicted**, GXL_627914, GeneID: 362092, Rattus norvegicus chr. 3<br>bromodomain containing 3 (predicted)			
**GXP_1163046** [GXP_1163046] (1 - 1036)	neur-nr2f	240 - 115	(-)
**Ntng2_predicted**, GXL_627926, GeneID: 311836, Rattus norvegicus chr. 3<br>netrin G2 (predicted)	neur-nr2f	752 - 889	(+)
**GXP_1163090** [GXP_1163090] (1 - 808)<br>**Ppp2r4_predicted**, GXL_627937, GeneID: 362102, Rattus norvegicus chr. 3<br>protein phosphatase 2A, regulatory subunit B (PR 53) (predicted)	neur-nr2f	768 - 649	(-)
**GXP_1413094** [GXP_1413094] (1 - 602)<br>**Ciz1_predicted**, GXL_627953, GeneID: 296639, Rattus norvegicus chr. 3<br>CDKN1A interacting zinc finger protein 1 (predicted)	neur-nr2f	295 - 167	(-)
**GXP_1163546** [GXP_1163546] (1 - 848)<br>**Hoxd4_predicted**, GXL_628106, GeneID: 288153, Rattus norvegicus chr. 3<br>homeo box D4 (predicted)	neur-nr2f	251 - 382	(+)
**GXP_1413587** [GXP_1413587] (1 - 620)<br>**Ehf_predicted**, GXL_628201, GeneID: 295965, Rattus norvegicus chr. 3<br>ets homologous factor (predicted)	neur-nr2f	372 - 517	(+)
**GXP_1413703** [GXP_1413703] (1 - 601)<br>**Pak6_predicted**, GXL_628262, GeneID: 296078, Rattus norvegicus chr. 3<br>p21 (CDKN1A)-activated kinase 6 (predicted)	neur-nr2f	122 - 5	(-)
**GXP_1413742** [GXP_1413742] (1 - 601)<br>**Pla2g4b_predicted**, GXL_628283, GeneID: 311341, Rattus norvegicus chr. 3<br>phospholipase A2, group IVB (cytosolic) (predicted)	neur-nr2f	347 - 211	(-)
**GXP_1164054** [GXP_1164054] (1 - 601)<br>**Snap23**, GXL_628290, GeneID: 64630, Rattus norvegicus chr. 3<br>synaptosomal-associated protein 23	neur-nr2f	75 - 210	(+)
**GXP_1164072** [GXP_1164072] (1 - 761)<br>**Epb4.2_predicted**, GXL_628296, GeneID: 362202, Rattus norvegicus chr. 3<br>erythrocyte protein band 4.2 (predicted)	neur-nr2f	534 - 382	(-)
**GXP_1414158** [GXP_1414158] (1 - 603)<br>**Myh7b_predicted**, GXL_628500, GeneID: 311570, Rattus norvegicus chr. 3<br>myosin, heavy polypeptide 7B, cardiac muscle, beta (predicted)	neur-nr2f	378 - 500	(+)
**GXP_1164850** [GXP_1164850] (1 - 678)<br>**Zfp217_predicted**, GXL_628580, GeneID: 311764, Rattus norvegicus chr. 3<br>zinc finger protein 217 (predicted)	neur-nr2f	87 - 207	(+)
**GXP_1164933** [GXP_1164933] (1 - 976)<br>**Datf1_predicted**, GXL_628613, GeneID: 362286, Rattus norvegicus chr. 3<br>death associated transcription factor 1 (predicted)	neur-nr2f	880 - 755	(-)
	neur-nr2f	56 - 193	(+)

**GXP_1414372** [GXP_1414372] (1 - 608)<br>**Datf1_predicted**, GXL_628613, GeneID: 362286, Rattus norvegicus chr. 3<br>death associated transcription factor 1 (predicted)			
**GXP_1414612** [GXP_1414612] (1 - 602)<br>**Mdfic_predicted**, GXL_628751, GeneID: 362325, Rattus norvegicus chr. 4<br>MyoD family inhibitor domain containing (predicted)	neur-nr2f	83 - 234	(+)
**GXP_1165594** [GXP_1165594] (1 - 601)<br>**Igf2bp3**, GXL_628866, GeneID: 312320, Rattus norvegicus chr. 4<br>insulin-like growth factor 2, binding protein 3	neur-nr2f	275 - 142	(-)
**GXP_1414862** [GXP_1414862] (1 - 737)<br>**Mpp6_predicted**, GXL_628871, GeneID: 362359, Rattus norvegicus chr. 4<br>membrane protein, palmitoylated 6 (MAGUK p55 subfamily member 6) (predicted)	neur-nr2f	54 - 169	(+)
**GXP_1165911** [GXP_1165911] (1 - 672)<br>**Smyd5_predicted**, GXL_629012, GeneID: 312503, Rattus norvegicus chr. 4<br>SET and MYND domain containing 5 (predicted)	neur-nr2f	141 - 25	(-)
**GXP_1415041** [GXP_1415041] (1 - 602)<br>**Alms1_predicted**, GXL_629014, GeneID: 297408, Rattus norvegicus chr. 4<br>Alstrom syndrome 1 (predicted)	neur-nr2f	72 - 219	(+)
**GXP_1165919** [GXP_1165919] (1 - 601)<br>**Cml5**, GXL_629018, GeneID: 114020, Rattus norvegicus chr. 4<br>camello-like 5	neur-nr2f	176 - 298	(+)
**GXP_1165980** [GXP_1165980] (1 - 703)<br>**Mcm2_predicted**, GXL_629040, GeneID: 312538, Rattus norvegicus chr. 4<br>minichromosome maintenance deficient 2 mitotin (S. cerevisiae) (predicted)	neur-nr2f	8 - 132	(+)
**GXP_1415083** [GXP_1415083] (1 - 601)<br>**Mcm2_predicted**, GXL_629040, GeneID: 312538, Rattus norvegicus chr. 4<br>minichromosome maintenance deficient 2 mitotin (S. cerevisiae) (predicted)	neur-nr2f	150 - 285	(+)
**GXP_1415201** [GXP_1415201] (1 - 603)<br>**Il17rc_predicted**, GXL_629100, GeneID: 297520, Rattus norvegicus chr. 4<br>interleukin 17 receptor C (predicted)	neur-nr2f	164 - 317	(+)
**GXP_1415248** [GXP_1415248] (1 - 683)<br>**Plxnd1_predicted**, GXL_629115, GeneID: 312652, Rattus norvegicus chr. 4<br>plexin D1 (predicted)	neur-nr2f	326 - 475	(+)
	neur-nr2f	422 - 539	(+)
**GXP_1166224** [GXP_1166224] (1 - 601)<br>**Rasgef1a_predicted**, GXL_629127, GeneID: 312664, Rattus norvegicus chr. 4<br>RasGEF domain family, member 1A (predicted)	neur-nr2f	383 - 244	(-)
**GXP_1166692** [GXP_1166692] (1 - 601)<br>**Rrs1_predicted**, GXL_629330, GeneID: 297784, Rattus norvegicus chr. 5<br>RRS1 ribosome biogenesis regulator homolog (S. cerevisiae) (predicted)	neur-nr2f	391 - 539	(+)
	neur-nr2f	437 - 321	(-)

**GXP_1167096** [GXP_1167096] (1 - 666)<br>**Galnt12_predicted**, GXL_629493, GeneID: 313233, Rattus norvegicus chr. 5<br>UDP-N-acetyl-alpha-D-galactosamine:polypeptide N-acetylgalactosaminyltransferase 12 (predicted)			
**GXP_1415900** [GXP_1415900] (1 - 602)<br>**Rnf20_predicted**, GXL_629499, GeneID: 313216, Rattus norvegicus chr. 5<br>ring finger protein 20 (predicted)	neur-nr2f	235 - 387	(+)
**GXP_1167187** [GXP_1167187] (1 - 629)<br>**Susd1_predicted**, GXL_629528, GeneID: 298031, Rattus norvegicus chr. 5<br>sushi domain containing 1 (predicted)	neur-nr2f	235 - 85	(-)
**GXP_1416076** [GXP_1416076] (1 - 602)<br>**Mtap_predicted**, GXL_629607, GeneID: 298227, Rattus norvegicus chr. 5<br>methylthioadenosine phosphorylase (predicted)	neur-nr2f	217 - 84	(-)
**GXP_1416089** [GXP_1416089] (1 - 659)<br>**Mysm1_predicted**, GXL_629626, GeneID: 298247, Rattus norvegicus chr. 5<br>myb-like, SWIRM and MPN domains 1 (predicted)	neur-nr2f	326 - 448	(+)
**GXP_1416231** [GXP_1416231] (1 - 610)<br>**Stil_predicted**, GXL_629710, GeneID: 313506, Rattus norvegicus chr. 5<br>Scl/Tal1 interrupting locus (predicted)	neur-nr2f	399 - 539	(+)
**GXP_1416251** [GXP_1416251] (1 - 605)<br>**Ipp_predicted**, GXL_629721, GeneID: 298439, Rattus norvegicus chr. 5<br>IAP promoted placental gene (predicted)	neur-nr2f	518 - 389	(-)
**GXP_1416436** [GXP_1416436] (1 - 826)<br>**Eif2c4_predicted**, GXL_629794, GeneID: 298533, Rattus norvegicus chr. 5<br>eukaryotic translation initiation factor 2C, 4 (predicted)	neur-nr2f	520 - 644	(+)
**GXP_1167909** [GXP_1167909] (1 - 612)<br>**Sync_predicted**, GXL_629815, GeneID: 362606, Rattus norvegicus chr. 5<br>syncoilin (predicted)	neur-nr2f	193 - 67	(-)
**GXP_1167915** [GXP_1167915] (1 - 734)<br>**Zbtb8_predicted**, GXL_629818, GeneID: 313049, Rattus norvegicus chr. 5<br>zinc finger and BTB domain containing 8 (predicted)	neur-nr2f	596 - 721	(+)
**GXP_1168036** [GXP_1168036] (1 - 601)<br>**Extl1_predicted**, GXL_629857, GeneID: 313610, Rattus norvegicus chr. 5<br>exostoses (multiple)-like 1 (predicted)	neur-nr2f	340 - 215	(-)
**GXP_1168093** [GXP_1168093] (1 - 602)<br>**Epha8**, GXL_629875, GeneID: 60589, Rattus norvegicus chr. 5<br>Eph receptor A8	neur-nr2f	323 - 170	(-)
**GXP_1416620** [GXP_1416620] (1 - 601)<br>**Eif4g3_predicted**, GXL_629882, GeneID: 298573, Rattus norvegicus chr. 5<br>eukaryotic translation initiation factor 4 gamma, 3 (predicted)	neur-nr2f	159 - 11	(-)
	neur-nr2f	71 - 222	(+)

GXP_1168184 [GXP_1168184] (1 - 602)
Crocc_predicted, GXL_629906, GeneID: 313663, Rattus norvegicus chr. 5
ciliary rootlet coiled-coil, rootletin (predicted)

GXP_1168285 [GXP_1168285] (1 - 601)
Mthfr_predicted, GXL_629949, GeneID: 362657, Rattus norvegicus chr. 5 — neur-nr2f 178 - 304 (+)
5, 10-methylenetetrahydrofolate reductase (NADPH) (predicted)

GXP_1416860 [GXP_1416860] (1 - 797)
Centb5_predicted, GXL_629998, GeneID: 313772, Rattus norvegicus chr. 5 — neur-nr2f 553 - 427 (-)
centaurin, beta 5 (predicted)

GXP_1417045 [GXP_1417045] (1 - 603)
Otof, GXL_630102, GeneID: 84573, Rattus norvegicus chr. 6 — neur-nr2f 480 - 334 (-)
otoferlin

GXP_1168790 [GXP_1168790] (1 - 601)
Ddef2_predicted, GXL_630143, GeneID: 362719, Rattus norvegicus chr. 6 — neur-nr2f 210 - 360 (+)
development and differentiation enhancing factor 2 (predicted)

GXP_1417126 [GXP_1417126] (1 - 602)
Grhl1_predicted, GXL_630145, GeneID: 313993, Rattus norvegicus chr. 6 — neur-nr2f 239 - 375 (+)
grainyhead-like 1 (Drosophila) (predicted)

GXP_1168974 [GXP_1168974] (1 - 601)
Nkx2-9_predicted, GXL_630219, GeneID: 299061, Rattus norvegicus chr. 6 — neur-nr2f 576 - 448 (-)
NK2 transcription factor related, locus 9 (Drosophila) (predicted)

GXP_1168989 [GXP_1168989] (1 - 620)
Mgea6_predicted, GXL_630228, GeneID: 299078, Rattus norvegicus chr. 6 — neur-nr2f 467 - 594 (+)
meningioma expressed antigen 6 (coiled-coil proline-rich) (predicted)

GXP_1417417 [GXP_1417417] (1 - 601)
Zfyve26_predicted, GXL_630308, GeneID: 314265, Rattus norvegicus chr. 6 — neur-nr2f 451 - 586 (+)
zinc finger, FYVE domain containing 26 (predicted)

GXP_1417554 [GXP_1417554] (1 - 631)
Ches1_predicted, GXL_630422, GeneID: 314374, Rattus norvegicus chr. 6 — neur-nr2f 402 - 541 (+)
checkpoint suppressor 1 (predicted)

GXP_1417612 [GXP_1417612] (1 - 601)
Serpina9_predicted, GXL_630453, GeneID: 299274, Rattus norvegicus chr. 6 — neur-nr2f 376 - 523 (+)
serine (or cysteine) peptidase inhibitor, clade A (alpha-1 antiproteinase, antitrypsin), member 9 (predicted)

GXP_1169512 [GXP_1169512] (1 - 633)
Dicer1, GXL_630463, GeneID: 299284, Rattus norvegicus chr. 6 — neur-nr2f 169 - 33 (-)
Dicer1, Dcr-1 homolog (Drosophila)

GXP_1169523 [GXP_1169523] (1 - 663)
Tcl1, GXL_630467, GeneID: 690575, Rattus norvegicus chr. 6 — neur-nr2f 581 - 438 (-)
T-cell leukemia/lymphoma protein 1A (P14 TCL1 protein) (TCL1 oncogene) (TCL-1 protein)
neur-nr2f 488 - 367 (-)

**GXP_1169590** [GXP_1169590] (1 - 695)<br>**Amn_predicted**, GXL_630497, GeneID: 314459, Rattus norvegicus chr. 6<br>amnionless (predicted)			
**GXP_1417735** [GXP_1417735] (1 - 602)<br>**Baz2a_predicted**, GXL_630553, GeneID: 304601, Rattus norvegicus chr. 7<br>bromodomain adjacent to zinc finger domain, 2A (predicted)	neur-nr2f	113 - 229	(+)
**GXP_1417778** [GXP_1417778] (1 - 601)<br>**Ankrd24_predicted**, GXL_630592, GeneID: 299639, Rattus norvegicus chr. 7<br>ankyrin repeat domain 24 (predicted)	neur-nr2f	472 - 324	(-)
**GXP_1169937** [GXP_1169937] (1 - 628)<br>**Cfd**, GXL_630635, GeneID: 54249, Rattus norvegicus chr. 7<br>complement factor D (adipsin)	neur-nr2f	219 - 88	(-)
**GXP_1417880** [GXP_1417880] (1 - 601)<br>**Polrmt_predicted**, GXL_630636, GeneID: 299604, Rattus norvegicus chr. 7<br>polymerase (RNA) mitochondrial (DNA directed) (predicted)	neur-nr2f	569 - 420	(-)
**GXP_1417891** [GXP_1417891] (1 - 616)<br>**Brd4**, GXL_630649, GeneID: 362844, Rattus norvegicus chr. 7<br>bromodomain containing 4	neur-nr2f	204 - 347	(+)
**GXP_1169979** [GXP_1169979] (1 - 661)<br>**Wiz_predicted**, GXL_630650, GeneID: 314598, Rattus norvegicus chr. 7<br>widely-interspaced zinc finger motifs (predicted)	neur-nr2f	159 - 24	(-)
**GXP_1417954** [GXP_1417954] (1 - 607)<br>**Aldh1l2_predicted**, GXL_630708, GeneID: 299699, Rattus norvegicus chr. 7<br>aldehyde dehydrogenase 1 family, member L2 (predicted)	neur-nr2f	490 - 355	(-)
**GXP_1170104** [GXP_1170104] (1 - 780)<br>**Gnn**, GXL_630714, GeneID: 362863, Rattus norvegicus chr. 7<br>Grp94 neighboring nucleotidase	neur-nr2f	449 - 310	(-)
**GXP_1170191** [GXP_1170191] (1 - 601)<br>**Plxnc1_predicted**, GXL_630746, GeneID: 362873, Rattus norvegicus chr. 7<br>plexin C1 (predicted)	neur-nr2f	211 - 337	(+)
**GXP_1170200** [GXP_1170200] (1 - 602)<br>**Eea1_predicted**, GXL_630751, GeneID: 314764, Rattus norvegicus chr. 7<br>early endosome antigen 1 (predicted)	neur-nr2f	21 - 153	(+)
**GXP_1418234** [GXP_1418234] (1 - 601)<br>**Stat6_predicted**, GXL_630846, GeneID: 362896, Rattus norvegicus chr. 7<br>signal transducer and activator of transcription 6 (predicted)	neur-nr2f	537 - 400	(-)
**GXP_1170839** [GXP_1170839] (1 - 645)<br>**Tmprss6_predicted**, GXL_631008, GeneID: 315388, Rattus norvegicus chr. 7<br>transmembrane serine protease 6 (predicted)	neur-nr2f	578 - 441	(-)
	neur-nr2f	7 - 150	(+)

**GXP_1418503** [GXP_1418503] (1 - 602)			
**Kdelr3_predicted**, GXL_631021, GeneID: 315131, Rattus norvegicus chr. 7			
KDEL (Lys-Asp-Glu-Leu) endoplasmic reticulum protein retention receptor 3 (predicted)			
**GXP_1170915** [GXP_1170915] (1 - 602)			
**Cacna1i**, GXL_631027, GeneID: 56827, Rattus norvegicus chr. 7	neur-nr2f	329 - 179	(-)
calcium channel, voltage-dependent, alpha 1I subunit			
**GXP_1418547** [GXP_1418547] (1 - 601)			
**Mei1_predicted**, GXL_631047, GeneID: 315162, Rattus norvegicus chr. 7	neur-nr2f	536 - 414	(-)
meiosis defective 1 (predicted)			
**GXP_1418558** [GXP_1418558] (1 - 601)			
**Nfam1_predicted**, GXL_631054, GeneID: 362966, Rattus norvegicus chr. 7	neur-nr2f	88 - 236	(+)
NFAT activating protein with ITAM motif 1 (predicted)			
**GXP_1418631** [GXP_1418631] (1 - 608)			
**Plxnb2**, GXL_631089, GeneID: 315217, Rattus norvegicus chr. 7	neur-nr2f	464 - 338	(-)
plexin B2			
**GXP_1418780** [GXP_1418780] (1 - 602)			
**eplin**, GXL_631161, GeneID: 300228, Rattus norvegicus chr. 7	neur-nr2f	272 - 410	(+)
epithelial protein lost in neoplasm			
**GXP_1171359** [GXP_1171359] (1 - 609)			
**Itgb7**, GXL_631185, GeneID: 25713, Rattus norvegicus chr. 7	neur-nr2f	322 - 466	(+)
integrin, beta 7			
**GXP_1418911** [GXP_1418911] (1 - 775)			
**Pin1_predicted**, GXL_631256, GeneID: 298696, Rattus norvegicus chr. 8	neur-nr2f	273 - 149	(-)
protein (peptidyl-prolyl cis/trans isomerase) NIMA-interacting 1 (predicted)			
**GXP_1418936** [GXP_1418936] (1 - 602)			
**Smarca4**, GXL_631266, GeneID: 171379, Rattus norvegicus chr. 8	neur-nr2f	569 - 444	(-)
SWI/SNF related, matrix associated, actin dependent regulator of chromatin, subfamily a, member 4			
**GXP_1418943** [GXP_1418943] (1 - 602)			
**Dock6_predicted**, GXL_631268, GeneID: 367039, Rattus norvegicus chr. 8	neur-nr2f	422 - 572	(+)
dedicator of cytokinesis 6 (predicted)			
**GXP_1418944** [GXP_1418944] (1 - 602)			
**Dock6_predicted**, GXL_631268, GeneID: 367039, Rattus norvegicus chr. 8	neur-nr2f	300 - 453	(+)
dedicator of cytokinesis 6 (predicted)			
**GXP_1419007** [GXP_1419007] (1 - 601)			
**Kirrel3_predicted**, GXL_631308, GeneID: 315546, Rattus norvegicus chr. 8	neur-nr2f	116 - 261	(+)
kin of IRRE like 3 (Drosophila) (predicted)			
**GXP_1419071** [GXP_1419071] (1 - 601)			
**Sorl1**, GXL_631374, GeneID: 300652, Rattus norvegicus chr. 8	neur-nr2f	534 - 387	(-)
sortilin-related receptor, LDLR class A repeats-containing			
	neur-nr2f	161 - 33	(-)

**GXP_1419124** [GXP_1419124] (1 - 602)			
**Phldb1**, GXL_631391, GeneID: 171434, Rattus norvegicus chr. 8			
pleckstrin homology-like domain, family B, member 1			
**GXP_1419125** [GXP_1419125] (1 - 602)			
**Phldb1**, GXL_631391, GeneID: 171434, Rattus norvegicus chr. 8	neur-nr2f	244 - 94	(-)
pleckstrin homology-like domain, family B, member 1			
**GXP_1172182** [GXP_1172182] (1 - 1271)			
**Pif1**, GXL_631505, GeneID: 367645, Rattus norvegicus chr. 8	neur-nr2f	178 - 49	(-)
Pif1/Rrm3 DNA-helicase-like protein			
**GXP_1419515** [GXP_1419515] (1 - 602)			
**Myo5c_predicted**, GXL_631541, GeneID: 315820, Rattus norvegicus chr. 8	neur-nr2f	12 - 150	(+)
myosin VC (predicted)			
**GXP_1419636** [GXP_1419636] (1 - 602)			
**Xrn1_predicted**, GXL_631601, GeneID: 300944, Rattus norvegicus chr. 8	neur-nr2f	151 - 267	(+)
5'-3' exoribonuclease 1 (predicted)			
**GXP_1172538** [GXP_1172538] (1 - 786)			
**Mrpl3_predicted**, GXL_631642, GeneID: 300974, Rattus norvegicus chr. 8	neur-nr2f	442 - 585	(+)
mitochondrial ribosomal protein L3 (predicted)			
**GXP_1419941** [GXP_1419941] (1 - 680)			
**Slc22a14_predicted**, GXL_631721, GeneID: 316061, Rattus norvegicus chr. 8	neur-nr2f	585 - 470	(-)
solute carrier family 22 (organic cation transporter), member 14 (predicted)			
**GXP_1420010** [GXP_1420010] (1 - 601)			
**Fyco1_predicted**, GXL_631745, GeneID: 301085, Rattus norvegicus chr. 8	neur-nr2f	311 - 432	(+)
FYVE and coiled-coil domain containing 1 (predicted)			
**GXP_1420086** [GXP_1420086] (1 - 605)			
**Xpo5_predicted**, GXL_631815, GeneID: 363194, Rattus norvegicus chr. 9	neur-nr2f	512 - 367	(-)
exportin 5 (predicted)			
**GXP_1173254** [GXP_1173254] (1 - 704)			
**Ankrd39_predicted**, GXL_631894, GeneID: 367251, Rattus norvegicus chr. 9	neur-nr2f	81 - 234	(+)
ankyrin repeat domain 39 (predicted)			
**GXP_1420260** [GXP_1420260] (1 - 601)			
**Eif5b**, GXL_631905, GeneID: 308306, Rattus norvegicus chr. 9	neur-nr2f	451 - 579	(+)
eukaryotic translation initiation factor 5B			
**GXP_1420261** [GXP_1420261] (1 - 602)			
**Eif5b**, GXL_631905, GeneID: 308306, Rattus norvegicus chr. 9	neur-nr2f	121 - 249	(+)
eukaryotic translation initiation factor 5B			
**GXP_1420388** [GXP_1420388] (1 - 601)			
**Als2**, GXL_631978, GeneID: 363235, Rattus norvegicus chr. 9	neur-nr2f	312 - 198	(-)
amyotrophic lateral sclerosis 2 (juvenile) homolog (human)			
	neur-nr2f	460 - 323	(-)

GXP_1420436 [GXP_1420436] (1 - 644)
Klf7_predicted, GXL_631995, GeneID: 363243, Rattus norvegicus chr. 9
Kruppel-like factor 7 (ubiquitous) (predicted)

GXP_1173580 [GXP_1173580] (1 - 601)
Rpe_predicted, GXL_632004, GeneID: 316460, Rattus norvegicus chr. 9 — neur-nr2f 160 - 284 (+)
ribulose-5-phosphate-3-epimerase (predicted)

GXP_1173652 [GXP_1173652] (1 - 754)
Vil1_predicted, GXL_632025, GeneID: 316521, Rattus norvegicus chr. 9 — neur-nr2f 708 - 593 (-)
villin 1 (predicted)

GXP_1420657 [GXP_1420657] (1 - 602)
Centg2_predicted, GXL_632083, GeneID: 316611, Rattus norvegicus chr. 9 — neur-nr2f 533 - 383 (-)
centaurin, gamma 2 (predicted)

GXP_1420720 [GXP_1420720] (1 - 618)
Neu4_predicted, GXL_632110, GeneID: 316642, Rattus norvegicus chr. 9 — neur-nr2f 199 - 66 (-)
sialidase 4 (predicted)

GXP_1420848 [GXP_1420848] (1 - 602)
Klhl13, GXL_632210, GeneID: 313445, Rattus norvegicus chr. X — neur-nr2f 168 - 317 (+)
kelch-like 13 (Drosophila)

GXP_1420891 [GXP_1420891] (1 - 601)
Bcor_predicted, GXL_632250, GeneID: 317346, Rattus norvegicus chr. X — neur-nr2f 94 - 212 (+)
Bcl6 interacting corepressor (predicted)

GXP_1174351 [GXP_1174351] (1 - 601)
Irs4_predicted, GXL_632314, GeneID: 315350, Rattus norvegicus chr. X — neur-nr2f 51 - 189 (+)
insulin receptor substrate 4 (predicted)

GXP_1421136 [GXP_1421136] (1 - 607)
Stard8_predicted, GXL_632488, GeneID: 312113, Rattus norvegicus chr. X — neur-nr2f 391 - 540 (+)
START domain containing 8 (predicted)

GXP_1421206 [GXP_1421206] (1 - 601)
Tbx, GXL_632554, GeneID: 302369, Rattus norvegicus chr. X — neur-nr2f 235 - 91 (-)
T-box 22

GXP_1421236 [GXP_1421236] (1 - 601)
Taf7l_predicted, GXL_632615, GeneID: 363493, Rattus norvegicus chr. X — neur-nr2f 571 - 429 (-)
TAF7-like RNA polymerase II, TATA box binding protein (TBP)-associated factor (predicted)

GXP_1408308 [GXP_1408308] (1 - 601)
Mmp2, GXL_688272, GeneID: 81686, Rattus norvegicus chr. 19 — neur-nr2f 219 - 345 (+)
matrix metallopeptidase 2

GXP_1408686 [GXP_1408686] (1 - 605)
Gcsh, GXL_688281, GeneID: 171133, Rattus norvegicus chr. 19 — neur-nr2f 299 - 437 (+)
glycine cleavage system protein H (aminomethyl carrier)
neur-nr2f 205 - 68 (-)

**GXP_1410154** [GXP_1410154] (1 - 601)<br>**Eef2k**, GXL_688301, GeneID: 25435, Rattus norvegicus chr. 1<br>eukaryotic elongation factor-2 kinase			
**GXP_1411269** [GXP_1411269] (1 - 601)<br>**Tead3**, GXL_688309, GeneID: 294299, Rattus norvegicus chr. 20<br>TEA domain family member 3	neur-nr2f	538 - 407	(-)
**GXP_1414418** [GXP_1414418] (1 - 602)<br>**Oprl1**, GXL_688342, GeneID: 29256, Rattus norvegicus chr. 3<br>opioid receptor-like 1	neur-nr2f	288 - 415	(+)
**GXP_1415654** [GXP_1415654] (1 - 601)<br>**Asph_predicted**, GXL_688353, GeneID: 312981, Rattus norvegicus chr. 5<br>aspartate-beta-hydroxylase (predicted)	neur-nr2f	281 - 163	(-)
**GXP_1418148** [GXP_1418148] (1 - 695)<br>**Rap1b**, GXL_688374, GeneID: 171337, Rattus norvegicus chr. 7<br>RAS related protein 1b	neur-nr2f	264 - 117	(-)
**GXP_1418461** [GXP_1418461] (1 - 816)<br>**Pvalb**, GXL_688376, GeneID: 25269, Rattus norvegicus chr. 7<br>parvalbumin	neur-nr2f	378 - 242	(-)
**GXP_1419860** [GXP_1419860] (1 - 601)<br>**Pthr1**, GXL_688389, GeneID: 56813, Rattus norvegicus chr. 8<br>parathyroid hormone receptor 1	neur-nr2f	363 - 214	(-)
**GXP_1419995** [GXP_1419995] (1 - 604)<br>**Tgm4**, GXL_688391, GeneID: 64679, Rattus norvegicus chr. 8<br>transglutaminase 4 (prostate)	neur-nr2f	68 - 201	(+)

A total of 788 matches (731 non-redundant matches) was found in 726 sequences.

Sequences searched: 28203 (18846677 bp).

Evaluation of results

Model: neur-nr2f

Number of input genes: 696

GO category "biological_process"

Number of genes annotated in GO: 443
Number of significant GO groups found: 30

GO group	p-value	list of genes	GeneIDs

		# genes (observed)	# genes (expected)		
regulation of developmental process	0.000421303	24	11.37	Cntn4, Fzd1, Notch2, Klf10, Hes5, Dynll1, Inhba, Timp2, Ntn1, Arhgef18_predicted, Wnt7b, Tgfb2, Twsg1_predicted, Ntf3, Efna1, Ereg, Chrd, Acin1, Prlr, Pvrl1, Nell1, Myst3, Dicer1, Plxnb2	116658, 58868, 29492, 81813, 79225, 58945, 29200, 29543, 114523, 304193, 315196, 81809, 363294, 81737, 94268, 59325, 117275, 305884, 24684, 192183, 81733, 306571, 299284, 315217
regulation of cell differentiation	0.000508592	18	7.53	Cntn4, Fzd1, Notch2, Klf10, Hes5, Inhba, Timp2, Wnt7b, Tgfb2, Twsg1_predicted, Ntf3, Ereg, Chrd, Acin1, Prlr, Nell1, Myst3, Dicer1	116658, 58868, 29492, 81813, 79225, 29200, 29543, 315196, 81809, 363294, 81737, 59325, 117275, 305884, 24684, 81733, 306571, 299284
osteoblast differentiation	0.000536761	7	1.49	Fzd1, Wnt7b, Twsg1_predicted, Chrd, Gabbr1, Nell1, Pthr1	58868, 315196, 363294, 117275, 81657, 81733, 56813
negative regulation of cell differentiation	0.000663321	10	2.97	Cntn4, Notch2, Hes5, Inhba, Tgfb2, Twsg1_predicted, Ereg, Chrd, Myst3, Dicer1	116658, 29492, 79225, 29200, 81809, 363294, 59325, 117275, 306571, 299284
dopamine metabolic process	0.00094984	5	0.82	Cyp2d22, Tgfb2, Nr4a2, Sncaip_predicted, Th	171522, 81809, 54278, 307309, 25085
regulation of neurotransmitter levels	0.00106735	14	5.47	Rab3a, Stx1a, Lin7a, Cyp2d22, Tgfb2, Lin7c, Trh, Syt3, Nr4a2, Syt1, Sncaip_predicted, Ppfia3, Tph1, Th	25531, 116470, 85327, 171522, 81809, 60442, 25569, 25731, 54278, 25716, 307309, 140591, 24848, 25085
regulation of osteoblast differentiation	0.0012637	5	0.86	Fzd1, Wnt7b, Twsg1_predicted, Chrd, Nell1	58868, 315196, 363294, 117275, 81733
synaptic transmission	0.00147683	30	16.84	Cntn4, Lynx1_predicted, Htr1d, Syp, Rab3a, Kcnmb1, Gabrg2, Stx1a, Dlgh4, Lin7a, Cyp2d22, Htr3b, Fnta, Tgfb2, Lin7c, Acp2, Grm8, Ntf3, Trh, Clstn3, Syt3, Ntf5, Dlgap1, Sv2c, Nr4a2, Syt1, Sncaip_predicted, Ppfia3, Tph1, Th	116658, 300018, 25323, 24804, 25531, 29747, 29709, 116470, 29495, 85327, 171522, 58963, 25318, 81809, 60442, 24162, 60590, 81737, 25569, 171393, 25731, 25730, 65040, 29643, 54278, 25716, 307309, 140591, 24848, 25085
tissue remodeling	0.0014962	14	5.66	Fzd1, Klf10, Wnt7b, Csrp3, Tgfb2, Ptprv, Vegfa, Twsg1_predicted, Tcirg1, Chrd, Gabbr1, Nell1, Mmp2, Pthr1	58868, 81813, 315196, 117505, 81809, 64576, 83785, 363294, 293650, 117275, 81657, 81733, 81686, 56813
negative regulation of developmental process	0.00193896	11	3.98	Cntn4, Notch2, Hes5, Inhba, Ntn1, Tgfb2, Twsg1_predicted, Ereg, Chrd, Myst3, Dicer1	116658, 29492, 79225, 29200, 114523, 81809, 363294, 59325, 117275, 306571, 299284
stem cell development	0.00196501	3	0.29	Notch2, Myst3, Dicer1	29492, 306571, 299284
stem cell maintenance	0.00196501	3	0.29	Notch2, Myst3, Dicer1	29492, 306571, 299284
cell organization and biogenesis	0.00200744	96	72.91	Hsd3b7, Rock2, M6pr, Cntn4, Zfp384, Eif2ak3, Notch2, Arhgef2, Frap1, Dynll1, Hrasls_predicted, Tnk2, Ap2m1, Mid1, Syp, Phb, Rab3a, H2afy,	246211, 25537, 312689, 116658, 171018, 29702, 29492, 310635, 56718, 58945, 288025, 303882,

				Ntrk2, Prm2, Ntn1, Rasd1, Pxn, Arhgef18_predicted, Ramp2, Dlgh4, Wnt7b, Abcd3, Pola2, Emp3, Ap3b2_predicted, Notch4, Tgfb2, Csrp1, Abtb2, Acp2, Tep1, Vegfa, Tuba4a, Smarcc1_predicted, Ntf3, Nolc1, Abi2, Pfn2, Lmna, Efna1, Pelo, Mkks, Sycp2, Vps54, Sf3a1_predicted, Kdelr1, Arntl, Eif3s8, Taf6l_predicted, Sqstm1, Pcnt1, Spnb2, Acin1, Katnb1, Pacsin3, Eif2ak4_predicted, Pacsin2, Hdac7a, Pvrl1, Nfasc, Alox12_predicted, Ap1m1, Sgcz_predicted, Myst3, Arhgap26_predicted, Spire1_predicted, Ankrd27_predicted, Tubgcp5_predicted, Eed_predicted, Hps3_predicted, Mbnl1, Flg, Ntng2_predicted, Snap23, Mcm2_predicted, Sync_predicted, Eif4g3_predicted, Nkx2-9_predicted, Dicer1, Baz2a_predicted, Eea1_predicted, Kdelr3_predicted, Plxnb2, Smarca4, Sorl1, Xpo5_predicted, Eif5b, Als2, Vil1_predicted, Mmp2	116563, 54252, 24804, 25344, 25531, 29384, 25054, 25345, 114523, 64455, 360820, 304193, 58966, 29495, 315196, 25270, 85242, 81505, 308777, 406162, 81809, 29276, 171440, 24162, 64523, 83785, 316531, 301020, 81737, 64896, 286928, 81531, 60374, 94268, 294754, 311456, 83820, 286932, 305479, 361577, 29657, 293484, 309194, 113894, 287830, 305614, 305884, 291852, 311187, 114859, 124461, 84582, 192183, 116690, 287454, 306332, 364605, 306571, 307459, 307348, 361555, 308663, 293104, 310288, 282635, 24641, 311836, 64630, 312538, 362606, 298573, 299061, 299284, 304601, 314764, 315131, 315217, 171379, 300652, 363194, 308306, 363235, 316521, 81686
glial cell fate commitment	0.00229565	2	0.10	Ntf3, Smarca4	81737, 171379
glial cell fate determination	0.00229565	2	0.10	Ntf3, Smarca4	81737, 171379
transmission of nerve impulse	0.00246398	32	18.95	Cntn4, Lynx1_predicted, Htr1d, Syp, Rab3a, Kcnmb1, Gabrg2, Stx1a, Dlgh4, Lin7a, Cyp2d22, Tpo1, Htr3b, Fnta, Tgfb2, Lin7c, Acp2, Grm8, Ntf3, Trh, Clstn3, Syt3, Ntf5, Dlgap1, Mpdz, Sv2c, Nr4a2, Syt1, Sncaip_predicted, Ppfia3, Tph1, Th	116658, 300018, 25323, 24804, 25531, 29747, 29709, 116470, 29495, 85327, 171522, 170907, 58963, 25318, 81809, 60442, 24162, 60590, 81737, 25569, 171393, 25731, 25730, 65040, 29365, 29643, 54278, 25716, 307309, 140591, 24848, 25085
stem cell differentiation	0.00331434	3	0.34	Notch2, Myst3, Dicer1	29492, 306571, 299284
neurotransmitter metabolic process	0.00431841	6	1.58	Cyp2d22, Tgfb2, Nr4a2, Sncaip_predicted, Tph1, Th	171522, 81809, 54278, 307309, 24848, 25085
cell-cell signaling	0.00510852	44	29.64	Cntn4, Fzd1, Lynx1_predicted, Htr1d, Syp, Wnt5a, Rab3a, Inhba, Kcnmb1, Gabrg2, Ntn1, Stx1a, Ccl11, Dlgh4, Lin7a, Wnt7b, Cyp2d22, Tpo1, Htr3b, Fnta, Tgfb2, Lin7c, Acp2, Grm8, Ntf3, Trh, Clstn3, Syt3, Ntf5, Dlgap1, Mpdz, Sv2c, Ereg, Ins1, Nr4a2, Syt1, Grap2, Trp63, Sncaip_predicted, Ppfia3, Tph1, Nmb_predicted, Th, Wnt8b_predicted	116658, 58868, 300018, 25323, 24804, 64566, 25531, 29200, 29747, 29709, 114523, 116470, 29397, 29495, 85327, 315196, 171522, 170907, 58963, 25318, 81809, 60442, 24162, 60590, 81737, 25569, 171393, 25731, 25730, 65040, 29365, 29643, 59325, 24505, 54278, 25716, 366962, 246334, 307309, 140591, 24848, 308751, 25085, 293990
synaptic vesicle transport	0.00519383	7	2.16	Syp, Stx1a, Dlgh4, Lin7a, Syt3, Syt1, Eea1_predicted	24804, 116470, 29495, 85327, 25731, 25716, 314764
catecholamine metabolic process	0.00594228	5	1.20	Cyp2d22, Tgfb2, Nr4a2, Sncaip_predicted, Th	171522, 81809, 54278, 307309, 25085

phenol metabolic process	0.00594228	5	1.20	Cyp2d22, Tgfb2, Nr4a2, Sncaip_predicted, Th	171522, 81809, 54278, 307309, 25085
synaptic vesicle maturation	0.00655803	2	0.14	Syp, Dlgh4	24804, 29495
positive regulation of neurotransmitter secretion	0.00655803	2	0.14	Stx1a, Trh	116470, 25569
regulation of gene expression, epigenetic	0.0067307	6	1.73	H2afy, Cntn1, Il1rl1, Eed_predicted, Dicer1, Smarca4	29384, 117258, 25556, 293104, 299284, 171379
cell fate determination	0.00707275	5	1.25	Notch2, Notch4, Ntf3, Ntf5, Smarca4	29492, 406162, 81737, 25730, 171379
drug metabolic process	0.00741184	3	0.43	Cyp2d22, Udpgtr2, Cyp2c6	171522, 286954, 246070
neurotransmitter secretion	0.0078628	9	3.50	Rab3a, Stx1a, Lin7a, Lin7c, Trh, Syt3, Syt1, Sncaip_predicted, Ppfia3	25531, 116470, 85327, 60442, 25569, 25731, 25716, 307309, 140591
tissue morphogenesis	0.00880128	6	1.82	Tbx6_predicted, Tgfb2, Twsg1_predicted, Chrd, Trp63, Flg	365371, 81809, 363294, 117275, 246334, 24641
mesoderm formation	0.00934254	4	0.86	Tbx6_predicted, Tgfb2, Twsg1_predicted, Chrd	365371, 81809, 363294, 117275

GO category "molecular_function"

Number of genes annotated in GO: 465
Number of significant GO groups found: 9

GO group	p-value	# genes (observed)	# genes (expected)	list of genes	GeneIDs
protein binding	0.000943263	210	177.48	Tacc2, Ctsc, Tnfrsf1b, Rock2, Zfp384, Fzd1, Itpr1, S100a11, Lmo4, Kcnd3, Notch2, Arhgef2, Dlgap4, Pscdbp, Acvr1, Lynx1_predicted, Slc2a1, Pla2g2a, Frap1, Casp9, Camkk2, Dynll1, Tnk2, Clcn2, Ap2m1, Apg3l, Mid1, Syp, Phb, Myr8, Wnt5a, Rab3a, Ntrk2, Inhba, Fbp1, Stat3, Timp2, Snip, Gabrg2, Maml1_predicted, Ntn1, Rasd1, Atp1b2, Lnx2_predicted, Pxn, Stx1a, Serpine1, Ramp2, Ccl11, Flot2, Dlgh4, Aoc3, Lin7a, Nfe2, Wnt7b, Grip1, Msn, Abcd3, Pola2, Tagln, Csrp3, Notch4, Ltb, Cdon, Tgfb2, Dpt_predicted, Csrp1, Lhx9, Lin7c, Chgb, Acp2, Ptk2b, Pdlim2, Igfbp5, Vegfa, Twsg1_predicted, Tuba4a, Orc2l, Per2, Hspcb, Adm2, Csf2rb1, Smarcc1_predicted, Oldlr1, Nos3, Ntf3, Trh, Syt3, Nphs1, Ntf5, Nolc1, Il1rl1, Abi2, Bcs1l, Dlgap1, Mpdz, Dbccr1, Pfn2, Lmna, Efna1, Pde4dip, Rab25_predicted, Arfrp1, Rgs19,	309025, 25423, 156767, 25537, 171018, 58868, 25262, 445415, 362051, 65195, 29492, 310635, 286930, 311047, 79558, 300018, 24778, 29692, 56718, 58918, 83506, 58945, 303882, 29232, 116563, 171415, 54252, 24804, 25344, 192253, 64566, 25531, 25054, 29200, 24362, 25125, 29543, 56029, 29709, 303101, 114523, 64455, 24214, 360761, 360820, 116470, 24617, 58966, 29397, 83764, 29495, 29473, 85327, 366998, 315196, 84016, 81521, 25270, 85242, 25123, 117505, 406162, 361795, 50938, 81809, 289178, 29276, 289048, 60442, 24259, 24162, 50646,

				Angptl2, Mkks, Stau1, Plcb2, Pdgfra, Rpl5, Ereg, Atp5a1, Catna1, Nufip1, Gnrhr, Cd48, Rps6ka2, Map4k1_predicted, Arntl, Ganab_predicted, Ins1, Sqstm1, Map2k3, Thrap4, Slc4a1, Mx1, Chrd, Mospd3, Ncor2_predicted, Fln29, Spnb2, Acin1, Ppp3cc, Wbp4, Katnb1, Prlr, Tiparp_predicted, Gabbr1, Nr4a2, Pacsin3, Rnpc2, Foxp1, Adipor2, Wwp1, Syt1, Grap2, Pacsin2, Hdac7a, Pvrl1, Hes6, Irak1_predicted, Nell1, Trp63, Rab3gap2, Hr, Pias4, Sag, Rpa1, Itga3_predicted, Sfrs15, Zfp295_predicted, Rimbp2, Rfc5_predicted, Elk4_predicted, Zfp509_predicted, Smtn, Spred2, Pbk_predicted, Dock9, Ap1m1, Sgcz_predicted, Myst3, Dok3_predicted, Dsc1_predicted, Sncaip_predicted, Cd226_predicted, Brd7_predicted, Cotl1_predicted, Ppfia3, Tubgcp5_predicted, Nmb_predicted, Eed_predicted, Sf3b2_predicted, Wnt8b_predicted, Ccna2, Ppp2r4_predicted, Snap23, Mpp6_predicted, Mcm2_predicted, Plxnd1_predicted, Ipp_predicted, Sync_predicted, Eif4g3_predicted, Mthfr_predicted, Ddef2_predicted, Mgea6_predicted, Ches1_predicted, Dicer1, Ankrd24_predicted, Brd4, Plxnc1_predicted, Eea1_predicted, Plxnb2, Itgb7, Pin1_predicted, Smarca4, Eif5b, Klf7_predicted, Rpe_predicted, Rap1b	290354, 25285, 83785, 363294, 316531, 301430, 63840, 301252, 399475, 171081, 301020, 140914, 24600, 81737, 25569, 25731, 64563, 25730, 64896, 25556, 286928, 301514, 65040, 29365, 140610, 81531, 60374, 94268, 64183, 310632, 117051, 59293, 171100, 311456, 84496, 85240, 25267, 81763, 59325, 65262, 307505, 364430, 81668, 245962, 117269, 292763, 29657, 293721, 24505, 113894, 303200, 619436, 24779, 24575, 117275, 288557, 360801, 114635, 305614, 305884, 171378, 114765, 291852, 24684, 310467, 81657, 54278, 311187, 362251, 297480, 312670, 297930, 25716, 366962, 124461, 84582, 192183, 316626, 363520, 81733, 246334, 289350, 60563, 362827, 25539, 287524, 360606, 245924, 304056, 266780, 304528, 304786, 305428, 289734, 305539, 290326, 259237, 306332, 364605, 306571, 306760, 291759, 307309, 307199, 361374, 361422, 140591, 308663, 308751, 293104, 293671, 293990, 114494, 362102, 64630, 362359, 312538, 312652, 298439, 362606, 298573, 362657, 362719, 299078, 314374, 299284, 299639, 362844, 362873, 314764, 315217, 25713, 298696, 171379, 308306, 363243, 316460, 171337
transferase activity, transferring hexosyl groups	0.00160576	12	4.48	Pomgnt1, Fut4, Pygl, Fut11, B3gnt6_predicted, Ugt1a1, Udpgtr2, Ugt2a1, Alg5, B4galt6, Galnt2_predicted, Mtap_predicted	362567, 60670, 64035, 286971, 293667, 24861, 286954, 63867, 295051, 65196, 292090, 298227
binding	0.00301622	291	262.01	Tacc2, Tbx6_predicted, Ctsc, Tnfrsf1b, Rock2, Hpcal1, Zfp384, Fzd1, Itpr1, S100a11, Lmo4, Kcnd3, Notch2, Arhgef2, Dlgap4, Rcn1_predicted, Pscdbp, C8g_predicted, Acvr1, Ptgds, Lynx1_predicted, Myf6, Eef2, Slc2a1, Pla2g2a, Frap1, Rpl22, Casp9, Hes5, Camkk2, Zcchc8_predicted, Dynll1, Tnk2, Clcn2, Ap2m1, Apg3l, Abcb10, Rbm10, Mid1, Syp, Phb, Ppp1r3b, Myr8, Wnt5a, Rab3a, H2afy, Ntrk2, Inhba, Fbp1, Stat3, Timp2, Prm2, Snip, Gabrg2, Maml1_predicted, Ntn1, Rasd1, Atp1b2, Lnx2_predicted, Pxn, Stx1a, Gtf2ird1, Serpine1, Ramp2, Ccl11, Flot2, Col23a1, Dlgh4, Aoc3, Lin7a, Nfe2, Wnt7b, Grip1, Cyp2d22, Avpr2, Msn, Fhl1, Abcd3, Gstm1, Pola2, Tagln, Asb2, Anpep, Csrp3, Inppl1, Notch4, Ltb, Cdon, Ireb2, Tgfb2, Dpt_predicted, Csrp1, Lbr, Lhx9, Fkbp1a, Lin7c, Chgb, Acp2, Ptk2b, Pdlim2, Igfbp5, Vegfa, Twsg1_predicted, Tuba4a, Orc2l, Per2, Hspcb, Adm2, Csf2rb1, Cntn1, Smarcc1_predicted, C3ar1, Oldlr1, Nos3, Ntf3,	309025, 365371, 25423, 156767, 25537, 50871, 171018, 58868, 25262, 445415, 362051, 65195, 29492, 310635, 286930, 362182, 311047, 296545, 79558, 25526, 300018, 25714, 29565, 24778, 29692, 56718, 81768, 58918, 79225, 83506, 288661, 58945, 303882, 29232, 116563, 171415, 361439, 64510, 54252, 24804, 25344, 192280, 192253, 64566, 25531, 29384, 25054, 29200, 24362, 25125, 29543, 25345, 56029, 29709, 303101, 114523, 64455, 24214, 360761, 360820, 116470, 246770, 24617, 58966, 29397, 83764, 353303, 29495, 29473, 85327, 366998, 315196, 84016, 171522, 25108, 81521, 25177, 25270, 24423, 85242, 25123, 299266, 81641,

				Trh, Syt3, Nphs1, Pglyrp1, Ntf5, Nolc1, Il1rl1, Abi2, Bcs1l, Dlgap1, Dio1, Mpdz, Dbccr1, Atpbd1b_predicted, Pfn2, Lmna, Efna1, Pde4dip, Rab25_predicted, Arfrp1, Rgs19, Angptl2, Mkks, Stau1, Plcb2, Sycp2, Pdgfra, Rpl5, Ereg, Atp5a1, Cxxc5, Catna1, Tdrd3, Nufip1, Gnrhr, Sf3a1_predicted, Cd48, Rps6ka2, Map4k1_predicted, Kdelr1, Arntl, Eif3s8, Taf6l_predicted, Ganab_predicted, Ins1, Sqstm1, Map2k3, Thrap4, Slc4a1, Sox9, Srp68_predicted, Mx1, Chrd, Mospd3, Ncor2_predicted, Fln29, Spnb2, Acin1, Ppp3cc, Wbp4, Nol4_predicted, Katnb1, Aprt_predicted, Prlr, Tiparp_predicted, Gabbr1, Nr4a2, Pacsin3, Elf5_predicted, Rnpc2, Foxp1, Adipor2, Adhfe1, Wwp1, Dspg3_predicted, Syt1, Ankrd46, Grap2, Pacsin2, Hdac7a, Pvrl1, Pcbp4_predicted, Hes6, Irak1_predicted, Nell1, Trp63, Rab3gap2, Hr, Slc14a2, Efcbp2, Pias4, Sag, Trim16_predicted, Rpa1, Itga3_predicted, Cdc6_predicted, Sfrs15, Prdm15_predicted, Zfp295_predicted, Lass4_predicted, Zfp68_predicted, Rimbp2, Fbxl10, Rfc5_predicted, Elk4_predicted, Zfp509_predicted, Smtn, Spred2, Pbk_predicted, Dock9, Ap1m1, Sgcz_predicted, Myst3, Dok3_predicted, Zfp169_predicted, Dsc1_predicted, Sncaip_predicted, Cd226_predicted, Nkd1_predicted, Brd7_predicted, Tnpo2_predicted, Rbm35b_predicted, Cotl1_predicted, Snai3_predicted, Ppfia3, Tubgcp5_predicted, Nmb_predicted, Eed_predicted, Th, Sf3b2_predicted, Wnt8b_predicted, Ccna2, Mbnl1, Ppp2r4_predicted, Ciz1_predicted, Hoxd4_predicted, Ehf_predicted, Pla2g4b_predicted, Snap23, Epb4.2_predicted, Zfp217_predicted, Mpp6_predicted, Mcm2_predicted, Plxnd1_predicted, Mysm1_predicted, Ipp_predicted, Sync_predicted, Eif4g3_predicted, Mthfr_predicted, Centb5_predicted, Ddef2_predicted, Mgea6_predicted, Ches1_predicted, Dicer1, Baz2a_predicted, Ankrd24_predicted, Polrmt_predicted, Brd4, Plxnc1_predicted, Eea1_predicted, Stat6_predicted, Plxnb2, Itgb7, Pin1_predicted, Smarca4, Mrpl3_predicted, Xpo5_predicted, Eif5b, Klf7_predicted, Rpe_predicted, Mmp2, Eef2k, Oprl1, Asph_predicted, Rap1b, Pvalb	117505, 65038, 406162, 361795, 50938, 64831, 81809, 289178, 29276, 89789, 289048, 25639, 60442, 24259, 24162, 50646, 290354, 25285, 83785, 363294, 316531, 301430, 63840, 301252, 399475, 171081, 117258, 301020, 84007, 140914, 24600, 81737, 25569, 25731, 64563, 84387, 25730, 64896, 25556, 286928, 301514, 65040, 25430, 29365, 140610, 362614, 81531, 60374, 94268, 64183, 310632, 117051, 59293, 171100, 311456, 84496, 85240, 83820, 25267, 81763, 59325, 65262, 291670, 307505, 306066, 364430, 81668, 305479, 245962, 117269, 292763, 361577, 29657, 293484, 309194, 293721, 24505, 113894, 303200, 619436, 24779, 140586, 363707, 24575, 117275, 288557, 360801, 114635, 305614, 305884, 171378, 114765, 307553, 291852, 292072, 24684, 310467, 81657, 54278, 311187, 366142, 362251, 297480, 312670, 362474, 297930, 314772, 25716, 299982, 366962, 124461, 84582, 192183, 363133, 316626, 363520, 81733, 246334, 289350, 60563, 54302, 170928, 362827, 25539, 303214, 287524, 360606, 360621, 245924, 304054, 304056, 304208, 304337, 266780, 304495, 304528, 304786, 305428, 289734, 305539, 290326, 259237, 306332, 364605, 306571, 306760, 306816, 291759, 307309, 307199, 364952, 361374, 304670, 307810, 361422, 307919, 140591, 308663, 308751, 293104, 25085, 293671, 293990, 114494, 282635, 362102, 296639, 288153, 295965, 311341, 64630, 362202, 311764, 362359, 312538, 312652, 298247, 298439, 362606, 298573, 362657, 313772, 362719, 299078, 314374, 299284, 304601, 299639, 299604, 362844, 362873, 314764, 362896, 315217, 25713, 298696, 171379, 300974, 363194, 308306, 363243, 316460, 81686, 25435, 29256, 312981, 171337, 25269
protein dimerization activity	0.00338682	22	11.75	S100a11, Notch2, Dynll1, Stat3, Stx1a, Ramp2, Aoc3, Pola2, Notch4, Tgfb2, Vegfa, Tuba4a, Pdgfra, Ereg, Katnb1, Prlr, Foxp1, Adipor2, Pvrl1, Rab3gap2, Ppp2r4_predicted, Eea1_predicted	445415, 29492, 58945, 25125, 116470, 58966, 29473, 85242, 406162, 81809, 83785, 316531, 25267, 59325, 291852, 24684, 297480, 312670, 192183, 289350, 362102, 314764

transferase activity, transferring glycosyl groups	0.00576501	14	6.55	Pomgnt1, Fut4, Pygl, Fut11, B3gnt6_predicted, Ugt1a1, Udpgtr2, Ugt2a1, Upp1, Aprt_predicted, Alg5, B4galt6, Galnt2_predicted, Mtap_predicted	362567, 60670, 64035, 286971, 293667, 24861, 286954, 63867, 289801, 292072, 295051, 65196, 292090, 298227
eukaryotic translation initiation factor 2alpha kinase activity	0.00660461	2	0.14	Eif2ak3, Eif2ak4_predicted	29702, 114859
neurotrophin p75 receptor binding	0.00660461	2	0.14	Ntf3, Ntf5	81737, 25730
protein heterodimerization activity	0.00697578	14	6.69	Notch2, Stx1a, Ramp2, Pola2, Notch4, Tgfb2, Tuba4a, Ereg, Katnb1, Foxp1, Adipor2, Pvrl1, Rab3gap2, Ppp2r4_predicted	29492, 116470, 58966, 85242, 406162, 81809, 316531, 59325, 291852, 297480, 312670, 192183, 289350, 362102
growth factor binding	0.00949646	7	2.41	Acvr1, Ntrk2, Igfbp5, Csf2rb1, Ntf3, Pdgfra, Ins1	79558, 25054, 25285, 171081, 81737, 25267, 24505

GO category "cellular_component"

Number of genes annotated in GO: 435
Number of significant GO groups found: 9

GO group	p-value	# genes (observed)	# genes (expected)	list of genes	GeneIDs
synapse	0.00068348	14	5.23	Itpr1, Slc32a1, Dlgap4, Syp, Dlgh4, Lin7a, Lin7c, Clstn3, Syt3, Dlgap1, Syt1, Pvrl1, Sncaip_predicted, Sync_predicted	25262, 83612, 286930, 24804, 29495, 85327, 60442, 171393, 25731, 65040, 25716, 192183, 307309, 362606
intracellular part	0.00125542	241	209.77	Tbx6_predicted, Ctsc, Pde4b, Rock2, M6pr, Zfp384, Itpr1, Eif2ak3, S100a11, Lmo4, Notch2, Rcn1_predicted, Pscdbp, Ptgds, Myf6, Fgf22, Slc2a1, Frap1, Nt5c1a_predicted, Rpl22, Casp9, Dynll1, Cyb5, Hrasls_predicted, Tnk2, Clcn2, Ap2m1, Apg3l, Ldhd, Mvd, Abcb10, Rbm10, Mid1, Syp, Phb, Ppp1r3b, Myr8, Rab3a, H2afy, Nmt2, Ntrk2, Fbp1, Stat3, Timp2, Prm2, Snip, Gabrg2, Maml1_predicted, Rasd1, Atp1b2, Tekt1, Pxn, Stx1a, Gtf2ird1, Ramp2, Dlgh4, Nfe2, Cyp2d22, Avpr2, Msn, Abcd3, Pola2, Mapkapk3, Fnta, Ap3b2_predicted, Anpep, Csrp3, Inppl1, Cln3, Cuzd1, Notch4, Ireb2, Tgfb2, Csrp1, Lbr, Ptpn7, Entpd6, Chgb, Acp2, Tep1, Ndrg2, Vegfa, Tuba4a, Orc2l, Per2, Hspcb, Smarcc1_predicted, Slc35b4_predicted, Opn1sw, Nos3, Ntf3, Trh, Ckm, Slc27a5, B3gnt6_predicted, Nolc1, Slc29a2, Ngep, Abi2, Bcs1l, Mrpl37, Pfn2, Sv2c, Lmna, Pde4dip, Pelo, Rgs19, Stau1, Plcb2, Sycp2, Rpl5, Atp5a1, Txnl1, Acaa2, Sucla2_predicted, Nufip1, Spergen2, Sf3a1_predicted, Rps6ka2, Kdelr1, Arntl, Eif3s8, Tcirg1, Taf6l_predicted, Ganab_predicted, Poll, Sqstm1, Thrap4, Vat1, Slc4a1, Sox9, Srp68_predicted, Mx1, Ncor2_predicted, Spnb2, Acin1, Ppp3cc, Wbp4, Actr8_predicted,	365371, 25423, 24626, 25537, 312689, 171018, 25262, 29702, 445415, 362051, 29492, 362182, 311047, 25526, 25714, 170579, 24778, 56718, 313574, 81768, 58918, 58945, 64001, 288025, 303882, 29232, 116563, 171415, 307858, 81726, 361439, 64510, 54252, 24804, 25344, 192280, 192253, 25531, 29384, 291318, 25054, 24362, 25125, 29543, 25345, 56029, 29709, 303101, 64455, 24214, 85270, 360820, 116470, 246770, 58966, 29495, 366998, 171522, 25108, 81521, 25270, 85242, 315994, 25318, 308777, 81641, 117505, 65038, 293485, 117179, 406162, 64831, 81809, 29276, 89789, 246781, 85260, 24259, 24162, 64523, 171114,

				Nol4_predicted, Wdr33_predicted, Katnb1, Txnl4b, Aprt_predicted, Gabbr1, Nr4a2, Pacsin3, Elf5_predicted, Rnpc2, Tnnc2, Foxp1, Lactb2, Wwp1, Syt1, Grap2, Pacsin2, Hdac7a, Gup1_predicted, Hes6, Nell1, Trp63, Hr, Efcbp2, Pias4, Sag, Zfp287_predicted, Trim16_predicted, Alox12_predicted, Rpa1, Psmd11_predicted, Cdc6_predicted, Prdm15_predicted, Lass4_predicted, Fbxl10, Rfc5_predicted, Smtn, Spred2, Pck2_predicted, Adprtl1, Ap1m1, Sgcz_predicted, Myst3, Dok3_predicted, Zfp169_predicted, Sncaip_predicted, Spire1_predicted, Brd7_predicted, Tnpo2_predicted, Snai3_predicted, Galnt2_predicted, Rnaset2_predicted, Ankrd27_predicted, Tph1, Tubgcp5_predicted, Mkrn3_predicted, Eed_predicted, Mrvi1_predicted, Th, Sf3b2_predicted, Wnt8b_predicted, Hps3_predicted, Ccna2, Mbnl1, Flg, Brd3_predicted, Ppp2r4_predicted, Ciz1_predicted, Hoxd4_predicted, Ehf_predicted, Pla2g4b_predicted, Snap23, Epb4.2_predicted, Alms1_predicted, Mcm2_predicted, Mysm1_predicted, Ipp_predicted, Sync_predicted, Eif4g3_predicted, Centb5_predicted, Ches1_predicted, Baz2a_predicted, Polrmt_predicted, Brd4, Eea1_predicted, Stat6_predicted, Kdelr3_predicted, Itgb7, Pin1_predicted, Smarca4, Xrn1_predicted, Mrpl3_predicted, Als2, Vil1_predicted, Neu4_predicted, Mmp2, Gcsh, Eef2k, Tead3, Asph_predicted, Pvalb, Pthr1	83785, 316531, 301430, 63840, 301252, 301020, 296969, 81644, 24600, 81737, 25569, 24265, 79111, 293667, 64896, 65194, 367318, 286928, 301514, 56281, 81531, 29643, 60374, 64183, 294754, 59293, 84496, 85240, 83820, 81763, 65262, 140922, 170465, 361071, 364430, 305491, 305479, 117269, 361577, 29657, 293484, 293650, 309194, 293721, 361767, 113894, 619436, 287721, 24779, 140586, 363707, 24575, 360801, 305614, 305884, 171378, 114765, 361107, 307553, 307524, 291852, 292008, 292072, 81657, 54278, 311187, 366142, 362251, 296369, 297480, 297768, 297930, 25716, 366962, 124461, 84582, 301073, 316626, 81733, 246334, 60563, 170928, 362827, 25539, 303212, 303214, 287454, 287524, 303353, 360621, 304054, 304208, 304495, 304528, 289734, 305539, 361042, 361046, 306332, 364605, 306571, 306760, 306816, 307309, 307348, 361374, 304670, 307919, 292090, 292306, 361555, 24848, 308663, 292988, 293104, 308899, 25085, 293671, 293990, 310288, 114494, 282635, 24641, 362092, 362102, 296639, 288153, 295965, 311341, 64630, 362202, 297408, 312538, 298247, 298439, 362606, 298573, 313772, 314374, 304601, 299604, 362844, 314764, 362896, 315131, 25713, 298696, 171379, 300944, 300974, 363235, 316521, 316642, 81686, 171133, 25435, 294299, 312981, 25269, 56813
synaptosome	0.00138841	7	1.73	Syp, Dlgh4, Lin7c, Acp2, Pvrl1, Itga3_predicted, Als2	24804, 29495, 60442, 24162, 192183, 360606, 363235
intracellular	0.00188359	245	215.05	Tbx6_predicted, Ctsc, Pde4b, Rock2, M6pr, Zfp384, Itpr1, Eif2ak3, S100a11, Lmo4, Notch2, Rcn1_predicted, Pscdbp, Ptgds, Myf6, Fgf22, Slc2a1, Frap1, Nt5c1a_predicted, Rpl22, Casp9, Camkk2, Dynll1, Cyb5, Hrasls_predicted, Tnk2, Clcn2, Ap2m1, Apg3l, Ldhd, Mvd, Abcb10, Rbm10, Mid1, Syp, Phb, Ppp1r3b, Myr8, Rab3a, H2afy, Nmt2, Ntrk2, Fbp1, Stat3, Timp2, Prm2, Snip, Gabrg2, Maml1_predicted, Rasd1, Atp1b2, Tekt1, Pxn, Stx1a, Gtf2ird1, Ramp2, Dlgh4, Nfe2, Cyp2d22, Avpr2, Msn, Abcd3, Pola2, Mapkapk3, Fnta, Ap3b2_predicted, Anpep, Csrp3, Inppl1, Cln3, Cuzd1, Notch4, Ireb2, Tgfb2, Csrp1, Lbr, Ptpn7, Entpd6, Chgb, Acp2, Tep1, Ndrg2, Vegfa, Tuba4a, Orc2l,	365371, 25423, 24626, 25537, 312689, 171018, 25262, 29702, 445415, 362051, 29492, 362182, 311047, 25526, 25714, 170579, 24778, 56718, 313574, 81768, 58918, 83506, 58945, 64001, 288025, 303882, 29232, 116563, 171415, 307858, 81726, 361439, 64510, 54252, 24804, 25344, 192280, 192253, 25531, 29384, 291318, 25054, 24362, 25125, 29543,

				Per2, Hspcb, Smarcc1_predicted, Slc35b4_predicted, Opn1sw, Nos3, Ntf3, Trh, Ckm, Slc27a5, B3gnt6_predicted, Nolc1, Slc29a2, Ngep, Abi2, Bcs1l, Mrpl37, Pfn2, Sv2c, Lmna, Pde4dip, Pelo, Rgs19, Stau1, Plcb2, Sycp2, Rpl5, Atp5a1, Txnl1, Acaa2, Sucla2_predicted, Nufip1, Spergen2, Sf3a1_predicted, Rps6ka2, Kdelr1, Arntl, Eif3s8, Tcirg1, Taf6l_predicted, Ganab_predicted, Poll, Sqstm1, Thrap4, Vat1, Slc4a1, Sox9, Srp68_predicted, Mx1, Ncor2_predicted, Spnb2, Acin1, Ppp3cc, Wbp4, Actr8_predicted, Nol4_predicted, Wdr33_predicted, Katnb1, Txnl4b, Aprt_predicted, Gabbr1, Nr4a2, Pacsin3, Elf5_predicted, Eif2ak4_predicted, Rnpc2, Tnnc2, Foxp1, Lactb2, Wwp1, Syt1, Grap2, Pacsin2, Hdac7a, Gup1_predicted, Hes6, Nell1, Trp63, Hr, Efcbp2, Pias4, Sag, Zfp287_predicted, Trim16_predicted, Alox12_predicted, Rpa1, Psmd11_predicted, Cdc6_predicted, Prdm15_predicted, Lass4_predicted, Zfp68_predicted, Fbxl10, Rfc5_predicted, Smtn, Spred2, Pck2_predicted, Adprtl1, Ap1m1, Sgcz_predicted, Myst3, Dok3_predicted, Zfp169_predicted, Sncaip_predicted, Spire1_predicted, Brd7_predicted, Tnpo2_predicted, Snai3_predicted, Galnt2_predicted, Rnaset2_predicted, Ankrd27_predicted, Tph1, Tubgcp5_predicted, Mkrn3_predicted, Eed_predicted, Mrvi1_predicted, Th, Sf3b2_predicted, Wnt8b_predicted, Hps3_predicted, Ccna2, Mbnl1, Flg, Brd3_predicted, Ppp2r4_predicted, Ciz1_predicted, Hoxd4_predicted, Ehf_predicted, Pla2g4b_predicted, Snap23, Epb4.2_predicted, Alms1_predicted, Mcm2_predicted, Mysm1_predicted, Ipp_predicted, Sync_predicted, Eif4g3_predicted, Centb5_predicted, Ches1_predicted, Dicer1, Baz2a_predicted, Polrmt_predicted, Brd4, Eea1_predicted, Stat6_predicted, Kdelr3_predicted, Itgb7, Pin1_predicted, Smarca4, Xrn1_predicted, Mrpl3_predicted, Als2, Vil1_predicted, Neu4_predicted, Mmp2, Gcsh, Eef2k, Tead3, Asph_predicted, Pvalb, Pthr1	25345, 56029, 29709, 303101, 64455, 24214, 85270, 360820, 116470, 246770, 58966, 29495, 366998, 171522, 25108, 81521, 25270, 85242, 315994, 25318, 308777, 81641, 117505, 65038, 293485, 117179, 406162, 64831, 81809, 29276, 89789, 246781, 85260, 24259, 24162, 64523, 171114, 83785, 316531, 301430, 63840, 301252, 301020, 296969, 81644, 24600, 81737, 25569, 24265, 79111, 293667, 64896, 65194, 367318, 286928, 301514, 56281, 81531, 29643, 60374, 64183, 294754, 59293, 84496, 85240, 83820, 81763, 65262, 140922, 170465, 361071, 364430, 305491, 305479, 117269, 361577, 29657, 293484, 293650, 309194, 293721, 361767, 113894, 619436, 287721, 24779, 140586, 363707, 24575, 360801, 305614, 305884, 171378, 114765, 361107, 307553, 307524, 291852, 292008, 292072, 81657, 54278, 311187, 366142, 114859, 362251, 296369, 297480, 297768, 297930, 25716, 366962, 124461, 84582, 301073, 316626, 81733, 246334, 60563, 170928, 362827, 25539, 303212, 303214, 287454, 287524, 303353, 360621, 304054, 304208, 304337, 304495, 304528, 289734, 305539, 361042, 361046, 306332, 364605, 306571, 306760, 306816, 307309, 307348, 361374, 304670, 307919, 292090, 292306, 361555, 24848, 308663, 292988, 293104, 308899, 25085, 293671, 293990, 310288, 114494, 282635, 24641, 362092, 362102, 296639, 288153, 295965, 311341, 64630, 362202, 297408, 312538, 298247, 298439, 362606, 298573, 313772, 314374, 299284, 304601, 299604, 362844, 314764, 362896, 315131, 25713, 298696, 171379, 300944, 300974, 363235, 316521, 316642, 81686, 171133, 25435, 294299, 312981, 25269, 56813
cell projection	0.00190982	29	16.37	Cntn4, S100a11, Kcnd3, Arhgef2, Slc34a3, Tnk2, Clcn2, Timp2, Pxn, Msn, Tgfb2, Fkbp1a, Mep1a, Opn1sw, Grm8, Abi2, Stau1, Catna1, Katnb1, Syt1, Pvrl1, Nfasc, Tph1, Th, Trpm6, Alms1_predicted, Vil1_predicted, Pvalb, Pthr1	116658, 445415, 65195, 310635, 246234, 303882, 29232, 29543, 360820, 81521, 81809, 25639, 25684, 81644, 60590,

					286928, 84496, 307505, 291852, 25716, 192183, 116690, 24848, 25085, 293874, 297408, 316521, 25269, 56813
neuron projection	0.00285938	17	8.06	Cntn4, Kcnd3, Arhgef2, Tnk2, Timp2, Tgfb2, Fkbp1a, Grm8, Abi2, Stau1, Katnb1, Syt1, Pvrl1, Nfasc, Tph1, Th, Pvalb	116658, 65195, 310635, 303882, 29543, 81809, 25639, 60590, 286928, 84496, 291852, 25716, 192183, 116690, 24848, 25085, 25269
secretory granule	0.00493614	8	2.69	Itpr1, Ap2m1, Stx1a, Cuzd1, Tgfb2, Chgb, Trh, Syt1	25262, 116563, 116470, 117179, 81809, 24259, 25569, 25716
plasma membrane	0.00622192	96	75.73	Cntn4, Zfp384, Itpr1, Kcnd3, Notch2, Slc32a1, Slc34a3, Acvr1, Lynx1_predicted, Slc2a1, Htr1d, Clcn2, Kcnj6, Phb, Testin, Ntrk2, Stat3, Cd7_predicted, Gabrg2, Atp1b2, Pxn, Stx1a, Ramp2, Flot2, Dlgh4, Aoc3, Lin7a, Grip1, Avpr2, Msn, Htr3b, Anpep, Cln3, Prss8, Notch4, Ltb, Xtrp3, Lin7c, Ptpra, Slc22a7, Mep1a, Gpr1, Csf2rb1, Opn1sw, Oldlr1, Ntf3, Trh, Nphs1, Kcnc3, Slc29a2, Ngep, Ugt1a1, Abi2, Mpdz, Ptprf, Pdgfra, Ereg, Catna1, Gnrhr, Cd48, Tcirg1, Slc4a1, Spnb2, Insrr, Gabbr1, Pvrl1, Nfasc, Alox12_predicted, Tm4sf5_predicted, Itga3_predicted, Spred2, Sgcz_predicted, Dsc1_predicted, Cd226_predicted, Slc12a7, Trpm4, Trpm6, Clca2_predicted, Ntng2_predicted, Snap23, Epb4.2_predicted, Mpp6_predicted, Plxnd1_predicted, Sync_predicted, Epha8, Plxnc1_predicted, Eea1_predicted, Tmprss6_predicted, Cacna1i, Nfam1_predicted, Itgb7, Kirrel3_predicted, Sorl1, Slc22a14_predicted, Oprl1, Pthr1	116658, 171018, 25262, 65195, 29492, 83612, 246234, 79558, 300018, 24778, 25323, 29232, 25743, 25344, 286916, 25054, 25125, 303747, 29709, 24214, 360820, 116470, 58966, 83764, 29495, 29473, 85327, 84016, 25108, 81521, 58963, 81641, 293485, 192107, 406162, 361795, 113918, 60442, 25167, 89776, 25684, 25457, 171081, 81644, 140914, 81737, 25569, 64563, 117101, 65194, 367318, 24861, 286928, 29365, 360406, 25267, 59325, 307505, 81668, 245962, 293650, 24779, 305614, 60663, 81657, 192183, 116690, 287454, 303256, 360606, 305539, 364605, 291759, 307199, 308069, 171143, 293874, 308016, 311836, 64630, 362202, 362359, 312652, 362606, 60589, 362873, 314764, 315388, 56827, 362966, 25713, 315546, 300652, 316061, 29256, 56813
nuclear origin of replication recognition complex	0.00656515	2	0.14	Orc2l, Mcm2_predicted	301430, 312538

Extraction Options

Sequence Extraction

Selected elements with start/end ± bp
Complete sequence reverse complement
in FASTA format GenBank format

GeneID Extraction

Excel Extraction

	**Compare results**
**Enter GeneIDs**	(separated by spaces, returns, or commas)
	**Further Evaluation of Matches**
**Search PubMed for**	**("gene name") AND**<br>(where "gene name" is automatically extracted from the description lines)<br>**Note:** This works only for annotated genomic DNA sequences from eukaryotes!

For comments, questions, or bug reports, please contact support@genomatix.de.

© Genomatix Software GmbH 1998-2008 - All rights reserved.

GEMS Launcher 4.6.1

License Agreement
